# Seasonal Sheep Grazing Does Not Enhance Stable or Total Soil Carbon Stocks in a Long‐Term Calcareous Grassland Experiment

**DOI:** 10.1002/ece3.71582

**Published:** 2025-06-30

**Authors:** David Encarnation, Deborah Ashworth, Richard Bardgett, Mona Edwards, Clive Hambler, Jeppe Kristensen, Andrew Hector

**Affiliations:** ^1^ Department of Plant Sciences University of Cambridge Cambridge UK; ^2^ Department of Earth and Environmental Sciences The University of Manchester Manchester UK; ^3^ Lancaster Environment Centre Lancaster University Lancaster UK; ^4^ School of Geography University of Oxford Oxford UK; ^5^ Department of Biology University of Oxford Oxford UK; ^6^ SustainScapes—Centre for Sustainable Landscapes Under Global Change, Department of Biology Aarhus University Aarhus Denmark; ^7^ Leverhulme Centre for Nature Recovery University of Oxford Oxford UK

**Keywords:** carbon persistence, climate change, grazing, mineral‐associated organic carbon, soil carbon

## Abstract

Soils hold a globally important carbon pool that is generally more persistent than the carbon stored in plant biomass. However, soil carbon is becoming increasingly vulnerable to environmental changes such as soil warming, fire, and erosion. Managing land to increase soil carbon sequestration and persistence may therefore improve long‐term soil carbon storage and contribute to climate change mitigation. It has been hypothesized that grazing by large herbivores may enhance the persistence of soil carbon by increasing the amount of soil organic matter forming more stable associations with mineral particles (mineral‐associated organic matter). We compared sheep‐grazed and ungrazed plots within the Gibson Grazing and Successional Experiment located in the Upper Seeds calcareous grassland in Wytham Woods, Oxfordshire, using organic matter fractionation to estimate the surface (0–5 cm) carbon stocks in the mineral‐associated and particulate organic matter fractions. Counter to expectations, after 35 years sheep grazing had not increased mineral‐associated organic matter carbon stocks relative to ungrazed plots. We hypothesize that this indicates the saturation of mineral surfaces in both grazed and ungrazed treatments and the inability of short‐duration mob‐grazing to increase soil fertility. Grazing also did not influence overall soil carbon stocks which, based on various assumptions, could be consistent with the concept of net carbon storage whereby soil carbon stocks are maintained despite reduced aboveground plant biomass inputs. The higher C:N ratio in the mineral‐associated organic carbon in the spring‐grazed plots suggests this could have resulted from increased rhizodeposition in response to grazing (although we have no direct evidence to support this). Overall, while our measurements suggest possible compensatory carbon inputs to offset losses due to sheep grazing, they demonstrate no increase of stable soil carbon over the 35‐year duration of the experiment.

## Graphical abstract

This study investigates whether sheep grazing enhances soil carbon persistence by increasing mineral‐associated organic matter formation in a 35‐year experiment in a calcareous grassland in the UK. Contrary to predictions, grazing did not increase mineral‐associated carbon stocks, challenging the hypothesis that grazing improves long‐term soil carbon storage.

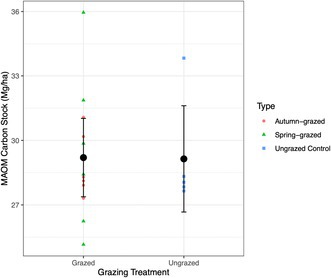

## 1 Introduction

The global soil carbon pool is more than three times the size of the atmospheric carbon pool (Lal 2004). Consequently, soil carbon sequestration (“transferring atmospheric CO_2_ into long‐lived pools and storing it securely so it is not immediately reemitted”) (Lal 2004) may be a useful tool to combat climate change. Land management regimes are often suggested as integral not only to soil carbon storage (Conant et al. 2017) but also the long‐term persistence of the soil carbon pool (e.g., Kristensen et al. 2022). As such, appropriate land management regimes are a potential tool for sequestration of carbon from the atmosphere into soils. Grasslands, which cover 20%–30% of the Earth's ice‐free land (Gibson and Newman 2019; Wilson et al. 2018) and 36% of the UK's land cover (Ward et al. 2016), provide multiple ecosystem services including carbon storage (Gibson and Newman 2019). Temperate grasslands store over 12% of global carbon and are the third largest store of carbon in soil and vegetation (Ward et al. 2016). However, nearly half of grassland area has already been degraded with associated declines in soil carbon stocks (Bardgett et al. 2021). Changes in grassland management therefore have the potential to affect soil carbon storage and persistence.

The amount of carbon stored in soils reflects the balance of inputs and outputs of carbon (Lal 2004), as influenced by environmental conditions (pH, oxygen availability, etc.). Carbon storage is the net outcome of a complex network of processes including the productivity of the system, the decomposability of the carbon inputs (intrinsic organic matter characteristics), and the protection against decomposition by physicochemical interactions with mineral particles. Intrinsic characteristics of organic matter, such as lignin concentration, have traditionally been seen as the main determinants of decomposability. However, recent perspectives emphasize “bioaccessibility”—the extent to which organic matter is accessible to microbial decomposition—as the primary factor (Just et al. 2021; Lehmann and Kleber 2015). While the intrinsic traits remain relevant, particularly for determining the initial decomposability, bioaccessibility is largely influenced by physical stabilization within soil aggregates and microaggregates and chemical stabilization through association with mineral particles (Six et al. 2002). This perspective divides soil organic matter into fractions with distinct formation pathways, carbon storage capacities, and persistence levels (Cotrufo et al. 2019; Lavallee et al. 2019; Von Lutzow et al. 2007), with the potential for grazing to impact these processes and fractions.

*Particulate organic matter (POM)* consists largely of undecomposed plant structural compounds such as root and shoot tissues. It is primarily stabilized through the intrinsic characteristics of organic matter, which reduce microbial decomposition (Cotrufo et al. 2019). However, particulate organic matter is vulnerable to increased decomposition under environmental changes like soil warming (Abramoff et al. 2021). The intrinsic characteristics only protect the particulate organic matter from decomposition for a few years to decades, though some types of particulate organic matter (e.g., when occluded in soil aggregates) can persist for hundreds of years (Angst et al. 2023; Lugato et al. 2021; Wasak and Drewnik 2015). Grazing has the potential to impact particulate organic matter, principally by consuming aboveground plant material, respiring much of the carbon and returning dung to the soil.

*Mineral‐associated organic matter (MAOM)* primarily consists of belowground carbon inputs like root exudates. Unlike particulate organic matter, mineral‐associated organic matter gains stability through physical protection in soil aggregates and microaggregates and chemical protection via sorption to mineral surfaces, making it more resistant to decomposition and less vulnerable to environmental perturbations (Kristensen et al. 2022; Lugato et al. 2021; Wasak and Drewnik 2015). This “physicochemical inhibition” allows mineral‐associated organic matter to persist for centuries, playing a key role in long‐term carbon storage (Kristensen et al. 2022). As such, carbon sequestration efforts should consider not only carbon pool sizes but also persistence when prioritizing land use and management strategies. Grazing has the potential to influence mineral‐associated organic matter, including by increasing levels of belowground carbon inputs to the soil through increased root growth and exudation.

While well‐documented, the impacts of grazing on soil carbon storage are highly variable, with studies reporting positive, negative, and neutral effects. A recent review (Abdalla et al. 2018) showed that grazing led to a reduction in soil carbon stocks globally, though this varied by climate, with grazing reducing soil carbon stocks the most in moist‐cool climate zones and increasing soil carbon stocks in other climates such as moist‐warm climate zones. Another global meta‐analysis showed that grazing effects on soil carbon depend on the interplay between climate, soil, and grassland type (McSherry and Ritchie 2013). For example, on fine‐textured soils of high clay content, grazing was found to negatively influence soil carbon at higher precipitation, whereas on coarse‐textured sandy soils, grazing had the opposite effect, increasing soil carbon at higher precipitation (McSherry and Ritchie 2013).

Moreover, Roy and Bagchi (2021) present the so‐called “paradox” of soil carbon in grazed ecosystems. They argue that one would expect grazing to reduce soil carbon as herbivores consume plant biomass, respiring carbon to the atmosphere and therefore reducing carbon inputs to the soil. However, using a long‐term grazing experiment, they showed that moderate grazing maintained soil carbon stocks despite reducing aboveground carbon inputs compared to grazing exclusion (net carbon storage). They propose that this results from decreased activity of enzymes involved in litter degradation, in line with the conceptual framework by Pausas and Bond (2020), which suggests that grazing competes with decomposition as a route to recycling carbon from biomass to the atmosphere. On top of this, Naidu et al. (2022) showed at the same site that moderate grazing increased both the size of the soil carbon pool and the stability of the soil carbon pool through time. Few sites have decadal time‐series of soil carbon measurements allowing such direct assessment of stability over time. Hence, indirect measures of the expected persistence of soil carbon may be valuable (Lavallee et al. 2019).

Kristensen et al. (2022) summarized mechanisms by which grazing may influence the distribution of carbon between pools of different persistence. In terms of grazer effects on carbon and nitrogen distributions between particulate organic matter and mineral‐associated organic matter pools within a given system (the focus here, as opposed to comparison across sites), the key mechanisms include grazing‐enhanced belowground input of root exudates and enhanced microbial carbon use efficiency. These mechanisms and the formation of particulate and mineral‐associated organic matter depend on many soil properties, primarily nitrogen. All soil organic matter contains nitrogen and is produced via microbial processes that require nitrogen. As a result, soil carbon stocks depend on nitrogen availability (Cotrufo et al. 2019). Particulate organic matter tends to have a low nitrogen content (a high carbon to nitrogen ratio), and mineral‐associated organic matter a relatively higher nitrogen content (a lower carbon to nitrogen ratio) (Cotrufo et al. 2019; Lugato et al. 2021). So, while mineral‐associated organic matter is more stable long‐term than particulate organic matter, its formation requires more nitrogen (Lugato et al. 2021). There is therefore the potential for grazing to affect soil carbon stocks via its impact on nitrogen.

This study used a long‐term sheep grazing experiment on a calcareous grassland to test the primary hypothesis that grazing by large herbivores enhances soil carbon persistence by increasing the amount of mineral‐associated organic carbon. We also tested the associated hypothesis that grazing by large herbivores increases soil fertility, increasing nitrogen stocks and decreasing C:N ratios over time.

## 2 Materials and Methods

### 2.1 Study Site

**FIGURE 1:**
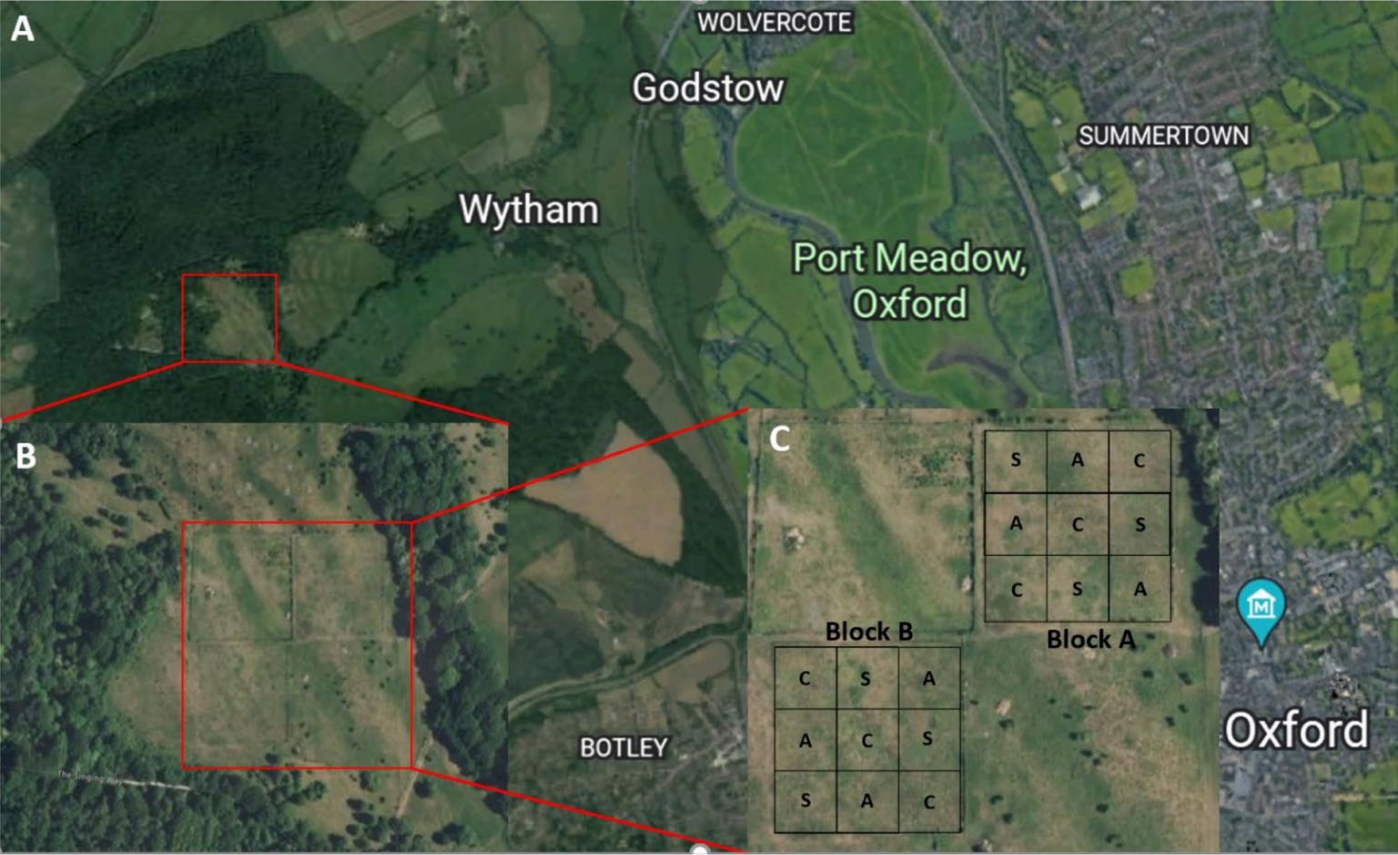
Study site. (A) Map of Wytham Woods relative to central Oxford (5 km northwest of Oxford); (B) Upper Seeds grassland within Wytham Woods. (C) Gibson Grazing and Successional Experiment within Upper Seeds. The experiment has a replicated Latin grid square design with two blocks (A and B) and three treatments within each block (A = autumn‐grazed; C = ungrazed control; S = spring‐grazed). Images retrieved from Google Satellite.

Soil samples were collected from the long‐term Gibson Grazing and Successional Experiment (“Gibson experiment,” formerly known as the “Upper Seeds Experiment”) within the Upper Seeds grassland in the Wytham Woods estate (51°46′10.6″ N, 1°19′54.9″ W) (Gibson 2011). The site lies on top of a small hill (160 m above mean sea level) with a south‐easterly aspect and mean annual rainfall (2008–2018) of 746 mm. Upper Seeds is a calcareous grassland approximately 10 ha in size (Figure 1) with a shallow (10–20 cm) soil crust on top of a calcareous bedrock made up of Jurassic coral reef (Stone 2020). The site was likely unfertilized grazed grassland for many centuries before World War II, then converted to fertilized cereal cropping until 1980, when it was set aside for conservation (Gibson 1986; Gibson et al. 1987). The resulting Upper Seeds soil is a shallow, well‐drained calcareous clay soil recovering from the previous disturbance (including ploughing for arable production) (Taylor et al. 2011). The Gibson Grazing and Successional Experiment ran for 35 years (1985–2020), though after 2014 there were a few occasions in which sheep were removed a few days early for logistical reasons so grazing duration was slightly lower. In 2021 fences between plots were removed so that sheep were free to graze the whole experiment for one growing season after the formal end of the experiment (see Section 4). Soil samples were collected in January 2022 to assess the cumulative effects of 35 years of differences in grazing treatments.

### 2.2 Experimental Design

The Gibson Experiment comprises three treatments imposed onto 18 30 × 30 m plots: two sheep‐grazed treatments (spring‐grazed and autumn‐grazed) and one ungrazed control. The grazing regime consisted of three sheep in each of the grazed plots, which equates to a stocking rate roughly double the recommendation by the National Sheep Association (n.d.) for productive grasslands. The spring‐ and autumn‐grazed paddocks were grazed for approximately 2 weeks in April and September, respectively (Gibson et al. 1987). Given the high stocking rate and short duration, the grazing regime could be viewed as high‐intensity, short‐duration mob‐grazing. Because of the focus of the Gibson Experiment on successional dynamics, the ungrazed plot was allowed to undergo secondary succession to a point at which young shrubs (mainly hawthorn [*Crategus monogyna* Jacq.] and blackthorn [ *Prunus spinosa* L.]) approached a size at which they would be problematic to remove, at which point secondary succession was reset by removal of shrubs and mowing. Hawthorn and other shrubs were removed to reset secondary succession in 2004, 2013, and 2017. The experiment has an unusual replicated blocked Latin square design with two blocks of nine plots and three replications of each treatment within each block (Figure 1C; Gibson et al. 1992). This design was intended to encompass known gradients in soil quality across the field, with Latin squares orientated to maximize variation encompassed within control sites and thus increase confidence in any contrast detected with a grazing treatment. Thus, the total sample size is 18: 3 treatments (2 Grazed, 1 Ungrazed) × 3 replications of each treatment within each of two blocks (3 × 3 × 2 = 18 plots).

### 2.3 Soil Sampling

**FIGURE A1:**
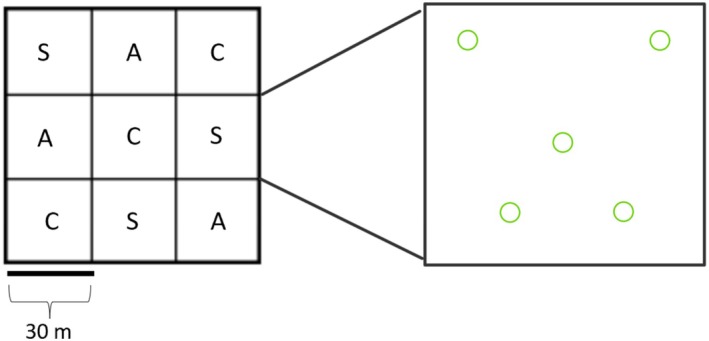
One block of plots showing Latin square layout (Left: A = autumn‐grazed; C = ungrazed control; S = spring‐grazed) and the W‐formation of sampling points within each plot (Green circles refer to each of the five subsamples taken within each plot. The five subsamples within each plot together form one composite sample).

To compare grazed and ungrazed plots, we collected 18 composite samples (one from each of the 18 Gibson plots) from the top 5 cm of soil. Each composite sample consisted of five subsamples (each 100 cm^3^) collected in a W‐formation (Figure A1) using a soil core approximately 5 cm in diameter. Composite samples were thoroughly mixed and dried at 40°C to constant weight, and any rocks or other debris were removed from the samples.

### 2.4 Determination of Soil Physical and Chemical Properties

Soil pH was measured using a 1:1 soil–water ratio using the mixed and dried composite samples. Bulk density (BD) was calculated for each composite sample (500 cm^3^) as:

(1) $$\mathrm{BD}\;\left(g/{\mathrm{cm}}^{3}\right)=\frac{{\text{Mass}}_{\mathrm{Dry}\;\text{soil}}\;\left(g\right)}{\text{Soil volume}\;\left({\mathrm{cm}}^{3}\right)}$$

where the dry soil mass refers to the mass of the sample after it has been dried at 40°C.

We used the loss on ignition (LOI) method to determine inorganic carbon contents. We heated each 2‐g sample to 105°C to determine the dry soil mass, heated it to 550°C for 4 h to remove the organic carbon in the samples and then to 950°C for 2 h to obtain the mass of CaCO_3_. We calculated the inorganic carbon stocks in the top 5 cm of soil as follows (Equation 2), where 0.12 corresponds to the carbon mass fraction in CaCO_3_ (Soil Survey Staff 2011):

(2) $$C_{\text{Inorganic}}\left(g\;C/{\mathrm{cm}}^{2}\right)=\mathrm{BD}\left(g\;C/{\mathrm{cm}}^{3}\right)\times \frac{{\text{Mass}}_{{\text{CaCO}}_{3}}\left(g\right)}{{\text{Mass}}_{\mathrm{Dry}\;\text{soil}}\left(g\right)}\times 0.12\times 5\;\mathrm{cm}$$

To estimate the total carbon and nitrogen content (%) of the samples, we performed CN elemental analysis using standard laboratory protocols (Soil Survey Staff 2011). To divide the organic matter into mineral‐associated organic matter and particulate organic matter, we performed size‐fractionation of organic matter. This method separates the soil into two size‐fractions: > 53 μm, which is dominated by particulate organic matter, and < 53 μm, which is dominated by mineral‐associated organic matter. To thoroughly mix each sample, we combined 2.5 g samples of dry soil with 35 mL of distilled water and sonicated the mixture until it reached 15,750 joules (450 joules/mL water). To separate the fractions, we strained this soil–water mixture through a 53‐μm sieve, dried the two fractions in an oven at 60°C, and then ground the samples. To estimate the carbon and nitrogen content of each of the fractions in each of the samples, we performed CN elemental analysis as described above after size‐fractionation. We calculated the carbon and nitrogen stocks in each fraction as follows (Equation 3), where carbon can be interchanged with nitrogen and mineral‐associated organic matter with particulate organic matter.

(3) $$\text{MAOC stock}\;\left(g\;C/{\mathrm{cm}}^{2}\right)=\mathrm{BD}\left(g\;C/{\mathrm{cm}}^{3}\right)\times \frac{{\text{Mass}}_{\text{MAOM}}\left(g\right)}{{\text{Mass}}_{\mathrm{Dry}\;\text{soil}}\left(g\right)}\times C_{\text{MAOM}}\times 5\;\mathrm{cm}$$

We calculated the C:N ratio in each fraction as follows (Equation 4), where mineral‐associated organic matter can be interchanged with particulate organic matter.

(4) $$C:N_{\text{MAOM}}=\frac{C_{\text{MAOM}}}{N_{\text{MAOM}}}$$

### 2.5 Statistical Analysis

Our analysis used linear mixed‐effects models implemented with version 3.3.3 of the nlme R package in R (R Core Team 2023; Pinheiro et al. 2024) following a model building approach (Pinheiro and Bates 2000). For this research question, the Latin square design is overly complex relative to the limited sample size leading to singularities when trying to estimate the coefficients in complex models, necessitating simplification to achieve a model that would converge and produce credible estimates of the coefficients of interest. The simplified mixed‐effects model included a fixed effect for the treatment of interest (grazing) and a random effect for blocks (estimates of treatment effects were very similar with alternative simpler models—see Appendix 1). A generic formula for the mixed‐effects model using the R language implementation of the Wilkinson and Rogers (1973) syntax is:

(5) $$\text{34𝑌}\sim \text{34𝑌𝑋}+\left(1|\text{Block}\right)$$

where, *Y* is a continuous response variable (one of the 12 listed in Table A1) and *X* is a fixed factor with either three levels (spring‐grazed, autumn‐grazed, and ungrazed) or a simplified two‐level factor comparing grazed and ungrazed plots. “Block” is a random factor with two levels for blocks (Gelman and Hill 2007).

## 3 Results

Of the 24 formal treatment comparisons, only one was conventionally statistically significant (see below). The results should be interpreted considering the likelihood of one significant result for every 20 tests at the *p* < 0.05 level. While the original Gibson experiment was designed with one ungrazed and two grazed treatments (spring‐ and autumn‐grazed), our core question of grazing impact on soil carbon persistence addresses the difference between grazed and ungrazed plots, with no specific hypothesis about the timing of grazing. Therefore, we focus presentation of the results on our question (contrast between grazed and ungrazed plots), but also consider the three‐treatment comparison following the original full experimental design.

### 3.1 Effects of Grazing on Soil Properties

**FIGURE 2:**
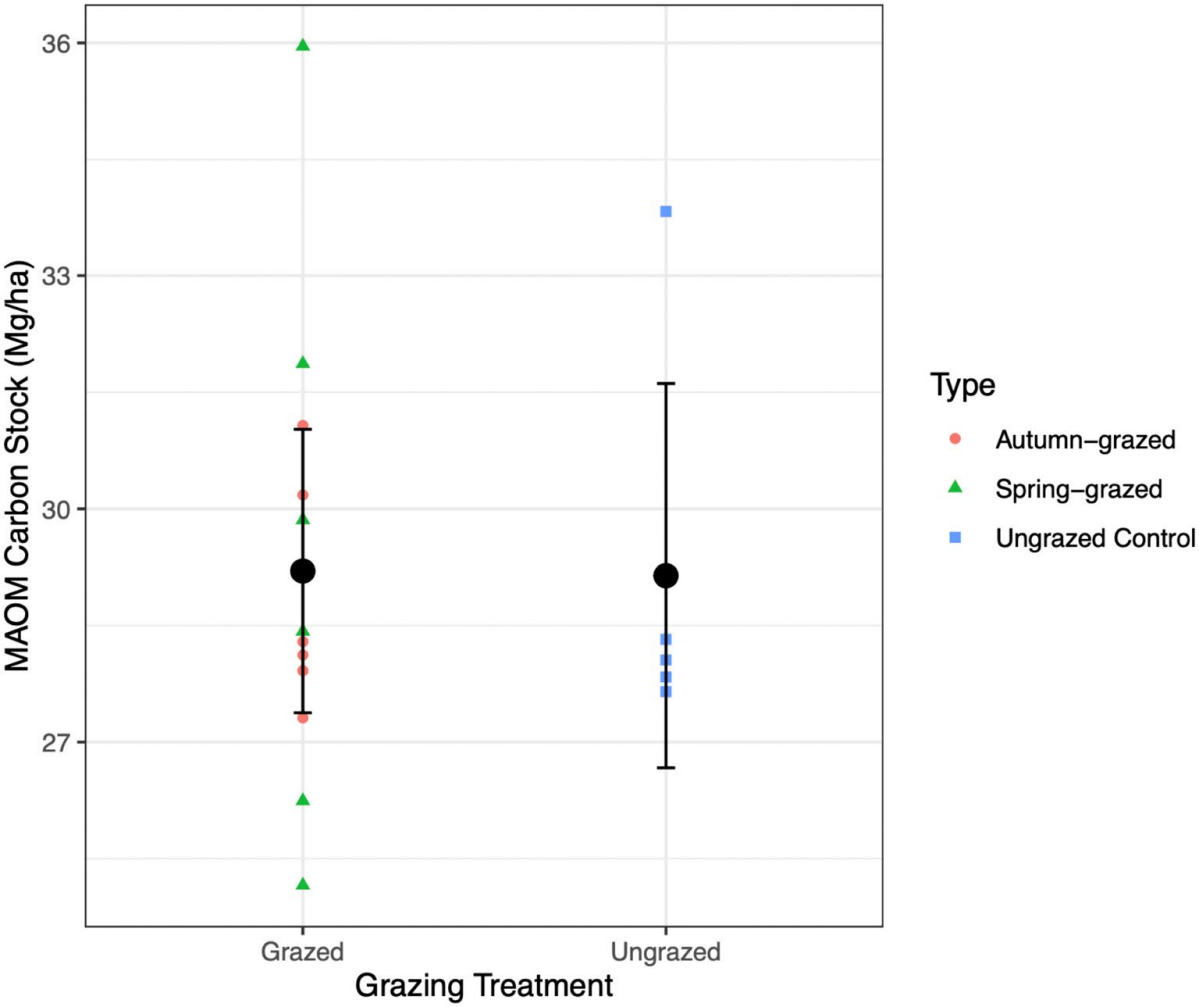
Carbon stocks in the MAOM fraction in grazed and ungrazed treatments: Small colored points refer to individual plots. Larger black dots represent the treatment means and bars indicate the 95% confidence intervals from the linear mixed‐effects model analysis likelihood profiles (*n*_total_ = 18; *n*_ungrazed_ = 6, *n*_grazed_ = 12).

To address our main hypothesis on the effects of grazing we first performed comparisons of the soil properties in the grazed and ungrazed plots (i.e., pooling the spring‐ and autumn‐grazed plots). In general, estimates of soil properties in the grazed and ungrazed treatments were similar with large overlap of confidence intervals (Table 1, Figure 4). The mixed‐effects model analysis revealed that sheep grazing did not affect the mineral‐associated organic carbon stock in the top 5 cm of soil (Figure 2). In addition, sheep grazing had no significant effect on the particulate organic matter carbon stocks, total organic carbon stocks, inorganic carbon stocks, or total carbon stocks, which all had relatively similar mean values and overlapping confidence intervals (Table 1, Figure 4, Table A2).

**TABLE 1:** Effects of grazing on selected soil properties in the top 5 cm. Treatment means with 95% CI bounds for ungrazed and grazed plots (combined and separately for spring‐ and autumn‐grazed).

Response	Ungrazed (*n* = 6)	Grazed (*n* = 12)	Spring‐grazed (*n* = 6)	Autumn‐grazed (*n* = 6)
MAOM C stocks (Mg C/ha)	29.14 (26.67, 31.61)	29.2 (27.38, 31.02)	29.58 (25.44, 33.73)	28.8 (27.27, 30.36)
POM C stocks (Mg C/ha)	7.66 (6.19, 9.13)	7.32 (6.22, 8.42)	7.9 (5.59, 10.21)	6.7 (5.73, 7.74)
SOC (Mg C/ha)	36.8 (33.69, 39.91)	36.52 (33.82, 39.22)	37.49 (31.27, 43.70)	35.55 (33.92, 37.19)
Inorganic C (Mg C/ha)	4.77 (3.27, 6.27)	5.16 (4.12, 6.20)	5.65 (3.39, 7.90)	4.68 (3.76, 5.59)
MAOM N stocks (kg N/m^2^)	0.25 (0.23, 0.28)	0.24 (0.23, 0.25)	0.24 (0.21, 0.26)	0.24 (0.22, 0.27)
POM N stocks (kg N/m^2^)	0.016 (0.015, 0.018)	0.015 (0.013, 0.017)	0.015 (0.013, 0.017)	0.014 (0.011, 0.018)
Total organic N (kg N/m^2^)	0.269 (0.245, 0.293)	0.257 (0.243, 0.270)	0.254 (0.229, 0.279)	0.259 (0.239, 0.279)
MAOM C:N ratio	11.53 (11.06, 12.01)	12.09 (11.67, 12.50)	12.37 (11.57, 13.17)	11.80 (11.37, 12.24)
POM C:N ratio	47.81 (38.32, 57.30)	49.78 (42.80, 56.76)	51.62 (38.32, 64.91)	47.94 (37.61, 58.28)
SOC C:N ratio	14.40 (13.21, 15.59)	15.52 (14.04, 16.70)	16.35 (13.14, 19.57)	14.68 (13.69, 15.68)
pH	7.57 (7.46, 7.67)	7.65 (7.58, 7.71)	7.70 (7.62, 7.79)	7.60 (7.49, 7.70)
Bulk density (g/cm^3^)	0.78 (0.71, 0.86)	0.78 (0.73, 0.83)	0.81 (0.72, 0.89)	0.75 (0.67, 0.84)

Across all treatments, the carbon stored in the mineral‐associated organic matter fraction made up most of the total soil carbon pool (range: 53.4%–89.1%). The particulate organic matter carbon stocks made up a smaller pool (range: 12.3%–26.4%). The smallest but still sizeable pool was inorganic carbon (range: 7.9%–19.8%) (Table 1, Figure 4). The C:N ratio of the soil organic carbon pools is an indicator of the quality of carbon inputs to the soil (Naidu et al. 2022). Across all treatments, the C:N ratio was higher in the particulate fraction than in the mineral‐associated organic matter fraction, as expected (Table 1).

Sheep grazing did not influence the C:N ratio in the particulate organic matter fraction or the total C:N ratio, but it did lead to a greater C:N ratio in the mineral‐associated organic matter fraction compared to the ungrazed treatment (Table 1, Figure 4). Due to the way mineral‐associated organic matter is formed in grasslands, the C:N ratio of mineral‐associated organic matter is more reflective of the average C:N of the microbes, whereas the C:N of the particulate organic matter is a more direct reflection of the C:N in the undecomposed plant material (litter) (Sokol et al. 2022). The sheep‐grazed and ungrazed treatments had similar total nitrogen stocks (Table 1, Figure 4, Table A2). Sheep grazing did not affect soil pH, with grazed and ungrazed plots having relatively similar mean values and overlapping confidence intervals (Table 1, Figure 4). Finally, the bulk densities in sheep‐grazed and ungrazed treatments were nearly identical, suggesting that sheep grazing did not affect bulk density (Table 1, Figure 4, Table A2).

### 3.2 Effects of Grazing Timing on Soil Properties

**FIGURE 3:**
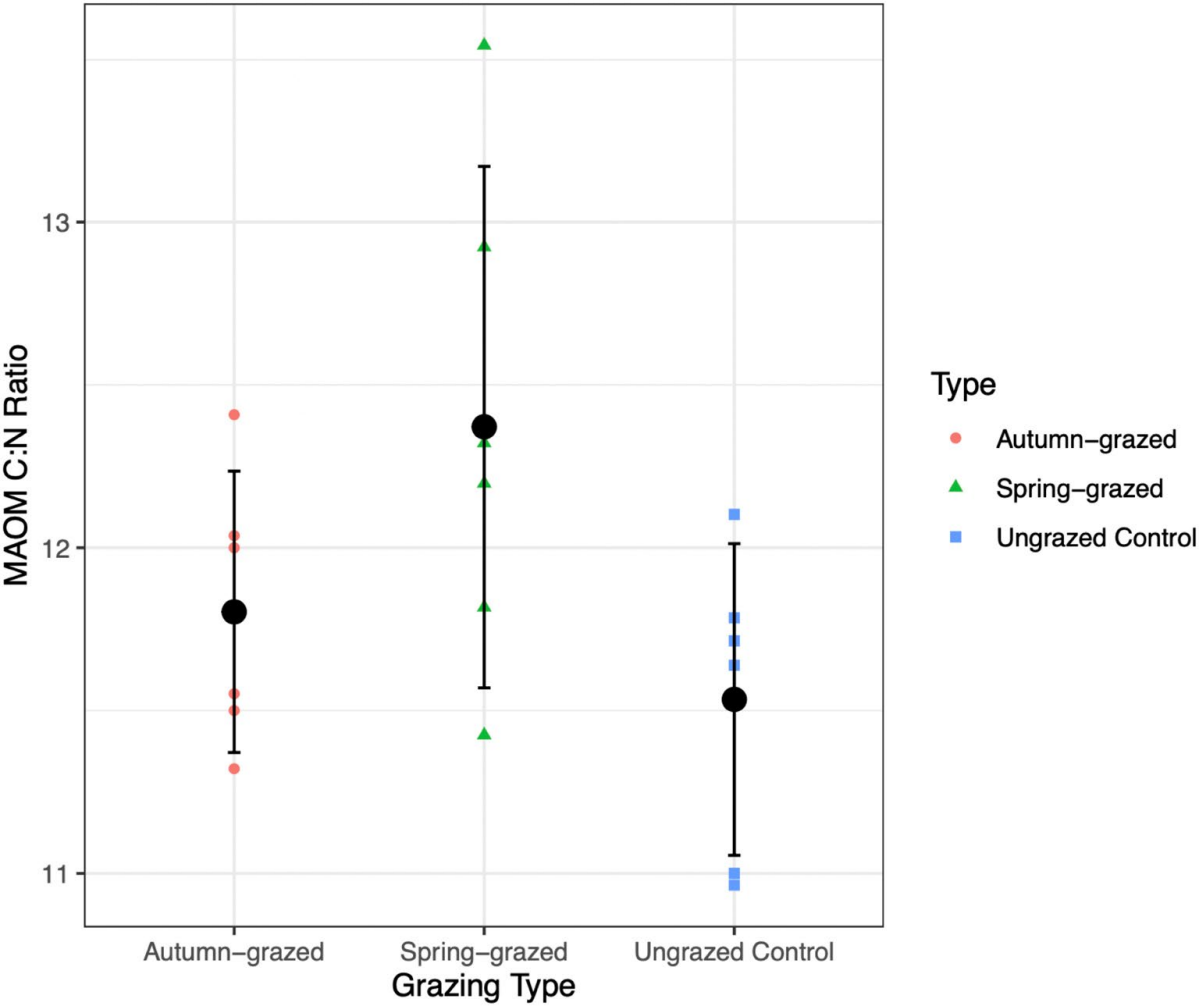
Comparison of C:N ratio in the MAOM of ungrazed, autumn‐grazed, and spring‐grazed plots: Small colored points refer to the MAOC stocks found in the individual plots. Larger black dots represent the grazing type (timing) means and bars indicate the 95% confidence intervals from the linear mixed‐effects model analysis likelihood profiles (*n*_total_ = 18; *n*_treatment_ = 6).

After performing an initial contrast of grazed versus ungrazed plots, we proceeded to separate the spring‐ and autumn‐grazed plots to compare all three treatments. Once again, soil properties were generally similar across treatments, with overlap of confidence intervals, with two exceptions. First, grazing timing had a marginal positive effect on pH (Table A3). Spring grazing led to a higher (more alkaline) pH than the ungrazed control, but autumn grazing did not alter the pH relative to the ungrazed control. However, the absolute difference is small (although the logarithmic scale of the pH unit must be kept in mind): the mean pH in the spring‐grazed plots was 7.70 compared to a mean of 7.57 in the ungrazed plots (Table 1). Second, grazing timing had a significant positive effect on the mineral‐associated organic matter C:N ratio (Table 1, Table A3). Spring grazing produced the highest C:N ratio in the mineral‐associated organic matter fraction (Table 1), significantly greater than the ungrazed control. In contrast, autumn grazing did not affect the C:N ratio in the mineral‐associated organic matter fraction relative to the ungrazed control (Figure 3, Table A3).

## 4 Discussion

**FIGURE 4:**
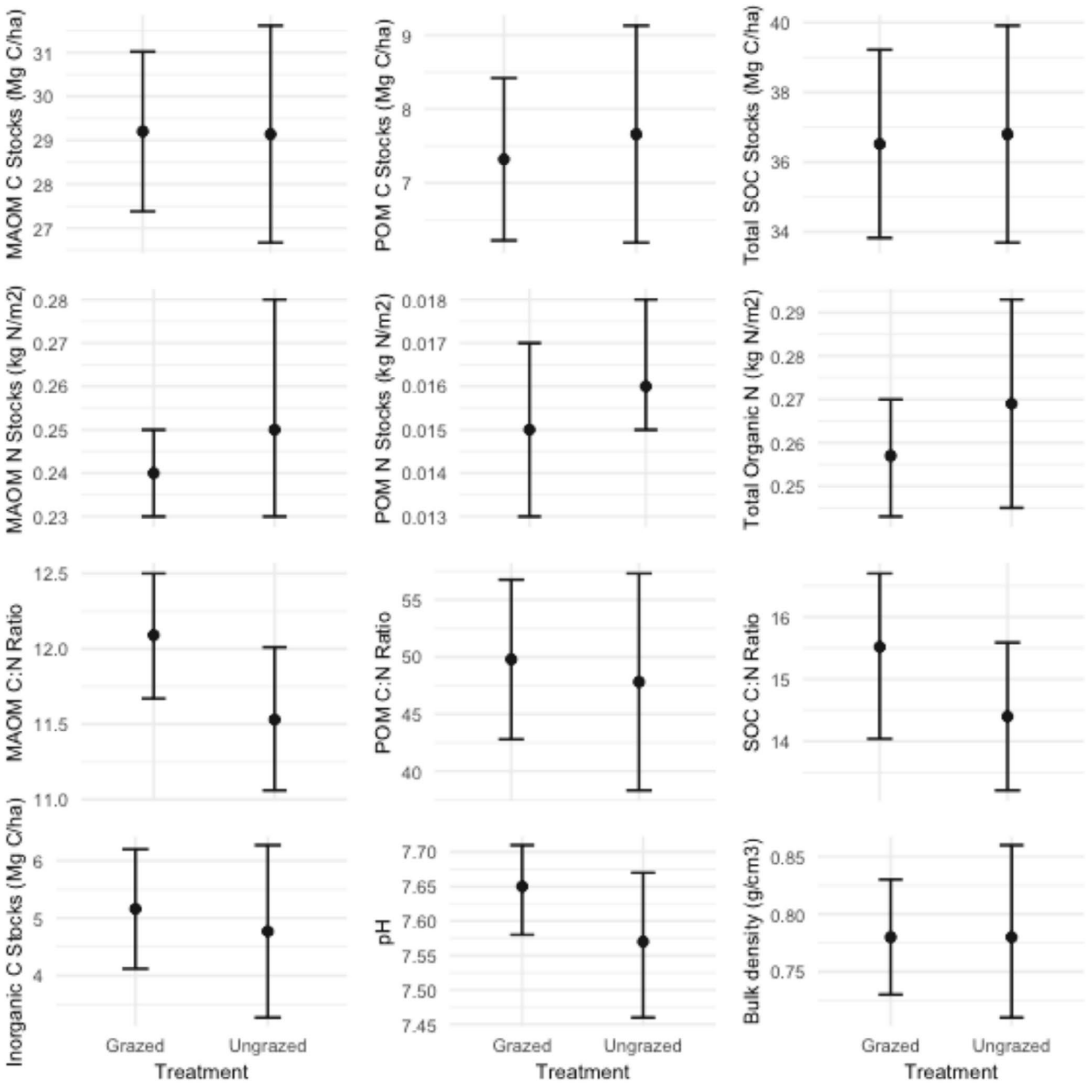
Effects of grazing on selected soil properties. Black dots represent the treatment means and bars indicate 95% confidence intervals from the linear mixed‐effects model analysis likelihood profiles.

We used a long‐term (35 years) sheep grazing experiment to test the hypothesis that grazing increases soil carbon persistence by increasing the carbon stored in the more stable mineral‐associated organic matter fraction. Our results showed that sheep grazing did not enhance the mineral‐associated stocks (Figure 2), nor total carbon stocks (Table 1, Figure 4). Similar carbon stocks in grazed and ungrazed plots, despite the loss of carbon through grazer consumption and respiration, imply net carbon storage through a compensating increase in belowground carbon inputs to the soil system (which we discuss further below). The only conventionally statistically significant result (*p* < 0.05) from the 24 formal tests performed was of a higher mineral‐associated organic matter C:N ratio in the grazed plots, though this was driven by increases in spring‐grazed plots specifically (Figure 3, Table A3). We discuss the implications of this result below, but given the low sample size (*n* = 18, 6 replicates of 3 treatments) and number of tests performed, we caution this result could be a false positive and needs confirming by independent study.

### 4.1 Sheep Grazing Did Not Increase Stable Soil Carbon in Mineral‐Associated Organic Matter

In this study, sheep grazing did not increase mineral‐associated carbon sequestration (Figure 2). This suggests that sheep grazing does not lead to a more persistent soil carbon pool in this system, contrary to the hypothesis proposed by Kristensen et al. (2022). The estimates of mineral‐associated organic matter in the grazed and ungrazed plots are nearly identical, as were the estimates of other soil properties (Table 1). While many relevant stocks, flows, and properties were not quantified in our study, our measurements can be assessed for consistency with different possible explanations for the lack of grazing effect on mineral‐associated organic matter.

The most likely explanation for our main result is that the soil at our study site lacked much potential for increased storage of mineral‐associated organic matter. In this study, mineral‐associated organic matter made up a high proportion of soil organic carbon and total soil carbon (53.4%–89.1% across all treatments) (Table 1). This suggests that the soils at Upper Seeds may have approached their finite mineral‐associated carbon storage capacity. Mineral‐associated carbon sequestration is saturating, whereas particulate carbon sequestration is not. The amount of carbon that can be stored in the mineral‐associated organic matter is finite and largely determined by the availability of adsorption sites at mineral surfaces—effectively a result of the clay content and type (Georgiou et al. 2022). This means that carbon inputs to the soil can only be incorporated into mineral‐associated organic matter when there is a “saturation deficit” (Castellano et al. 2015). Once the mineral surfaces become saturated with carbon, mineral‐associated carbon stocks can no longer increase, meaning that the organo‐mineral exchange is at dynamic equilibrium (Cotrufo et al. 2019). Thus, it is possible that the lack of effect of sheep grazing on mineral‐associated carbon stocks shown here reveals that this soil has become carbon‐saturated and that there are few available mineral surfaces to shield the organic matter from decomposition. Given that mineral‐associated carbon sequestration is constrained by saturation limits, any potential impact of grazing on these stocks would likely depend on factors that influence soil fertility and carbon inputs.

Kristensen et al. (2022) suggested that grazing by large herbivores should enhance mineral‐associated carbon sequestration in part by increasing soil fertility (particularly nitrogen), which in turn increases plant productivity and the quantity of carbon inputs to the soil, particularly belowground. The persistence of soil carbon can also be nitrogen dependent, with mineral‐associated organic matter generally having a lower C:N ratio than particulate organic matter (Lugato et al. 2021). This general trend is reflected in this calcareous grassland as well, with the particulate organic matter C:N ratio higher than the mineral‐associated organic matter C:N ratio across all treatments (Table 1).

While a recent global review showed that grazing generally led to significant increases in total soil nitrogen (Abdalla et al. 2018), the grazing regime in our study did not increase soil nitrogen stocks (Table 1, Figure 4), with no reason to expect associated increases in productivity and the quantity of carbon inputs to the soil. This could be due to the “harvest export” (nutrient stripping) from the system, where the sheep are moved to different fields (and ultimately from the system when they are used for products such as wool and meat) although they may also import nutrients when moved into an area. All plots—both grazed and ungrazed—also experienced potential export of nutrients from the system during the three rounds of scrub removal needed to maintain the system as a grassland (see Section 2). However, scrub build up was more intense in the ungrazed than grazed plots, and it is unlikely that the unequal removal of scrub across the treatments would act in a way to equalize soil characteristics.

### 4.2 Grazing May Have Led to Compensatory Carbon Inputs That Offset Losses due to Grazing

Although we did not quantify aboveground biomass inputs to the soil, by consuming aboveground vegetation, grazers are expected to reduce aboveground plant biomass and associated carbon inputs to the soil and thereby reduce soil carbon stocks. However, we observed no such change in soil carbon stocks. So, while sheep grazing did not increase soil carbon stocks or persistence (as discussed above), the lack of reduction in soil carbon stocks can be viewed as increased net storage of carbon, that is, proportionally greater carbon storage per unit of carbon input. This is consistent with previous studies (e.g., Naidu et al. 2022; Roy and Bagchi 2021) that showed that moderate grazing can paradoxically increase net soil carbon storage compared to ungrazed plots. We only have measures of total soil carbon stocks in the grazed and ungrazed plots, not the processes that influence the size of these stocks. The inference of compensatory net carbon storage is therefore based on assuming everything remains largely the same except the loss of carbon from the system due to grazing. One key assumption is that soil microbial biomass and activity does not increase in ungrazed plots to decompose the biomass otherwise consumed by grazers in the grazed plots, which would offset the grazing‐induced reduction in biomass inputs in the grazed plots. A recent meta‐analysis (Zhao et al. 2017) showed that light and moderate grazing had no effect on soil microbial, bacterial, and fungal community size and associated soil respiration. Further, Bardgett et al. (1997) showed that removal of sheep grazing on two grassland types in the UK reduced both microbial biomass and microbial activity. This suggests that any increase in respiration by sheep grazing of aboveground biomass is additional (i.e., does not lead to a commensurate and offsetting increase in microbial respiration). Assuming other processes are similar in grazed and ungrazed plots, we can make a rough estimate of the carbon removed from the system through grazer respiration.

If we can assume that one 70‐kg mule sheep (Agriculture and Horticulture Development Board, n.d.) consumes approximately 4% of its body weight per day, this would be 2.8 kg (2800 g) of grass dry matter per day (Vipond 2017). Assuming the grass has a carbon content of 45% (Adamovics et al. 2018) and given there were three sheep in each 30 × 30 m (900 m^2^) paddock grazing for 14 days, the amount of carbon inputs consumed are as follows:

(6) $$\frac{2800\;g\;\text{Grass}\times 0.45\times 14\;\text{days}\times 3\;\text{sheep}}{900\;m^{2}}=58.8\;g\;C/m^{2}\;\text{consumed}$$

Some of this carbon is obviously returned to the soil as dung. If we assume that each sheep produces roughly 1800 g of dung per day (Utah State University Extension, n.d.), with a 40% carbon content (Li et al. 2022), the amount of grass‐derived carbon inputs returned to the soil as dung is as follows:

(7) $$\frac{1800\;g\;\text{Dung}\times 0.4\times 14\;\text{days}\times 3\;\text{sheep}}{900\;m^{2}}=33.6\;g\;C/m^{2}\;\text{returned}$$

We can conclude that roughly 25 g C/m^2^ is removed from the system. In the nearby RainDrop experiment (located only a few hundred meters from the Gibson experiment), a recent study revealed the average aboveground net primary productivity to be roughly 300 g/m^2^ (Jackson et al. 2024), which would equate to ~135 g C/m^2^. Thus, the consumption of biomass by sheep in our study plots represents a substantial reduction in carbon input. Yet, despite this reduction in carbon inputs to the soil for 35 years, the grazed plots did not experience a loss in soil carbon stocks. There are several possible reasons for this.

### 4.3 Possible Causes of the Potential Net Carbon Storage

One likely explanation for the possible net carbon storage is that sheep grazing promotes compensatory growth and alters the quantity and quality of carbon inputs to the soil. While grazing removes aboveground biomass, it may lead to increased carbon inputs belowground, for example, from increased root biomass or root exudates. These belowground inputs may be of a different quality than aboveground inputs and more shielded from microbial decomposition, which allows the grazed plots to maintain the same carbon stocks as ungrazed plots despite a reduction in total carbon inputs.

While grazing is expected to reduce plant biomass and aboveground carbon inputs (Roy and Bagchi 2021), if it can promote compensatory growth and induce pulses of root exudation, then it could increase the production of belowground carbon inputs that can easily be transferred to the soil solution and mineral‐associated organic matter pool (Hamilton et al. 2008; Villarino et al. 2021). We observed that the mineral‐associated organic matter C:N ratio was significantly higher in the sheep‐grazed treatment than in the ungrazed control. This was not consistent across the two grazing times, with only spring grazing leading to a higher mineral‐associated C:N ratio than the ungrazed control (Figure 3, Table A3).

The C:N ratio can be considered a fingerprint of what types of carbon inputs end up forming mineral‐associated organic matter. The C:N ratio decreases during decomposition, so the higher C:N ratio in the spring‐grazed plots suggests that a higher proportion of the organic matter in the mineral‐associated fraction in the spring‐grazed treatment is formed directly from dissolved organic carbon from plants (e.g., root exudates) rather than microbial necromass or shoot tissue. Dissolved organic carbon from rhizodeposits has a higher average C:N ratio than other carbon inputs such as shoot tissues (Ostrowska and Porebska 2015). Thus, the higher C:N ratio in the spring‐grazed treatment could suggest that spring grazing leads to greater rhizodeposition, probably due to compensatory growth (Villarino et al. 2021; Hamilton et al. 2008). Compensatory growth is a strategy evolved to cope with disturbances causing a loss of biomass (Järemo et al. 1996). When plants are consumed by grazers, they respond by growing more biomass, both above‐ and belowground. Belowground compensatory growth often involves increasing root growth and root exudation in exchange for nutrients from soil microorganisms but is only relevant during periods of active plant growth (Hamilton et al. 2008). Hence, in the early growing season, when both growth rates and forage quality are highest, this mechanism is most pronounced. In the autumn, when root networks are more established, the compensatory response is likely to be concentrated aboveground. This could explain why the mineral‐associated organic matter C:N ratio is higher in spring‐grazed but not in autumn‐grazed plots.

In addition to altering the quantity of carbon inputs to the soil, grazing can also impact the quality of the carbon inputs. Again, C:N ratio is one such indicator of input quality (Naidu et al. 2022). The higher C:N ratio observed in the mineral‐associated organic matter in the grazed plots (specifically the spring‐grazed) (Table 1, Figure 3) aligns with the concept that the carbon inputs that remain after grazing consist of the least decomposable inputs and thus the incoming litter is potentially less available for microbial decomposition (Olff and Ritchie 1998).

Overall, this suggests that compensatory growth, particularly increased root exudation in response to grazing, and the resulting change in carbon input quality (higher C:N ratio) could explain the maintenance of soil carbon stocks despite the grazing‐induced reduction in aboveground carbon inputs. However, it is important to emphasize that this explanation is to some degree speculative, based on the measurements taken, with many potential processes unmeasured in our study.

### 4.4 Other Influences on Soil Carbon Sequestration and Persistence

Soil pH influences carbon sequestration, but its impact is most pronounced when comparing sites that differ appreciably (e.g., acid vs. alkaline soils) rather than within a single site with a narrow pH range. Carbon storage in both particulate and mineral‐associated organic matter increases as pH decreases, primarily due to reduced microbial decomposition (Lugato et al. 2021) and enhanced sorption to mineral surfaces, which peaks at pH 4.3–4.7 (Lutzow et al. 2006). Organic matter sequestration in expandable clay interlayers occurs only at pH < 5 (Lutzow et al. 2006). While grazing can alter pH (Hiernaux et al. 1999), the minor shifts observed in our study (where average pH ranged from 7.57 to 7.70) were insufficient to affect soil carbon storage. In calcareous grasslands with shallow soils over limestone, high buffering capacity from base cations minimizes acidification, maintaining pH above neutrality and limiting pH‐induced effects on mineral‐associated organic matter sorption (Abramoff et al. 2021).

As described earlier (see Section 2), the stocking density was quite high and grazing duration was relatively low with three sheep in each grazed paddock for 2 weeks in either the spring or autumn. Our results show that not only did grazing have minimal effect on the investigated soil properties (Figure 4, Table 1), but the timing of grazing similarly also had minimal effect (Table A3). This suggests that the mob‐grazing regime did not provide sufficient disturbance to perturb the soil system away from the ungrazed state. This is supported by the neutral effect of grazing on bulk density. Trampling by grazers is often expected to increase bulk density by compacting the soil (Abdalla et al. 2018). However, in this case, bulk density was uniformly low (Table 1, Figure 4), perhaps because the grazing treatments in this study involved short‐duration mob‐grazing that did not compact the soil beyond its natural resilience, as sheep tend to compact soil less than larger grazers like cattle (Cournane et al. 2011; Gibson 2011).

Lastly, the cessation of the continuous management of the Gibson Grazing and Successional Experiment in 2020 and subsequent removal of the fences between the plots meant that sheep were free to graze the whole experiment for one season before the soil sampling occurred. However, it seems unlikely that grazing for 2 weeks in one season would have eliminated any treatment effects that had built up over the 35 years of the Grazing Experiment.

## 5 Conclusion

Our study shows that 35 years of sheep grazing in the Upper Seeds grassland did not influence topsoil carbon persistence in our study system (as measured by the size of the mineral‐associated carbon pool). Our measurements suggest this is most likely due to the saturation of mineral surfaces and the harvest export of nutrients from the system. Our study also showed no effect of grazing on total soil carbon stocks, which were similar in grazed and ungrazed plots. Given greater carbon losses in the grazed plots, this could be consistent with net carbon storage, where carbon stocks are maintained despite the reduction in aboveground biomass inputs. This could be the result of compensatory growth and increased rhizodeposition in response to grazing, as evidenced by a higher C:N ratio in the mineral‐associated carbon pool in the grazed plots, particularly the spring‐grazed treatment. In total, our results are consistent with a compensatory increase in less‐decomposable belowground carbon inputs in grazed plots but no increase in stable soil carbon pools, suggesting the role of livestock grazing in promoting soil carbon persistence be re‐evaluated.

## Author Contributions

**David Encarnation:** conceptualization (lead), data curation (lead), formal analysis (lead), investigation (lead), methodology (equal), project administration (lead), writing – original draft (lead), writing – review and editing (equal). **Deborah Ashworth:** methodology (supporting), resources (supporting), writing – review and editing (supporting). **Richard Bardgett:** project administration (supporting), resources (supporting), writing – review and editing (supporting). **Mona Edwards:** methodology (supporting), resources (supporting), supervision (supporting), writing – review and editing (supporting). **Clive Hambler:** project administration (supporting), resources (supporting), writing – review and editing (supporting). **Jeppe Kristensen:** conceptualization (equal), investigation (supporting), methodology (supporting), project administration (supporting), supervision (equal), writing – original draft (supporting), writing – review and editing (supporting). **Andrew Hector:** conceptualization (supporting), formal analysis (equal), funding acquisition (lead), investigation (supporting), methodology (supporting), project administration (equal), resources (equal), supervision (equal), visualization (supporting), writing – original draft (equal), writing – review and editing (equal).

## Conflicts of Interest

The authors declare no conflicts of interest.

## Supporting information


Appendix S1

Appendix S2

## Data Availability

The data (including primary data and R Markdown script) that support the findings of this study are available in the Supporting Information.

## Appendix 1

### Statistical Analysis

The blocked Latin square design of the Gibson grazing experiment is too complex given the small sample size of 18 plots. A classical linear model (i.e., implemented with the R lm() function) that includes fixed factors for block and the rows and columns of the Latin square design is over‐parameterized such that some factor levels cannot be estimated. Mixed‐effects models can be useful for the analysis of small datasets like this, since treating factors as random effects requires fewer degrees of freedom because a single variance component (costing one degree of freedom) is estimated for each factor regardless of the number of levels. However, for this experimental design a mixed‐effects ANOVA with random effects for block, row, and column (fitted with the R lme4 package lmer() function) warns of singularities and contains estimates of zero for variance components for both block and column (see Appendix S1: R Markdown [pp. 10–14]). There was, therefore, no option but to fit simpler mixed‐effects model: the small sample size was able to support a reduced model with a fixed effect for treatment and a random effect for block as reported in the main text (fitted using the lme() function from the nlme package for R). Importantly, the coefficients of interest—the treatment means (or differences in means) and their confidence intervals—were virtually unchanged by the different model formulations and so our results are insensitive to these fine details of model formulation.

**TABLE A1:** Response variables included in the statistical analysis.

Response variable	Description
MAOM carbon stocks	Amount of carbon (Mg C/ha) stored in the MAOM fraction in the top 5 cm of soil
POM carbon stocks	Amount of carbon (Mg C/ha) stored in the POM fraction in the top 5 cm of soil
Total organic carbon stocks	Amount of carbon (Mg C/ha) stored in both the MAOM and POM fractions in the top 5 cm of soil
Inorganic carbon stocks	Amount of carbon (Mg C/ha) stored in the inorganic fraction in the top 5 cm of soil
Total carbon stocks	Amount of carbon (Mg C/ha) stored in both organic and inorganic carbon stocks in the top 5 cm of soil
MAOM nitrogen stocks	Amount of nitrogen (kg N/m^2^) stored in the MAOM fraction in the top 5 cm of soil
POM nitrogen stocks	Amount of nitrogen (kg N/m^2^) stored in the POM fraction in the top 5 cm of soil
Total nitrogen stocks	Amount of nitrogen (kg N/m^2^) stored in both the MAOM and POM fractions in the top 5 cm of soil. Based on the assumption that most soil nitrogen is stored in an organic form (Post et al. 1985)
MAOM C/N ratio	Ratio of carbon to nitrogen in the MAOM fraction in the top 5 cm of soil
POM C/N ratio	Ratio of carbon to nitrogen in the POM fraction in the top 5 cm of soil
Total C/N ratio	Ratio of carbon to nitrogen in the top 5 cm of bulk soil
pH	Measure of acidity/alkalinity of the samples
Bulk density	The mass of soil divided by the volume of the soil

**TABLE A2:** Grazing treatment (grazed vs. ungrazed) ANOVA table.

Response variable	*F*_1,15_	*p*
MAOM C stocks (Mg C/ha)	0.001	0.980
POM C stocks (Mg C/ha)	0.177	0.679
SOC (Mg C/ha)	0.021	0.886
Inorganic C (Mg C/ha)	0.241	0.630
MAOM N stocks (kg N/ha)	1.28	0.277
POM N stocks (kg N/ha)	1.17	0.296
Total N (kg N/ha)	1.53	0.235
MAOM C:N ratio	4.69	0.047
POM C:N ratio	0.143	0.711
SOC C:N ratio	1.29	0.274
pH	2.52	0.134
Bulk density	< 0.001	0.976

**TABLE A3:** Grazing type (spring, autumn, and ungrazed) ANOVA table.

Response variable	*F*_2,14_	*p*
MAOM C stocks (Mg C/ha)	0.092	0.912
POM C stocks (Mg C/ha)	0.885	0.435
SOC (Mg C/ha)	0.330	0.724
Inorganic C (Mg C/ha)	0.693	0.517
MAOM N stocks (kg N/ha)	0.728	0.500
POM N stocks (kg N/ha)	0.773	0.480
Total N (kg N/ha)	0.812	0.464
MAOM C:N ratio	4.93	0.024
POM C:N ratio	0.247	0.784
SOC C:N ratio	1.72	0.215
pH	3.56	0.056
Bulk density	1.30	0.305
