# Supplementary material for: Seasonal Sheep Grazing Does Not Enhance Stable or Total Soil Carbon Stocks in a Long‐Term Calcareous Grassland Experiment
Source: Ecol Evol. 2025 Jun 30;15(7):e71582. doi: 10.1002/ece3.71582 (PMC12207319; doi:10.1002/ece3.71582)
Supplement: Supplementary file 1 — Appendix S1 [file ECE3-15-e71582-s002.pdf]

Gibson soil analysis

Table of Contents: Part 1 - Carbon Stocks Section 1 - MAOM C Stocks Section 2 - POM C Stocks Section 3 - Total C Stocks

Part 2 - Nitrogen Stocks Section 1 - MAOM N Stocks Section 2- POM N Stocks Section 3 - Total N Stocks

Part 3 - C/N Ratio Section 1 - MAOM C/N Ratio Section 2 - POM C/N Ratio Section 3 - Total C/N Ratio

Part 4 - Other soil properties Section 1 - pH Section 2 - Bulk Density Section 3 - Inorganic C

In the data set, the following columns are defined: Treatment = Factor w/ 2 levels ("Grazed", "Ungrazed") Type = Factor w/ 4 levels ("Gibson Ungrazed", "Gibson Spring", "Gibson Autumn", "Raindrop Ungrazed")

Other important definitions: MAOM = Mineral-associated organic matter, the more stable long-term fraction of organic matter POM = Particulate organic matter, the less stable fraction of organic matter

Load relevant packages and data

```
library(ggplot2)
library(tidyr)
library(dplyr)
library(rlang)
library(tidyverse)
library(nlme)
library(knitr)
library(survey)
library(MASS)
library(lme4)

citation()
```

```
##
## To cite R in publications use:
##
##   R Core Team (2023). R: A language and environment for statistical
##   computing. R Foundation for Statistical Computing, Vienna, Austria.
##   URL https://www.R-project.org/.
##
## A BibTeX entry for LaTeX users is
##
##   @Manual{,
##     title = {R: A Language and Environment for Statistical Computing},
##     author = {{R Core Team}},
##     organization = {R Foundation for Statistical Computing},
##     address = {Vienna, Austria},
##     year = {2023},
##     url = {https://www.R-project.org/},
##   }
##
## We have invested a lot of time and effort in creating R, please cite it
## when using it for data analysis. See also 'citation("pkgname")' for
```

```
## citing R packages.
```

```r
rm(list = ls(all.names = TRUE)) # Clear workspace
options(width = 58, show.signif.stars = TRUE) # R options
theme_set(theme_bw()) # Black and white figure theme

completedata <- read.csv("Fractionation_analysis.csv")

completedata2 <- completedata[c(1,2,3,4,5,6,7,8,9,10,11,12,13,14,15,16,17,18),]
completedata2$Block <- factor(completedata2$Block)
completedata2$Row <- factor(completedata2$Row)
completedata2$Column <- factor(completedata2$Column)
```

Calculating means and confidence intervals

```r
MAOMstockSummary <- completedata2 %>%
  group_by(Treatment) %>%
  summarise(
    Mean = mean(MAOM.C.Stock..tons.ha.),
    SD = sd(MAOM.C.Stock..tons.ha.),
    N = n(), # Calculate the sample size for each group
    SEM = SD / sqrt(N), # Calculate the Standard Error of the Mean (SEM)
    CI_Lower = Mean - qt(0.975, df=N-1) * SEM, # Calculate the lower bound of the 95% CI
    CI_Upper = Mean + qt(0.975, df=N-1) * SEM # Calculate the upper bound of the 95% CI
  )
MAOMstockSummary
```

```
## # A tibble: 2 x 7
##   Treatment  Mean    SD     N   SEM CI_Lower CI_Upper
##   <chr>     <dbl> <dbl> <int> <dbl>    <dbl>    <dbl>
## 1 Grazed     29.2  2.87    12 0.828     27.4     31.0
## 2 Ungrazed   29.1  2.35     6 0.961     26.7     31.6
```

```r
MAOMstockSummary2 <- completedata2 %>%
  group_by(Type) %>%
  summarise(
    Mean = mean(MAOM.C.Stock..tons.ha.),
    SD = sd(MAOM.C.Stock..tons.ha.),
    N = n(), # Calculate the sample size for each group
    SEM = SD / sqrt(N), # Calculate the Standard Error of the Mean (SEM)
    CI_Lower = Mean - qt(0.975, df=N-1) * SEM, # Calculate the lower bound of the 95% CI
    CI_Upper = Mean + qt(0.975, df=N-1) * SEM # Calculate the upper bound of the 95% CI
  )
MAOMstockSummary2
```

```
## # A tibble: 3 x 7
##   Type              Mean    SD     N   SEM CI_Lower CI_Upper
##   <chr>            <dbl> <dbl> <int> <dbl>    <dbl>    <dbl>
## 1 Autumn-grazed     28.8  1.47     6 0.599     27.3     30.4
## 2 Spring-grazed     29.6  3.95     6 1.61      25.4     33.7
## 3 Ungrazed Cont~    29.1  2.35     6 0.961     26.7     31.6
```

```r
POMstockSummary <- completedata2 %>%
  group_by(Treatment) %>%
  summarise(
    Mean = mean(POM.C.Stock..tons.ha.),
    SD = sd(POM.C.Stock..tons.ha.),
```

```r
    N = n(), # Calculate the sample size for each group
    SEM = SD / sqrt(N), # Calculate the Standard Error of the Mean (SEM)
    CI_Lower = Mean - qt(0.975, df=N-1) * SEM, # Calculate the lower bound of the 95% CI
    CI_Upper = Mean + qt(0.975, df=N-1) * SEM # Calculate the upper bound of the 95% CI
  )
POMstockSummary
```

```
## # A tibble: 2 x 7
##   Treatment  Mean    SD     N   SEM CI_Lower CI_Upper
##   <chr>     <dbl> <dbl> <int> <dbl>    <dbl>    <dbl>
## 1 Grazed     7.32  1.73    12 0.499     6.22     8.42
## 2 Ungrazed   7.66  1.40     6 0.572     6.19     9.13
```

```r
POMstockSummary2 <- completedata2 %>%
  group_by(Type) %>%
  summarise(
    Mean = mean(POM.C.Stock..tons.ha.),
    SD = sd(POM.C.Stock..tons.ha.),
    N = n(), # Calculate the sample size for each group
    SEM = SD / sqrt(N), # Calculate the Standard Error of the Mean (SEM)
    CI_Lower = Mean - qt(0.975, df=N-1) * SEM, # Calculate the lower bound of the 95% CI
    CI_Upper = Mean + qt(0.975, df=N-1) * SEM # Calculate the upper bound of the 95% CI
  )
POMstockSummary2
```

```
## # A tibble: 3 x 7
##   Type             Mean    SD     N   SEM CI_Lower CI_Upper
##   <chr>           <dbl> <dbl> <int> <dbl>    <dbl>    <dbl>
## 1 Autumn-grazed    6.74 0.956     6 0.390     5.73     7.74
## 2 Spring-grazed    7.90 2.20      6 0.898     5.59    10.2
## 3 Ungrazed Cont~   7.66 1.40      6 0.572     6.19     9.13
```

```r
SOCstockSummary <- completedata2 %>%
  group_by(Treatment) %>%
  summarise(
    Mean = mean(Total.SOM.C.Stock..tons.ha.),
    SD = sd(Total.SOM.C.Stock..tons.ha.),
    N = n(), # Calculate the sample size for each group
    SEM = SD / sqrt(N), # Calculate the Standard Error of the Mean (SEM)
    CI_Lower = Mean - qt(0.975, df=N-1) * SEM, # Calculate the lower bound of the 95% CI
    CI_Upper = Mean + qt(0.975, df=N-1) * SEM # Calculate the upper bound of the 95% CI
  )
SOCstockSummary
```

```
## # A tibble: 2 x 7
##   Treatment  Mean    SD     N   SEM CI_Lower CI_Upper
##   <chr>     <dbl> <dbl> <int> <dbl>    <dbl>    <dbl>
## 1 Grazed     36.5  4.25    12  1.23     33.8     39.2
## 2 Ungrazed   36.8  2.96     6  1.21     33.7     39.9
```

```r
SOCstockSummary2 <- completedata2 %>%
  group_by(Type) %>%
  summarise(
    Mean = mean(Total.SOM.C.Stock..tons.ha.),
    SD = sd(Total.SOM.C.Stock..tons.ha.),
    N = n(), # Calculate the sample size for each group
```

```
    SEM = SD / sqrt(N), # Calculate the Standard Error of the Mean (SEM)
    CI_Lower = Mean - qt(0.975, df=N-1) * SEM, # Calculate the lower bound of the 95% CI
    CI_Upper = Mean + qt(0.975, df=N-1) * SEM # Calculate the upper bound of the 95% CI
  )
SOCstockSummary2
```

```
## # A tibble: 3 x 7
##   Type              Mean    SD     N   SEM CI_Lower CI_Upper
##   <chr>            <dbl> <dbl> <int> <dbl>    <dbl>    <dbl>
## 1 Autumn-grazed     35.6  1.56     6 0.636     33.9     37.2
## 2 Spring-grazed     37.5  5.92     6 2.42      31.3     43.7
## 3 Ungrazed Cont~    36.8  2.96     6 1.21      33.7     39.9
```

```
completedata2$Inorganic.C.stocks..tons.ha. <- completedata2$Inorganic.C.stocks..kgC.m2. * 10
InorganicstockSummary <- completedata2 %>%
  group_by(Treatment) %>%
  summarise(
    Mean = mean(Inorganic.C.stocks..tons.ha.),
    SD = sd(Inorganic.C.stocks..tons.ha.),
    N = n(), # Calculate the sample size for each group
    SEM = SD / sqrt(N), # Calculate the Standard Error of the Mean (SEM)
    CI_Lower = Mean - qt(0.975, df=N-1) * SEM, # Calculate the lower bound of the 95% CI
    CI_Upper = Mean + qt(0.975, df=N-1) * SEM # Calculate the upper bound of the 95% CI
  )
InorganicstockSummary
```

```
## # A tibble: 2 x 7
##   Treatment  Mean    SD     N   SEM CI_Lower CI_Upper
##   <chr>     <dbl> <dbl> <int> <dbl>    <dbl>    <dbl>
## 1 Grazed     5.16  1.64    12 0.474     4.12     6.20
## 2 Ungrazed   4.77  1.43     6 0.584     3.27     6.27
```

```
InorganicstockSummary2 <- completedata2 %>%
  group_by(Type) %>%
  summarise(
    Mean = mean(Inorganic.C.stocks..tons.ha.),
    SD = sd(Inorganic.C.stocks..tons.ha.),
    N = n(), # Calculate the sample size for each group
    SEM = SD / sqrt(N), # Calculate the Standard Error of the Mean (SEM)
    CI_Lower = Mean - qt(0.975, df=N-1) * SEM, # Calculate the lower bound of the 95% CI
    CI_Upper = Mean + qt(0.975, df=N-1) * SEM # Calculate the upper bound of the 95% CI
  )
InorganicstockSummary2
```

```
## # A tibble: 3 x 7
##   Type              Mean    SD     N   SEM CI_Lower CI_Upper
##   <chr>            <dbl> <dbl> <int> <dbl>    <dbl>    <dbl>
## 1 Autumn-grazed     4.68 0.869     6 0.355     3.76     5.59
## 2 Spring-grazed     5.65 2.15      6 0.877     3.39     7.90
## 3 Ungrazed Cont~    4.77 1.43      6 0.584     3.27     6.27
```

```
MAOMNStockSummary <- completedata2 %>%
  group_by(Treatment) %>%
  summarise(
    Mean = mean(MAOM.N.Stock..kgN.m2.),
    SD = sd(MAOM.N.Stock..kgN.m2.),
```

```
    N = n(), # Calculate the sample size for each group
    SEM = SD / sqrt(N), # Calculate the Standard Error of the Mean (SEM)
    CI_Lower = Mean - qt(0.975, df=N-1) * SEM, # Calculate the lower bound of the 95% CI
    CI_Upper = Mean + qt(0.975, df=N-1) * SEM # Calculate the upper bound of the 95% CI
  )
MAOMNStockSummary
```

```
## # A tibble: 2 x 7
##   Treatment  Mean     SD     N     SEM CI_Lower CI_Upper
##   <chr>     <dbl>  <dbl> <int>   <dbl>    <dbl>    <dbl>
## 1 Grazed    0.242 0.0208    12 0.00601    0.228    0.255
## 2 Ungrazed  0.253 0.0222     6 0.00906    0.230    0.276
```

```
MAOMNStockSummary2 <- completedata2 %>%
  group_by(Type) %>%
  summarise(
    Mean = mean(MAOM.N.Stock..kgN.m2.),
    SD = sd(MAOM.N.Stock..kgN.m2.),
    N = n(), # Calculate the sample size for each group
    SEM = SD / sqrt(N), # Calculate the Standard Error of the Mean (SEM)
    CI_Lower = Mean - qt(0.975, df=N-1) * SEM, # Calculate the lower bound of the 95% CI
    CI_Upper = Mean + qt(0.975, df=N-1) * SEM # Calculate the upper bound of the 95% CI
  )
MAOMNStockSummary2
```

```
## # A tibble: 3 x 7
##   Type          Mean     SD     N     SEM CI_Lower CI_Upper
##   <chr>        <dbl>  <dbl> <int>   <dbl>    <dbl>    <dbl>
## 1 Autumn-gra~ 0.245 0.0195     6 0.00796    0.224    0.265
## 2 Spring-gra~ 0.239 0.0235     6 0.00960    0.214    0.263
## 3 Ungrazed C~ 0.253 0.0222     6 0.00906    0.230    0.276
```

```
POMNStockSummary <- completedata2 %>%
  group_by(Treatment) %>%
  summarise(
    Mean = mean(POM.N.Stock..kgN.m2.),
    SD = sd(POM.N.Stock..kgN.m2.),
    N = n(), # Calculate the sample size for each group
    SEM = SD / sqrt(N), # Calculate the Standard Error of the Mean (SEM)
    CI_Lower = Mean - qt(0.975, df=N-1) * SEM, # Calculate the lower bound of the 95% CI
    CI_Upper = Mean + qt(0.975, df=N-1) * SEM # Calculate the upper bound of the 95% CI
  )
POMNStockSummary
```

```
## # A tibble: 2 x 7
##   Treatment   Mean      SD     N     SEM CI_Lower CI_Upper
##   <chr>      <dbl>   <dbl> <int>   <dbl>    <dbl>    <dbl>
## 1 Grazed    0.0149 0.00259    12 7.49e-4   0.0132   0.0165
## 2 Ungrazed  0.0161 0.00114     6 4.64e-4   0.0149   0.0173
```

```
POMNStockSummary2 <- completedata2 %>%
  group_by(Type) %>%
  summarise(
    Mean = mean(POM.N.Stock..kgN.m2.),
    SD = sd(POM.N.Stock..kgN.m2.),
    N = n(), # Calculate the sample size for each group
```

```
    SEM = SD / sqrt(N), # Calculate the Standard Error of the Mean (SEM)
    CI_Lower = Mean - qt(0.975, df=N-1) * SEM, # Calculate the lower bound of the 95% CI
    CI_Upper = Mean + qt(0.975, df=N-1) * SEM # Calculate the upper bound of the 95% CI
  )
POMNStockSummary2
```

```
## # A tibble: 3 x 7
##   Type         Mean      SD     N     SEM CI_Lower CI_Upper
##   <chr>       <dbl>   <dbl> <int>   <dbl>    <dbl>    <dbl>
## 1 Autumn-g~ 0.0144 0.00329     6 1.34e-3   0.0110   0.0179
## 2 Spring-g~ 0.0153 0.00188     6 7.67e-4   0.0133   0.0173
## 3 Ungrazed~ 0.0161 0.00114     6 4.64e-4   0.0149   0.0173
```

```
TotalNStockSummary <- completedata2 %>%
  group_by(Treatment) %>%
  summarise(
    Mean = mean(Total.SOM.N.Stock..kgN.m2.),
    SD = sd(Total.SOM.N.Stock..kgN.m2.),
    N = n(), # Calculate the sample size for each group
    SEM = SD / sqrt(N), # Calculate the Standard Error of the Mean (SEM)
    CI_Lower = Mean - qt(0.975, df=N-1) * SEM, # Calculate the lower bound of the 95% CI
    CI_Upper = Mean + qt(0.975, df=N-1) * SEM # Calculate the upper bound of the 95% CI
  )
TotalNStockSummary
```

```
## # A tibble: 2 x 7
##   Treatment  Mean     SD     N     SEM CI_Lower CI_Upper
##   <chr>     <dbl>  <dbl> <int>   <dbl>    <dbl>    <dbl>
## 1 Grazed    0.257 0.0209    12 0.00602    0.243    0.270
## 2 Ungrazed  0.269 0.0229     6 0.00935    0.245    0.293
```

```
TotalNStockSummary2 <- completedata2 %>%
  group_by(Type) %>%
  summarise(
    Mean = mean(Total.SOM.N.Stock..kgN.m2.),
    SD = sd(Total.SOM.N.Stock..kgN.m2.),
    N = n(), # Calculate the sample size for each group
    SEM = SD / sqrt(N), # Calculate the Standard Error of the Mean (SEM)
    CI_Lower = Mean - qt(0.975, df=N-1) * SEM, # Calculate the lower bound of the 95% CI
    CI_Upper = Mean + qt(0.975, df=N-1) * SEM # Calculate the upper bound of the 95% CI
  )
TotalNStockSummary2
```

```
## # A tibble: 3 x 7
##   Type           Mean     SD     N     SEM CI_Lower CI_Upper
##   <chr>         <dbl>  <dbl> <int>   <dbl>    <dbl>    <dbl>
## 1 Autumn-gra~ 0.259 0.0191     6 0.00779    0.239    0.279
## 2 Spring-gra~ 0.254 0.0240     6 0.00981    0.229    0.279
## 3 Ungrazed C~ 0.269 0.0229     6 0.00935    0.245    0.293
```

```
MAOMRatioSummary1 <- completedata2 %>%
  group_by(Treatment) %>%
  summarise(
    Mean = mean(MAOM.C.N.Ratio),
    SD = sd(MAOM.C.N.Ratio),
    N = n(), # Calculate the sample size for each group
```

```r
    SEM = SD / sqrt(N), # Calculate the Standard Error of the Mean (SEM)
    CI_Lower = Mean - qt(0.975, df=N-1) * SEM, # Calculate the lower bound of the 95% CI
    CI_Upper = Mean + qt(0.975, df=N-1) * SEM # Calculate the upper bound of the 95% CI
  )
MAOMRatioSummary1
```

```
## # A tibble: 2 x 7
##   Treatment  Mean    SD     N   SEM CI_Lower CI_Upper
##   <chr>     <dbl> <dbl> <int> <dbl>    <dbl>    <dbl>
## 1 Grazed     12.1 0.656    12 0.189     11.7     12.5
## 2 Ungrazed   11.5 0.456     6 0.186     11.1     12.0
```

```r
MAOMRatioSummary2 <- completedata2 %>%
  group_by(Type) %>%
  summarise(
    Mean = mean(MAOM.C.N.Ratio),
    SD = sd(MAOM.C.N.Ratio),
    N = n(), # Calculate the sample size for each group
    SEM = SD / sqrt(N), # Calculate the Standard Error of the Mean (SEM)
    CI_Lower = Mean - qt(0.975, df=N-1) * SEM, # Calculate the lower bound of the 95% CI
    CI_Upper = Mean + qt(0.975, df=N-1) * SEM # Calculate the upper bound of the 95% CI
  )
MAOMRatioSummary2
```

```
## # A tibble: 3 x 7
##   Type             Mean    SD     N   SEM CI_Lower CI_Upper
##   <chr>           <dbl> <dbl> <int> <dbl>    <dbl>    <dbl>
## 1 Autumn-grazed    11.8 0.412     6 0.168     11.4     12.2
## 2 Spring-grazed    12.4 0.764     6 0.312     11.6     13.2
## 3 Ungrazed Cont~   11.5 0.456     6 0.186     11.1     12.0
```

```r
POMRatioSummary1 <- completedata2 %>%
  group_by(Treatment) %>%
  summarise(
    Mean = mean(POM.C.N.Ratio),
    SD = sd(POM.C.N.Ratio),
    N = n(), # Calculate the sample size for each group
    SEM = SD / sqrt(N), # Calculate the Standard Error of the Mean (SEM)
    CI_Lower = Mean - qt(0.975, df=N-1) * SEM, # Calculate the lower bound of the 95% CI
    CI_Upper = Mean + qt(0.975, df=N-1) * SEM # Calculate the upper bound of the 95% CI
  )
POMRatioSummary1
```

```
## # A tibble: 2 x 7
##   Treatment  Mean    SD     N   SEM CI_Lower CI_Upper
##   <chr>     <dbl> <dbl> <int> <dbl>    <dbl>    <dbl>
## 1 Grazed     49.8 11.0     12  3.17     42.8     56.8
## 2 Ungrazed   47.8  9.04     6  3.69     38.3     57.3
```

```r
POMRatioSummary2 <- completedata2 %>%
  group_by(Type) %>%
  summarise(
    Mean = mean(POM.C.N.Ratio),
    SD = sd(POM.C.N.Ratio),
    N = n(), # Calculate the sample size for each group
    SEM = SD / sqrt(N), # Calculate the Standard Error of the Mean (SEM)
```

```r
    CI_Lower = Mean - qt(0.975, df=N-1) * SEM, # Calculate the lower bound of the 95% CI
    CI_Upper = Mean + qt(0.975, df=N-1) * SEM # Calculate the upper bound of the 95% CI
  )
POMRatioSummary2
```

```
## # A tibble: 3 x 7
##   Type              Mean    SD     N   SEM CI_Lower CI_Upper
##   <chr>            <dbl> <dbl> <int> <dbl>    <dbl>    <dbl>
## 1 Autumn-grazed     47.9  9.84     6  4.02     37.6     58.3
## 2 Spring-grazed     51.6 12.7      6  5.17     38.3     64.9
## 3 Ungrazed Cont~    47.8  9.04     6  3.69     38.3     57.3
```

```r
TotalRatioSummary1 <- completedata2 %>%
  group_by(Treatment) %>%
  summarise(
    Mean = mean(C.N.Ratio),
    SD = sd(C.N.Ratio),
    N = n(), # Calculate the sample size for each group
    SEM = SD / sqrt(N), # Calculate the Standard Error of the Mean (SEM)
    CI_Lower = Mean - qt(0.975, df=N-1) * SEM, # Calculate the lower bound of the 95% CI
    CI_Upper = Mean + qt(0.975, df=N-1) * SEM # Calculate the upper bound of the 95% CI
  )
TotalRatioSummary1
```

```
## # A tibble: 2 x 7
##   Treatment  Mean    SD     N   SEM CI_Lower CI_Upper
##   <chr>     <dbl> <dbl> <int> <dbl>    <dbl>    <dbl>
## 1 Grazed     15.5  2.33    12 0.673     14.0     17.0
## 2 Ungrazed   14.4  1.14     6 0.464     13.2     15.6
```

```r
TotalRatioSummary2 <- completedata2 %>%
  group_by(Type) %>%
  summarise(
    Mean = mean(C.N.Ratio),
    SD = sd(C.N.Ratio),
    N = n(), # Calculate the sample size for each group
    SEM = SD / sqrt(N), # Calculate the Standard Error of the Mean (SEM)
    CI_Lower = Mean - qt(0.975, df=N-1) * SEM, # Calculate the lower bound of the 95% CI
    CI_Upper = Mean + qt(0.975, df=N-1) * SEM # Calculate the upper bound of the 95% CI
  )
TotalRatioSummary2
```

```
## # A tibble: 3 x 7
##   Type              Mean    SD     N   SEM CI_Lower CI_Upper
##   <chr>            <dbl> <dbl> <int> <dbl>    <dbl>    <dbl>
## 1 Autumn-grazed     14.7 0.946     6 0.386     13.7     15.7
## 2 Spring-grazed     16.4 3.06      6 1.25      13.1     19.6
## 3 Ungrazed Cont~    14.4 1.14      6 0.464     13.2     15.6
```

```r
pHSummary1 <- completedata2 %>%
  group_by(Treatment) %>%
  summarise(
    Mean = mean(pH),
    SD = sd(pH),
    N = n(), # Calculate the sample size for each group
    SEM = SD / sqrt(N), # Calculate the Standard Error of the Mean (SEM)
```

```
    CI_Lower = Mean - qt(0.975, df=N-1) * SEM, # Calculate the lower bound of the 95% CI
    CI_Upper = Mean + qt(0.975, df=N-1) * SEM # Calculate the upper bound of the 95% CI
  )
pHSummary1
```

```
## # A tibble: 2 x 7
##   Treatment  Mean     SD     N    SEM CI_Lower CI_Upper
##   <chr>     <dbl>  <dbl> <int>  <dbl>    <dbl>    <dbl>
## 1 Grazed     7.65 0.103     12 0.0298     7.58     7.71
## 2 Ungrazed   7.57 0.0997     6 0.0407     7.46     7.67
```

```
pHSummary2 <- completedata2 %>%
  group_by(Type) %>%
  summarise(
    Mean = mean(pH),
    SD = sd(pH),
    N = n(), # Calculate the sample size for each group
    SEM = SD / sqrt(N), # Calculate the Standard Error of the Mean (SEM)
    CI_Lower = Mean - qt(0.975, df=N-1) * SEM, # Calculate the lower bound of the 95% CI
    CI_Upper = Mean + qt(0.975, df=N-1) * SEM # Calculate the upper bound of the 95% CI
  )
pHSummary2
```

```
## # A tibble: 3 x 7
##   Type           Mean     SD     N    SEM CI_Lower CI_Upper
##   <chr>         <dbl>  <dbl> <int>  <dbl>    <dbl>    <dbl>
## 1 Autumn-graz~  7.60 0.0989     6 0.0404     7.49     7.70
## 2 Spring-graz~  7.70 0.0809     6 0.0330     7.62     7.79
## 3 Ungrazed Co~  7.57 0.0997     6 0.0407     7.46     7.67
```

```
BDSummary1 <- completedata2 %>%
  group_by(Treatment) %>%
  summarise(
    Mean = mean(BD),
    SD = sd(BD),
    N = n(), # Calculate the sample size for each group
    SEM = SD / sqrt(N), # Calculate the Standard Error of the Mean (SEM)
    CI_Lower = Mean - qt(0.975, df=N-1) * SEM, # Calculate the lower bound of the 95% CI
    CI_Upper = Mean + qt(0.975, df=N-1) * SEM # Calculate the upper bound of the 95% CI
  )
BDSummary1
```

```
## # A tibble: 2 x 7
##   Treatment  Mean     SD     N    SEM CI_Lower CI_Upper
##   <chr>     <dbl>  <dbl> <int>  <dbl>    <dbl>    <dbl>
## 1 Grazed    0.780 0.0813    12 0.0235    0.729    0.832
## 2 Ungrazed  0.781 0.0704     6 0.0287    0.708    0.855
```

```
BDSummary2 <- completedata2 %>%
  group_by(Type) %>%
  summarise(
    Mean = mean(BD),
    SD = sd(BD),
    N = n(), # Calculate the sample size for each group
    SEM = SD / sqrt(N), # Calculate the Standard Error of the Mean (SEM)
    CI_Lower = Mean - qt(0.975, df=N-1) * SEM, # Calculate the lower bound of the 95% CI
```

```r
    CI_Upper = Mean + qt(0.975, df=N-1) * SEM # Calculate the upper bound of the 95% CI
  )
BDSummary2
```

```
## # A tibble: 3 x 7
##   Type           Mean     SD     N    SEM CI_Lower CI_Upper
##   <chr>         <dbl>  <dbl> <int>  <dbl>    <dbl>    <dbl>
## 1 Autumn-graz~ 0.755 0.0773     6 0.0316    0.673    0.836
## 2 Spring-graz~ 0.806 0.0835     6 0.0341    0.719    0.894
## 3 Ungrazed Co~ 0.781 0.0704     6 0.0287    0.708    0.855
```

Part 0 - Trials of different models

Section 1 - Figure out what transformation to use for the MAOM data

```r
## Model 1 - Original model with only fixed effect of treatment and random effect of Block
MAOMStockModel1 <- lme(MAOM.C.Stock..tons.ha. ~ Treatment, random = ~ 1|Block, data = completedata2)
summary(MAOMStockModel1)
```

```
## Linear mixed-effects model fit by REML
##   Data: completedata2
##        AIC      BIC    logLik
##   89.68588 92.77624 -40.84294
##
## Random effects:
##  Formula: ~1 | Block
##         (Intercept) Residual
## StdDev: 9.267791e-05 2.718552
##
## Fixed effects:  MAOM.C.Stock..tons.ha. ~ Treatment
##                         Value Std.Error DF  t-value p-value
## (Intercept)         29.199343 0.7847784 15 37.20712  0.0000
## TreatmentUngrazed -0.059268 1.3592760 15 -0.04360  0.9658
##  Correlation:
##                   (Intr)
## TreatmentUngrazed -0.577
##
## Standardized Within-Group Residuals:
##        Min         Q1        Med         Q3        Max
## -1.4865831 -0.4773317 -0.3174003  0.3305177  2.4851308
##
## Number of Observations: 18
## Number of Groups: 2
```

```r
anova(MAOMStockModel1)
```

```
##             numDF denDF   F-value p-value
## (Intercept)     1    15 2073.7455  <.0001
## Treatment       1    15    0.0019  0.9658
```

```r
plot(MAOMStockModel1)
```

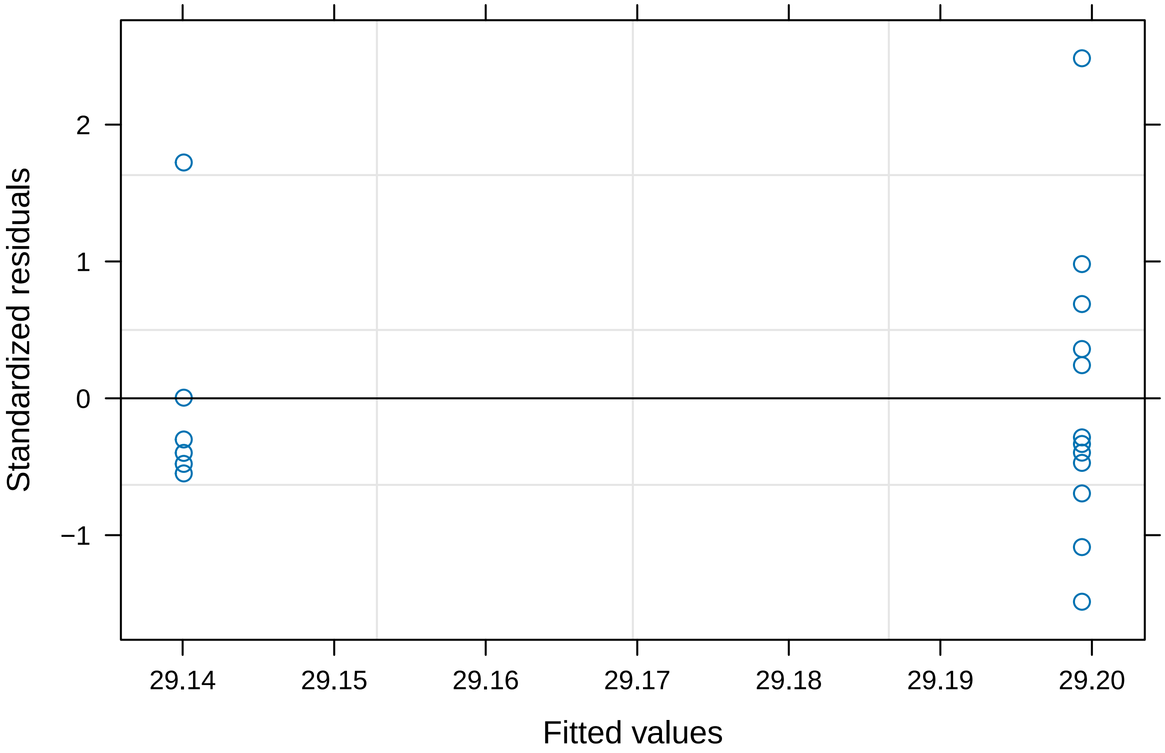


```
qqnorm(MAOMStockModel1$residuals)
```

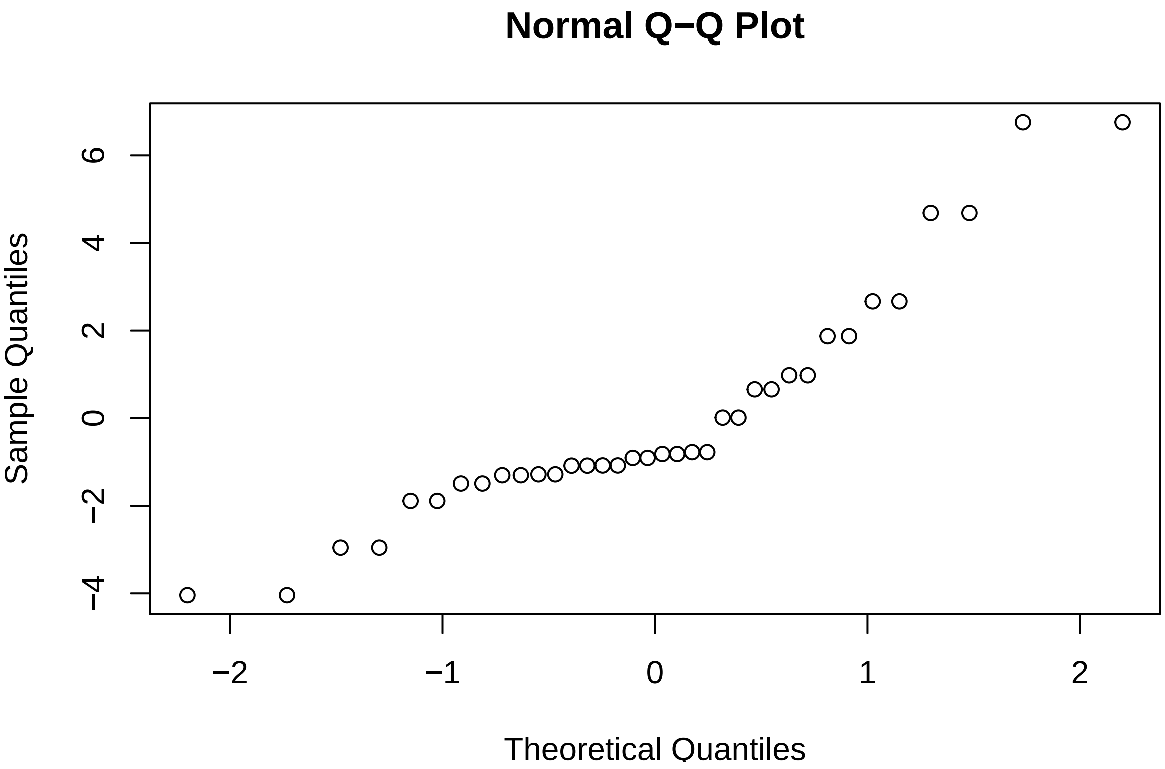

Section 2 - Run script for different model types

```
## Model 1 - Original model with only fixed effect of treatment and random effect of Block
MAOMStockModel1 <- lme(MAOM.C.Stock..tons.ha. ~ Treatment, random = ~ 1|Block, data = completedata2)
```

```r
summary(MAOMStockModel1)
```

```
## Linear mixed-effects model fit by REML
##   Data: completedata2
##        AIC      BIC    logLik
##   89.68588 92.77624 -40.84294
##
## Random effects:
##  Formula: ~1 | Block
##         (Intercept) Residual
## StdDev: 9.267791e-05 2.718552
##
## Fixed effects:  MAOM.C.Stock..tons.ha. ~ Treatment
##                       Value Std.Error DF  t-value p-value
## (Intercept)       29.199343 0.7847784 15 37.20712  0.0000
## TreatmentUngrazed -0.059268 1.3592760 15 -0.04360  0.9658
##  Correlation:
##                   (Intr)
## TreatmentUngrazed -0.577
##
## Standardized Within-Group Residuals:
##        Min         Q1        Med         Q3        Max
## -1.4865831 -0.4773317 -0.3174003  0.3305177  2.4851308
##
## Number of Observations: 18
## Number of Groups: 2
```

```r
anova(MAOMStockModel1)
```

```
##             numDF denDF   F-value p-value
## (Intercept)     1    15 2073.7455  <.0001
## Treatment       1    15    0.0019  0.9658
```

```r
plot(MAOMStockModel1)
```

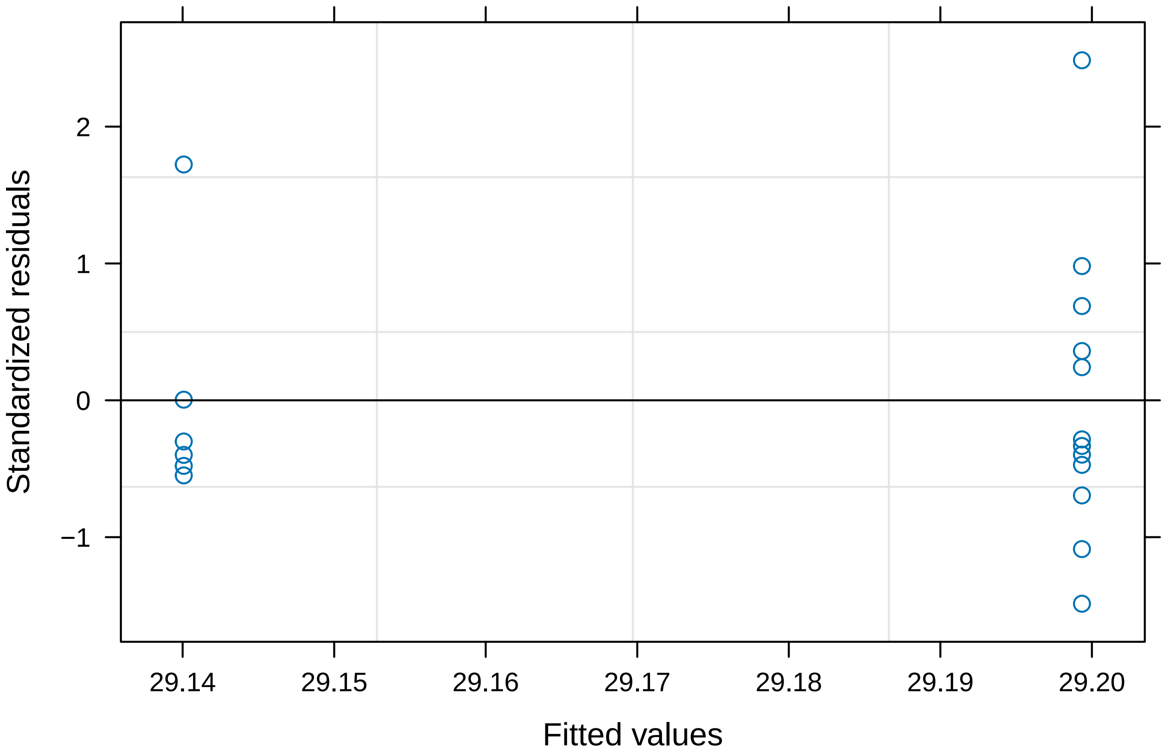


```r
qqnorm(MAOMStockModel1$residuals)
```

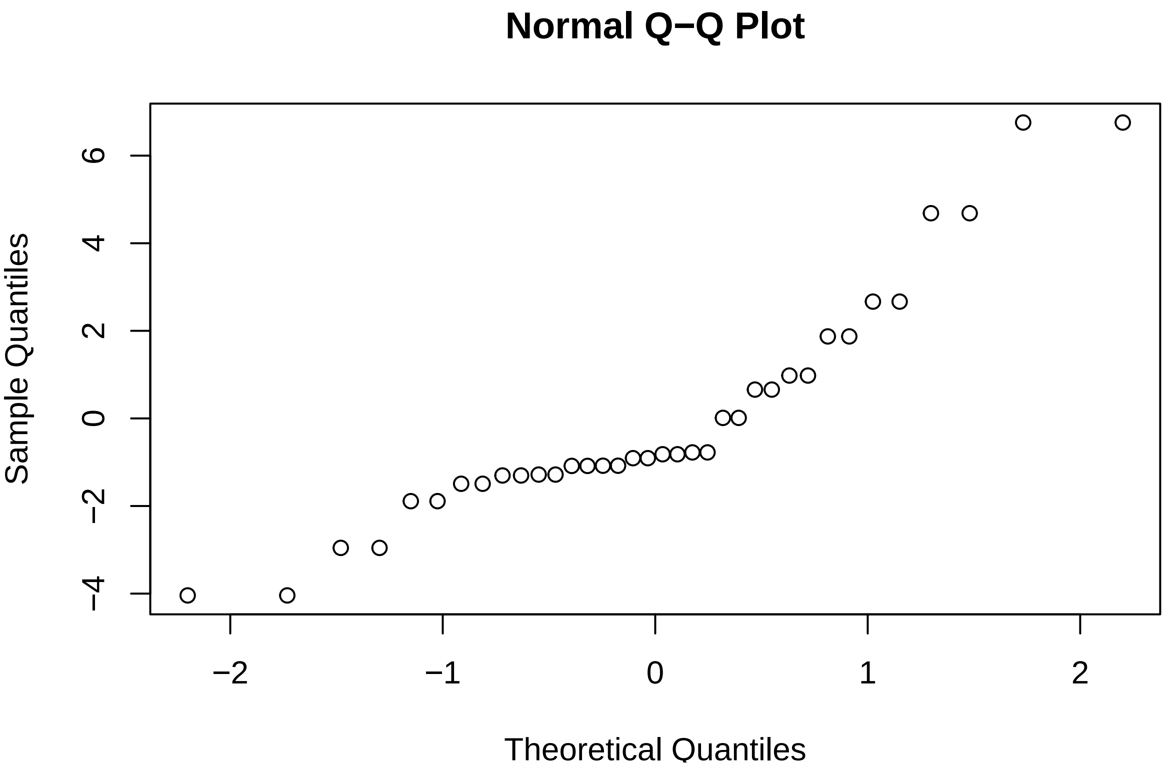


```r
## Model 2 - Adding in Row and Column as fixed effects
# MAOMStockModel2 <- lme(MAOM.C.Stock..tons.ha. ~ Row + Column + Treatment, random = ~ 1|Block, data =
# summary(MAOMStockModel2)
# anova(MAOMStockModel2)
```

```r
# plot(MAOMStockModel2)
# qqnorm(MAOMStockModel2$residuals)

## Model 3 - Considering Row and Column to be Random factors, where rows are nested within blocks and c
# MAOMStockModel3 <- lme(MAOM.C.Stock..tons.ha. ~ Treatment, random = ~ 1|Block/Row/Column, data = comp
# summary(MAOMStockModel3)
# anova(MAOMStockModel3)
# plot(MAOMStockModel3)
# qqnorm(MAOMStockModel3$residuals)


## Model 4 - Using lmer() function from lme4 package to remove nested structure of random effects
# MAOMStockModel4 <- lmer(MAOM.C.Stock..tons.ha. ~ Treatment + (1|Block) + (1|Row) + (1|Column), data =
# summary(MAOMStockModel4)
# anova(MAOMStockModel4)
# plot(MAOMStockModel4)
# qqnorm(MAOMStockModel4$residuals)
```

Section 3 - Figure out treatment vs type for each model comparison

```r
# First, for original model
MAOMStockModelTreatment1 <- lme(MAOM.C.Stock..tons.ha. ~ Treatment, random = ~ 1|Block, data = complete
MAOMStockModelType1 <- lme(MAOM.C.Stock..tons.ha. ~ Type, random = ~ 1|Block, data = completedata2)

anova(MAOMStockModelTreatment1, MAOMStockModelType1) #p-value of 0.08 so not significantly different fr
```

```
##                          Model df      AIC      BIC
## MAOMStockModelTreatment1     1  4 89.68588 92.77624
## MAOMStockModelType1          2  5 88.68761 92.22786
##                             logLik   Test  L.Ratio
## MAOMStockModelTreatment1 -40.84294
## MAOMStockModelType1      -39.34381 1 vs 2 2.998269
##                          p-value
## MAOMStockModelTreatment1
## MAOMStockModelType1       0.0834
```

```r
# Second, for model where row and column are random effects
MAOMStockModelTreatment2 <- lme(MAOM.C.Stock..tons.ha. ~ Treatment, random = ~ 1|Block/Row/Column, data
MAOMStockModelType2 <- lme(MAOM.C.Stock..tons.ha. ~ Type, random = ~ 1|Block/Row/Column, data = complet
anova(MAOMStockModelTreatment2, MAOMStockModelType2) # again, p-value of 0.08 so not significantly diff
```

```
##                          Model df      AIC     BIC
## MAOMStockModelTreatment2     1  6 93.66306 98.2986
## MAOMStockModelType2          2  7 92.68465 97.6410
##                             logLik   Test L.Ratio p-value
## MAOMStockModelTreatment2 -40.83153
## MAOMStockModelType2      -39.34233 1 vs 2 2.97841  0.0844
```

Part 1 - C Stocks Part 1 (Section 1) - MAOM C Stocks

Visualization

```r
MAOMstock <- completedata2 %>% dplyr::select(Block, ID, Type, Treatment, Study, MAOM.C.Stock..tons.ha.)
yvar <- MAOMstock$MAOM.C.Stock..tons.ha.

MAOMstockTreatment_means <- MAOMstock %>% group_by(Treatment) %>% summarise(Treatment_mean_MAOM.C.Stock
MAOMstockTreatment_means
```

```
## # A tibble: 2 x 2
##   Treatment Treatment_mean_MAOM.C.Stock..tons.ha.
##   <chr>                                     <dbl>
## 1 Grazed                                     29.2
## 2 Ungrazed                                   29.1
```

```
MAOMstockType_means <- MAOMstock %>% group_by(Type) %>% summarise(Type_mean_MAOM.C.Stock..tons.ha. = mea
MAOMstockType_means
```

```
## # A tibble: 3 x 2
##   Type             Type_mean_MAOM.C.Stock..tons.ha.
##   <chr>                                        <dbl>
## 1 Autumn-grazed                                 28.8
## 2 Spring-grazed                                 29.6
## 3 Ungrazed Control                              29.1
```

```
ggplot(MAOMstock) +  geom_point(mapping = aes(x=Treatment, y=yvar, shape = Type, col=Type)) + labs(x =
```

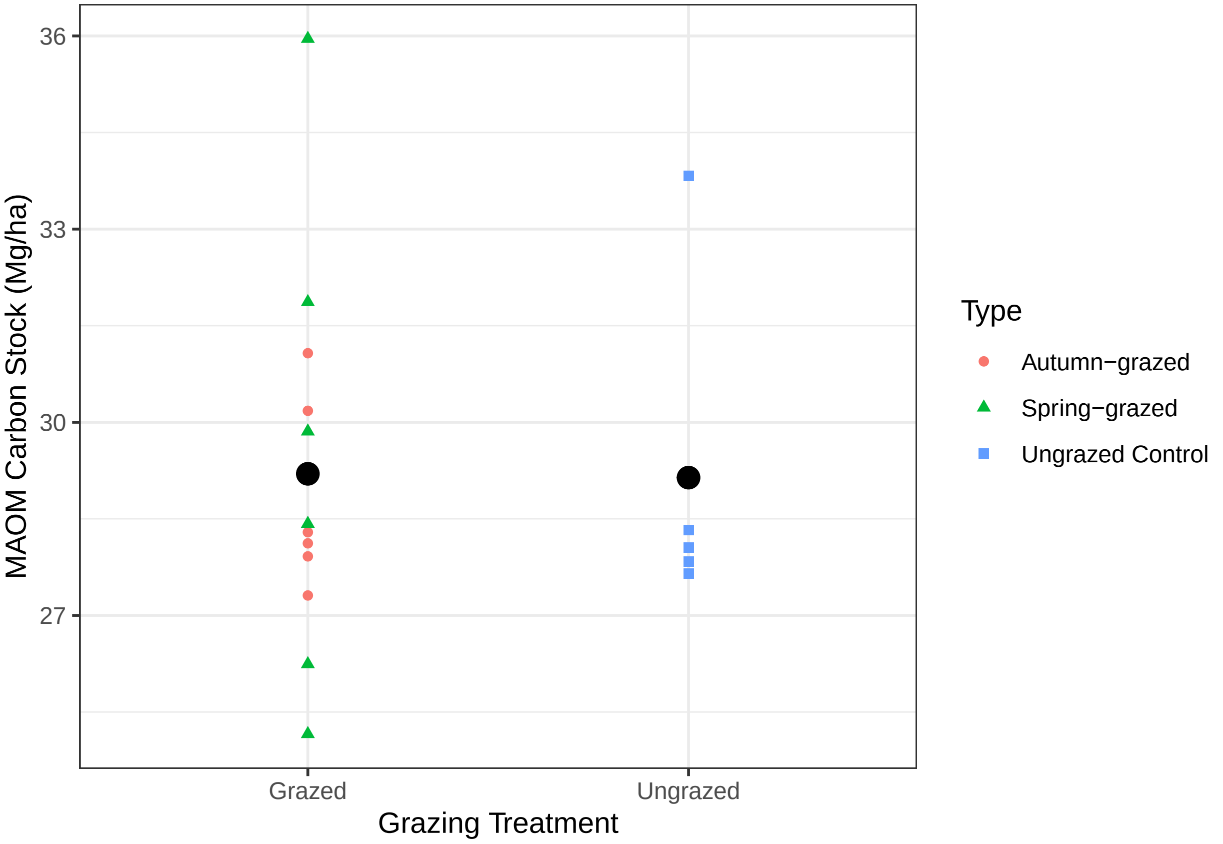


```
## Add standard deviation error bars
# First create dataset with the means and standard deviations of each of the treatment groups

MAOMstockSummary <- MAOMstock %>%
  group_by(Treatment) %>%
  summarise(
    Mean = mean(MAOM.C.Stock..tons.ha.),
    SD = sd(MAOM.C.Stock..tons.ha.)
  )
```

```r
# Base ggplot call with only the x aesthetic set globally, as it's common across all layers
ggplot(MAOMstock, aes(x=Treatment)) +
  # First geom_point layer for individual observations, specifying y aesthetic individually
  geom_point(aes(y=MAOM.C.Stock..tons.ha., shape=Type, color=Type)) +
  labs(x = "Grazing Treatment", y = "MAOM Carbon Stock (Mg/ha)") +
  # Second geom_point layer for means from MAOMstockSummary, specifying y aesthetic for Mean
  geom_point(data=MAOMstockSummary, aes(x=Treatment, y=Mean), size=3.5, color="black", alpha=1) +
  # geom_errorbar layer for error bars, using MAOMstockSummary and specifying ymin and ymax based on Me
  geom_errorbar(data=MAOMstockSummary, aes(x=Treatment, ymin=Mean-SD, ymax=Mean+SD), width=.05, color="
```

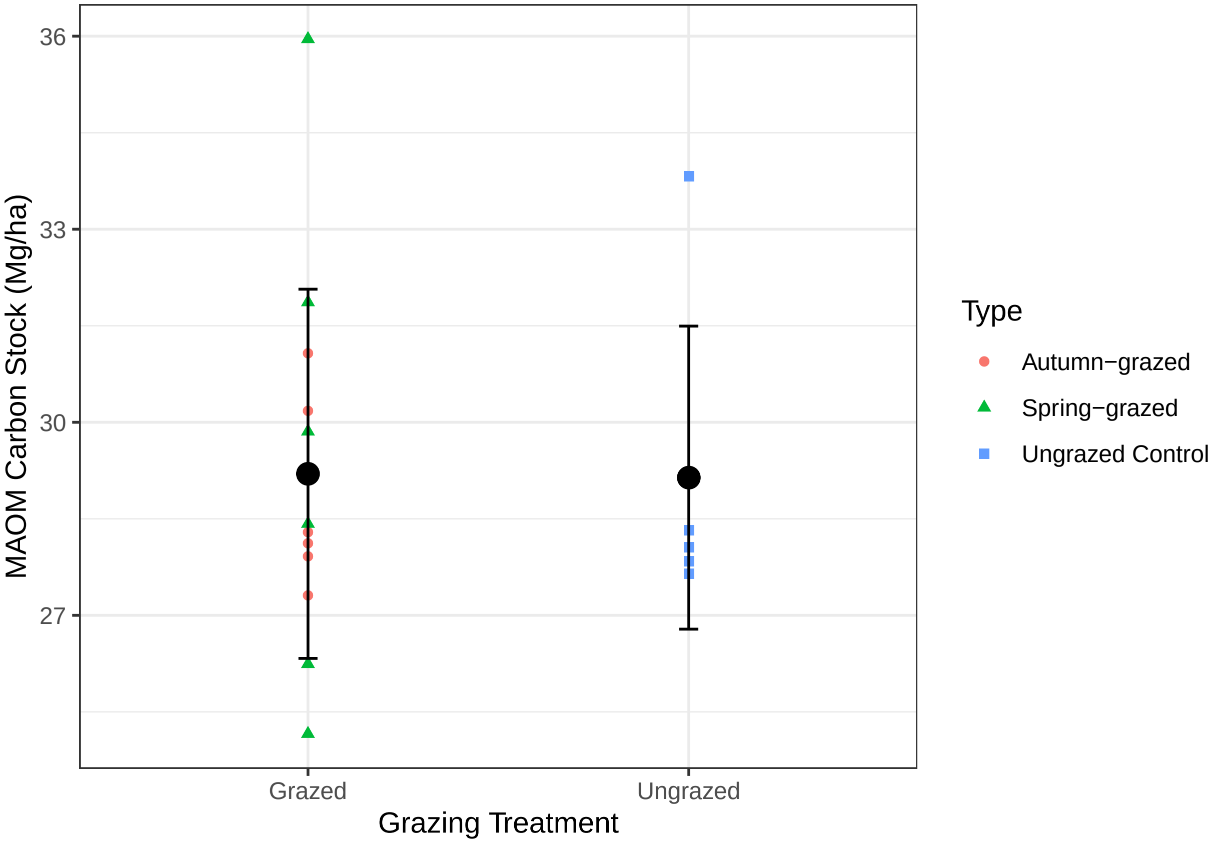


```r
### Adding confidence interval error bars
MAOMstockSummary <- MAOMstock %>%
  group_by(Treatment) %>%
  summarise(
    Mean = mean(MAOM.C.Stock..tons.ha.),
    SD = sd(MAOM.C.Stock..tons.ha.),
    N = n(), # Calculate the sample size for each group
    SEM = SD / sqrt(N), # Calculate the Standard Error of the Mean (SEM)
    CI_Lower = Mean - qt(0.975, df=N-1) * SEM, # Calculate the lower bound of the 95% CI
    CI_Upper = Mean + qt(0.975, df=N-1) * SEM # Calculate the upper bound of the 95% CI
  )

# Base ggplot call with only the x aesthetic set globally, as it's common across all layers
ggplot(MAOMstock, aes(x=Treatment)) +
  # First geom_point layer for individual observations, specifying y aesthetic individually
  geom_point(aes(y=MAOM.C.Stock..tons.ha., shape=Type, color=Type)) +
  labs(x = "Grazing Treatment", y = "MAOM Carbon Stock (Mg/ha)") +
```

```
# Second geom_point layer for means from MAOMstockSummary, specifying y aesthetic for Mean
geom_point(data=MAOMstockSummary, aes(x=Treatment, y=Mean), size=3.5, color="black", alpha=1) +
# geom_errorbar layer for 95% CI error bars, using MAOMstockSummary and specifying ymin and ymax base
geom_errorbar(data=MAOMstockSummary, aes(x=Treatment, ymin=CI_Lower, ymax=CI_Upper), width=.05, color=
```

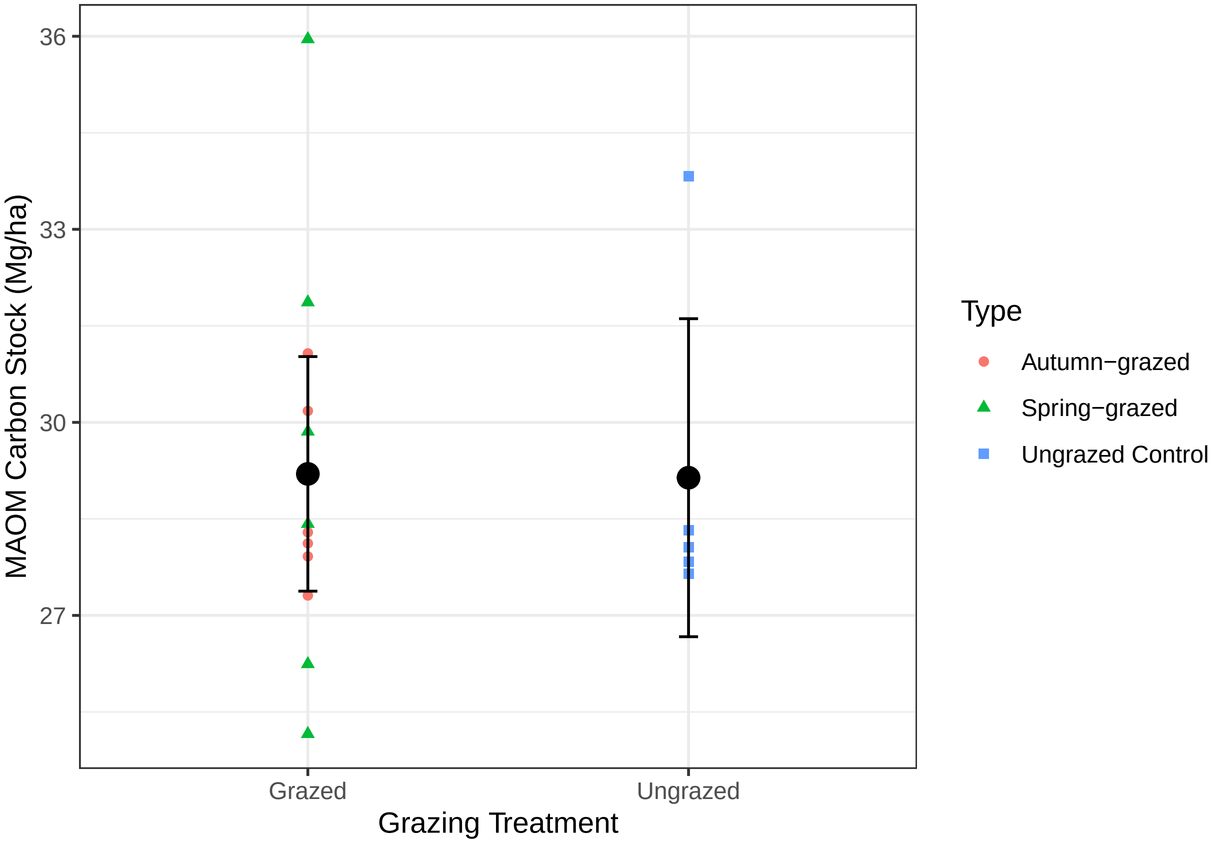


```
ggplot(MAOMstock) +  geom_point(mapping = aes(x=Type, y=yvar, shape = Type, col=Type)) + labs(x = "Grazi
```

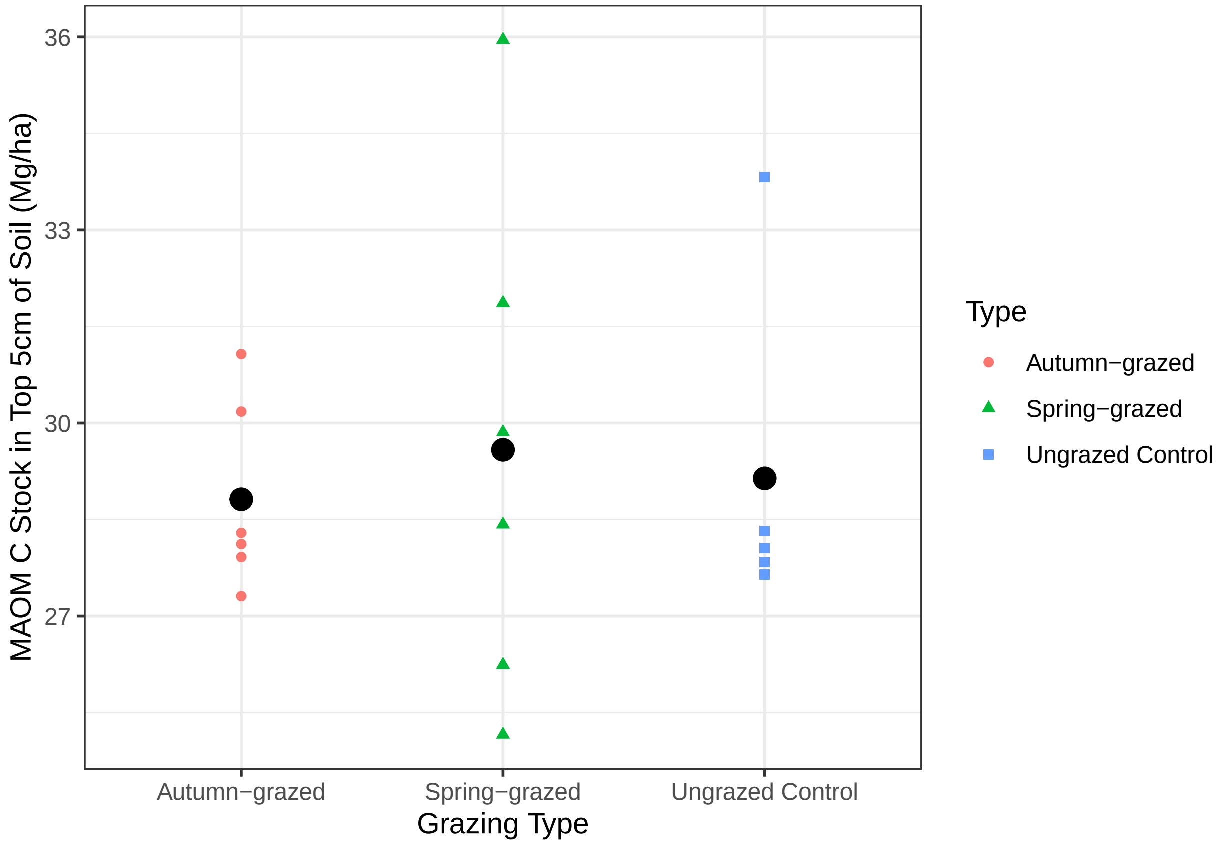


```
ggplot(MAOMstock, aes(x=Type, y=MAOM.C.Stock..tons.ha.)) + geom_boxplot(trim=FALSE) + labs(x = "Grazing
```

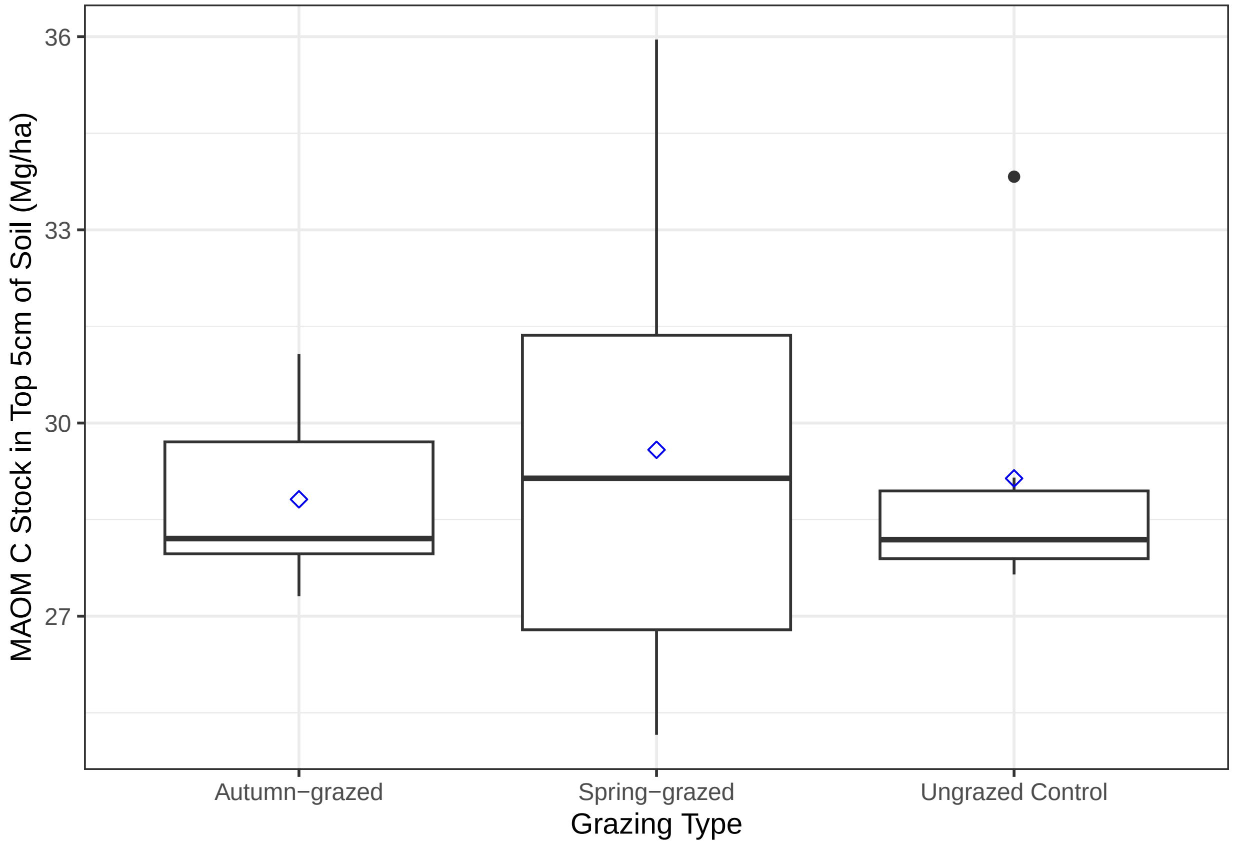


```
# Calculating 95% confidence intervals
UngrazedMAOM <- MAOMstock[c(1,2,3,10,11,12),]
1.96 * sd(UngrazedMAOM$MAOM.C.Stock..kgC.m2.)/sqrt(6)
```

```
## [1] NA
```

```
SpringMAOM <- MAOMstock[c(7,8,9,16,17,18),]
1.96 * sd(SpringMAOM$MAOM.C.Stock..kgC.m2.)/sqrt(6)
```

```
## [1] NA
```

```
AutumnMAOM <- MAOMstock[c(4,5,6,13,14,15),]
1.96 * sd(AutumnMAOM$MAOM.C.Stock..kgC.m2.)/sqrt(6)
```

```
## [1] NA
```

```
GrazedMAOM <- MAOMstock[c(4,5,6,7,8,9,13,14,15,16,17,18),]
1.96 * sd(GrazedMAOM$MAOM.C.Stock..kgC.m2.)/sqrt(6)
```

```
## [1] NA
```

Visual conclusions: Grazing treatment does not appear to have a significant effect on MAOM C stock, and grazing type does not seem to be important either. The only difference is that the variation is much higher in the spring-grazed type than in either the autumn-grazed and ungrazed controls.

Mixed Effects Model looking at the effects of just grazing type (Spring vs Autumn vs Ungrazed Control)

```
MAOMStockModel1 <- lme(MAOM.C.Stock..kgC.m2. ~ Type, random = ~ 1|Block, data = MAOMstock)
```

```
## Error in eval(predvars, data, env): object 'MAOM.C.Stock..kgC.m2.' not found
```

```r
summary(MAOMStockModel1)
```

```
## Linear mixed-effects model fit by REML
##   Data: completedata2
##        AIC      BIC    logLik
##   89.68588 92.77624 -40.84294
##
## Random effects:
##  Formula: ~1 | Block
##         (Intercept) Residual
## StdDev: 9.267791e-05 2.718552
##
## Fixed effects:  MAOM.C.Stock..tons.ha. ~ Treatment
##                        Value Std.Error DF  t-value p-value
## (Intercept)        29.199343 0.7847784 15 37.20712  0.0000
## TreatmentUngrazed -0.059268 1.3592760 15 -0.04360  0.9658
##  Correlation:
##                   (Intr)
## TreatmentUngrazed -0.577
##
## Standardized Within-Group Residuals:
##        Min         Q1        Med         Q3        Max
## -1.4865831 -0.4773317 -0.3174003  0.3305177  2.4851308
##
## Number of Observations: 18
## Number of Groups: 2
```

```r
anova(MAOMStockModel1)
```

```
##             numDF denDF   F-value p-value
## (Intercept)     1    15 2073.7455  <.0001
## Treatment       1    15    0.0019  0.9658
```

```r
plot(MAOMStockModel1)
```

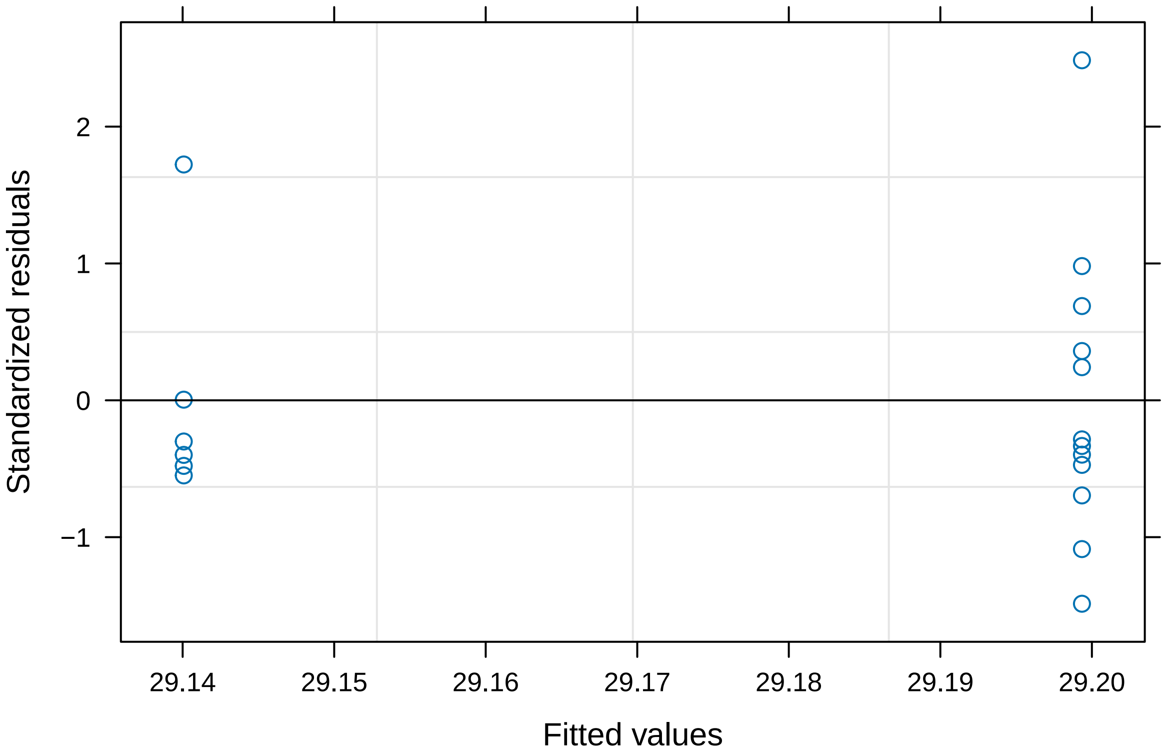


```r
qqnorm(MAOMStockModel1$residuals)
```

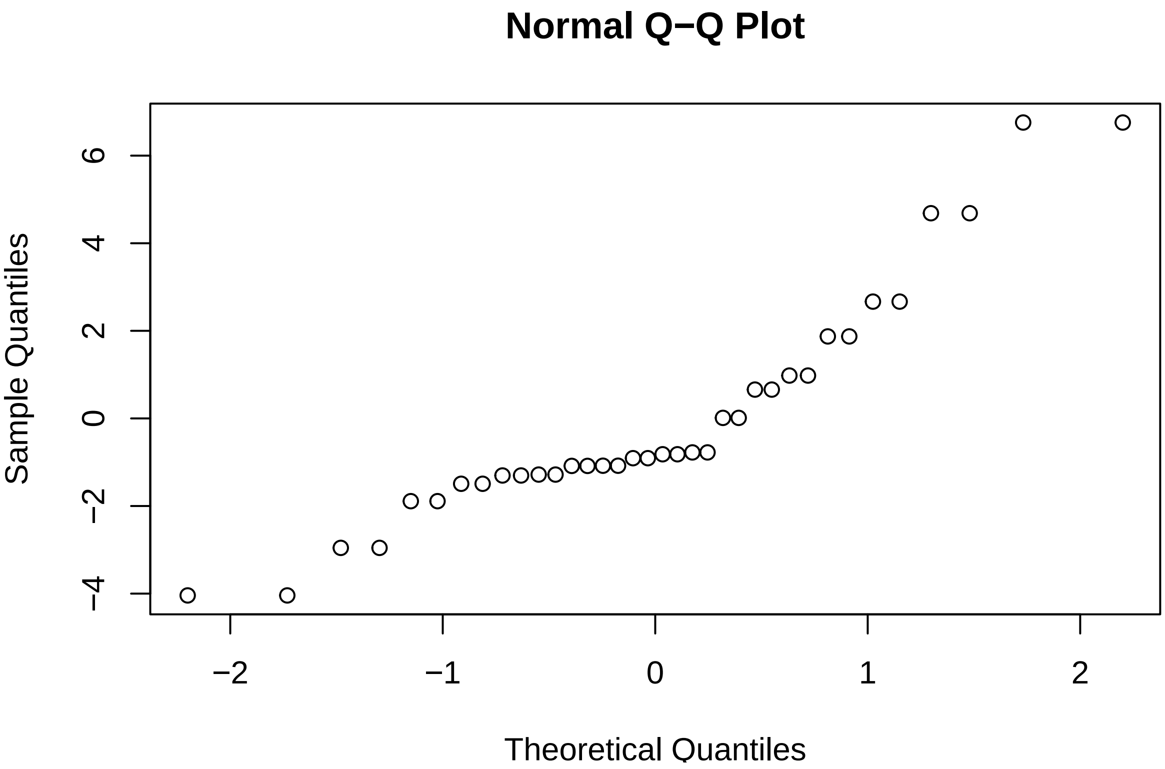


```r
## Changing order to get last comparison
levels(MAOMstock$Type)
```

```
## NULL
```

```r
MAOMstock$Type <- factor(MAOMstock$Type, levels=c('Ungrazed Control', 'Autumn-grazed', 'Spring-grazed'))
MAOMStockModel2 <- lme(MAOM.C.Stock..kgC.m2. ~ Type, random = ~ 1|Block, data = MAOMstock)
```

```
## Error in eval(predvars, data, env): object 'MAOM.C.Stock..kgC.m2.' not found
```

```r
summary(MAOMStockModel2)
```

```
## Error in h(simpleError(msg, call)): error in evaluating the argument 'object' in selecting a method f
```

```r
anova(MAOMStockModel2)
```

```
## Error in anova(MAOMStockModel2): object 'MAOMStockModel2' not found
```

```r
plot(MAOMStockModel2)
```

```
## Error in plot(MAOMStockModel2): object 'MAOMStockModel2' not found
```

```r
qqnorm(MAOMStockModel2$residuals)
```

```
## Error in qqnorm(MAOMStockModel2$residuals): object 'MAOMStockModel2' not found
```

This model exhibits some overdispersion (variance increases with the mean, RvF is fan-shaped).

Square root transformation

```r
MAOMtransformation <- sqrt(MAOMstock$MAOM.C.Stock..kgC.m2.)
```

```
## Error in sqrt(MAOMstock$MAOM.C.Stock..kgC.m2.): non-numeric argument to mathematical function
```

```r
MAOMStockModel3 <- lme(MAOMtransformation ~ Type, random = ~ 1|Block, data = MAOMstock)
```

```
## Error in eval(predvars, data, env): object 'MAOMtransformation' not found
```

```r
summary(MAOMStockModel3)
```

```
## Error in h(simpleError(msg, call)): error in evaluating the argument 'object' in selecting a method f
```

```r
anova(MAOMStockModel3)
```

```
## Error in anova(MAOMStockModel3): object 'MAOMStockModel3' not found
```

```r
plot(MAOMStockModel3)
```

```
## Error in plot(MAOMStockModel3): object 'MAOMStockModel3' not found
```

```r
qqnorm(MAOMStockModel3$residuals)
```

```
## Error in qqnorm(MAOMStockModel3$residuals): object 'MAOMStockModel3' not found
```

Square root transformation doesn't fully fit either but given that the results are clearly NS this doesn't really matter. We can go ahead with the sqrt transformation. This suggests that grazing type does not have any effect on MAOM C stock ($F_{2,14}=0.092$, $p = 0.912$).

Mixed effects model looking at the effect of just Treatment (grazed vs ungrazed)

```r
MAOMStockModel4 <- lme(MAOM.C.Stock..kgC.m2. ~ Treatment, random = ~ 1|Block, data = MAOMstock)
```

```
## Error in eval(predvars, data, env): object 'MAOM.C.Stock..kgC.m2.' not found
```

```r
summary(MAOMStockModel4)
```

```
## Error in h(simpleError(msg, call)): error in evaluating the argument 'object' in selecting a method f
```

```r
anova(MAOMStockModel4)
```

```
## Error in anova(MAOMStockModel4): object 'MAOMStockModel4' not found
```

```r
plot(MAOMStockModel4)
```

```
## Error in plot(MAOMStockModel4): object 'MAOMStockModel4' not found
```

```r
qqnorm(MAOMStockModel4$residuals)
```

```
## Error in qqnorm(MAOMStockModel4$residuals): object 'MAOMStockModel4' not found
```

```r
MAOMtransformation2 <- sqrt(MAOMstock$MAOM.C.Stock..kgC.m2.)
```

```
## Error in sqrt(MAOMstock$MAOM.C.Stock..kgC.m2.): non-numeric argument to mathematical function
```

```r
MAOMStockModel5 <- lme(MAOMtransformation2 ~ Treatment, random = ~ 1|Block, data = MAOMstock)
```

```
## Error in eval(predvars, data, env): object 'MAOMtransformation2' not found
```

```r
summary(MAOMStockModel5)
```

```
## Error in h(simpleError(msg, call)): error in evaluating the argument 'object' in selecting a method f
```

```r
anova(MAOMStockModel5)
```

```
## Error in anova(MAOMStockModel5): object 'MAOMStockModel5' not found
```

```r
plot(MAOMStockModel5)
```

```
## Error in plot(MAOMStockModel5): object 'MAOMStockModel5' not found
```

```r
qqnorm(MAOMStockModel5$residuals)
```

```
## Error in qqnorm(MAOMStockModel5$residuals): object 'MAOMStockModel5' not found
```

Again, using the sqrt transformation even though it isn't perfect. Here, we see that grazing clearly does not have an effect on MAOM C stock ($F1,15 = 0.001$, $p = 0.9797$). Given the results above on grazing type (i.e., that type is not significant either), this suggests we can accurately conclude that grazing (regardless of grazing type) does not influence MAOM C stock.

Part 1 (Section 2) - POM C Stocks Visualization:

```r
POMstock <- completedata2 %>% dplyr::select(Block, ID, Type, Treatment, Study, POM.C.Stock..tons.ha.)
yvar <- POMstock$POM.C.Stock..tons.ha.

POMstockTreatment_means <- POMstock %>% group_by(Treatment) %>% summarise(Treatment_mean_POM.C.Stock..to
POMstockTreatment_means
```

```
## # A tibble: 2 x 2
##   Treatment Treatment_mean_POM.C.Stock..tons.ha.
##   <chr>                                    <dbl>
## 1 Grazed                                    7.32
## 2 Ungrazed                                  7.66
```

```r
POMstockType_means <- POMstock %>% group_by(Type) %>% summarise(Type_mean_POM.C.Stock..tons.ha. = mean(P
POMstockType_means
```

```
## # A tibble: 3 x 2
##   Type             Type_mean_POM.C.Stock..tons.ha.
##   <chr>                                      <dbl>
## 1 Autumn-grazed                               6.74
## 2 Spring-grazed                               7.90
## 3 Ungrazed Control                            7.66
```

```
ggplot(POMstock) +  geom_point(mapping = aes(x=Treatment, y=yvar, shape = Type, col=Type)) + labs(x = "G
```

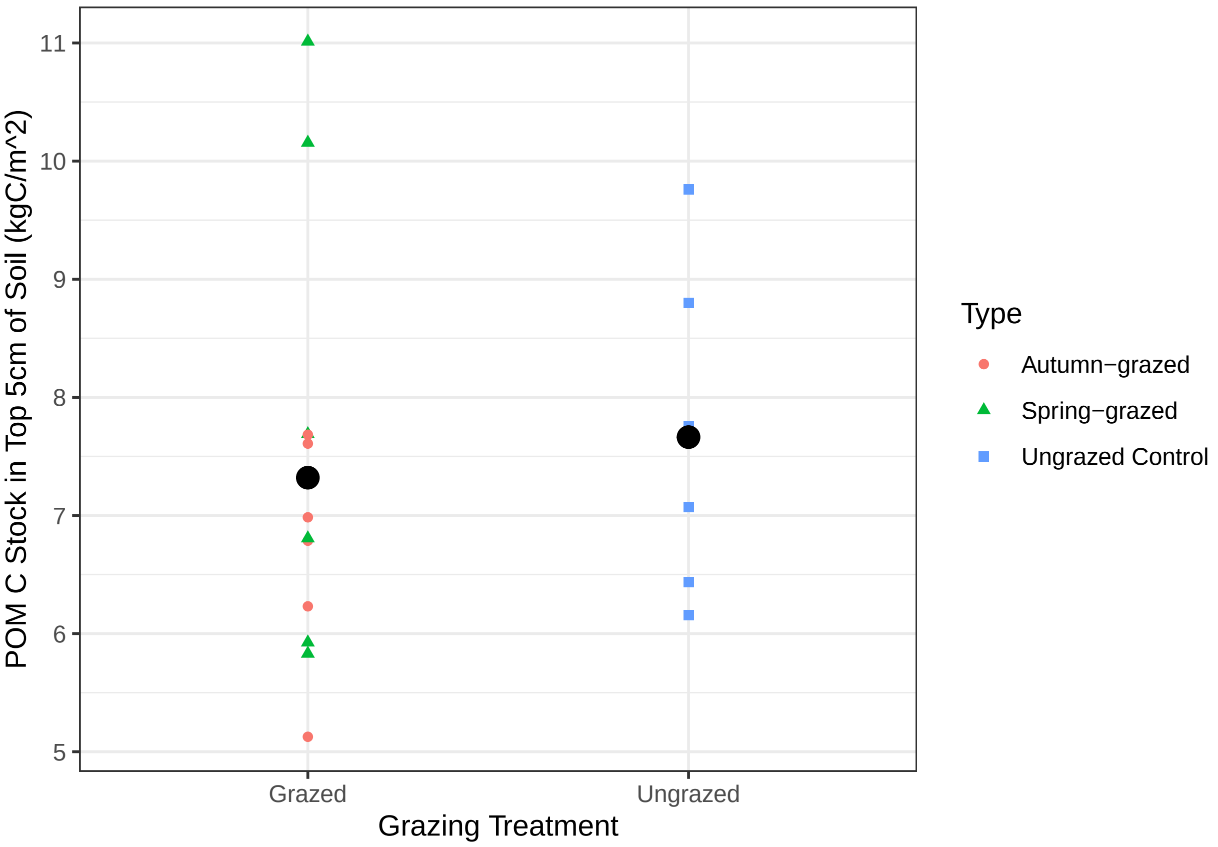


```
ggplot(POMstock, aes(x=Type, y=POM.C.Stock..tons.ha.)) + geom_boxplot(trim=FALSE) + labs(x = "Grazing Ty
```

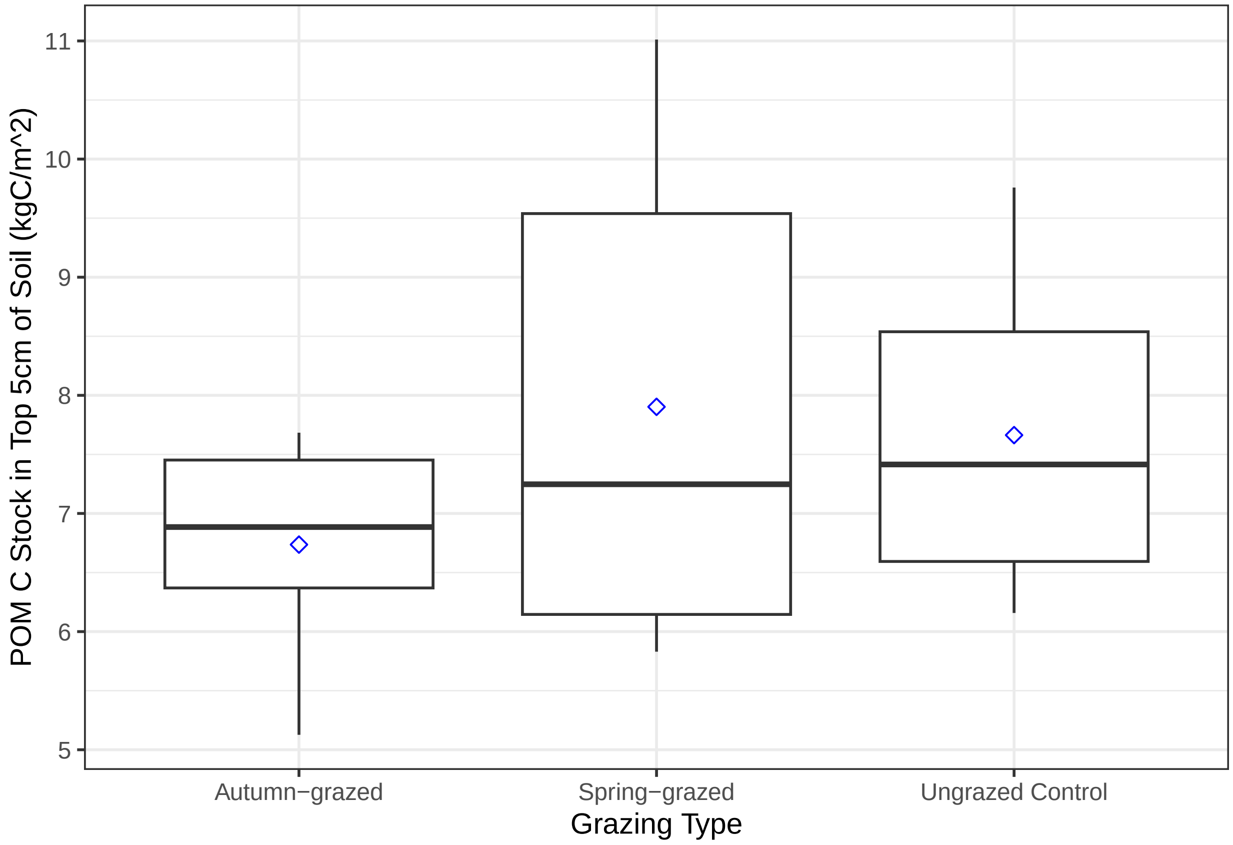


```
# Calculating 95% confidence intervals
UngrazedPOM <- POMstock[c(1,2,3,10,11,12),]
1.96 * sd(UngrazedPOM$POM.C.Stock..tons.ha.)/sqrt(6)
```

```
## [1] 1.121201
```

```
SpringPOM <- POMstock[c(7,8,9,16,17,18),]
1.96 * sd(SpringPOM$POM.C.Stock..tons.ha.)/sqrt(6)
```

```
## [1] 1.760258
```

```
AutumnPOM <- POMstock[c(4,5,6,13,14,15),]
1.96 * sd(AutumnPOM$POM.C.Stock..tons.ha.)/sqrt(6)
```

```
## [1] 0.7649377
```

```
GrazedPOM <- POMstock[c(4,5,6,7,8,9,13,14,15,16,17,18),]
1.96 * sd(GrazedPOM$POM.C.Stock..tons.ha.)/sqrt(12)
```

```
## [1] 0.977721
```

Visually, grazing treatment doesn't seem to have a big effect, though there is an interesting gap between Gibson Autumn and Gibson Spring-grazed types (i.e., grazing type may be important).

Mixed Effects Model looking at the effects of just grazing type (Spring vs Autumn vs Ungrazed Control):

```
POMStockModel1 <- lme(POM.C.Stock..kgC.m2. ~ Type, random = ~ 1|Block, data = POMstock)
```

```
## Error in eval(predvars, data, env): object 'POM.C.Stock..kgC.m2.' not found
```

```r
summary(POMStockModel1)
```

```
## Error in h(simpleError(msg, call)): error in evaluating the argument 'object' in selecting a method f
```

```r
anova(POMStockModel1)
```

```
## Error in anova(POMStockModel1): object 'POMStockModel1' not found
```

```r
plot(POMStockModel1)
```

```
## Error in plot(POMStockModel1): object 'POMStockModel1' not found
```

```r
qqnorm(POMStockModel1$residuals)
```

```
## Error in qqnorm(POMStockModel1$residuals): object 'POMStockModel1' not found
```

```r
## Changing order to get last comparison
levels(POMstock$Type)
```

```
## NULL
```

```r
POMstock$Type <- factor(POMstock$Type, levels=c('Autumn-grazed', 'Spring-grazed', 'Ungrazed Control'))
POMStockModel2 <- lme(POM.C.Stock..kgC.m2. ~ Type, random = ~ 1|Block, data = POMstock)
```

```
## Error in eval(predvars, data, env): object 'POM.C.Stock..kgC.m2.' not found
```

```r
summary(POMStockModel2)
```

```
## Error in h(simpleError(msg, call)): error in evaluating the argument 'object' in selecting a method f
```

```r
anova(POMStockModel2)
```

```
## Error in anova(POMStockModel2): object 'POMStockModel2' not found
```

```r
plot(POMStockModel2)
```

```
## Error in plot(POMStockModel2): object 'POMStockModel2' not found
```

```r
qqnorm(POMStockModel2$residuals)
```

```
## Error in qqnorm(POMStockModel2$residuals): object 'POMStockModel2' not found
```

The analysis suggests that grazing type does not have an effect on POM C stock ($F_{2,14} = 0.88$, $p = 0.435$).

Mixed effects model looking at the effect of just Treatment (grazed vs ungrazed):

```r
POMStockModel3 <- lme(POM.C.Stock..kgC.m2. ~ Treatment, random = ~ 1|Block, data = POMstock)
```

```
## Error in eval(predvars, data, env): object 'POM.C.Stock..kgC.m2.' not found
```

```r
summary(POMStockModel3)
```

```
## Error in h(simpleError(msg, call)): error in evaluating the argument 'object' in selecting a method f
```

```r
anova(POMStockModel3)
```

```
## Error in anova(POMStockModel3): object 'POMStockModel3' not found
```

```r
plot(POMStockModel3)
```

```
## Error in plot(POMStockModel3): object 'POMStockModel3' not found
```

```r
qqnorm(POMStockModel3$residuals)
```

```
## Error in qqnorm(POMStockModel3$residuals): object 'POMStockModel3' not found
```

This model seems to meet the assumptions fine. It suggests that grazing does not influence POM C stock (F1,15 = 0.177, p = 0.679). This seems to be an accurate conclusion given that the further partitioning into grazing type (above) is also non-significant.

Part 1 (Section 3) - Total SOM C Stocks Visualization:

```
Totalstock <- completedata2 %>% dplyr::select(Block, ID, Type, Treatment, Study, Total.SOM.C.Stock..kgC
yvar <- Totalstock$Total.SOM.C.Stock..kgC.m2.

TotalstockTreatment_means <- Totalstock %>% group_by(Treatment) %>% summarise(Treatment_mean_Total.SOM.(
TotalstockTreatment_means
```

```
## # A tibble: 2 x 2
##   Treatment Treatment_mean_Total.SOM.C.Stock..kgC.m2.
##   <chr>                                         <dbl>
## 1 Grazed                                         3.65
## 2 Ungrazed                                       3.68
```

```
TotalstockType_means <- Totalstock %>% group_by(Type) %>% summarise(Type_mean_Total.SOM.C.Stock..kgC.m2
TotalstockType_means
```

```
## # A tibble: 3 x 2
##   Type             Type_mean_Total.SOM.C.Stock..kgC.m2.
##   <chr>                                           <dbl>
## 1 Autumn-grazed                                    3.56
## 2 Spring-grazed                                    3.75
## 3 Ungrazed Control                                 3.68
```

```
ggplot(Totalstock) +  geom_point(mapping = aes(x=Treatment, y=yvar, shape = Type, col=Type)) + labs(x =
```

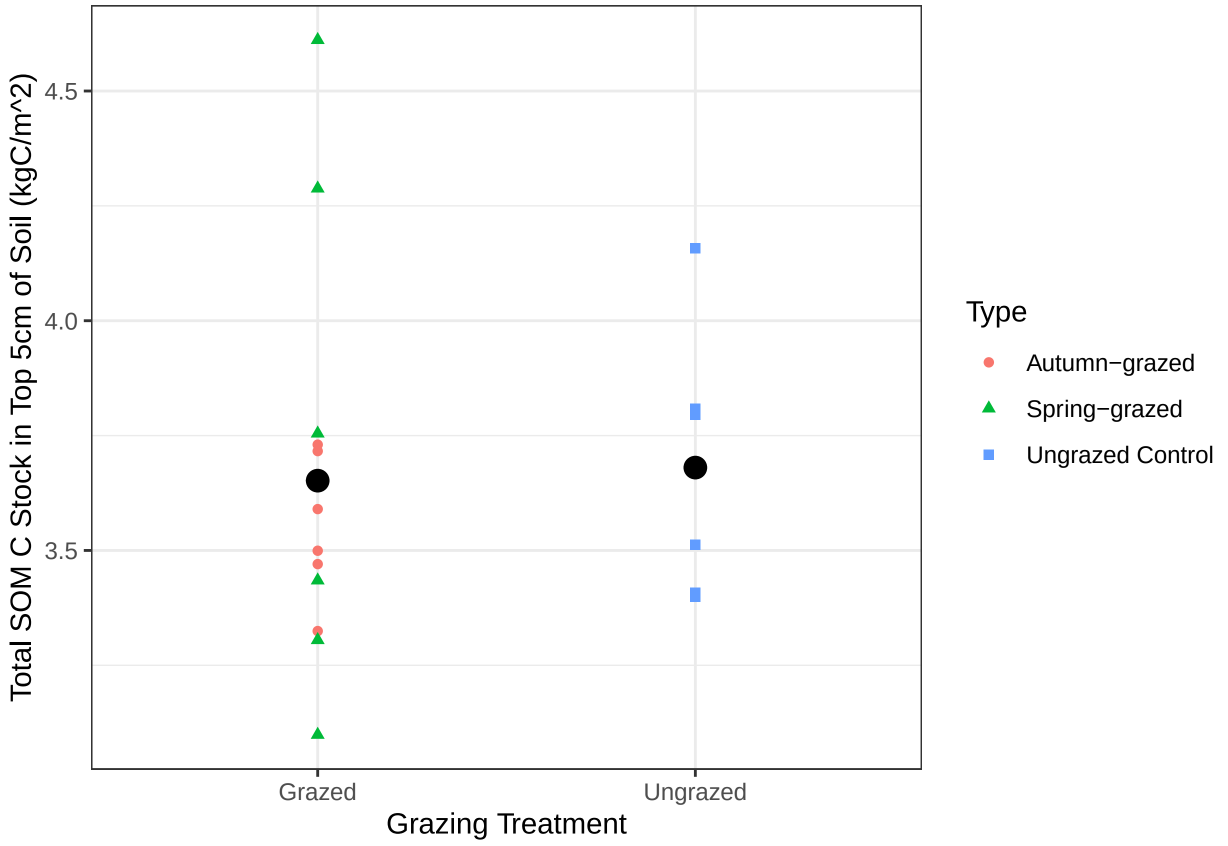

```r
ggplot(Totalstock, aes(x=Type, y=Total.SOM.C.Stock..kgC.m2.)) + geom_boxplot(trim=FALSE) + labs(x = "Gra
```

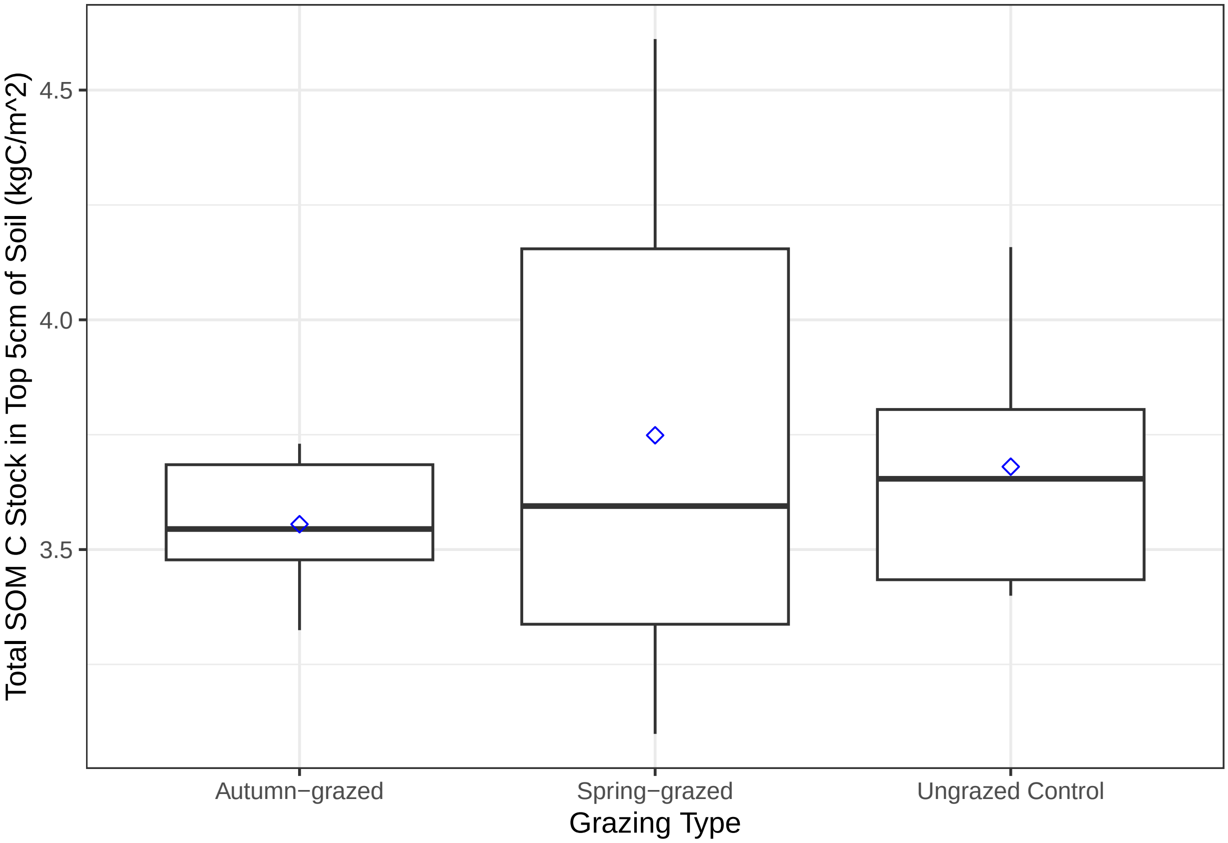


```r
# Calculating 95% confidence intervals
UngrazedTotal <- Totalstock[c(1,2,3,10,11,12),]
1.96 * sd(UngrazedTotal$Total.SOM.C.Stock..kgC.m2.)/sqrt(12)
```

```
## [1] 0.1676258
```

```r
SpringTotal <- Totalstock[c(7,8,9,16,17,18),]
1.96 * sd(SpringTotal$Total.SOM.C.Stock..kgC.m2.)/sqrt(6)
```

```
## [1] 0.4736743
```

```r
AutumnTotal <- Totalstock[c(4,5,6,13,14,15),]
1.96 * sd(AutumnTotal$Total.SOM.C.Stock..kgC.m2.)/sqrt(6)
```

```
## [1] 0.1246302
```

```r
GrazedTotal <- Totalstock[c(4,5,6,7,8,9,13,14,15,16,17,18),]
1.96 * sd(GrazedTotal$Total.SOM.C.Stock..kgC.m2.)/sqrt(12)
```

```
## [1] 0.2404024
```

Visually, this suggests that grazing treatment has no real impact, but grazing type might (spring-grazed has higher mean). Also important to note the variability is much higher in spring-grazed than in either autumn-grazed or ungrazed control.

Mixed Effects Model looking at the effects of just grazing type (Spring vs Autumn vs Ungrazed Control):

```r
TotalStockModel1 <- lme(Total.SOM.C.Stock..kgC.m2. ~ Type, random = ~ 1|Block, data = Totalstock)
summary(TotalStockModel1)
```

```
## Linear mixed-effects model fit by REML
##   Data: Totalstock
##        AIC      BIC    logLik
##   29.89627 33.43653 -9.948137
##
## Random effects:
##  Formula: ~1 | Block
##         (Intercept) Residual
## StdDev: 6.222304e-06 0.392623
##
## Fixed effects:  Total.SOM.C.Stock..kgC.m2. ~ Type
##                          Value Std.Error DF   t-value
## (Intercept)           3.555128 0.1602877 14 22.179672
## TypeSpring-grazed     0.193545 0.2266810 14  0.853821
## TypeUngrazed Control 0.125200 0.2266810 14  0.552319
##                      p-value
## (Intercept)           0.0000
## TypeSpring-grazed     0.4076
## TypeUngrazed Control  0.5894
##  Correlation:
##                      (Intr) TypSp-
## TypeSpring-grazed    -0.707
## TypeUngrazed Control -0.707  0.500
##
## Standardized Within-Group Residuals:
##         Min          Q1         Med          Q3
## -1.65518266 -0.66664698 -0.06354148  0.38908408
##         Max
##  2.19652140
##
## Number of Observations: 18
## Number of Groups: 2
```

```r
anova(TotalStockModel1)
```

```
##             numDF denDF  F-value p-value
## (Intercept)     1    14 1565.344  <.0001
## Type            2    14    0.375   0.694
```

```r
plot(TotalStockModel1)
```

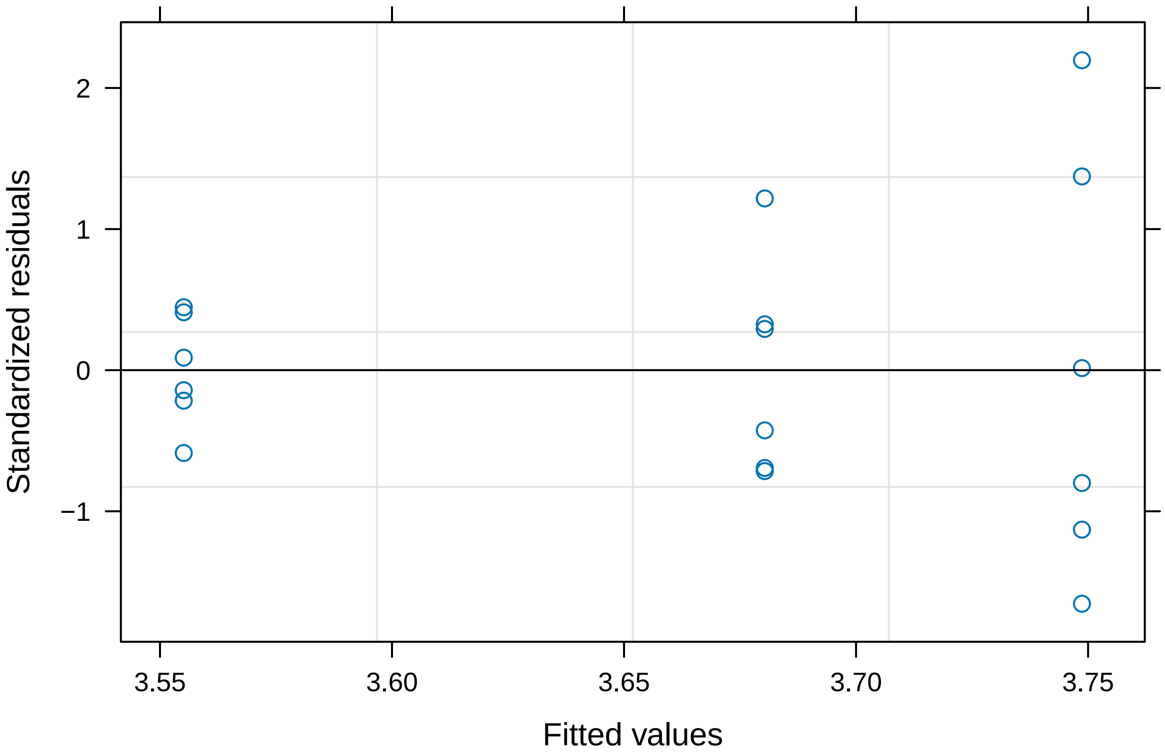


```r
qqnorm(TotalStockModel1$residuals)
```

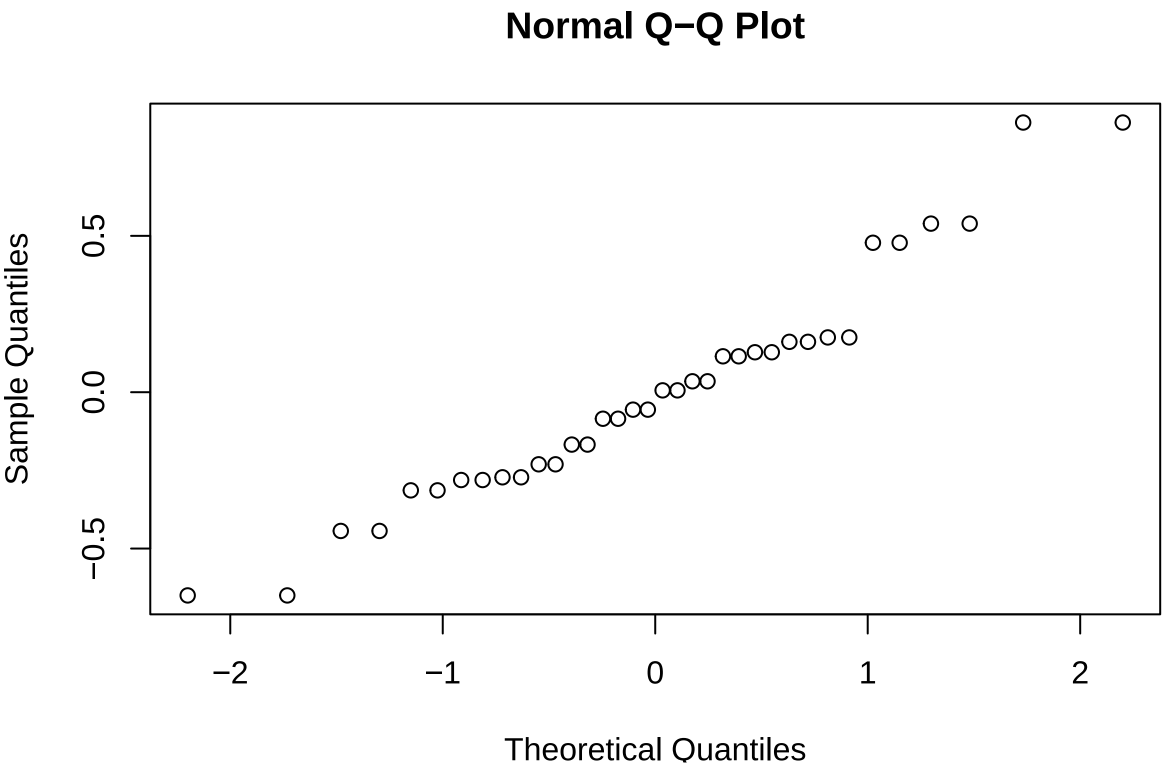


```r
# Transformation
TotalStockModel1A <- lme(sqrt(Total.SOM.C.Stock..kgC.m2.) ~ Type, random = ~ 1|Block, data = Totalstock)
summary(TotalStockModel1A)
```

```
## Linear mixed-effects model fit by REML
##   Data: Totalstock
##         AIC       BIC   logLik
##   -10.91953 -7.379278 10.45976
##
## Random effects:
##  Formula: ~1 | Block
##          (Intercept)  Residual
## StdDev: 1.625488e-06 0.1007179
##
## Fixed effects:  sqrt(Total.SOM.C.Stock..kgC.m2.) ~ Type
##                          Value  Std.Error DF  t-value
## (Intercept)          1.8851259 0.04111790 14 45.84685
## TypeSpring-grazed    0.0460974 0.05814948 14  0.79274
## TypeUngrazed Control 0.0320195 0.05814948 14  0.55064
##                      p-value
## (Intercept)           0.0000
## TypeSpring-grazed     0.4412
## TypeUngrazed Control  0.5906
##  Correlation:
##                      (Intr) TypSp-
## TypeSpring-grazed    -0.707
## TypeUngrazed Control -0.707  0.500
##
## Standardized Within-Group Residuals:
##         Min          Q1         Med          Q3
## -1.69661494 -0.68218805 -0.03984604  0.40252011
##         Max
##  2.14578469
##
## Number of Observations: 18
## Number of Groups: 2
```

```
anova(TotalStockModel1A)
```

```
##             numDF denDF  F-value p-value
## (Intercept)     1    14 6481.206  <.0001
## Type            2    14    0.330  0.7243
```

```
plot(TotalStockModel1A)
```

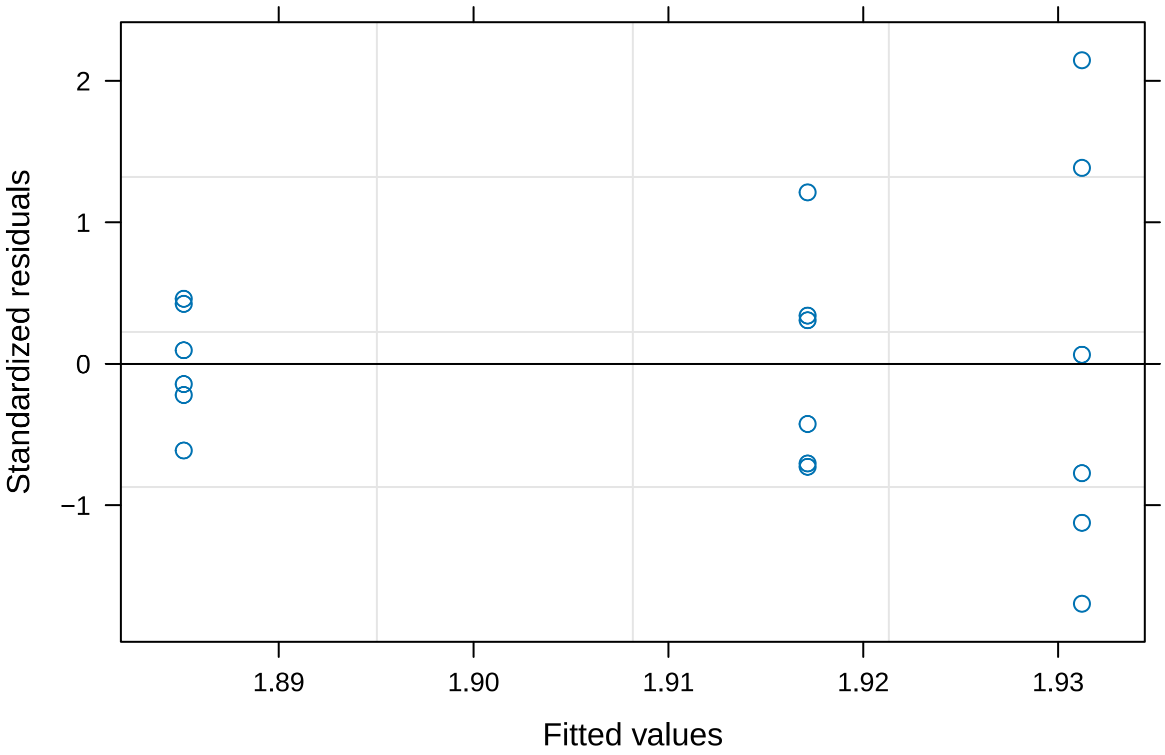


```
qqnorm(TotalStockModel1A$residuals)
```

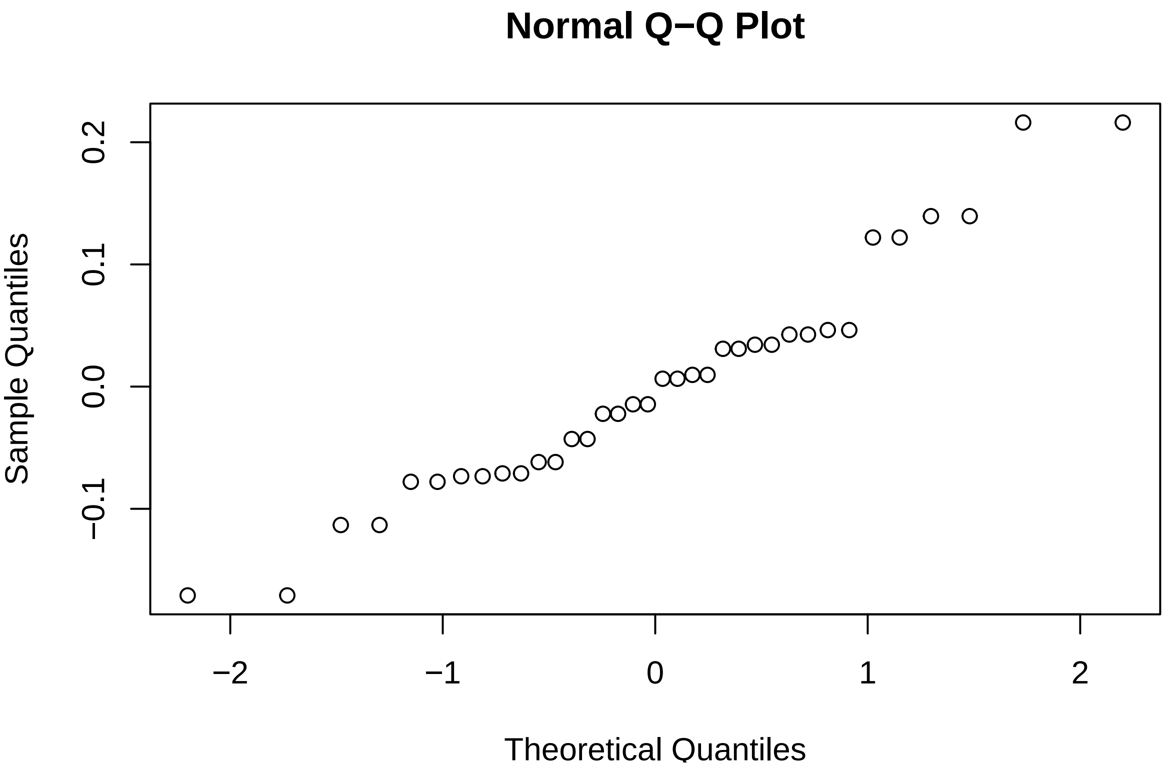

This model suggests that grazing type does not influence the total C stock ($F2,14 = 0.330$, $p = 0.724$).

Mixed effects model looking at the effect of just Treatment (grazed vs ungrazed):

```r
TotalStockModel3 <- lme(Total.SOM.C.Stock..kgC.m2. ~ Treatment, random = ~ 1|Block, data = Totalstock)
summary(TotalStockModel3)
```

```
## Linear mixed-effects model fit by REML
##   Data: Totalstock
##        AIC      BIC    logLik
##   27.49242 30.58277 -9.746209
##
## Random effects:
##  Formula: ~1 | Block
##         (Intercept)  Residual
## StdDev: 6.169855e-06 0.3892839
##
## Fixed effects:  Total.SOM.C.Stock..kgC.m2. ~ Treatment
##                        Value Std.Error DF  t-value p-value
## (Intercept)       3.651901 0.1123766 15 32.49699  0.0000
## TreatmentUngrazed 0.028428 0.1946420 15  0.14605  0.8858
##  Correlation:
##                   (Intr)
## TreatmentUngrazed -0.577
##
## Standardized Within-Group Residuals:
##        Min         Q1        Med         Q3        Max
## -1.4207889 -0.6637360 -0.2753967  0.2870315  2.4639532
##
## Number of Observations: 18
## Number of Groups: 2
```

```r
anova(TotalStockModel3)
```

```
##             numDF denDF   F-value p-value
## (Intercept)     1    15 1592.3127  <.0001
## Treatment       1    15    0.0213  0.8858
```

```r
plot(TotalStockModel3)
```

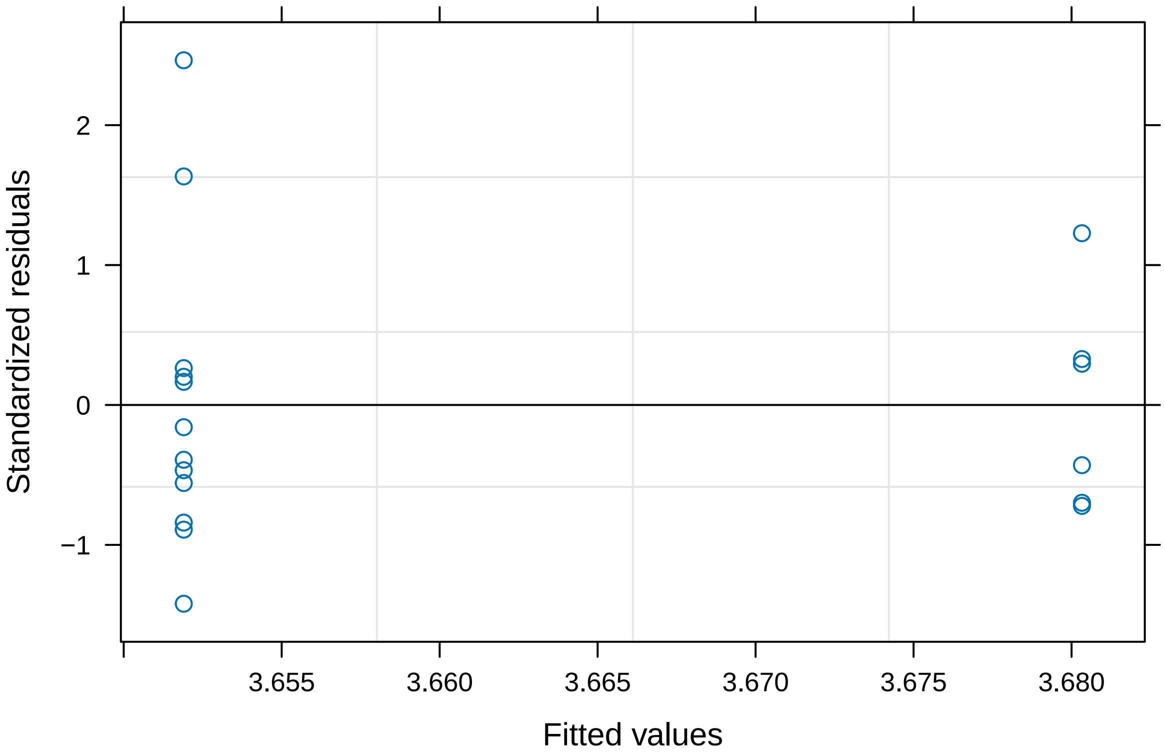


```r
qqnorm(TotalStockModel3$residuals)
```

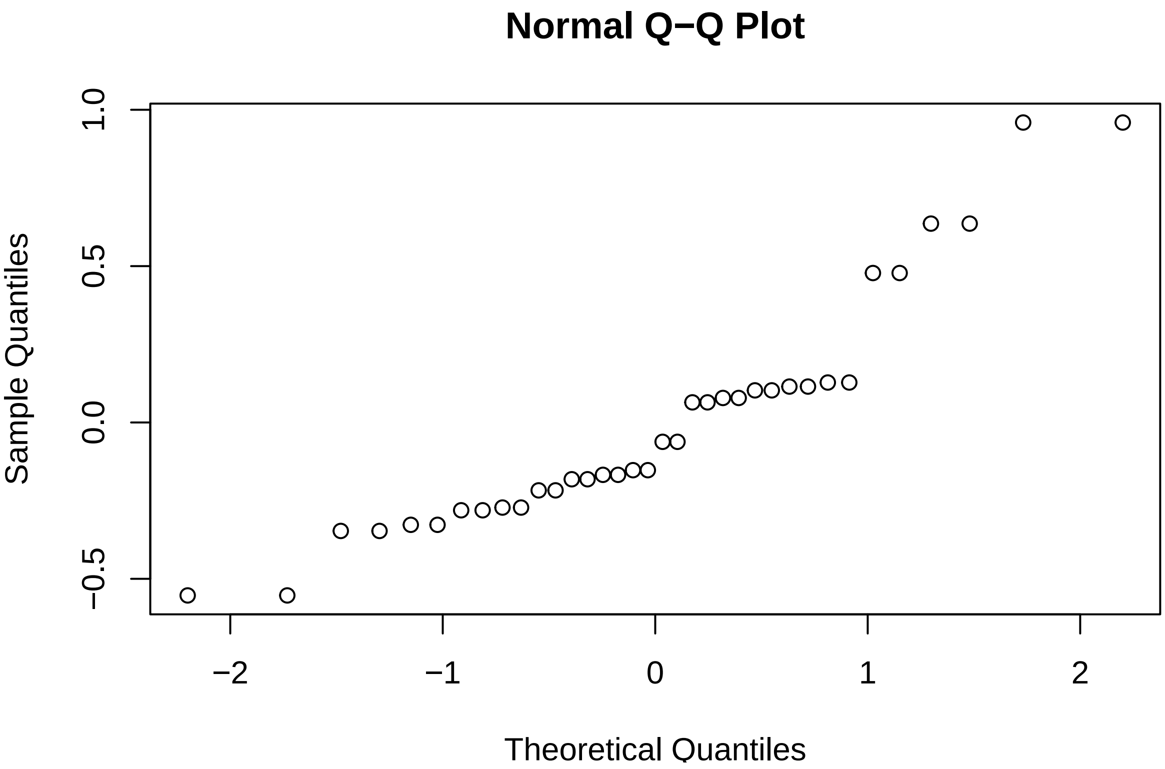

Slightly underdispersed but acceptable. Grazing does not affect total C stocks in the SOM (F1,15 = 0.021, p = 0.886).

C Stocks Bar chart:

```r
Cbardata <- completedata %>% dplyr::select(Survey, Fraction, C.stocks)
Cbardata <- Cbardata[c(1,2,3,4,5,6,7,8,9,10,11,12),]
ggplot(Cbardata, aes(fill=Fraction, y=C.stocks, x=Survey)) +
  geom_bar(position='stack', stat='identity')
```

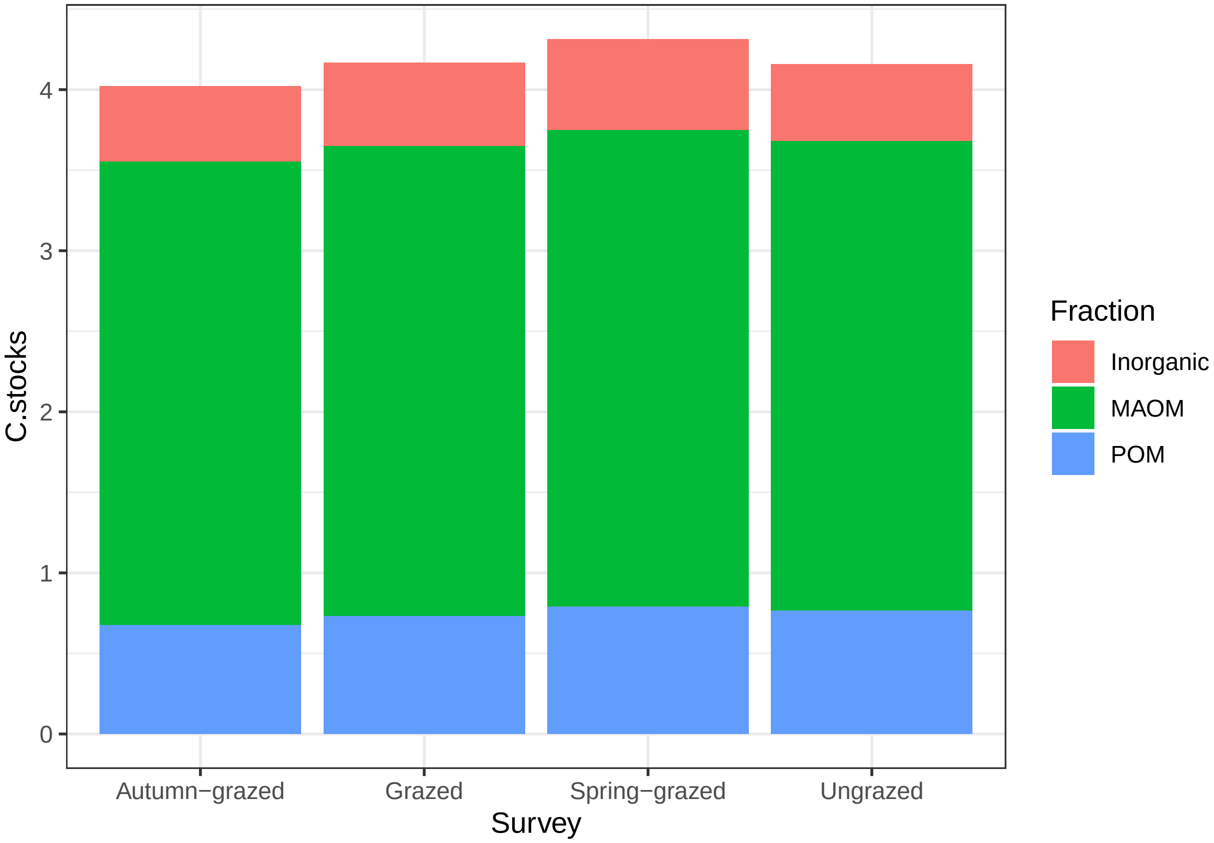


```r
levels(Cbardata$Survey)
```

```
## NULL
```

```r
Cbardata$Survey <- factor (Cbardata$Survey, levels=c('Ungrazed', 'Grazed','Spring-grazed','Autumn-graze
ggplot(Cbardata, aes(fill=Fraction, y=C.stocks, x=Survey)) +
  geom_bar(position='stack', stat='identity') + labs(x = "Grazing Treatment", y = "Carbon Stock in Top 
```

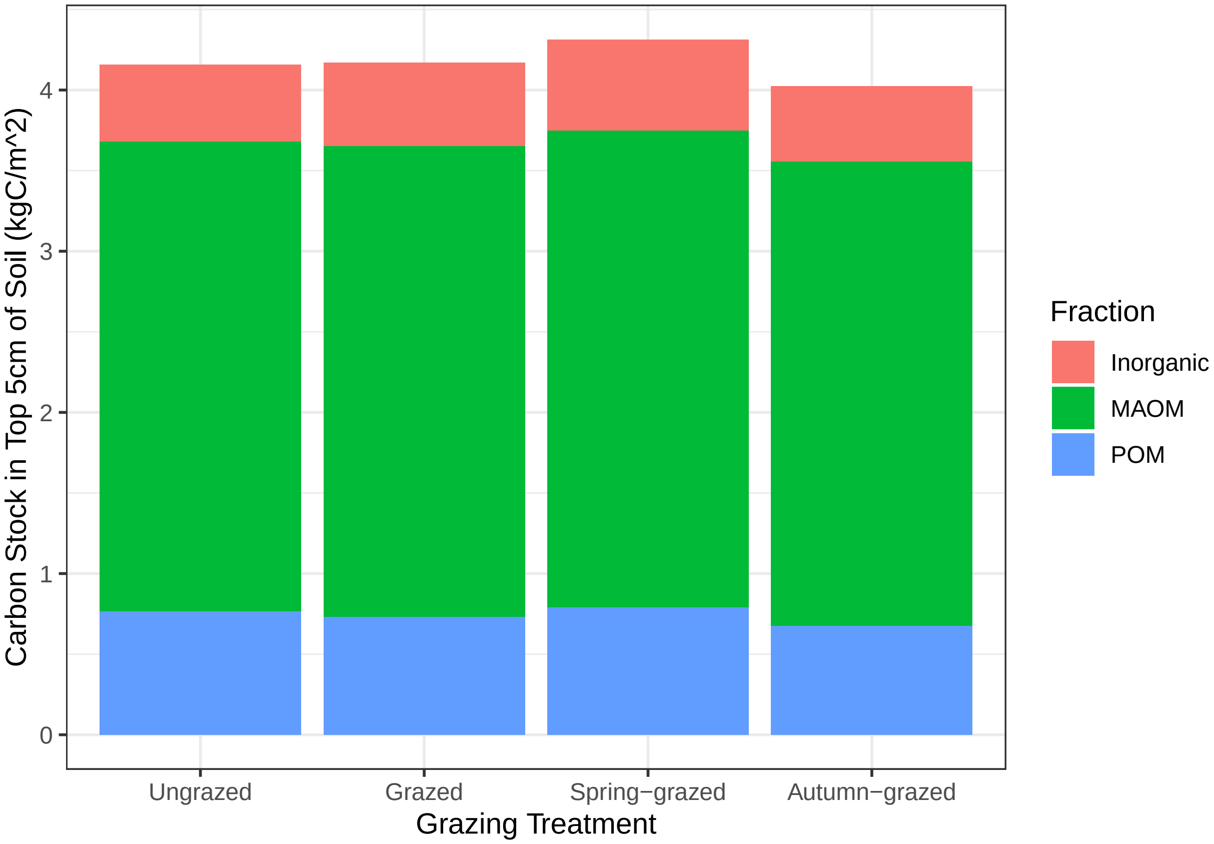


```
Cbardata4 <-Cbardata[c(3,4,5,6,7,8),]
ggplot(Cbardata4, aes(fill=Fraction, y=C.stocks, x=Survey)) +
  geom_bar(position='stack', stat='identity') + labs(x = "Grazing Type", y = "Carbon Stock in Top 5cm of
```

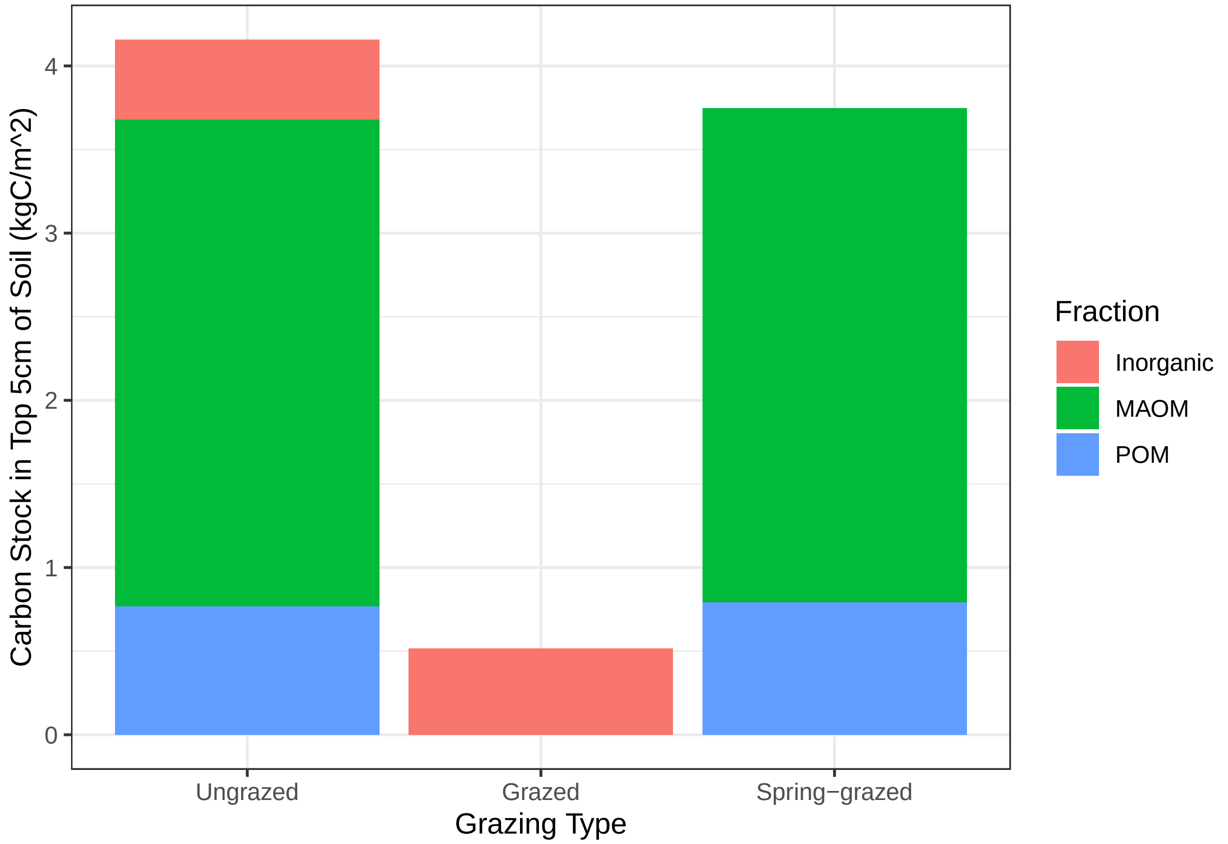


```
Cbardata2 <-Cbardata[c(1,2,3,4),]
Cbardata3 <-Cbardata[c(5,6,7,8),]
ggplot(Cbardata2, aes(fill=Fraction, y=C.stocks, x=Survey)) +
  geom_bar(position='stack', stat='identity') + ylim(0,4) + labs(x = "Grazing Treatment", y = "Carbon S
```

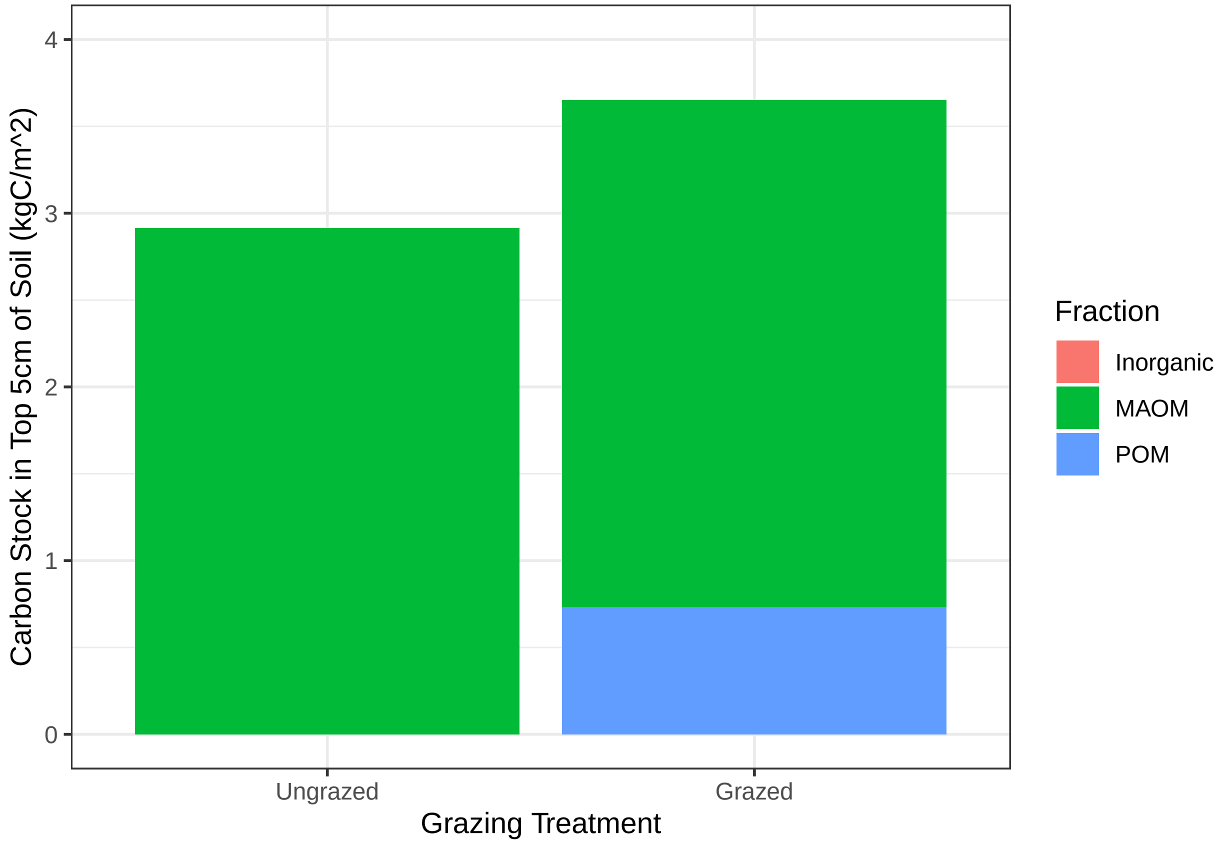


```
ggplot(Cbardata3, aes(fill=Fraction, y=C.stocks, x=Survey)) +
  geom_bar(position='stack', stat='identity') + ylim(0,4) + labs(x = "Grazing Type", y = "Carbon Stock i
```

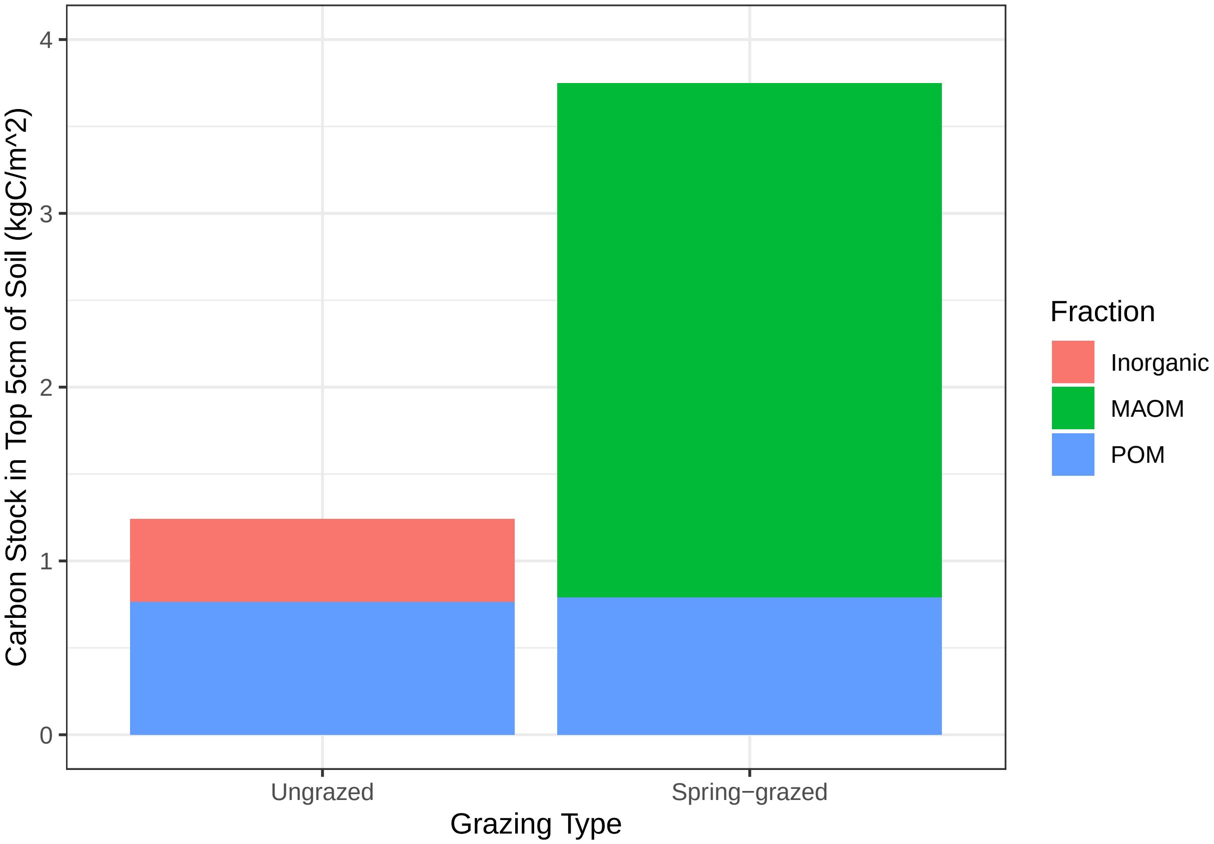


```
levels(Cbardata2$Survey)
```

```
## [1] "Ungrazed"      "Grazed"        "Spring-grazed"
## [4] "Autumn-grazed"
```

Part 2 - Nitrogen Stocks Part 2 (Section 1) - MAOM N Stocks Visualization:

```
MAOMNstock <- completedata2 %>% dplyr::select(Block, ID, Type, Treatment, Study, MAOM.N.Stock..kgN.m2.)
yvar <- MAOMNstock$MAOM.N.Stock..kgN.m2.

MAOMNstockTreatment_means <- MAOMNstock %>% group_by(Treatment) %>% summarise(Treatment_mean_MAOM.N.Sto
MAOMNstockTreatment_means
```

```
## # A tibble: 2 x 2
##   Treatment Treatment_mean_MAOM.N.Stock..kgN.m2.
##   <chr>                                    <dbl>
## 1 Grazed                                   0.242
## 2 Ungrazed                                 0.253
```

```
MAOMNstockType_means <- MAOMNstock %>% group_by(Type) %>% summarise(Type_mean_MAOM.N.Stock..kgN.m2. = me
MAOMNstockType_means
```

```
## # A tibble: 3 x 2
##   Type             Type_mean_MAOM.N.Stock..kgN.m2.
##   <chr>                                      <dbl>
## 1 Autumn-grazed                              0.245
## 2 Spring-grazed                              0.239
## 3 Ungrazed Control                           0.253
```

```r
ggplot(MAOMNstock) +  geom_point(mapping = aes(x=Treatment, y=yvar, shape = Type, col=Type)) + labs(x =
```

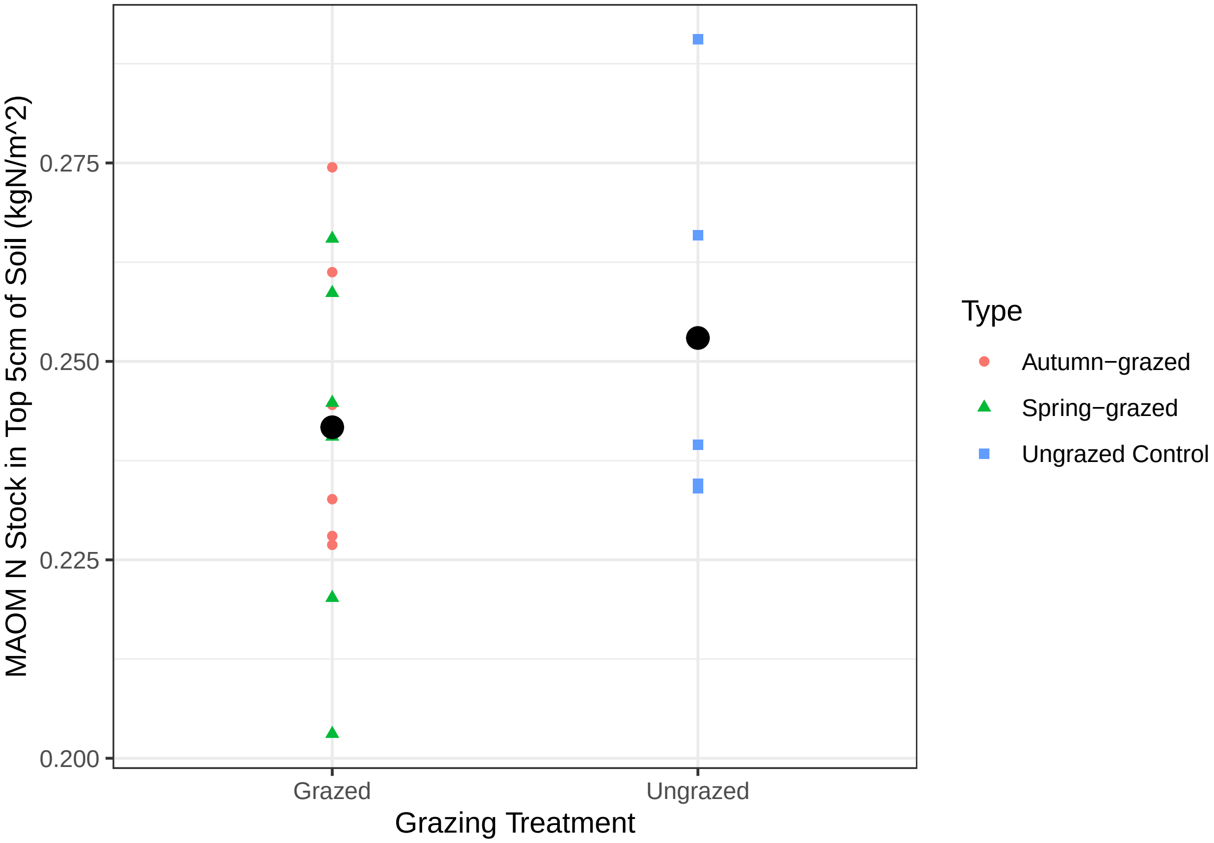


```r
ggplot(MAOMNstock, aes(x=Type, y=MAOM.N.Stock..kgN.m2.)) + geom_boxplot(trim=FALSE) + labs(x = "Grazing
```

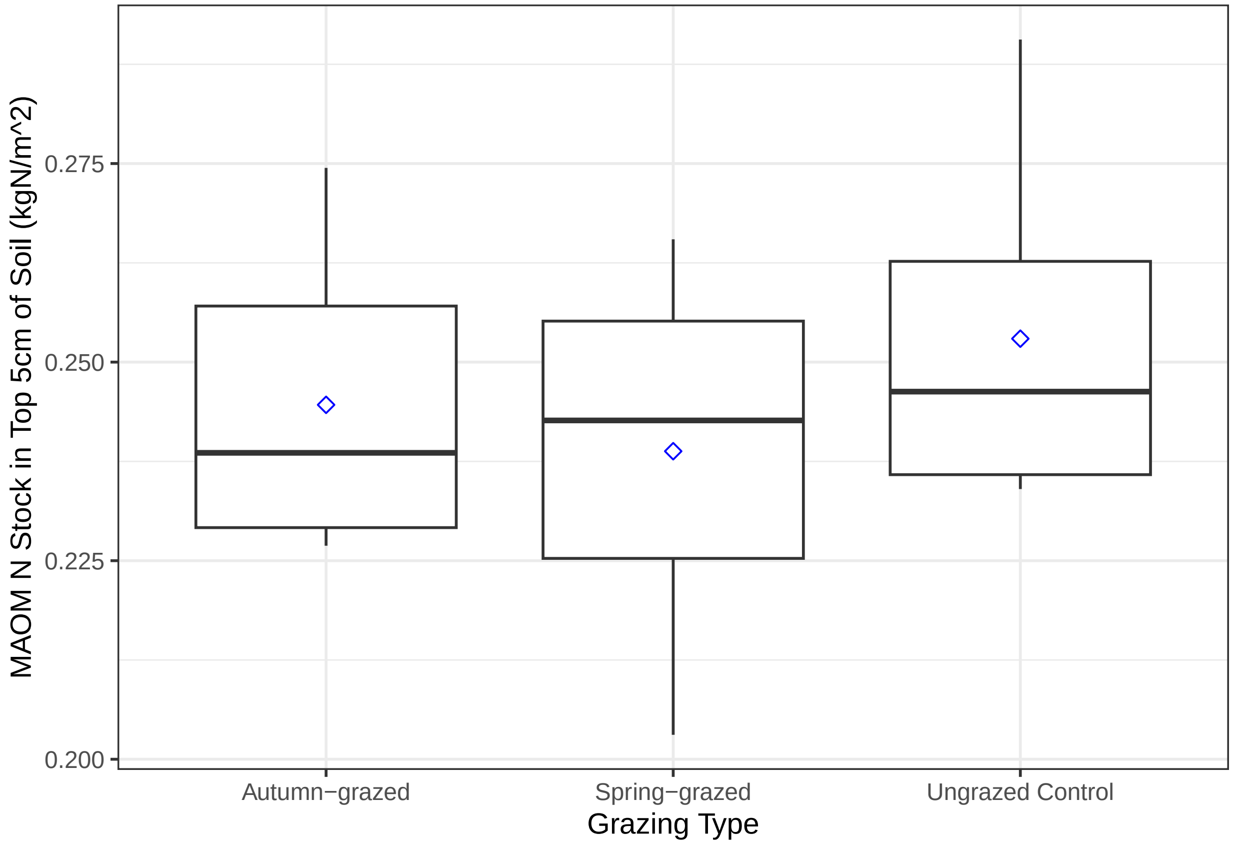


```
# Calculating 95% confidence intervals
NUngrazedMAOM <- MAOMNstock[c(1,2,3,10,11,12),]
1.96 * sd(NUngrazedMAOM$MAOM.N.Stock..kgN.m2.)/sqrt(6)
```

```
## [1] 0.01775557
```

```
NSpringMAOM <- MAOMNstock[c(7,8,9,16,17,18),]
1.96 * sd(NSpringMAOM$MAOM.N.Stock..kgN.m2.)/sqrt(6)
```

```
## [1] 0.01881579
```

```
NAutumnMAOM <- MAOMNstock[c(4,5,6,13,14,15),]
1.96 * sd(NAutumnMAOM$MAOM.N.Stock..kgN.m2.)/sqrt(6)
```

```
## [1] 0.01560054
```

```
NGrazedMAOM <- MAOMNstock[c(4,5,6,7,8,9,13,14,15,16,17,18),]
1.96 * sd(NGrazedMAOM$MAOM.N.Stock..kgN.m2.)/sqrt(6)
```

```
## [1] 0.01665866
```

Visual conclusions: Mean MAOM N stock is higher in the ungrazed treatment, but this may not be significant. It doesn't look like grazing type has much of an effect.

Mixed Effects Model looking at the effects of just grazing type (Spring vs Autumn vs Ungrazed Control):

```
MAOMNstockModel1 <- lme(MAOM.N.Stock..kgN.m2. ~ Type, random = ~ 1|Block, data = MAOMNstock)
summary(MAOMNstockModel1)
```

```
## Linear mixed-effects model fit by REML
##   Data: MAOMNstock
```

```
##       AIC       BIC  logLik
##   -57.641 -54.10074 33.8205
##
## Random effects:
##  Formula: ~1 | Block
##         (Intercept)   Residual
## StdDev: 0.009757579 0.02044567
##
## Fixed effects:  MAOM.N.Stock..kgN.m2. ~ Type
##                             Value  Std.Error DF   t-value
## (Intercept)           0.24462330 0.01082941 14 22.588798
## TypeSpring-grazed    -0.00584258 0.01180431 14 -0.494953
## TypeUngrazed Control  0.00832751 0.01180431 14  0.705464
##                      p-value
## (Intercept)           0.0000
## TypeSpring-grazed     0.6283
## TypeUngrazed Control  0.4921
##  Correlation:
##                      (Intr) TypSp-
## TypeSpring-grazed    -0.545
## TypeUngrazed Control -0.545  0.500
##
## Standardized Within-Group Residuals:
##        Min         Q1        Med         Q3        Max
## -1.4696801 -0.6132079 -0.2764097  0.6109427  1.5817617
##
## Number of Observations: 18
## Number of Groups: 2
```

```
anova(MAOMNstockModel1)
```

```
##             numDF denDF  F-value p-value
## (Intercept)     1    14 850.5931  <.0001
## Type            2    14   0.7279  0.5003
```

```
plot(MAOMNstockModel1)
```

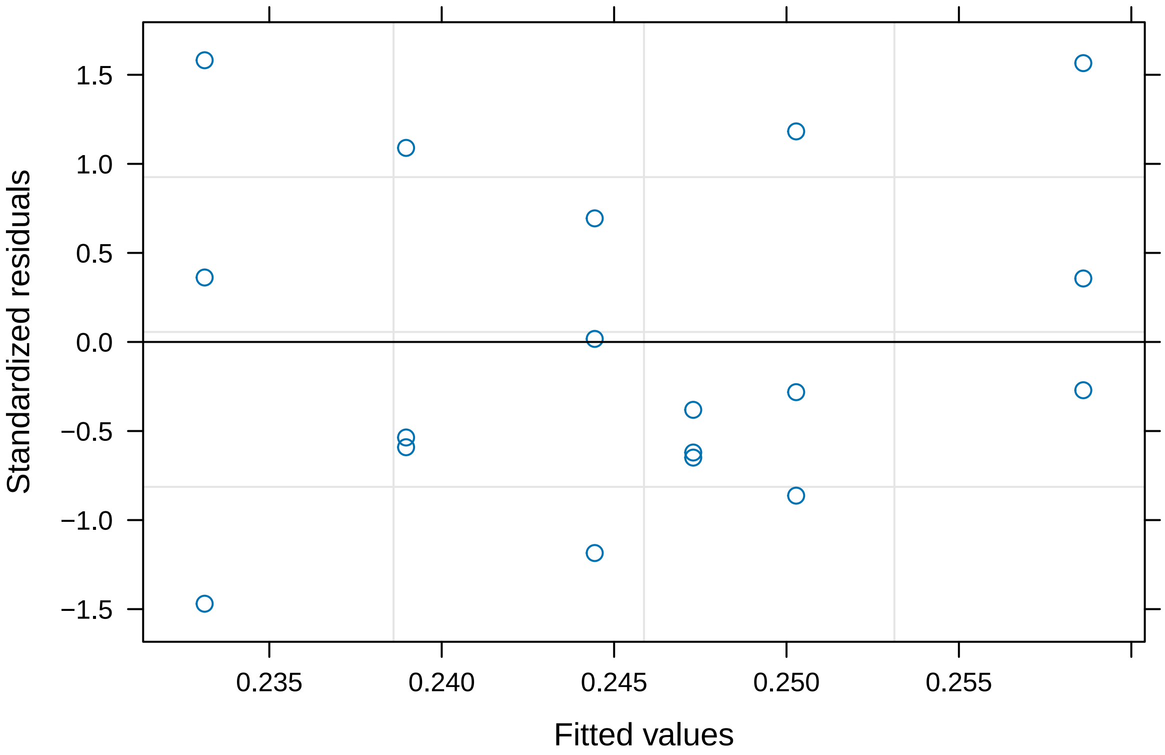


```r
qqnorm(MAOMNstockModel1$residuals)
```

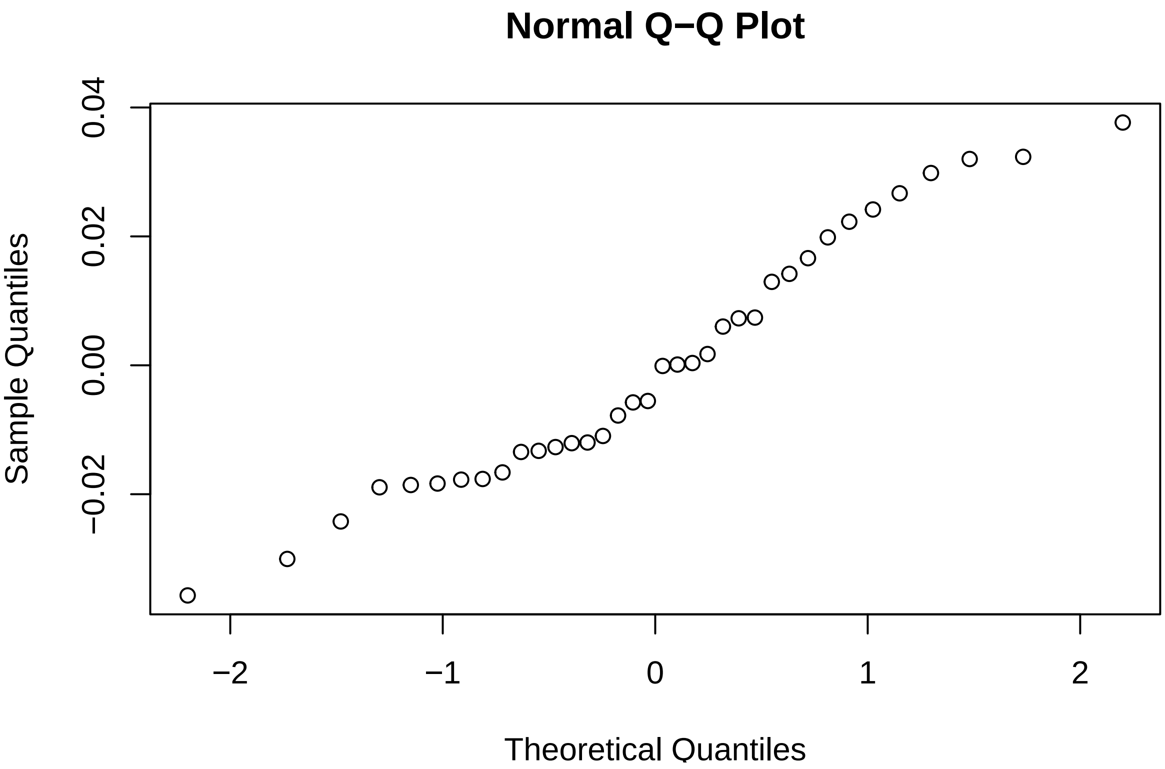


```r
## Changing order to get last comparison
levels(MAOMNstock$Type)
```

```
## NULL
```

```r
MAOMNstock$Type <- factor(MAOMNstock$Type, levels=c('Autumn-grazed', 'Spring-grazed', 'Ungrazed Control
MAOMNstockModel2 <- lme(MAOM.N.Stock..kgN.m2. ~ Type, random = ~ 1|Block, data = MAOMNstock)
summary(MAOMNstockModel2)
```

```
## Linear mixed-effects model fit by REML
##   Data: MAOMNstock
##       AIC       BIC  logLik
##   -57.641 -54.10074 33.8205
##
## Random effects:
##  Formula: ~1 | Block
##         (Intercept)   Residual
## StdDev: 0.009757579 0.02044567
##
## Fixed effects:  MAOM.N.Stock..kgN.m2. ~ Type
##                              Value  Std.Error DF   t-value
## (Intercept)             0.24462330 0.01082941 14 22.588798
## TypeSpring-grazed      -0.00584258 0.01180431 14 -0.494953
## TypeUngrazed Control    0.00832751 0.01180431 14  0.705464
##                        p-value
## (Intercept)             0.0000
## TypeSpring-grazed       0.6283
## TypeUngrazed Control    0.4921
##  Correlation:
##                        (Intr) TypSp-
## TypeSpring-grazed      -0.545
## TypeUngrazed Control -0.545  0.500
##
## Standardized Within-Group Residuals:
##        Min         Q1        Med         Q3        Max
## -1.4696801 -0.6132079 -0.2764097  0.6109427  1.5817617
##
## Number of Observations: 18
## Number of Groups: 2
```

```r
anova(MAOMNstockModel2)
```

```
##             numDF denDF  F-value p-value
## (Intercept)     1    14 850.5931  <.0001
## Type            2    14   0.7279  0.5003
```

```r
plot(MAOMNstockModel2)
```

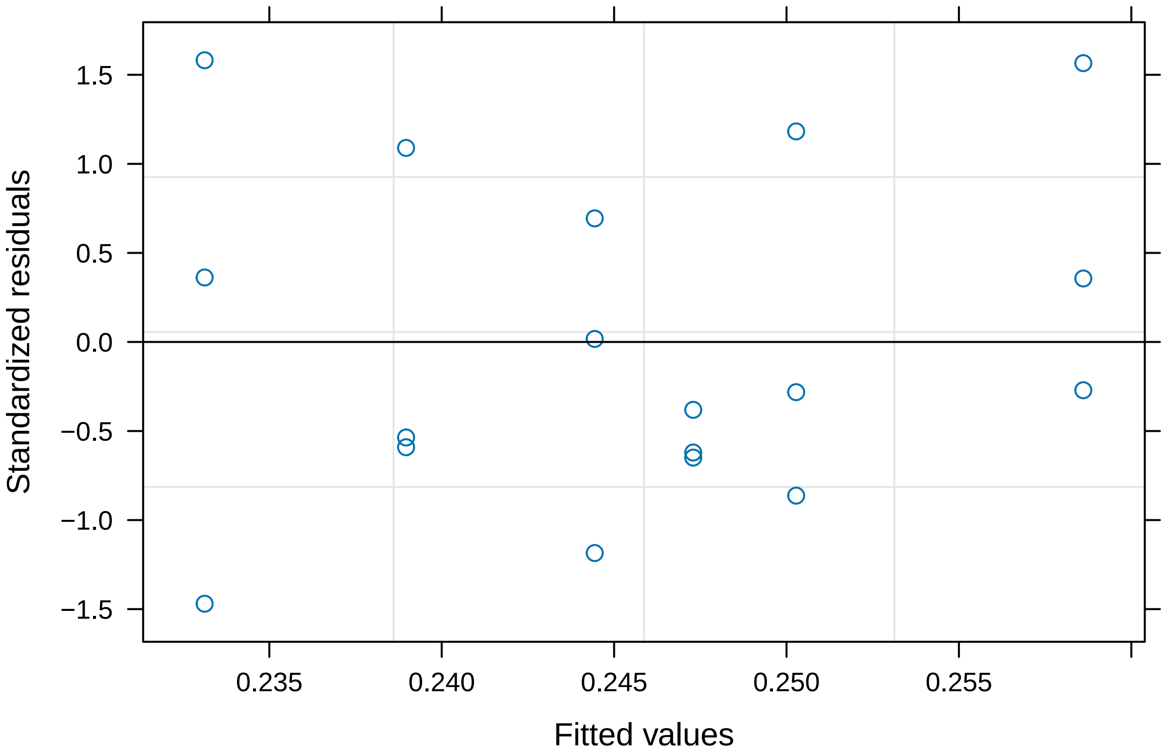


```r
qqnorm(MAOMNstockModel2$residuals)
```

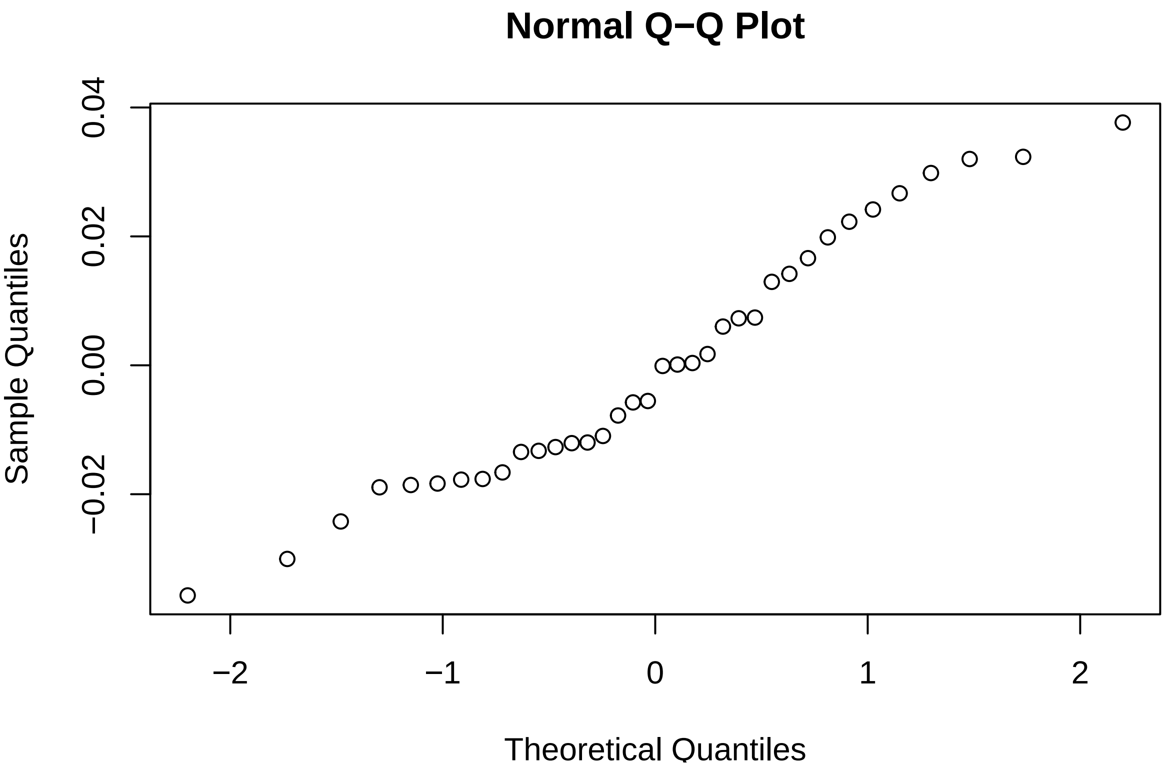

Meets assumptions fine. This model suggests that grazing type does not influence the MAOM N stock (F2,14 = 0.728, p = 0.500).

Mixed effects model looking at the effect of just Treatment (grazed vs ungrazed):

```r
MAOMNstockModel3 <- lme(MAOM.N.Stock..kgN.m2. ~ Treatment, random = ~ 1|Block, data = MAOMNstock)
summary(MAOMNstockModel3)
```

```
## Linear mixed-effects model fit by REML
##   Data: MAOMNstock
##         AIC       BIC   logLik
##   -66.45639 -63.36603 37.22819
##
## Random effects:
##  Formula: ~1 | Block
##         (Intercept)   Residual
## StdDev: 0.009876652 0.01992446
##
## Fixed effects:  MAOM.N.Stock..kgN.m2. ~ Treatment
##                         Value   Std.Error DF   t-value
## (Intercept)       0.24170200 0.009047438 15 26.714965
## TreatmentUngrazed 0.01124881 0.009962231 15  1.129145
##                   p-value
## (Intercept)        0.0000
## TreatmentUngrazed  0.2766
##  Correlation:
##                   (Intr)
## TreatmentUngrazed -0.367
##
## Standardized Within-Group Residuals:
##        Min         Q1        Med         Q3        Max
## -1.6477729 -0.5856231 -0.2173023  0.5085698  1.5994888
##
## Number of Observations: 18
## Number of Groups: 2
```

```r
anova(MAOMNstockModel3)
```

```
##             numDF denDF  F-value p-value
## (Intercept)     1    15 850.5931  <.0001
## Treatment       1    15   1.2750  0.2766
```

```r
plot(MAOMNstockModel3)
```

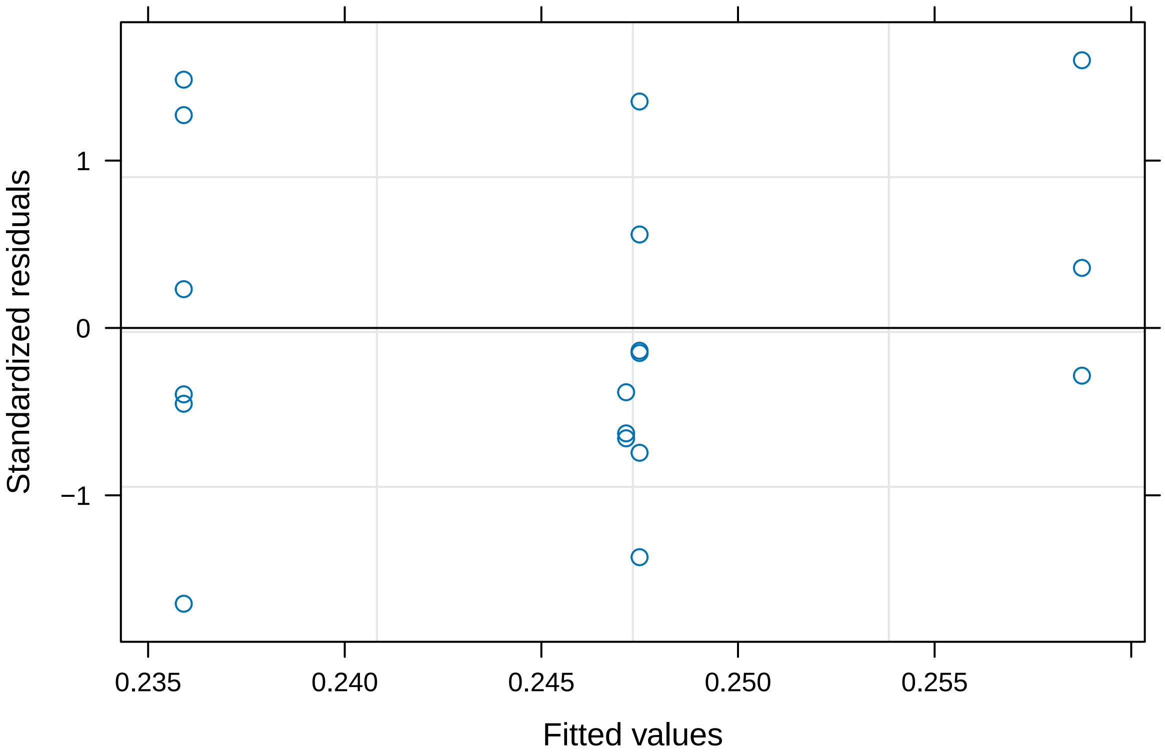


```r
qqnorm(MAOMNstockModel3$residuals)
```

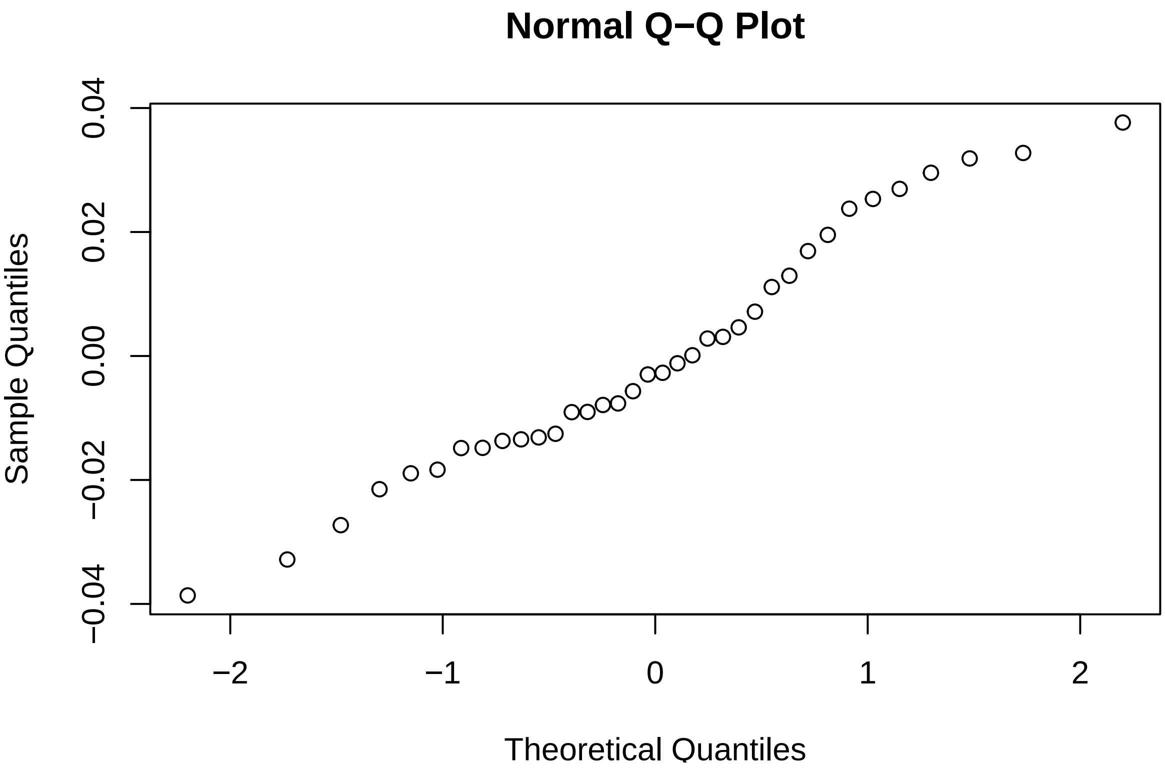

Meets assumptions fine. Model suggests grazing treatment does not influence the MAOM N stock (F1,15 = 1.28, p = 0.277). This appears an accurate conclusion given that underlying grazing type is also non-significant (above)

Part 2 (Section 2) - POM N Stocks Visualization:

```r
POMNstock <- completedata2 %>% dplyr::select(Block, ID, Type, Treatment, Study, POM.N.Stock..kgN.m2.)
yvar <- POMNstock$POM.N.Stock..kgN.m2.

POMNstockTreatment_means <- POMNstock %>% group_by(Treatment) %>% summarise(Treatment_mean_POM.N.Stock.
POMNstockTreatment_means
```

```
## # A tibble: 2 x 2
##   Treatment Treatment_mean_POM.N.Stock..kgN.m2.
##   <chr>                                   <dbl>
## 1 Grazed                                 0.0149
## 2 Ungrazed                               0.0161
```

```r
POMNstockType_means <- POMNstock %>% group_by(Type) %>% summarise(Type_mean_POM.N.Stock..kgN.m2. = mean
POMNstockType_means
```

```
## # A tibble: 3 x 2
##   Type             Type_mean_POM.N.Stock..kgN.m2.
##   <chr>                                     <dbl>
## 1 Autumn-grazed                            0.0144
## 2 Spring-grazed                            0.0153
## 3 Ungrazed Control                         0.0161
```

```r
ggplot(POMNstock) +  geom_point(mapping = aes(x=Treatment, y=yvar, shape = Type, col=Type)) + labs(x =
```

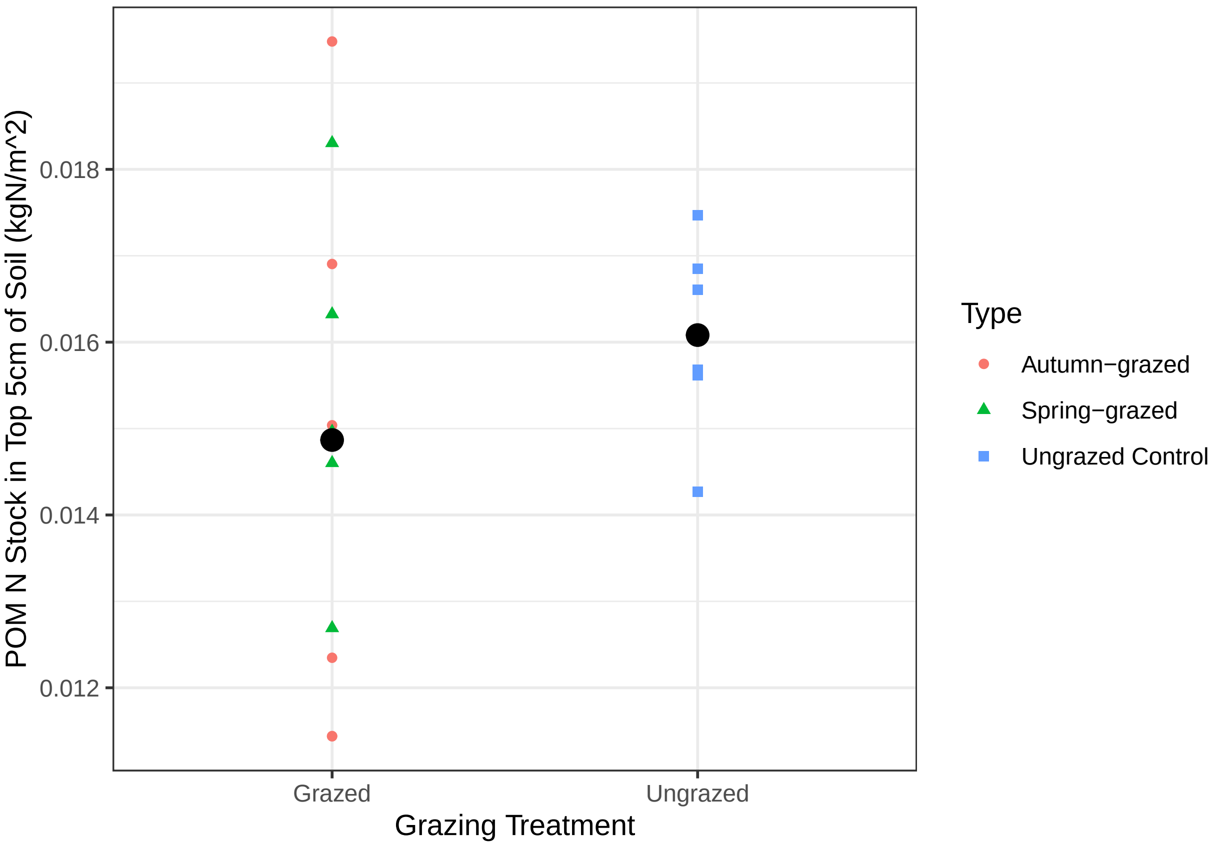


```r
ggplot(POMNstock, aes(x=Type, y=POM.N.Stock..kgN.m2.)) + geom_boxplot(trim=FALSE) + labs(x = "Grazing Ty
```

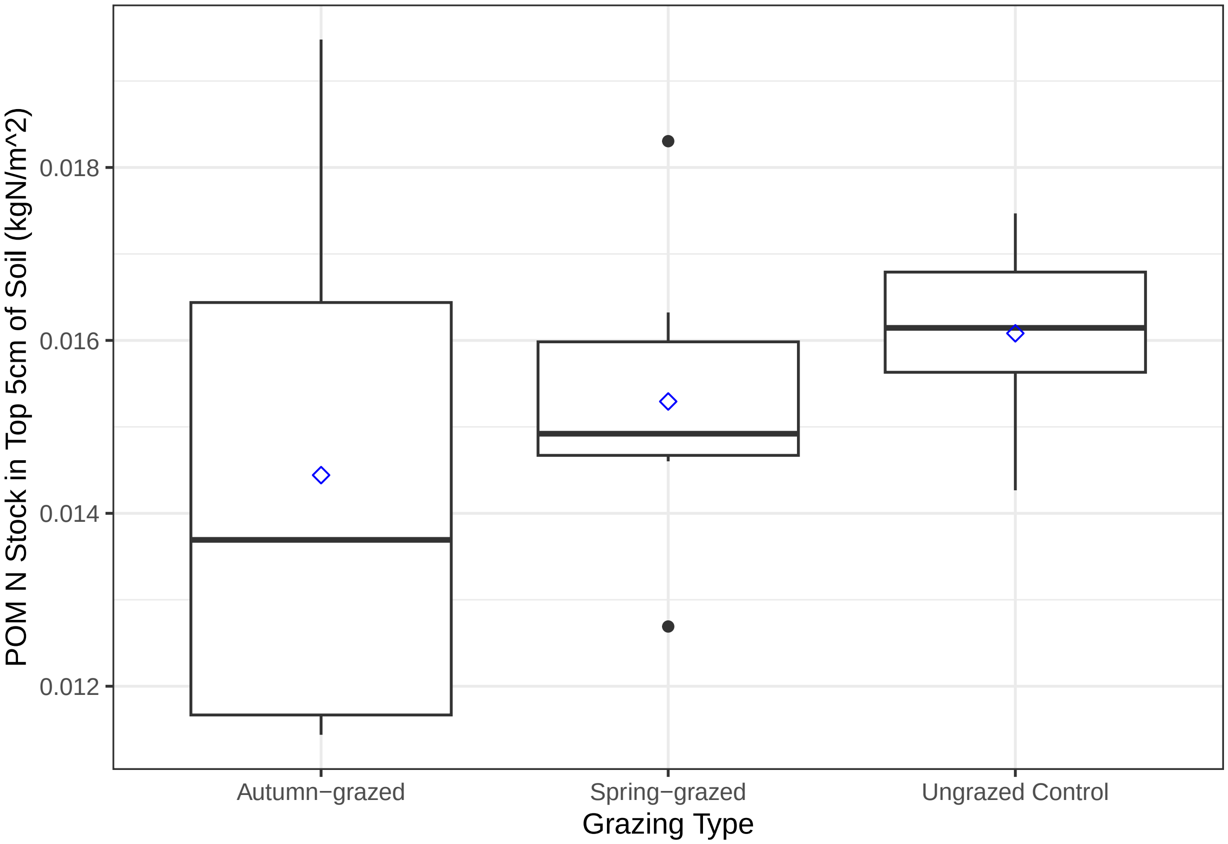


```
# Calculating 95% confidence intervals
NUngrazedPOM <- POMNstock[c(1,2,3,10,11,12),]
1.96 * sd(NUngrazedPOM$POM.N.Stock..kgN.m2.)/sqrt(6)
```

```
## [1] 0.0009101219
```

```
NSpringPOM <- POMNstock[c(7,8,9,16,17,18),]
1.96 * sd(NSpringPOM$POM.N.Stock..kgN.m2.)/sqrt(6)
```

```
## [1] 0.001503858
```

```
NAutumnPOM <- POMNstock[c(4,5,6,13,14,15),]
1.96 * sd(NAutumnPOM$POM.N.Stock..kgN.m2.)/sqrt(6)
```

```
## [1] 0.002634886
```

```
NGrazedPOM <- POMNstock[c(4,5,6,7,8,9,13,14,15,16,17,18),]
1.96 * sd(NGrazedPOM$POM.N.Stock..kgN.m2.)/sqrt(6)
```

```
## [1] 0.002076168
```

Visual conclusions: POM N stock mean is higher in the ungrazed treatment, but again may not be significantly different. Again, grazing type doesn't appear that important. Worth noting, though, that the variation is much greater for autumn-grazed than for spring-grazed on ungrazed control.

Mixed Effects Model looking at the effects of just grazing type (Spring vs Autumn vs Ungrazed Control):

```
POMNstockModel1 <- lme(POM.N.Stock..kgN.m2. ~ Type, random = ~ 1|Block, data = POMNstock)
summary(POMNstockModel1)
```

```
## Linear mixed-effects model fit by REML
```

```
##   Data: POMNstock
##         AIC       BIC   logLik
##   -124.4928 -120.9526 67.24642
##
## Random effects:
##  Formula: ~1 | Block
##          (Intercept)    Residual
## StdDev: 1.074824e-07 0.002285411
##
## Fixed effects:  POM.N.Stock..kgN.m2. ~ Type
##                              Value    Std.Error DF
## (Intercept)          0.014441538 0.0009330151 14
## TypeSpring-grazed    0.000851940 0.0013194827 14
## TypeUngrazed Control 0.001640308 0.0013194827 14
##                        t-value p-value
## (Intercept)          15.478354  0.0000
## TypeSpring-grazed     0.645662  0.5289
## TypeUngrazed Control  1.243145  0.2342
##  Correlation:
##                      (Intr) TypSp-
## TypeSpring-grazed    -0.707
## TypeUngrazed Control -0.707  0.500
##
## Standardized Within-Group Residuals:
##        Min          Q1         Med          Q3         Max
## -1.3141655 -0.6715584 -0.1596573  0.4220596  2.2045539
##
## Number of Observations: 18
## Number of Groups: 2
```

```
anova(POMNstockModel1)
```

```
##             numDF denDF  F-value p-value
## (Intercept)     1    14 803.8075  <.0001
## Type            2    14   0.7731  0.4803
```

```
plot(POMNstockModel1)
```

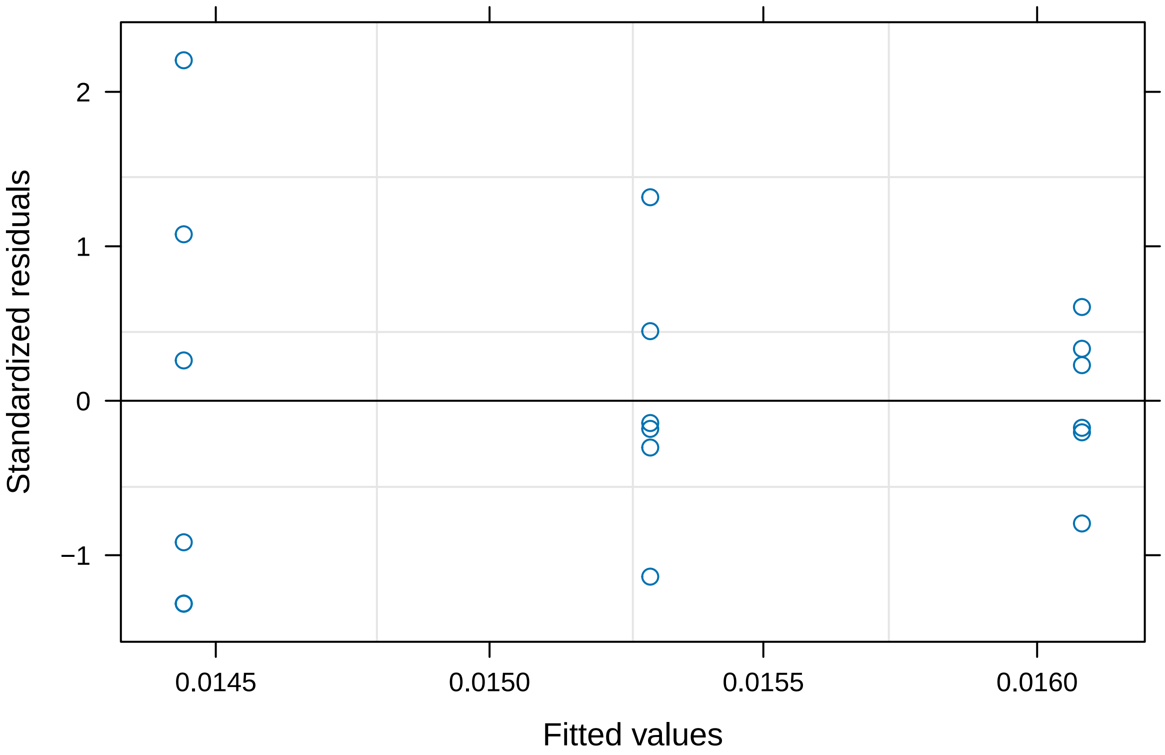


```r
qqnorm(POMNstockModel1$residuals)
```

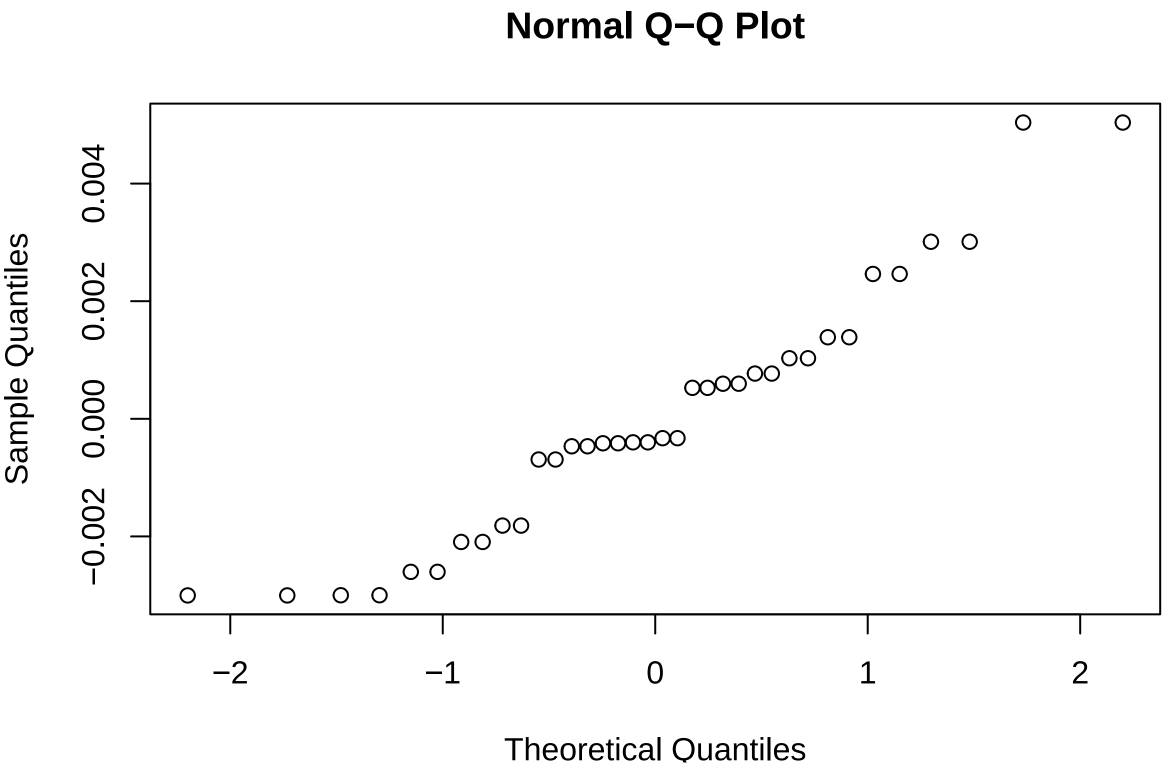


```r
## Changing order to get last comparison
levels(POMNstock$Type)
```

```
## NULL
```

```r
POMNstock$Type <- factor(POMNstock$Type, levels=c('Ungrazed Control', 'Autumn-grazed', 'Spring-grazed'))
POMNstockModel2 <- lme(POM.N.Stock..kgN.m2. ~ Type, random = ~ 1|Block, data = POMNstock)
summary(POMNstockModel2)
```

```
## Linear mixed-effects model fit by REML
##   Data: POMNstock
##         AIC       BIC   logLik
##   -124.4928 -120.9526 67.24642
##
## Random effects:
##  Formula: ~1 | Block
##          (Intercept)    Residual
## StdDev: 1.074867e-07 0.002285411
##
## Fixed effects:  POM.N.Stock..kgN.m2. ~ Type
##                          Value    Std.Error DF   t-value
## (Intercept)        0.016081847 0.0009330151 14 17.236426
## TypeAutumn-grazed -0.001640308 0.0013194827 14 -1.243145
## TypeSpring-grazed -0.000788368 0.0013194827 14 -0.597483
##                   p-value
## (Intercept)        0.0000
## TypeAutumn-grazed  0.2342
## TypeSpring-grazed  0.5597
##  Correlation:
##                   (Intr) TypAt-
## TypeAutumn-grazed -0.707
## TypeSpring-grazed -0.707  0.500
##
## Standardized Within-Group Residuals:
##        Min         Q1        Med         Q3        Max
## -1.3141655 -0.6715584 -0.1596573  0.4220596  2.2045539
##
## Number of Observations: 18
## Number of Groups: 2
```

```r
anova(POMNstockModel2)
```

```
##             numDF denDF  F-value p-value
## (Intercept)     1    14 803.8075  <.0001
## Type            2    14   0.7731  0.4803
```

```r
plot(POMNstockModel2)
```

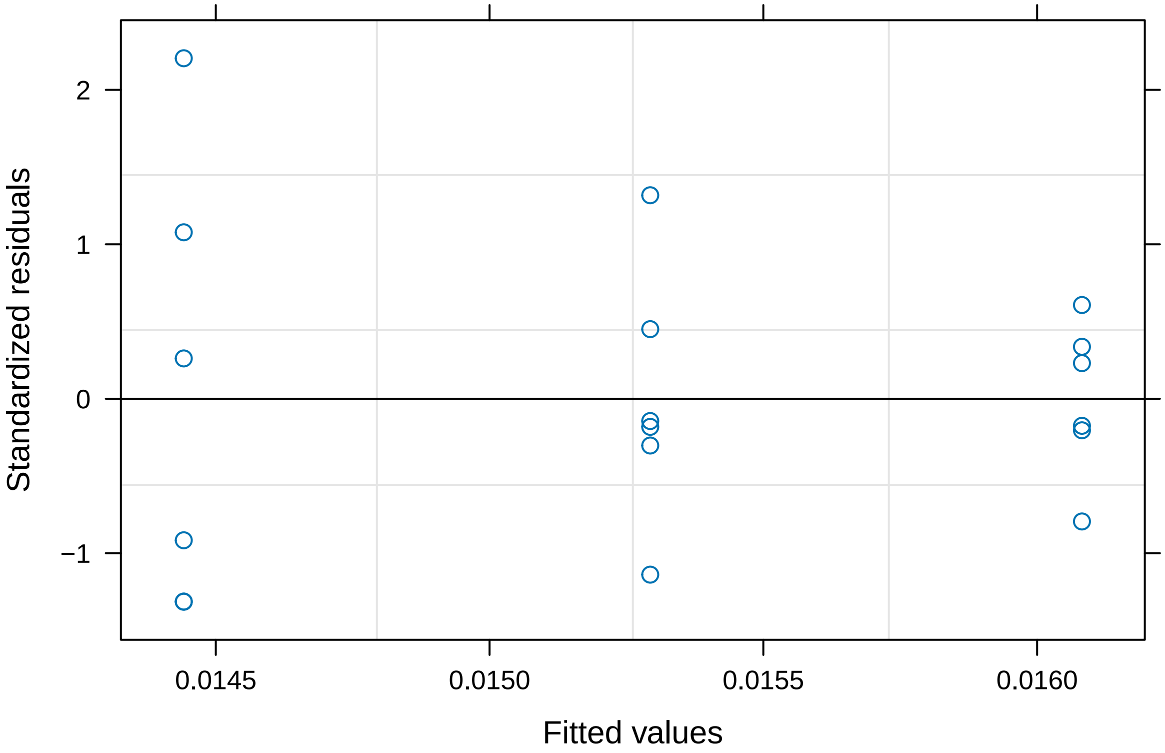


```r
qqnorm(POMNstockModel2$residuals)
```

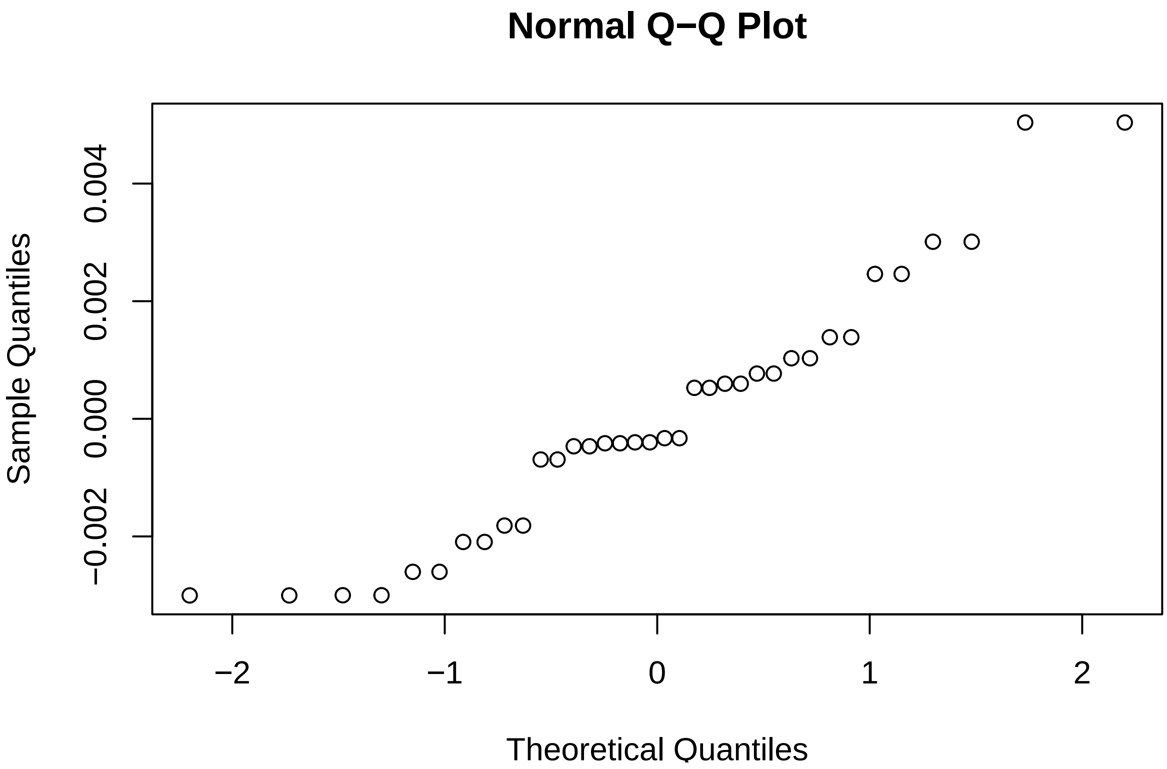

Slightly underdispersed but acceptable. Model suggests grazing type does not affect POM N stock ($F2,14 = 0.773$, $p = 0.480$).

Mixed effects model looking at the effect of just Treatment (grazed vs ungrazed):

```r
POMNstockModel3 <- lme(POM.N.Stock..kgN.m2. ~ Treatment, random = ~ 1|Block, data = POMNstock)
summary(POMNstockModel3)
```

```
## Linear mixed-effects model fit by REML
##   Data: POMNstock
##         AIC       BIC   logLik
##   -137.51 -134.4197 72.75501
##
## Random effects:
##  Formula: ~1 | Block
##          (Intercept)    Residual
## StdDev: 1.057354e-07 0.002243379
##
## Fixed effects:  POM.N.Stock..kgN.m2. ~ Treatment
##                          Value    Std.Error DF   t-value
## (Intercept)       0.014867508 0.0006476076 15 22.957587
## TreatmentUngrazed 0.001214338 0.0011216893 15  1.082598
##                    p-value
## (Intercept)         0.0000
## TreatmentUngrazed  0.2961
##  Correlation:
##                   (Intr)
## TreatmentUngrazed -0.577
##
## Standardized Within-Group Residuals:
##         Min          Q1         Med          Q3
## -1.52866680 -0.65907921  0.02366594  0.54950303
##         Max
##  2.05598009
##
## Number of Observations: 18
## Number of Groups: 2
```

```r
anova(POMNstockModel3)
```

```
##             numDF denDF  F-value p-value
## (Intercept)     1    15 834.2103  <.0001
## Treatment       1    15   1.1720  0.2961
```

```r
plot(POMNstockModel3)
```

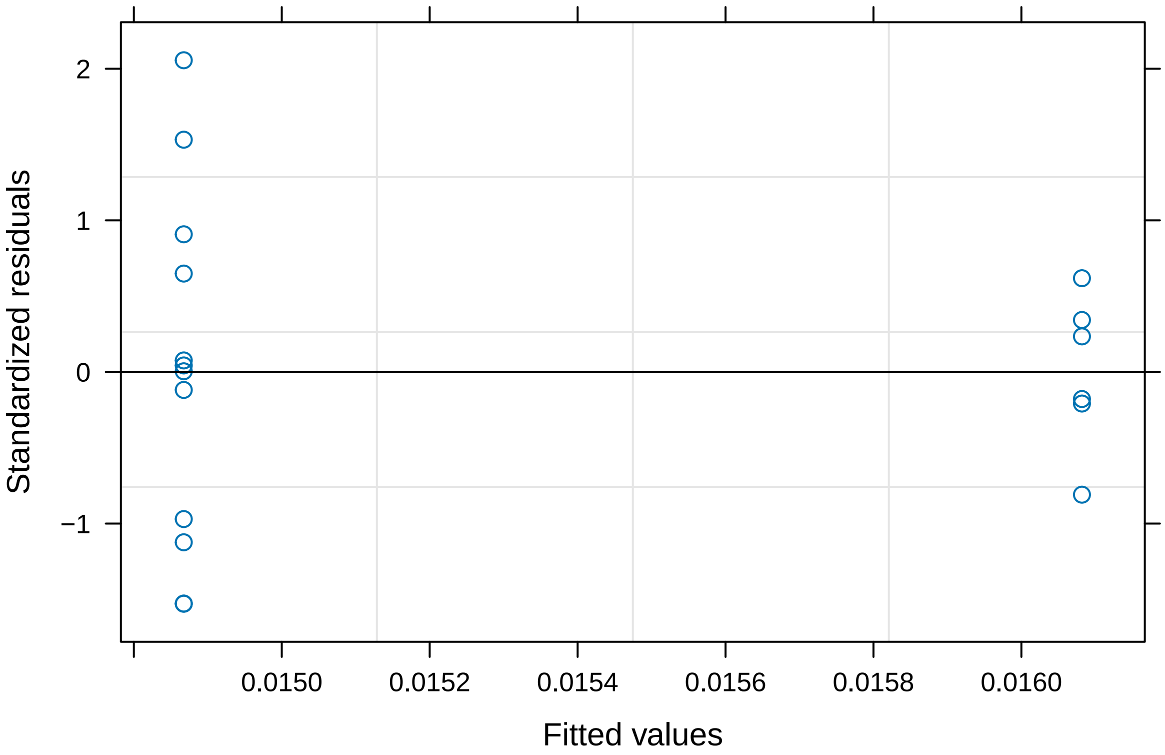


```r
qqnorm(POMNstockModel3$residuals)
```

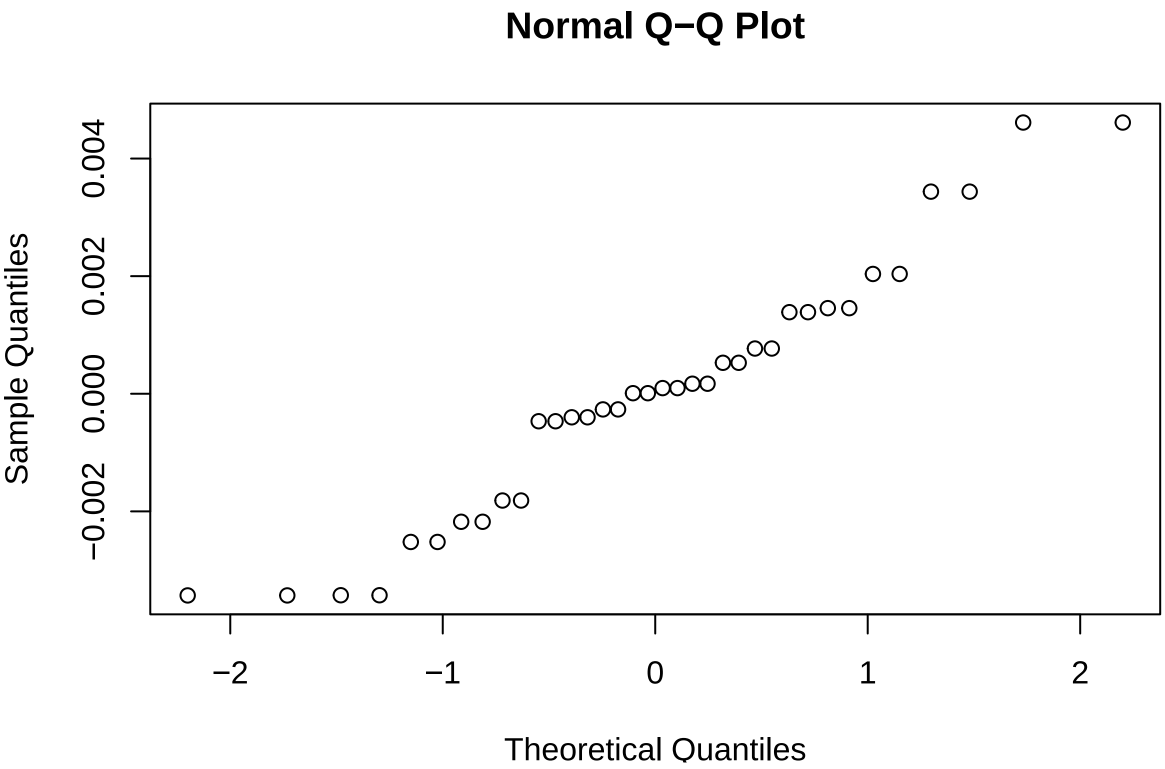

Also underdispersed but acceptable. Suggests that grazing does not influence POM N stock ($F_{1,15}$ = 1.17, p = 0.296). This is the accurate conclusion given above results that underlying grazing type is non-significant.

Part 2 (Section 3) - Total SOM N Stocks SOM N stocks are a good proxy for total nitrogen in the soil given that estimates suggest up to 88% of terrestrial nitrogen is stored in SOM. Visualization:

```r
TotalSOMNstock <- completedata2 %>% dplyr::select(Block, ID, Type, Treatment, Study, Total.SOM.N.Stock.
yvar <- TotalSOMNstock$Total.SOM.N.Stock..kgN.m2.

TotalSOMNstockTreatment_means <- TotalSOMNstock %>% group_by(Treatment) %>% summarise(Treatment_mean_To
TotalSOMNstockTreatment_means
```

```
## # A tibble: 2 x 2
##   Treatment Treatment_mean_Total.SOM.N.Stock..kgN.m2.
##   <chr>                                         <dbl>
## 1 Grazed                                        0.257
## 2 Ungrazed                                      0.269
```

```r
TotalSOMNstockType_means <- TotalSOMNstock %>% group_by(Type) %>% summarise(Type_mean_Total.SOM.N.Stock
TotalSOMNstockType_means
```

```
## # A tibble: 3 x 2
##   Type             Type_mean_Total.SOM.N.Stock..kgN.m2.
##   <chr>                                           <dbl>
## 1 Autumn-grazed                                   0.259
## 2 Spring-grazed                                   0.254
## 3 Ungrazed Control                                0.269
```

```r
ggplot(TotalSOMNstock) +  geom_point(mapping = aes(x=Treatment, y=yvar, shape = Type, col=Type)) + labs
```

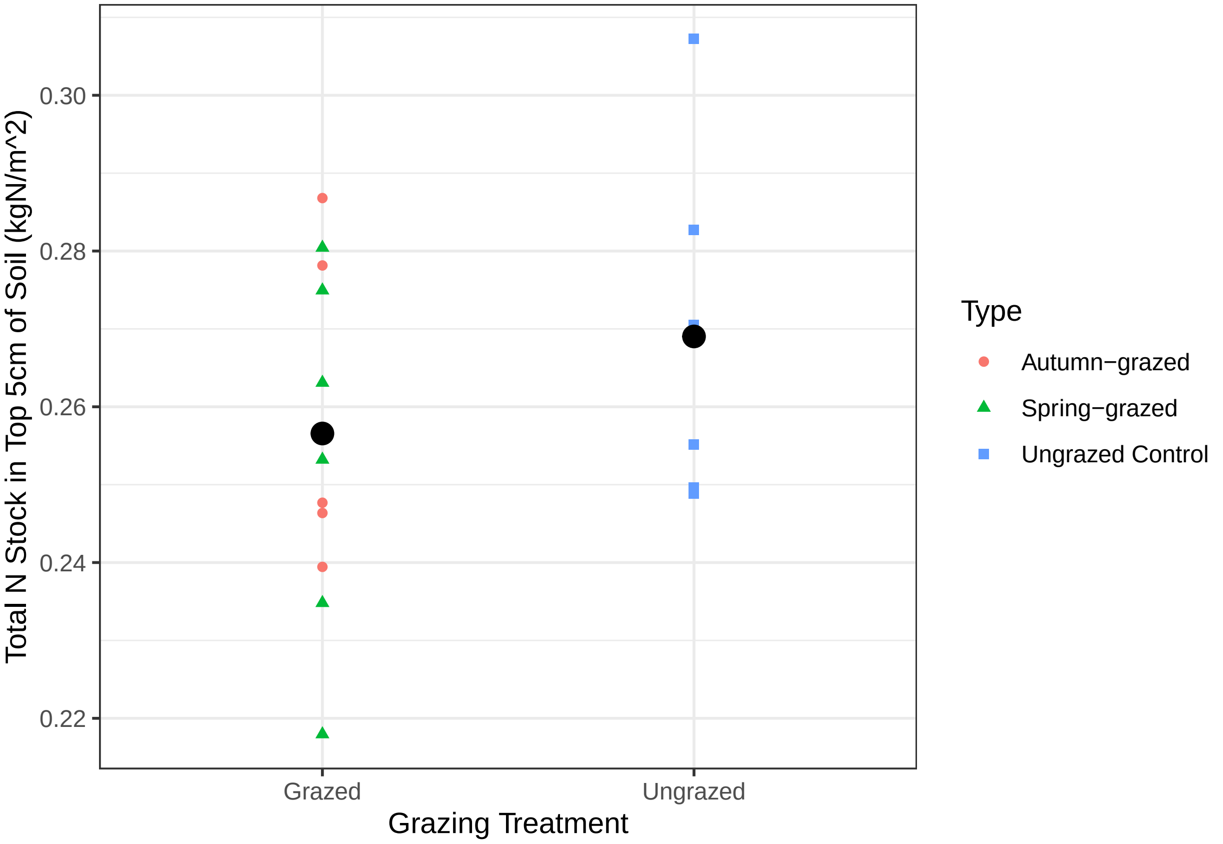


```r
ggplot(TotalSOMNstock, aes(x=Type, y=Total.SOM.N.Stock..kgN.m2.)) + geom_boxplot(trim=FALSE) + labs(x =
```

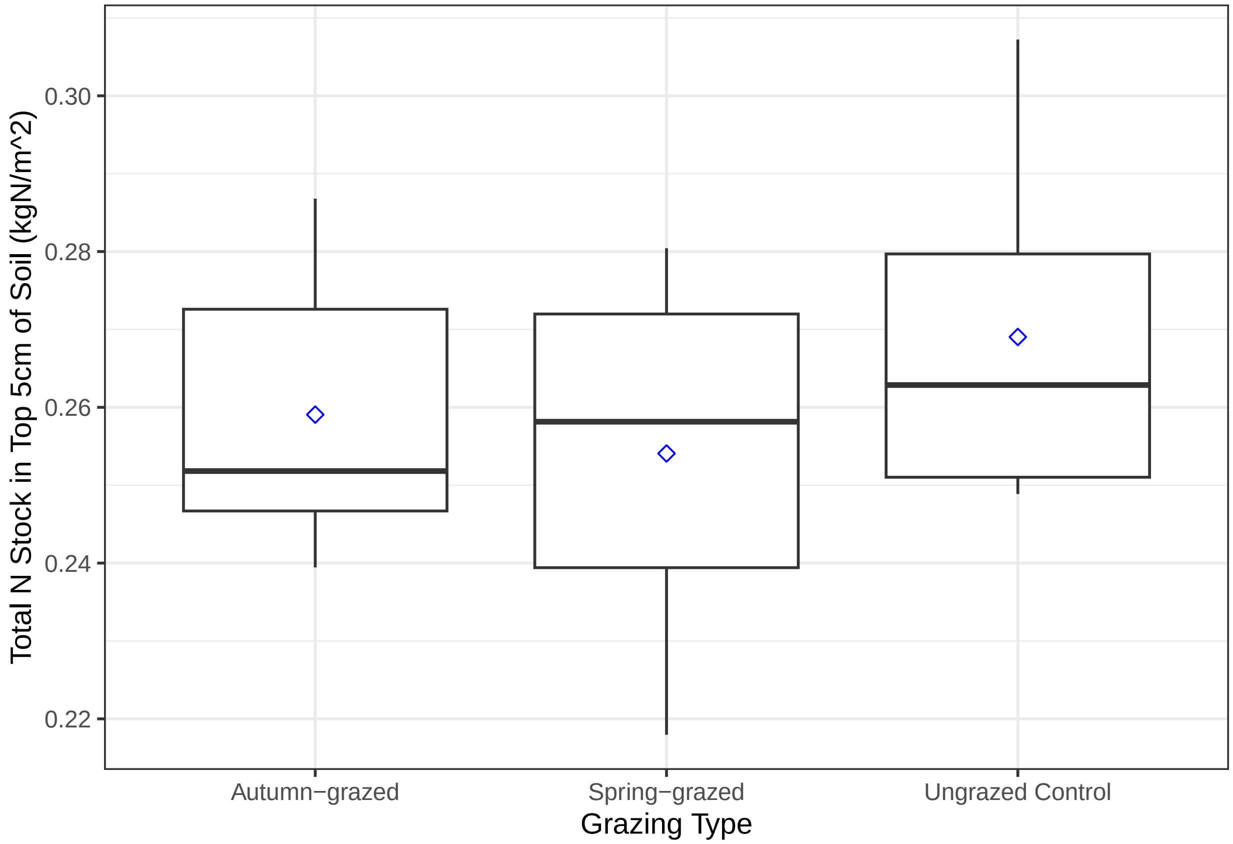


```r
# Calculating 95% confidence intervals
NUngrazedTotal <- TotalSOMNstock[c(1,2,3,10,11,12),]
1.96 * sd(NUngrazedTotal$Total.SOM.N.Stock..kgN.m2.)/sqrt(6)
```

```
## [1] 0.01832849
```

```r
NSpringTotal <- TotalSOMNstock[c(7,8,9,16,17,18),]
1.96 * sd(NSpringTotal$Total.SOM.N.Stock..kgN.m2.)/sqrt(6)
```

```
## [1] 0.01923128
```

```r
NAutumnTotal <- TotalSOMNstock[c(4,5,6,13,14,15),]
1.96 * sd(NAutumnTotal$Total.SOM.N.Stock..kgN.m2.)/sqrt(6)
```

```
## [1] 0.01526221
```

```r
NGrazedTotal <- TotalSOMNstock[c(4,5,6,7,8,9,13,14,15,16,17,18),]
1.96 * sd(NGrazedTotal$Total.SOM.N.Stock..kgN.m2.)/sqrt(6)
```

```
## [1] 0.01668349
```

Visual conclusions: Mean total N stock is higher in ungrazed (this reflects the higher MAOM N stock in ungrazed), but unlikely to be significant. Grazing type doesn't seem to be important.

Mixed Effects Model looking at the effects of just grazing type (Spring vs Autumn vs Ungrazed Control):

```r
TotalSOMNstockModel1 <- lme(Total.SOM.N.Stock..kgN.m2. ~ Type, random = ~ 1|Block, data = TotalSOMNstoc
summary(TotalSOMNstockModel1)
```

```
## Linear mixed-effects model fit by REML
##   Data: TotalSOMNstock
```

```
##         AIC       BIC   logLik
##   -57.24364 -53.70339 33.62182
##
## Random effects:
##  Formula: ~1 | Block
##         (Intercept)   Residual
## StdDev: 0.009989658 0.02070827
##
## Fixed effects:  Total.SOM.N.Stock..kgN.m2. ~ Type
##                             Value  Std.Error DF   t-value
## (Intercept)            0.25906483 0.01101675 14 23.515548
## TypeSpring-grazed     -0.00499065 0.01195592 14 -0.417420
## TypeUngrazed Control   0.00996782 0.01195592 14  0.833714
##                       p-value
## (Intercept)            0.0000
## TypeSpring-grazed      0.6827
## TypeUngrazed Control   0.4184
##  Correlation:
##                       (Intr) TypSp-
## TypeSpring-grazed     -0.543
## TypeUngrazed Control -0.543  0.500
##
## Standardized Within-Group Residuals:
##        Min         Q1        Med         Q3        Max
## -1.4636623 -0.6641884 -0.2703946  0.6409320  1.5636082
##
## Number of Observations: 18
## Number of Groups: 2
```

```
anova(TotalSOMNstockModel1)
```

```
##             numDF denDF  F-value p-value
## (Intercept)     1    14 922.0882  <.0001
## Type            2    14   0.8116   0.464
```

```
plot(TotalSOMNstockModel1)
```

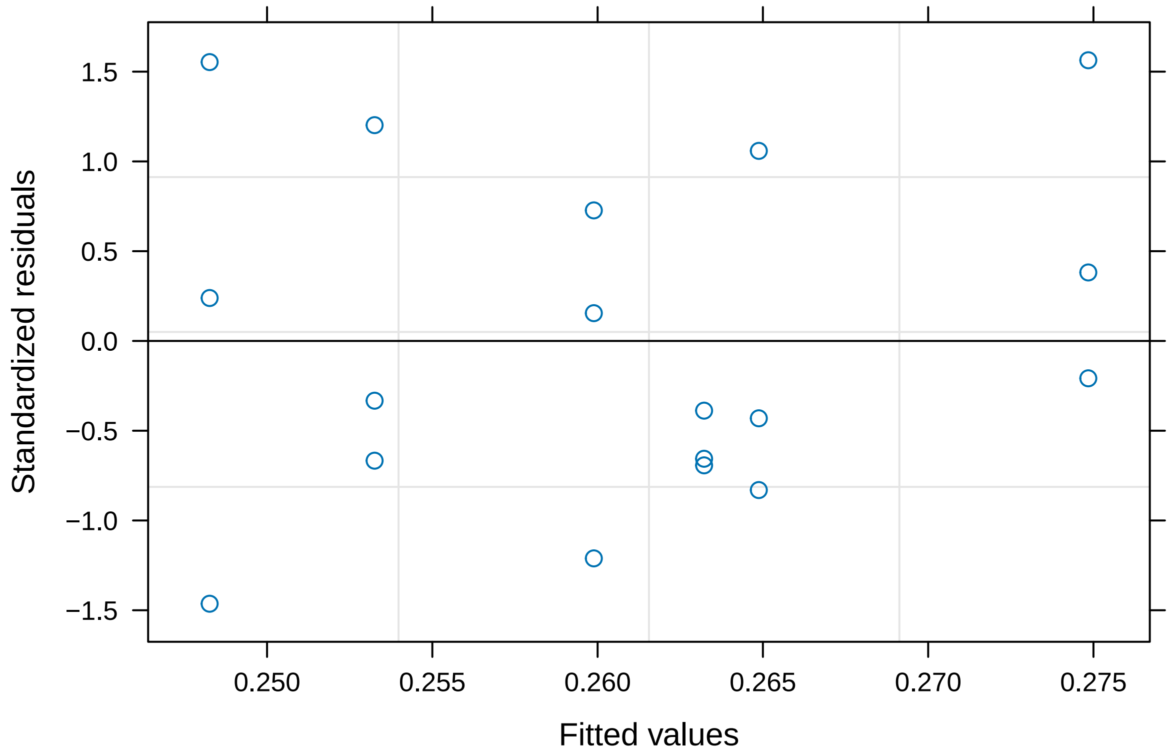


```
qqnorm(TotalSOMNstockModel1$residuals)
```

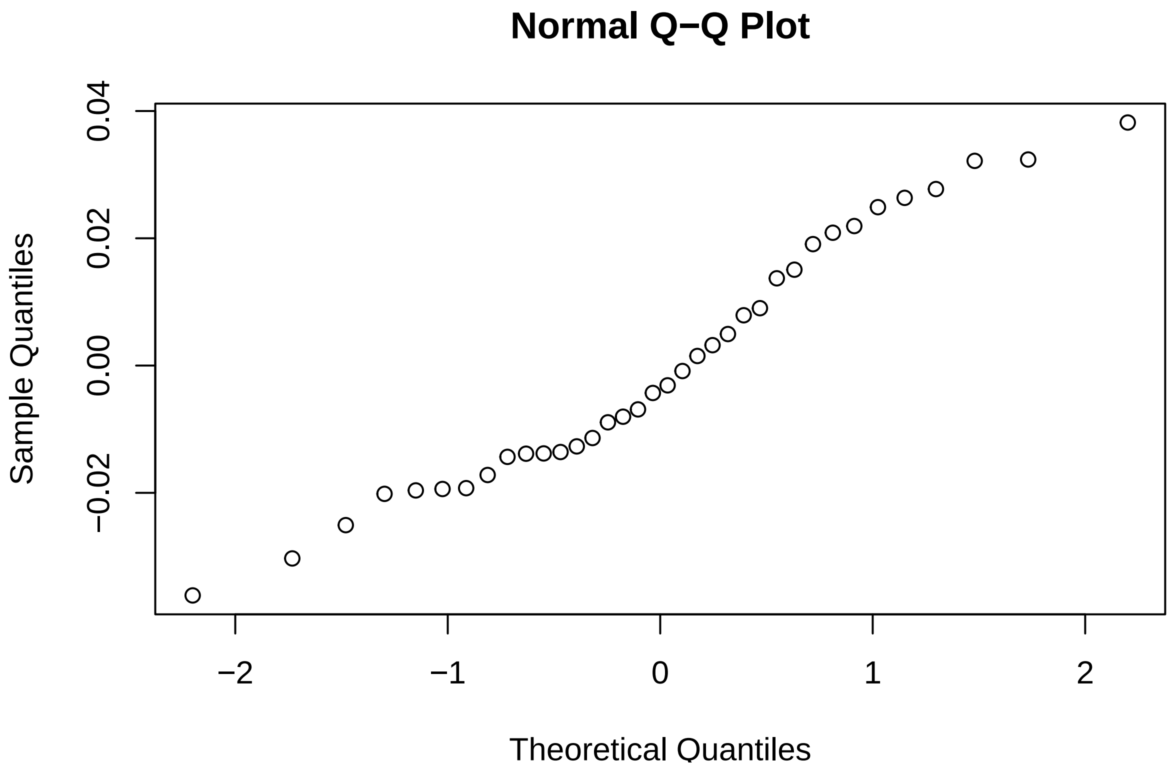


```
## Changing order to get last comparison
levels(TotalSOMNstock$Type)
```

```
## NULL
```

```r
TotalSOMNstock$Type <- factor(TotalSOMNstock$Type, levels=c('Spring-grazed','Ungrazed Control','Autumn-g
TotalSOMNstockModel2 <- lme(Total.SOM.N.Stock..kgN.m2. ~ Type, random = ~ 1|Block, data = TotalSOMNstoc
summary(TotalSOMNstockModel2)
```

```
## Linear mixed-effects model fit by REML
##   Data: TotalSOMNstock
##         AIC       BIC   logLik
##   -57.24364 -53.70339 33.62182
##
## Random effects:
##  Formula: ~1 | Block
##         (Intercept)   Residual
## StdDev: 0.009989658 0.02070827
##
## Fixed effects:  Total.SOM.N.Stock..kgN.m2. ~ Type
##                           Value  Std.Error DF   t-value
## (Intercept)          0.25407419 0.01101675 14 23.062543
## TypeUngrazed Control 0.01495847 0.01195592 14  1.251134
## TypeAutumn-grazed    0.00499065 0.01195592 14  0.417420
##                      p-value
## (Intercept)           0.0000
## TypeUngrazed Control  0.2314
## TypeAutumn-grazed     0.6827
##  Correlation:
##                      (Intr) TypUnC
## TypeUngrazed Control -0.543
## TypeAutumn-grazed    -0.543  0.500
##
## Standardized Within-Group Residuals:
##        Min         Q1        Med         Q3        Max
## -1.4636623 -0.6641884 -0.2703946  0.6409320  1.5636082
##
## Number of Observations: 18
## Number of Groups: 2
```

```r
anova(TotalSOMNstockModel2)
```

```
##             numDF denDF  F-value p-value
## (Intercept)     1    14 922.0882  <.0001
## Type            2    14   0.8116   0.464
```

```r
plot(TotalSOMNstockModel2)
```

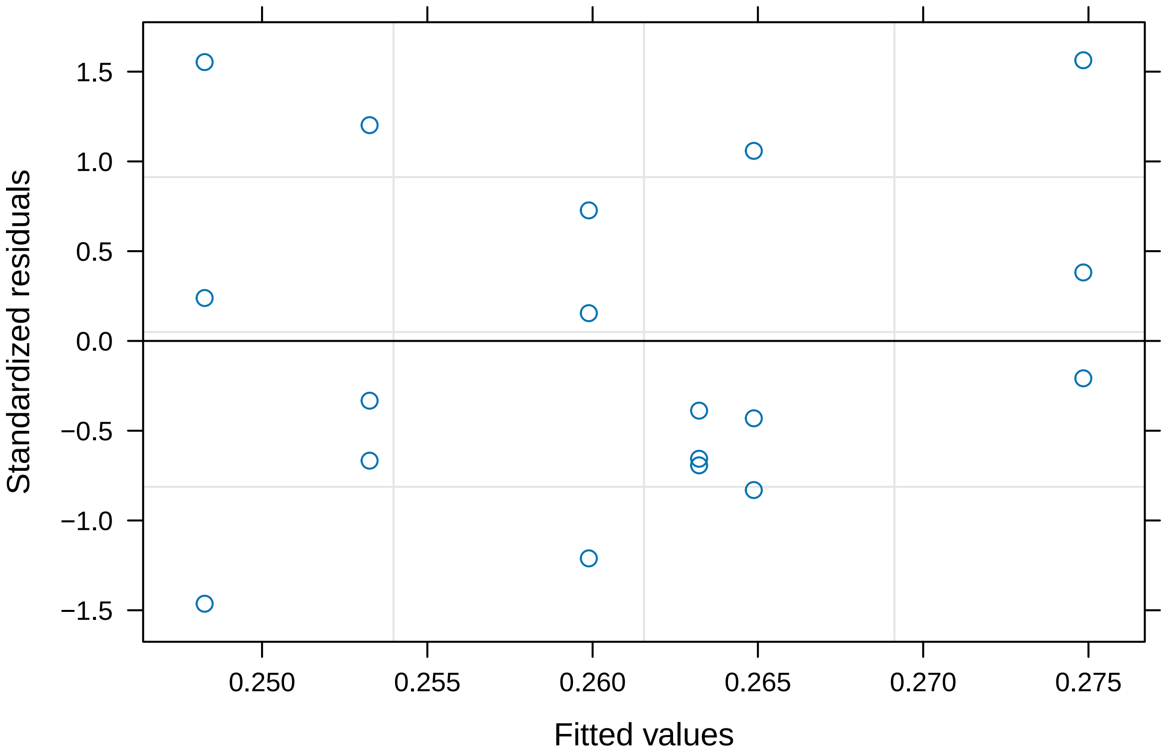


```r
qqnorm(TotalSOMNstockModel2$residuals)
```

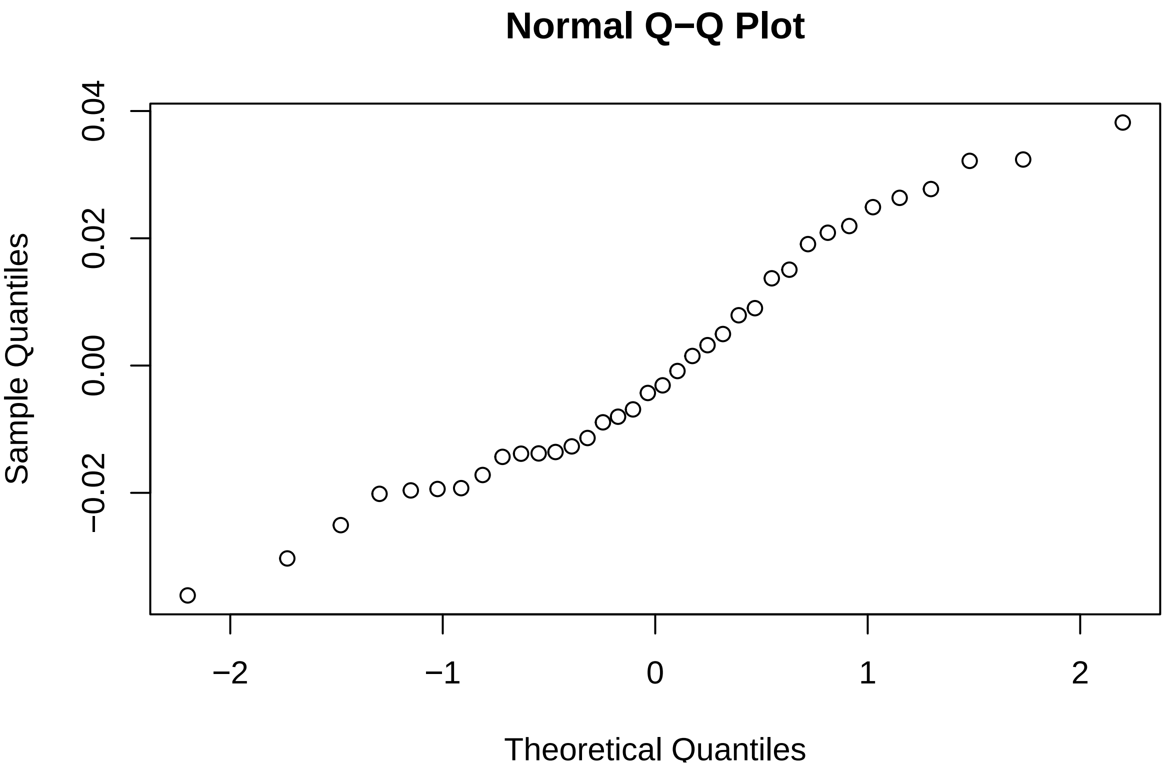

Meets assumptions fine. Suggests grazing type does not influence the total N stock stored in the SOM (F2,14 = 0.812, p = 0.464).

Mixed effects model looking at the effect of just Treatment (grazed vs ungrazed):

```r
TotalSOMNstockModel3 <- lme(Total.SOM.N.Stock..kgN.m2. ~ Treatment, random = ~ 1|Block, data = TotalSOM
summary(TotalSOMNstockModel3)
```

```
## Linear mixed-effects model fit by REML
##   Data: TotalSOMNstock
##         AIC       BIC   logLik
##   -66.10818 -63.01783 37.05409
##
## Random effects:
##  Formula: ~1 | Block
##         (Intercept)  Residual
## StdDev:   0.0101201 0.0201302
##
## Fixed effects:  Total.SOM.N.Stock..kgN.m2. ~ Treatment
##                          Value   Std.Error DF   t-value
## (Intercept)       0.25656951 0.009218292 15 27.832651
## TreatmentUngrazed 0.01246314 0.010065099 15  1.238253
##                   p-value
## (Intercept)        0.0000
## TreatmentUngrazed  0.2347
##  Correlation:
##                   (Intr)
## TreatmentUngrazed -0.364
##
## Standardized Within-Group Residuals:
##        Min         Q1        Med         Q3        Max
## -1.6220647 -0.6391281 -0.2161798  0.5587799  1.6009216
##
## Number of Observations: 18
## Number of Groups: 2
```

```r
anova(TotalSOMNstockModel3)
```

```
##             numDF denDF  F-value p-value
## (Intercept)     1    15 922.0881  <.0001
## Treatment       1    15   1.5333  0.2347
```

```r
plot(TotalSOMNstockModel3)
```

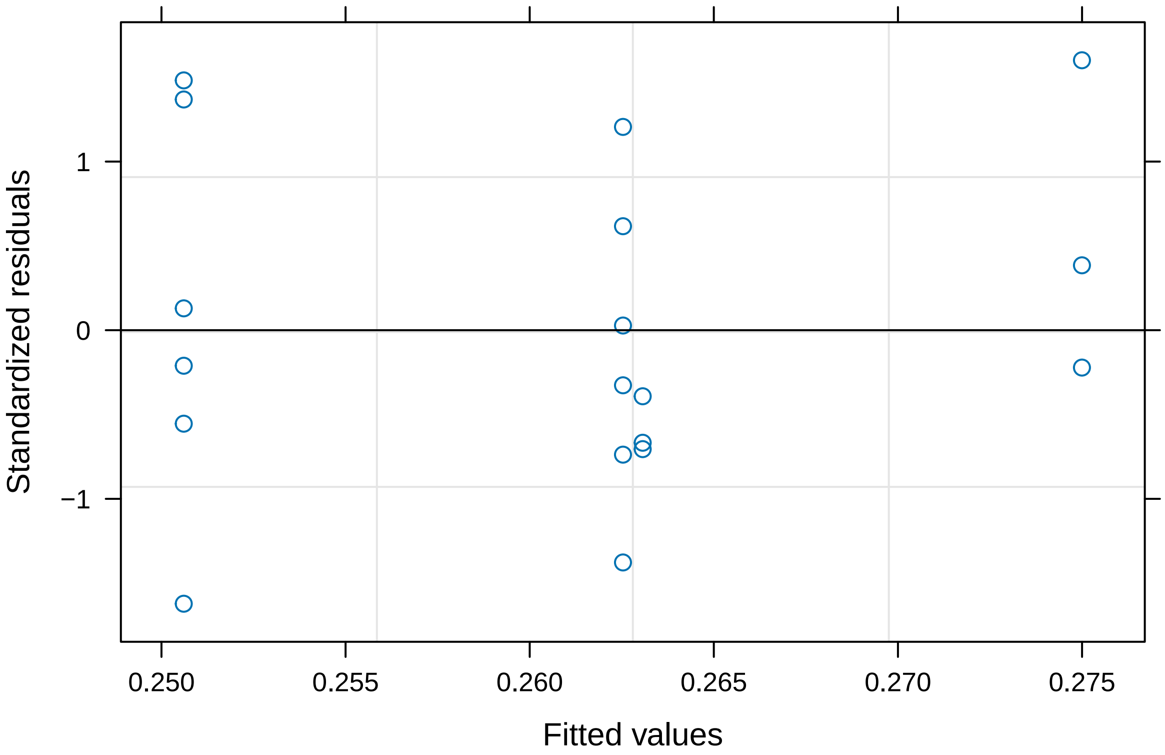


```r
qqnorm(TotalSOMNstockModel3$residuals)
```

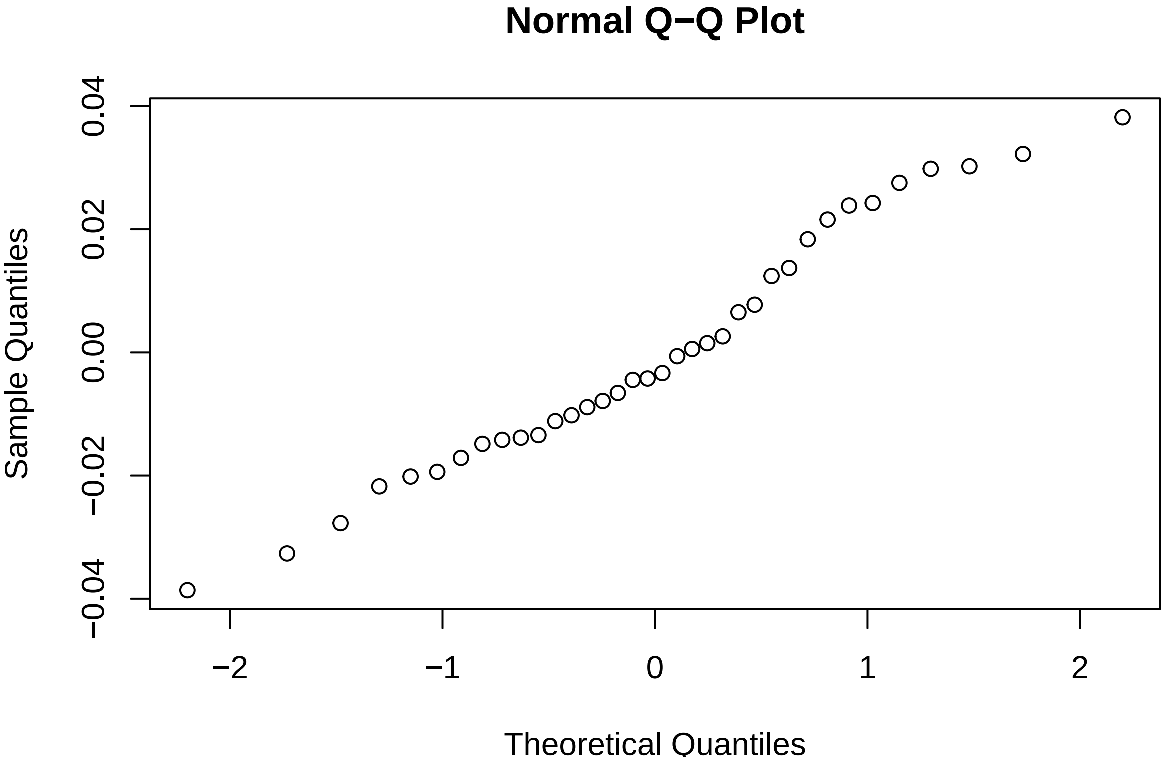

Meets assumptions fine. Suggests that grazing does not produce a change in total SOM N stock ($F_{1,15} = 1.53$, $p = 0.235$).

N Stocks Bar chart:

```r
Nbardata <- completedata %>% dplyr::select(Survey, Fraction, N.stocks)
Nbardata <- Nbardata[c(1,2,3,4,5,6,7,8),]
ggplot(Nbardata, aes(fill=Fraction, y=N.stocks, x=Survey)) +
  geom_bar(position='stack', stat='identity')
```

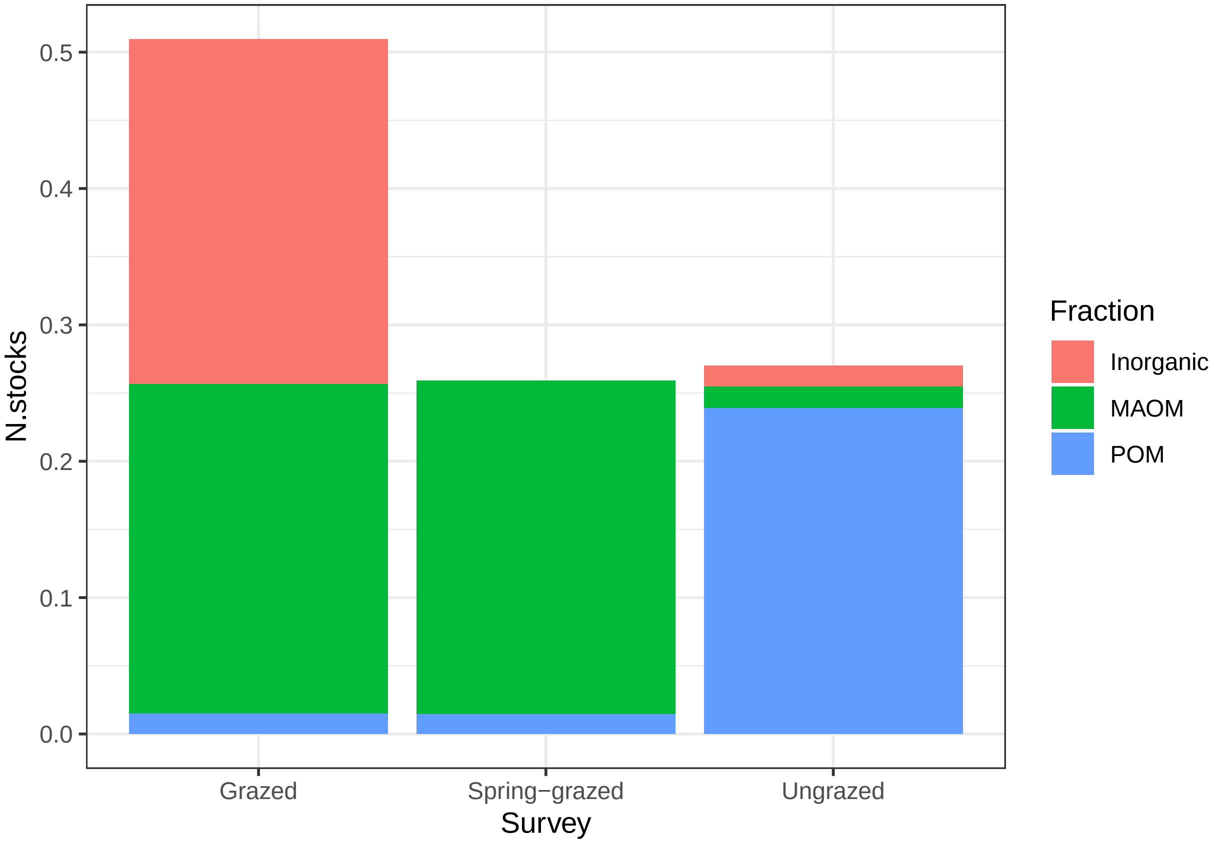


```r
levels(Nbardata$Survey)
```

```
## NULL
```

```r
Nbardata$Survey <- factor (Nbardata$Survey, levels=c('Ungrazed', 'Grazed','Spring-grazed','Autumn-graze
ggplot(Nbardata, aes(fill=Fraction, y=N.stocks, x=Survey)) +
  geom_bar(position='stack', stat='identity') + labs(x = "Grazing Treatment", y = "Nitrogen Stock in To
```

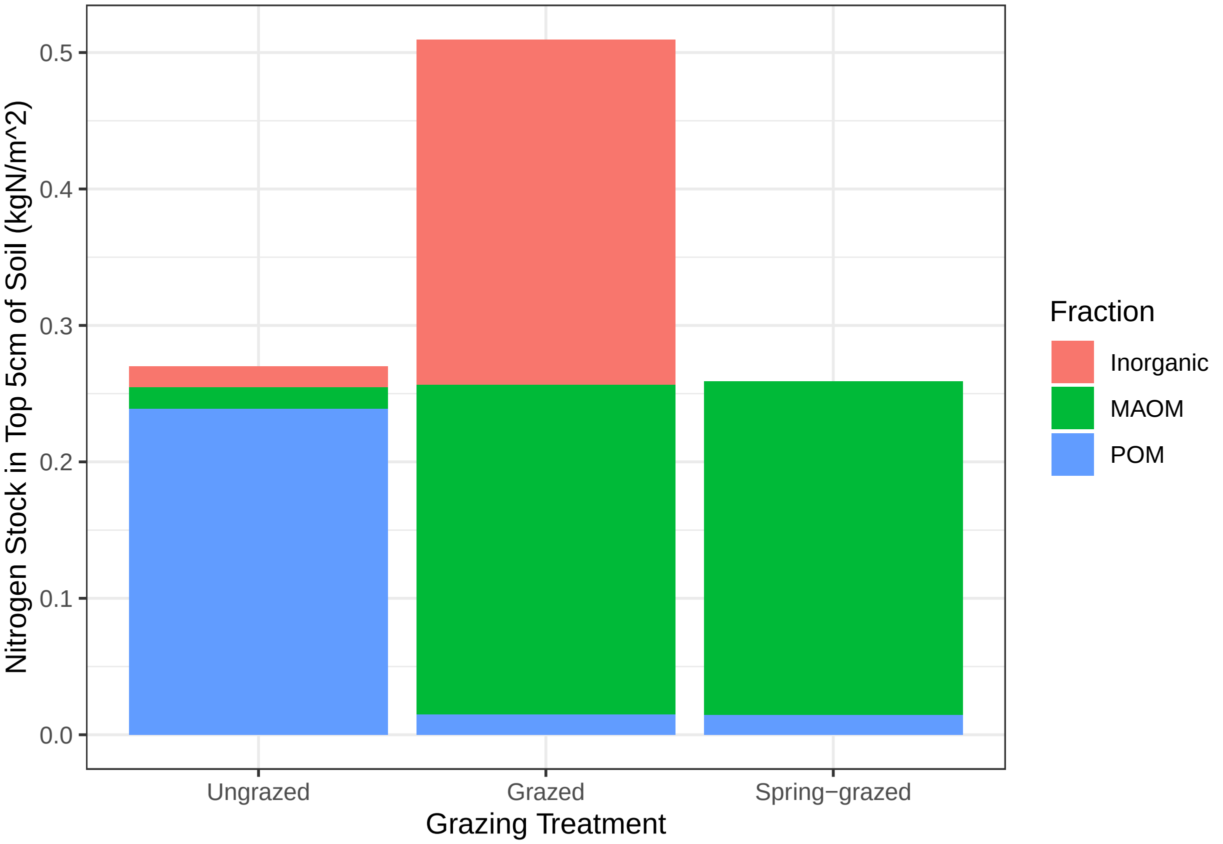


```
Nbardata4 <-Nbardata[c(3,4,5,6,7,8),]
ggplot(Nbardata4, aes(fill=Fraction, y=N.stocks, x=Survey)) +
  geom_bar(position='stack', stat='identity') + labs(x = "Grazing Type", y = "Nitrogen Stock in Top 5cm
```

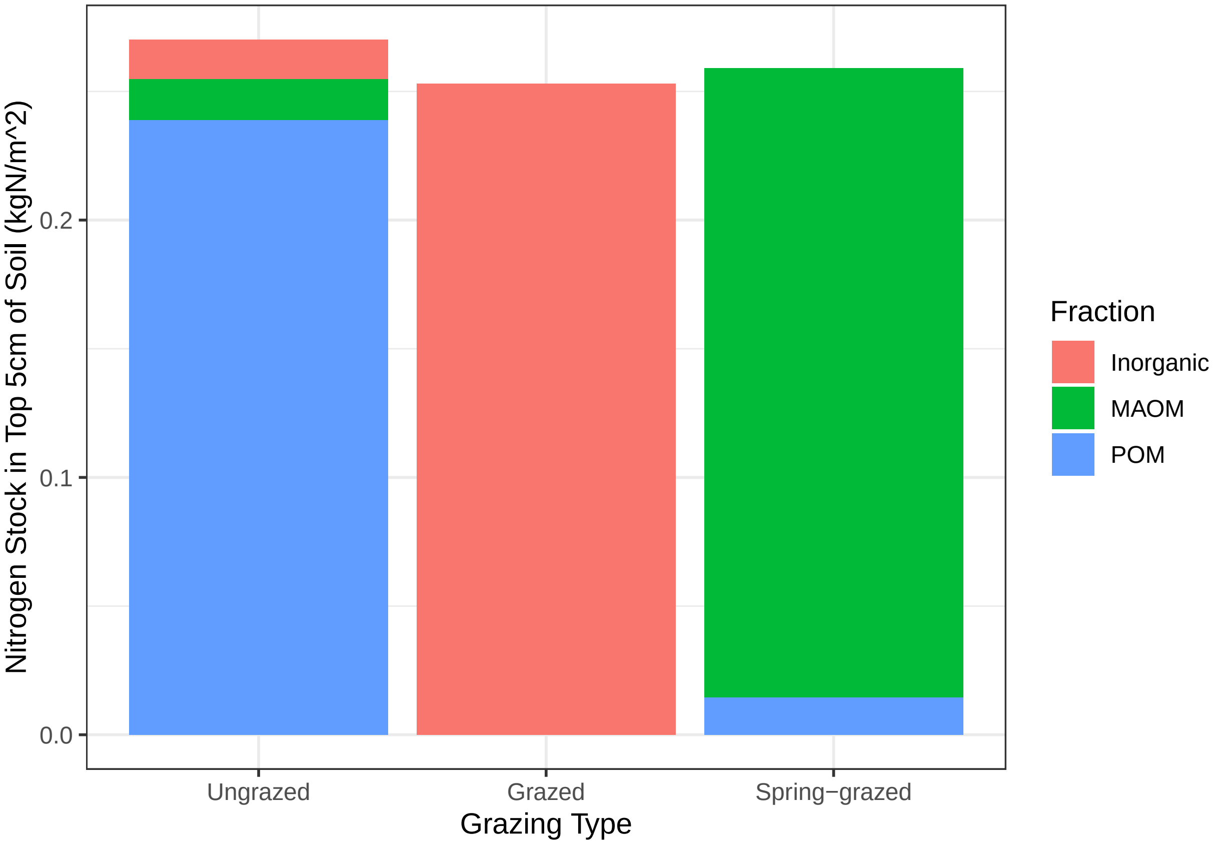


```
Nbardata2 <-Nbardata[c(1,2,3,4),]
Nbardata3 <-Nbardata[c(5,6,7,8),]
ggplot(Nbardata2, aes(fill=Fraction, y=N.stocks, x=Survey)) +
  geom_bar(position='stack', stat='identity') + ylim(0.0,0.3) + labs(x = "Grazing Treatment", y = "Nitro
```

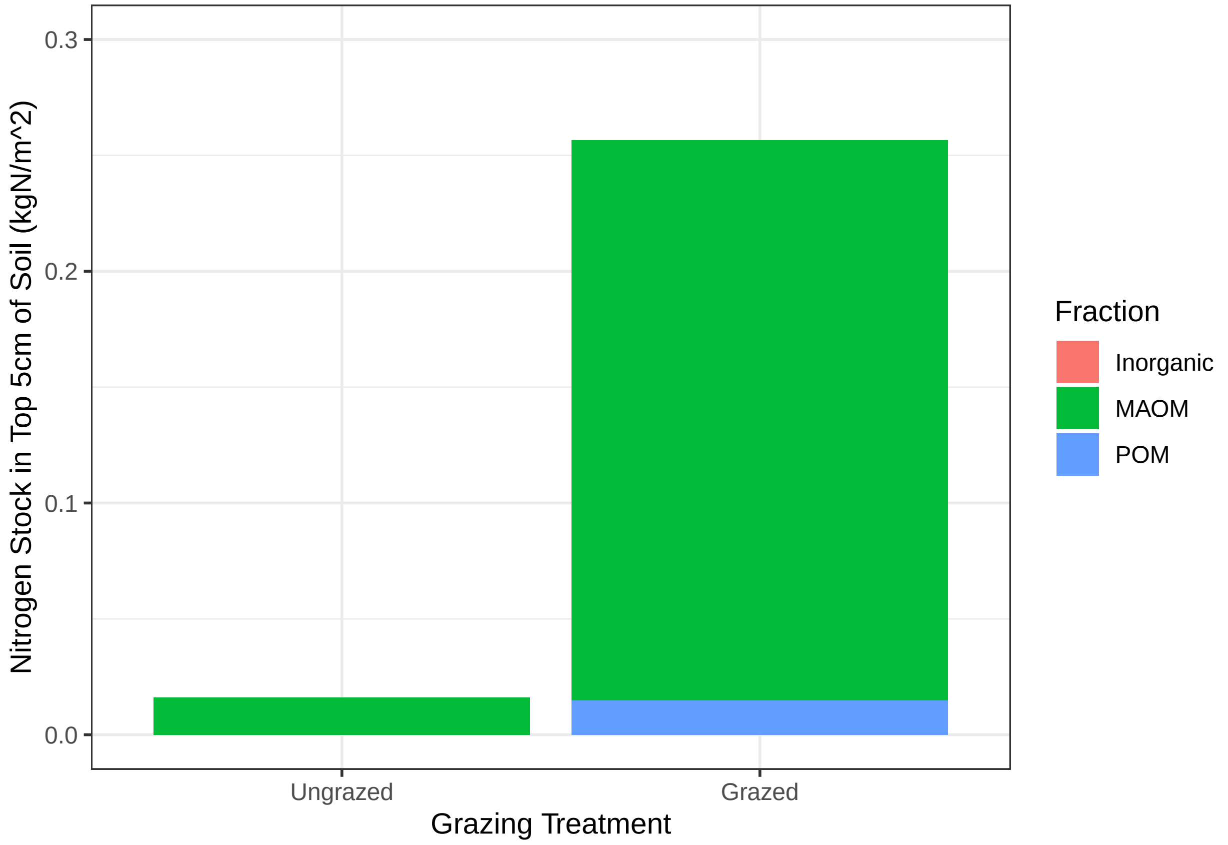


```
ggplot(Nbardata3, aes(fill=Fraction, y=N.stocks, x=Survey)) +
  geom_bar(position='stack', stat='identity') + ylim(0.0,0.3) + labs(x = "Grazing Type", y = "Nitrogen S
```

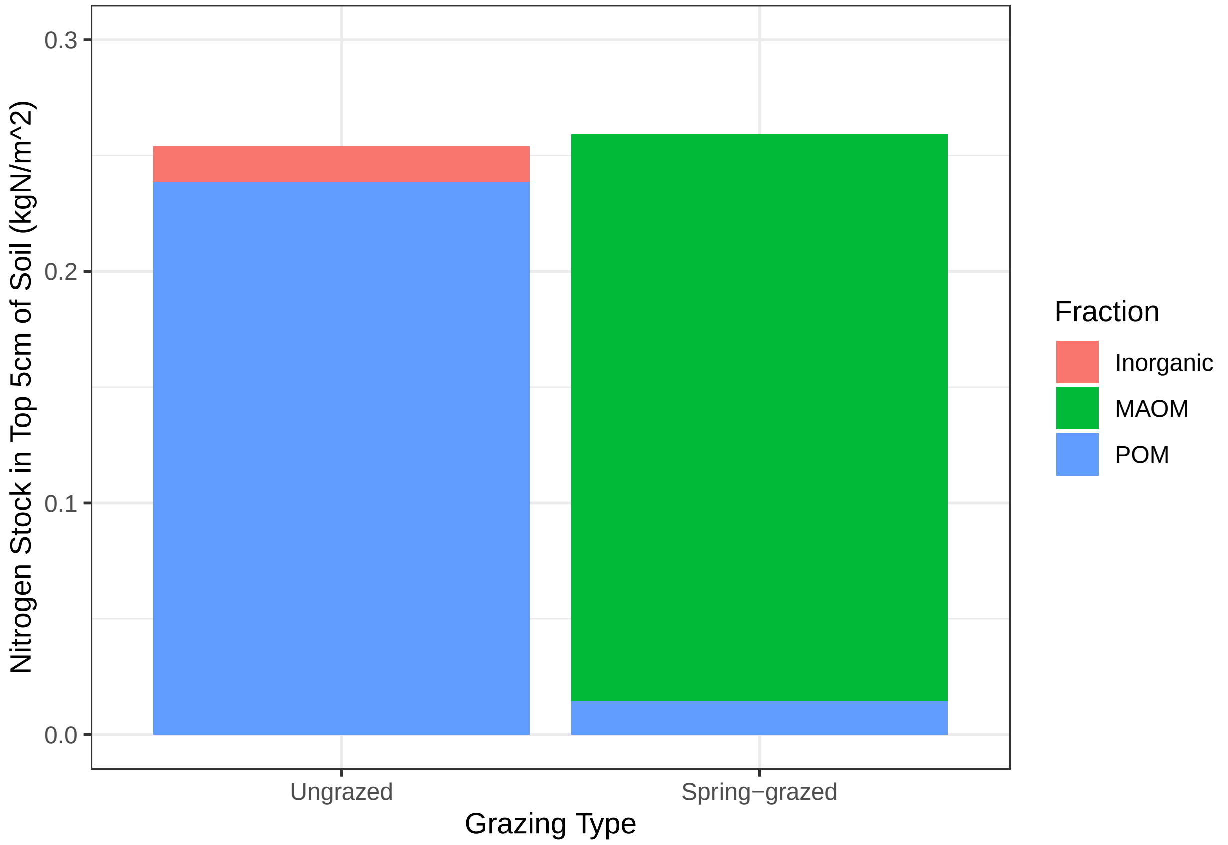


```
levels(Nbardata2$Survey)
```

```
## [1] "Ungrazed"      "Grazed"        "Spring-grazed"
## [4] "Autumn-grazed"
```

Part 3 - C/N Ratio Part 3 (Section 1) - MAOM C/N Ratio Visualization:

```
MAOMRatio <- completedata2 %>% dplyr::select(Block, ID, Type, Treatment, Study, MAOM.C.N.Ratio)
yvar <- MAOMRatio$MAOM.C.N.Ratio

MAOMRatioTreatment_means <- MAOMRatio %>% group_by(Treatment) %>% summarise(Treatment_mean_MAOM.C.N.Rat
MAOMRatioTreatment_means
```

```
## # A tibble: 2 x 2
##   Treatment Treatment_mean_MAOM.C.N.Ratio
##   <chr>                             <dbl>
## 1 Grazed                             12.1
## 2 Ungrazed                           11.5
```

```
MAOMRatioType_means <- MAOMRatio %>% group_by(Type) %>% summarise(Type_mean_MAOM.C.N.Ratio = mean(MAOM.
MAOMRatioType_means
```

```
## # A tibble: 3 x 2
##   Type             Type_mean_MAOM.C.N.Ratio
##   <chr>                               <dbl>
## 1 Autumn-grazed                        11.8
## 2 Spring-grazed                        12.4
## 3 Ungrazed Control                     11.5
```

```
ggplot(MAOMRatio) +  geom_point(mapping = aes(x=Treatment, y=yvar, shape = Type, col=Type)) + labs(x =
```

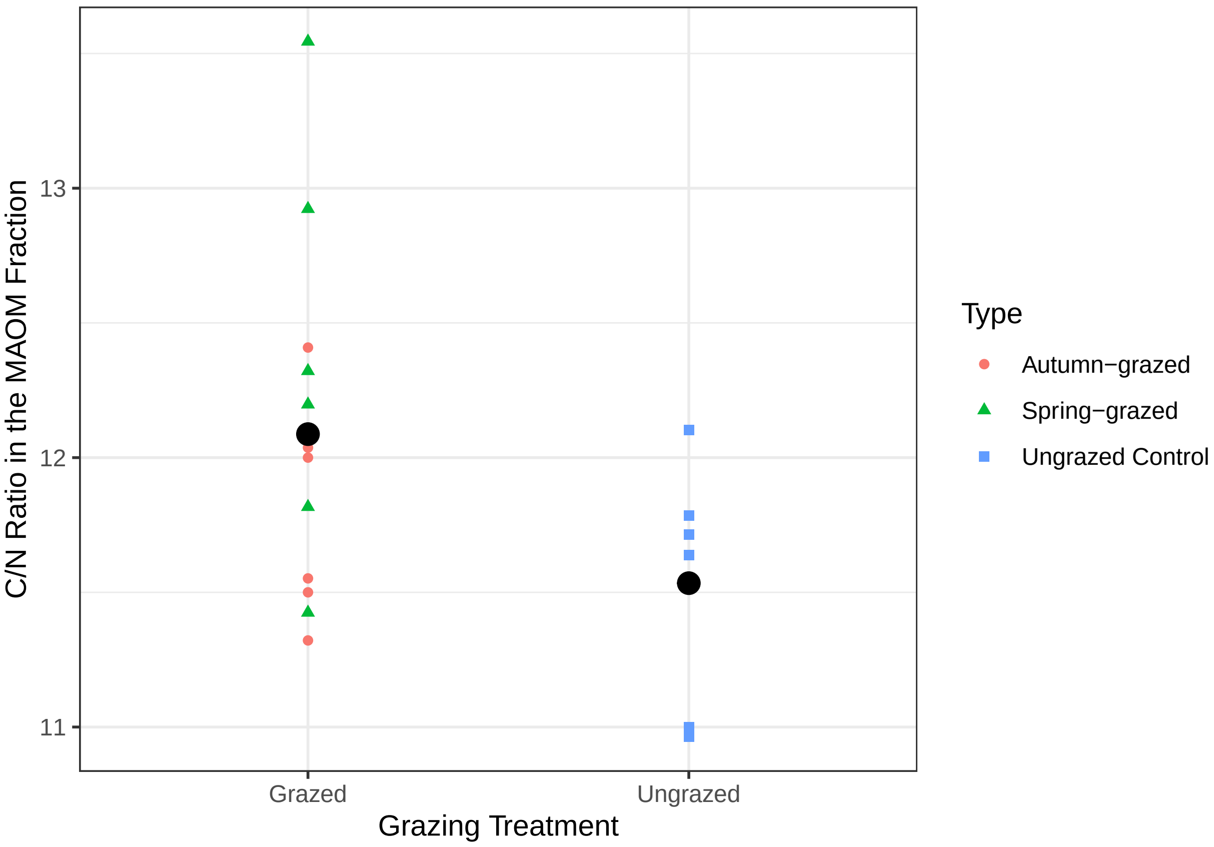


```
## This is the one to use
ggplot(MAOMRatio) +  geom_point(mapping = aes(x=Type, y=yvar, shape = Type, col=Type)) + labs(x = "Grazi
```

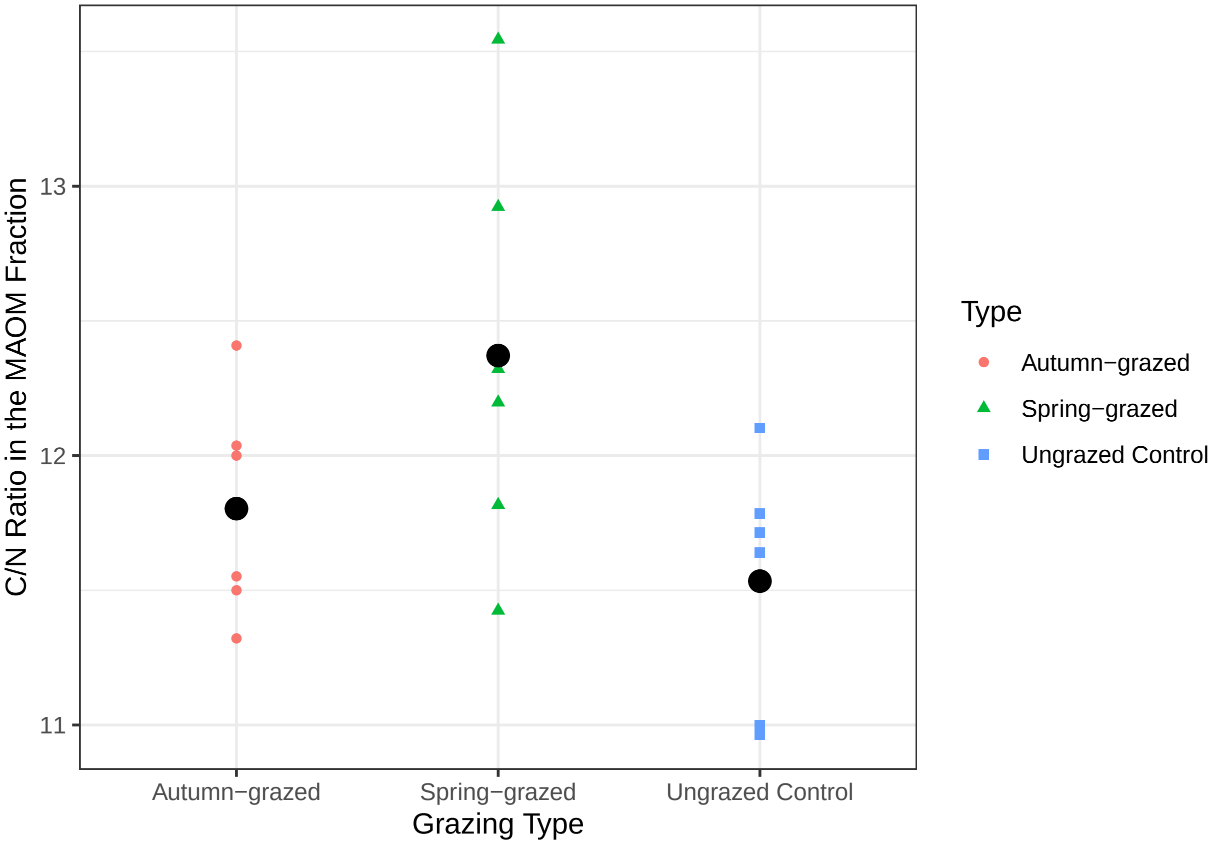


```r
## Adding confidence intervals
MAOMRatioSummary <- MAOMRatio %>%
  group_by(Type) %>%
  summarise(
    Mean = mean(MAOM.C.N.Ratio),
    SD = sd(MAOM.C.N.Ratio)
  )

# Base ggplot call with only the x aesthetic set globally, as it's common across all layers
ggplot(MAOMRatio, aes(x=Type)) +
  # First geom_point layer for individual observations, specifying y aesthetic individually
  geom_point(aes(y=MAOM.C.N.Ratio, shape=Type, color=Type)) +
  labs(x = "Grazing Type", y = "MAOM C:N Ratio") +
  # Second geom_point layer for means from MAOMRatioSummary, specifying y aesthetic for Mean
  geom_point(data=MAOMRatioSummary, aes(x=Type, y=Mean), size=3.5, color="black", alpha=1) +
  # geom_errorbar layer for error bars, using MAOMstockSummary and specifying ymin and ymax based on Me
  geom_errorbar(data=MAOMRatioSummary, aes(x=Type, ymin=Mean-SD, ymax=Mean+SD), width=.05, color="black
```

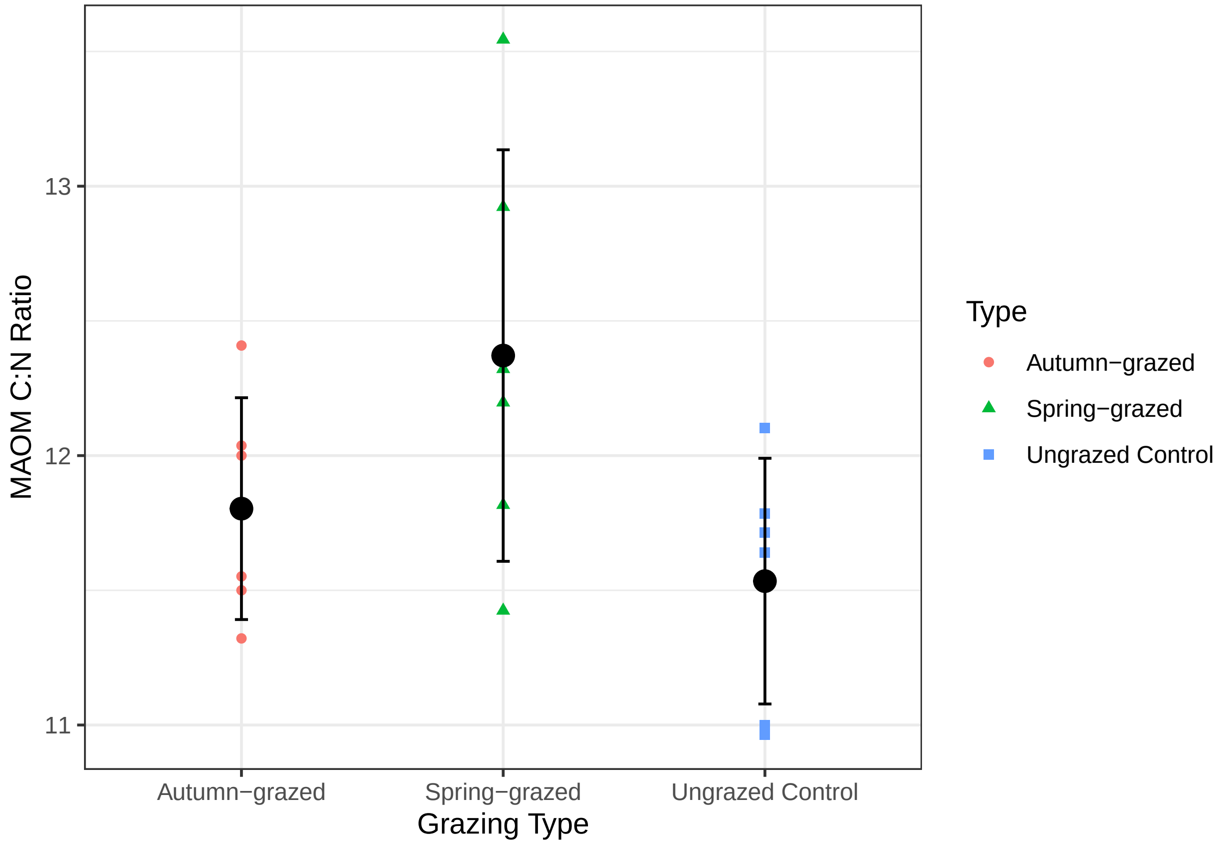


```r
### Adding confidence interval error bars
MAOMRatioSummary <- MAOMRatio %>%
  group_by(Type) %>%
  summarise(
    Mean = mean(MAOM.C.N.Ratio),
    SD = sd(MAOM.C.N.Ratio),
    N = n(), # Calculate the sample size for each group
    SEM = SD / sqrt(N), # Calculate the Standard Error of the Mean (SEM)
    CI_Lower = Mean - qt(0.975, df=N-1) * SEM, # Calculate the lower bound of the 95% CI
    CI_Upper = Mean + qt(0.975, df=N-1) * SEM # Calculate the upper bound of the 95% CI
  )

# Base ggplot call with only the x aesthetic set globally, as it's common across all layers
ggplot(MAOMRatio, aes(x=Type)) +
  # First geom_point layer for individual observations, specifying y aesthetic individually
  geom_point(aes(y=MAOM.C.N.Ratio, shape=Type, color=Type)) +
  labs(x = "Grazing Type", y = "MAOM C:N Ratio") +
  # Second geom_point layer for means from MAOMRatioSummary, specifying y aesthetic for Mean
  geom_point(data=MAOMRatioSummary, aes(x=Type, y=Mean), size=3.5, color="black", alpha=1) +
  # geom_errorbar layer for 95% CI error bars, using MAOMRatioSummary and specifying ymin and ymax base
  geom_errorbar(data=MAOMRatioSummary, aes(x=Type, ymin=CI_Lower, ymax=CI_Upper), width=.05, color="bla
```

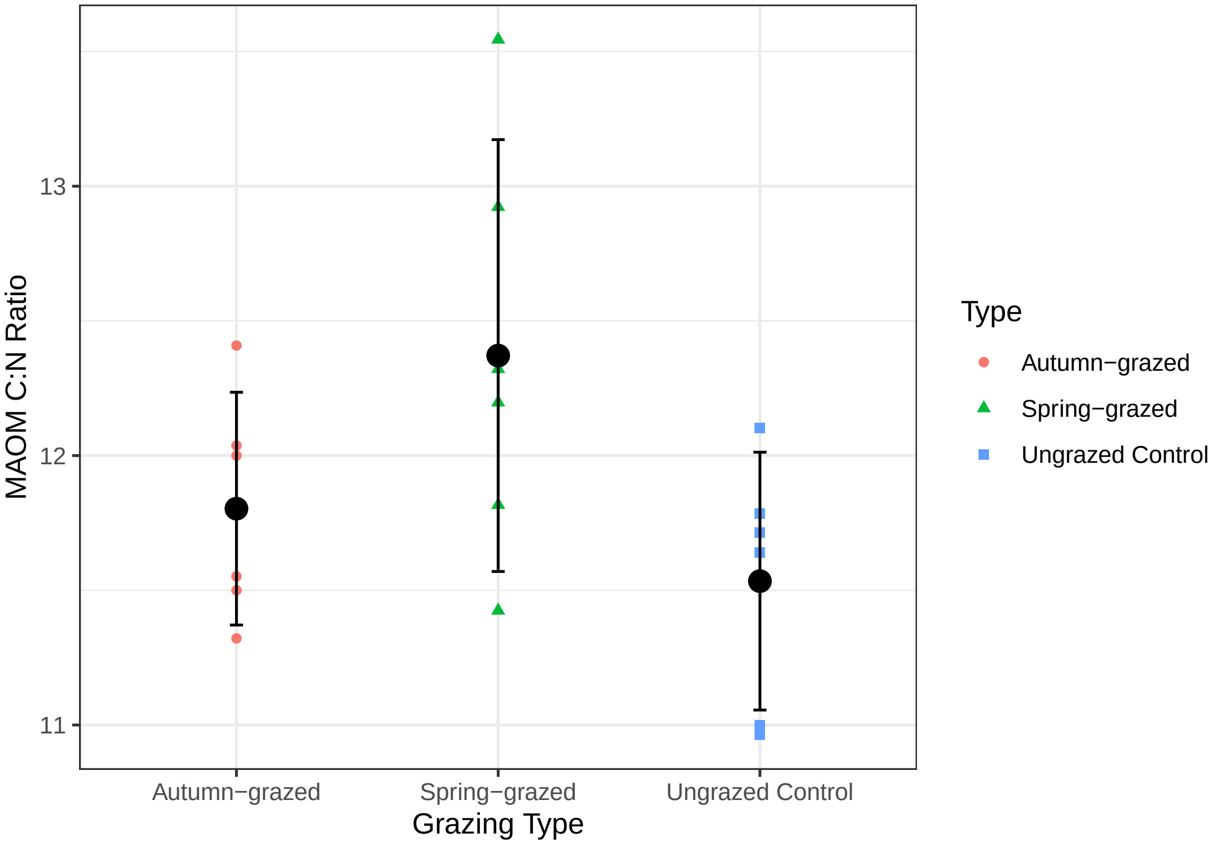


```
ggplot(MAOMRatio, aes(x=Type, y=MAOM.C.N.Ratio)) + geom_boxplot(trim=FALSE) + labs(x = "Grazing Type", y
```

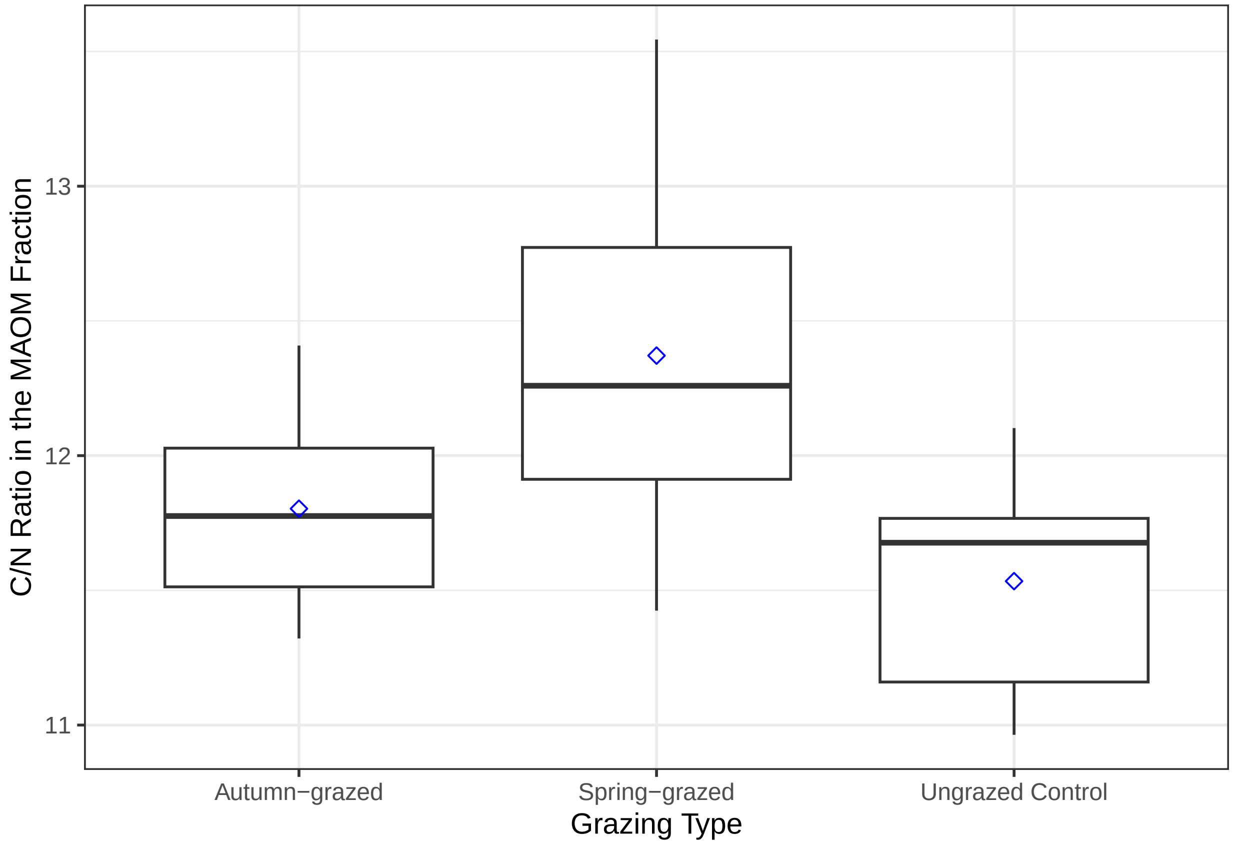


```
# Calculating 95% confidence intervals
UngrazedMAOMRatio <- MAOMRatio[c(1,2,3,10,11,12),]
1.96 * sd(UngrazedMAOMRatio$MAOM.C.N.Ratio)/sqrt(6)
```

```
## [1] 0.3648664
```

```
SpringMAOMRatio <- MAOMRatio[c(7,8,9,16,17,18),]
1.96 * sd(SpringMAOMRatio$MAOM.C.N.Ratio)/sqrt(6)
```

```
## [1] 0.611113
```

```
AutumnMAOMRatio <- MAOMRatio[c(4,5,6,13,14,15),]
1.96 * sd(AutumnMAOMRatio$MAOM.C.N.Ratio)/sqrt(6)
```

```
## [1] 0.3293725
```

```
GrazedMAOMRatio <- MAOMRatio[c(4,5,6,7,8,9,13,14,15,16,17,18),]
1.96 * sd(GrazedMAOMRatio$MAOM.C.N.Ratio)/sqrt(6)
```

```
## [1] 0.5248228
```

Visual conlcusions: Grazed treatment has higher mean C/N ratio in MAOM fraction than the ungrazed treatment. This seems largely driven by the higher MAOM C/N ratio in the spring-grazed treatment (i.e., type may have an effect).

Mixed Effects Model looking at the effects of just grazing type (Spring vs Autumn vs Ungrazed Control):

```
MAOMRatioModel1 <- lme(MAOM.C.N.Ratio ~ Type, random = ~ 1|Block, data = MAOMRatio)
summary(MAOMRatioModel1)
```

```
## Linear mixed-effects model fit by REML
```

```
##   Data: MAOMRatio
##        AIC      BIC    logLik
##   37.42111 40.96136 -13.71056
##
## Random effects:
##  Formula: ~1 | Block
##         (Intercept)  Residual
## StdDev:   0.4038038 0.4715744
##
## Fixed effects:  MAOM.C.N.Ratio ~ Type
##                          Value Std.Error DF  t-value
## (Intercept)          11.803132 0.3443726 14 34.27430
## TypeSpring-grazed     0.568186 0.2722636 14  2.08690
## TypeUngrazed Control -0.269065 0.2722636 14 -0.98825
##                      p-value
## (Intercept)           0.0000
## TypeSpring-grazed     0.0557
## TypeUngrazed Control  0.3398
##  Correlation:
##                      (Intr) TypSp-
## TypeSpring-grazed    -0.395
## TypeUngrazed Control -0.395  0.500
##
## Standardized Within-Group Residuals:
##        Min         Q1        Med         Q3        Max
## -1.7395508 -0.5405149 -0.0503811  0.6319503  1.9231386
##
## Number of Observations: 18
## Number of Groups: 2
```

```
anova(MAOMRatioModel1)
```

```
##             numDF denDF   F-value p-value
## (Intercept)     1    14 1509.0811  <.0001
## Type            2    14    4.9294   0.024
```

```
plot(MAOMRatioModel1)
```

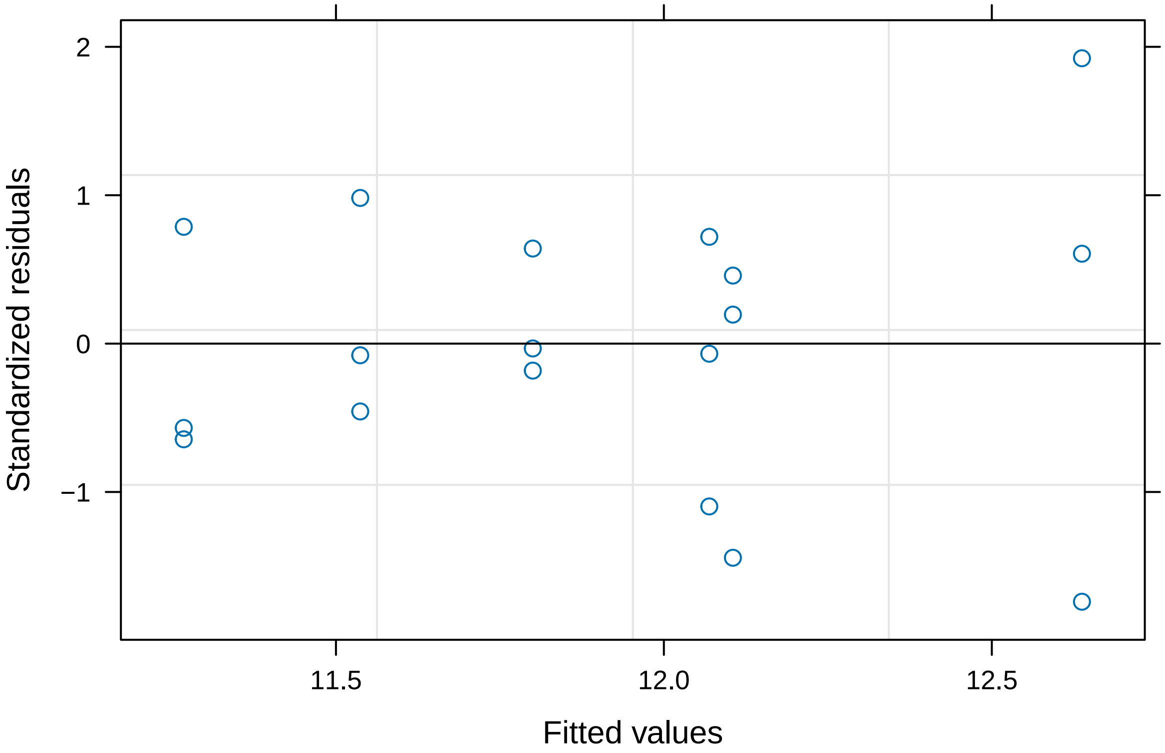


```r
qqnorm(MAOMRatioModel1$residuals)
```

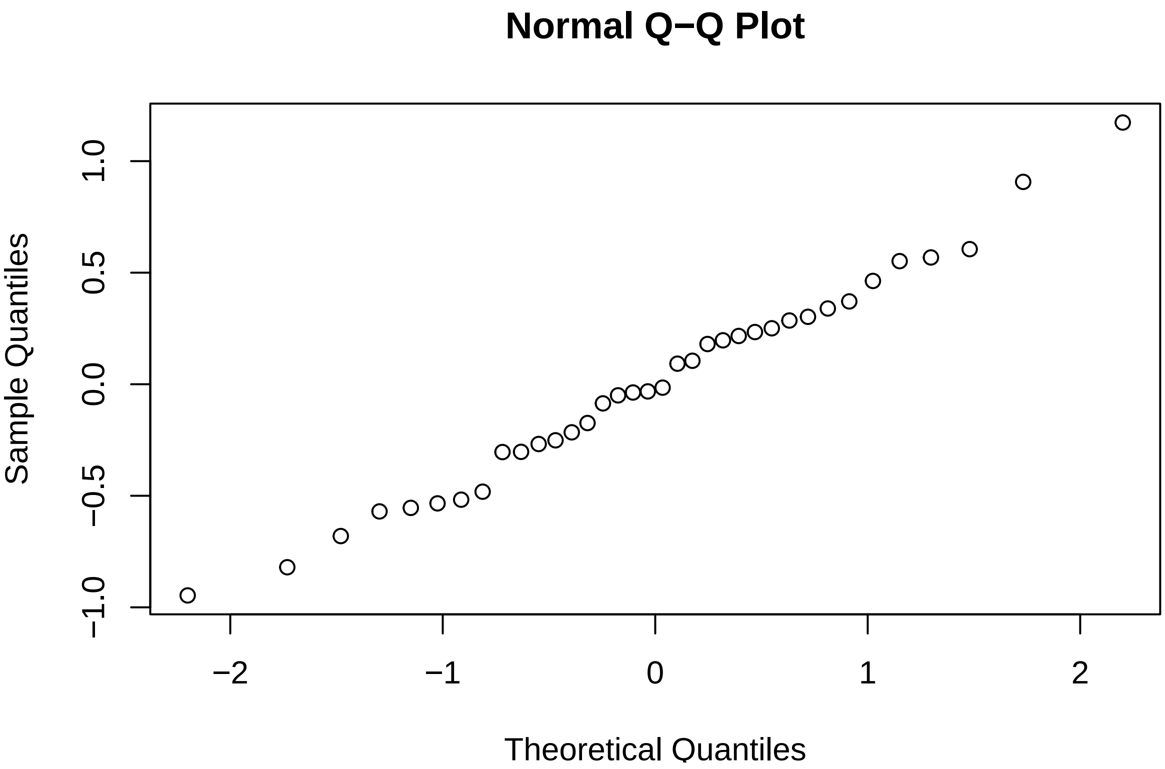


```r
## Changing order to get last comparison
levels(MAOMRatio$Type)
```

```
## NULL
```

```r
MAOMRatio$Type <- factor(MAOMRatio$Type, levels=c('Ungrazed Control', 'Autumn-grazed', 'Spring-grazed'))
MAOMRatioModel2 <- lme(MAOM.C.N.Ratio ~ Type, random = ~ 1|Block, data = MAOMRatio)
summary(MAOMRatioModel2)
```

```
## Linear mixed-effects model fit by REML
##   Data: MAOMRatio
##        AIC      BIC    logLik
##   37.42111 40.96136 -13.71056
##
## Random effects:
##  Formula: ~1 | Block
##         (Intercept)  Residual
## StdDev:   0.4038043 0.4715744
##
## Fixed effects:  MAOM.C.N.Ratio ~ Type
##                         Value Std.Error DF  t-value p-value
## (Intercept)         11.534067 0.3443729 14 33.49296  0.0000
## TypeAutumn-grazed    0.269065 0.2722636 14  0.98825  0.3398
## TypeSpring-grazed    0.837252 0.2722636 14  3.07515  0.0082
##  Correlation:
##                   (Intr) TypAt-
## TypeAutumn-grazed -0.395
## TypeSpring-grazed -0.395  0.500
##
## Standardized Within-Group Residuals:
##         Min          Q1         Med          Q3
## -1.73955108 -0.54051475 -0.05038128  0.63195015
##         Max
##  1.92313854
##
## Number of Observations: 18
## Number of Groups: 2
```

```r
anova(MAOMRatioModel2)
```

```
##             numDF denDF   F-value p-value
## (Intercept)     1    14 1509.0781  <.0001
## Type            2    14    4.9294   0.024
```

```r
plot(MAOMRatioModel2)
```

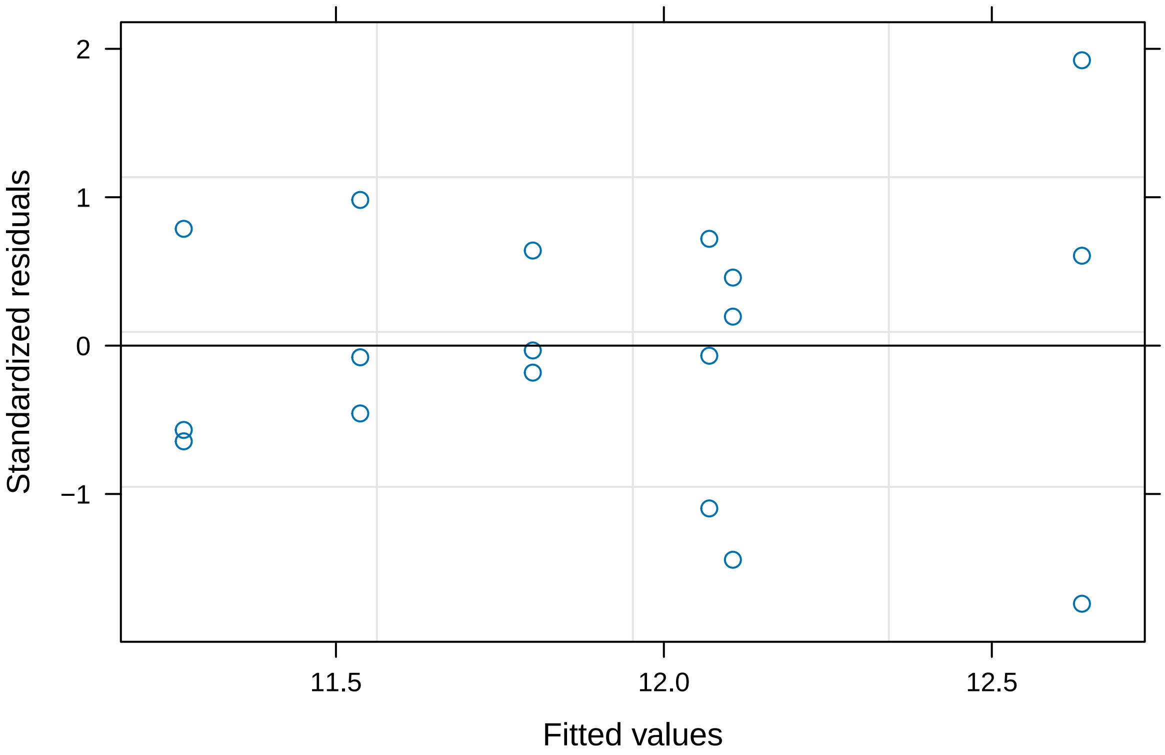


```r
qqnorm(MAOMRatioModel2$residuals)
```

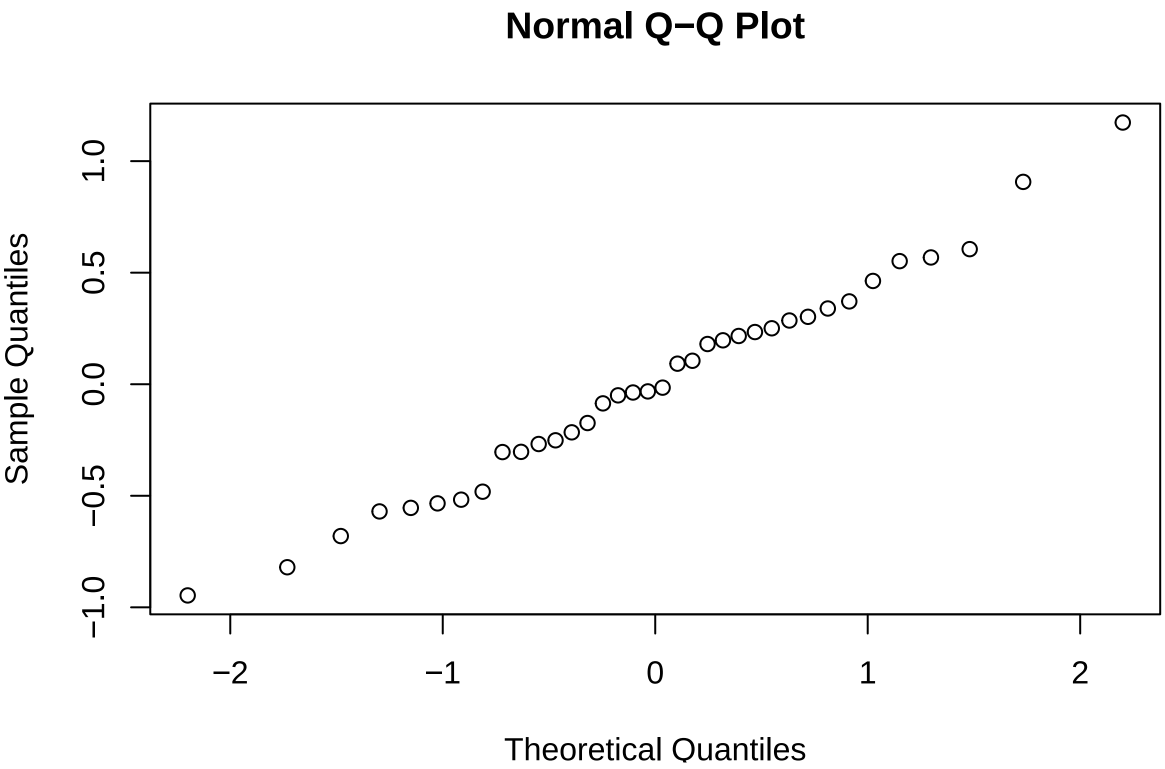

Meets assumptions fine. Overall, grazing type has a significant effect on C/N MAOM ratio ($F2,14 = 4.93$, $p = 0.024$) The spring-grazed treatment produces the highest C/N ratio in the MAOM fraction, significantly greater than the ungrazed control ($t14 = 3.08$, $p = 0.008$) and weakly significantly greater than the autumn-grazed paddocks ($t14 = 2.09$, $p = 0.056$). In contrast, the autumn-grazed and ungrazed control paddocks do not

differ from each other in the C/N ratio in the MAOM fraction ($t14 = 0.98$, $p = 0.340$).

Mixed effects model looking at the effect of just Treatment (grazed vs ungrazed):

```
MAOMRatioModel3 <- lme(MAOM.C.N.Ratio ~ Treatment, random = ~ 1|Block, data = MAOMRatio)
summary(MAOMRatioModel3)
```

```
## Linear mixed-effects model fit by REML
##   Data: MAOMRatio
##        AIC      BIC    logLik
##   38.68491 41.77526 -15.34245
##
## Random effects:
##  Formula: ~1 | Block
##         (Intercept)  Residual
## StdDev:   0.3969006 0.5216551
##
## Fixed effects:  MAOM.C.N.Ratio ~ Treatment
##                       Value Std.Error DF  t-value p-value
## (Intercept)       12.087225 0.3184997 15 37.95051   0.000
## TreatmentUngrazed -0.553158 0.2608276 15 -2.12078   0.051
##  Correlation:
##                   (Intr)
## TreatmentUngrazed -0.273
##
## Standardized Within-Group Residuals:
##         Min          Q1         Med          Q3
## -1.51932674 -0.62475726 -0.07971231  0.66981980
##         Max
##  2.30039919
##
## Number of Observations: 18
## Number of Groups: 2
```

```
anova(MAOMRatioModel3)
```

```
##             numDF denDF   F-value p-value
## (Intercept)     1    15 1509.0856  <.0001
## Treatment       1    15    4.4977   0.051
```

```
plot(MAOMRatioModel3)
```

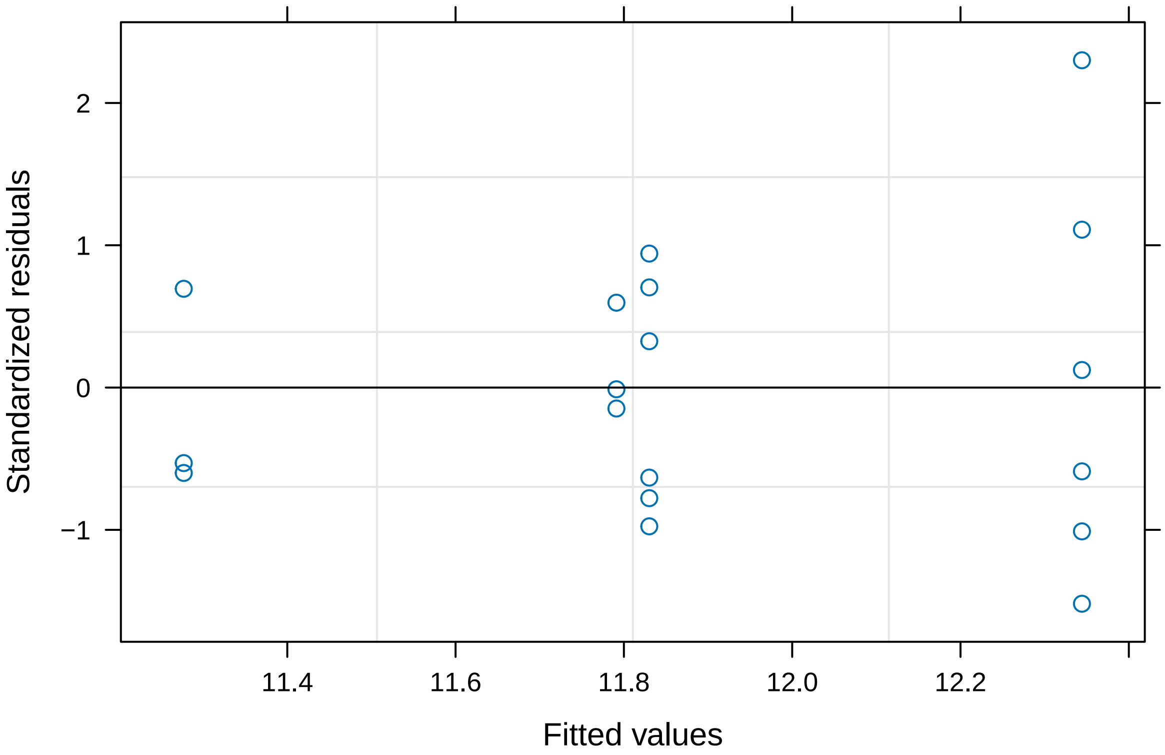


```r
qqnorm(MAOMRatioModel3$residuals)
```

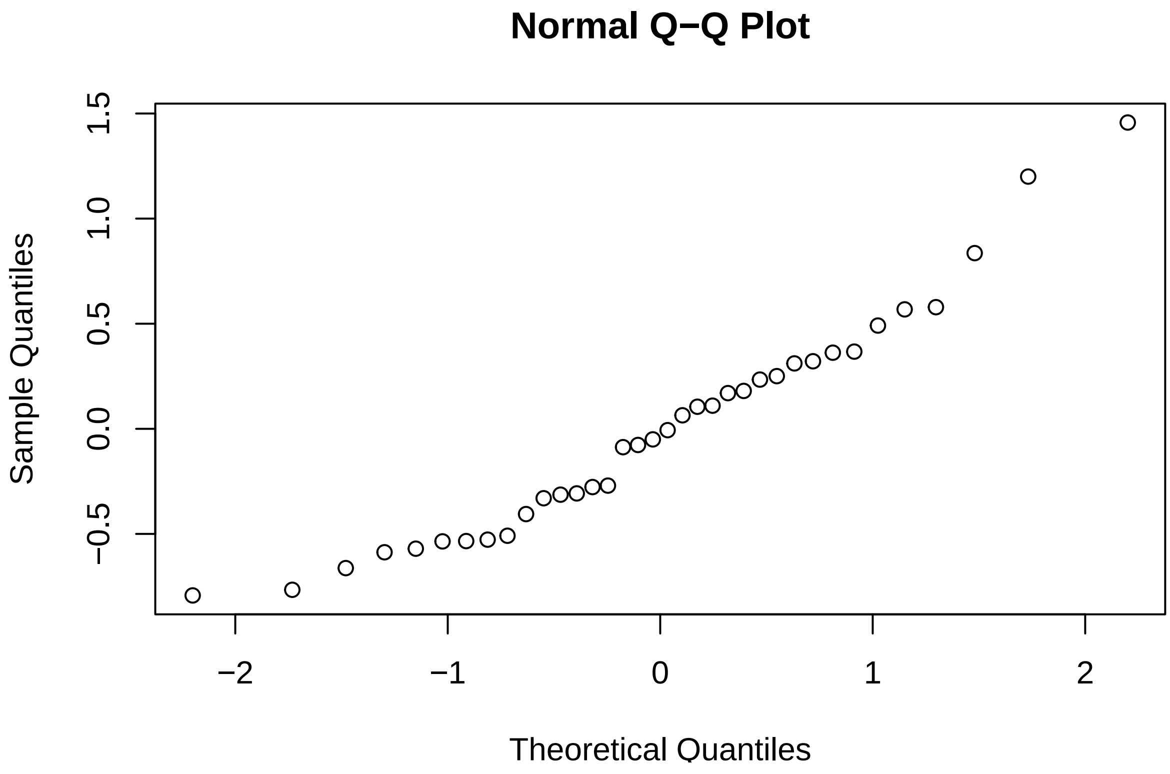


```r
MAOMRatioModel4 <- lme(sqrt(MAOM.C.N.Ratio) ~ Treatment, random = ~ 1|Block, data = MAOMRatio)
summary(MAOMRatioModel4)
```

```
## Linear mixed-effects model fit by REML
```

```
##   Data: MAOMRatio
##         AIC       BIC   logLik
##   -23.55456 -20.46421 15.77728
##
## Random effects:
##  Formula: ~1 | Block
##         (Intercept)   Residual
## StdDev:   0.0573706 0.07454648
##
## Fixed effects:  sqrt(MAOM.C.N.Ratio) ~ Treatment
##                        Value  Std.Error DF  t-value
## (Intercept)         3.475520 0.04592157 15 75.68381
## TreatmentUngrazed -0.079892 0.03727324 15 -2.14341
##                    p-value
## (Intercept)         0.0000
## TreatmentUngrazed  0.0489
##  Correlation:
##                   (Intr)
## TreatmentUngrazed -0.271
##
## Standardized Within-Group Residuals:
##         Min          Q1         Med          Q3
## -1.52874147 -0.63323607 -0.06835704  0.68964548
##         Max
##  2.24710322
##
## Number of Observations: 18
## Number of Groups: 2
```

```
anova(MAOMRatioModel4)
```

```
##             numDF denDF  F-value p-value
## (Intercept)     1    15 6086.105  <.0001
## Treatment       1    15    4.594  0.0489
```

```
plot(MAOMRatioModel4)
```

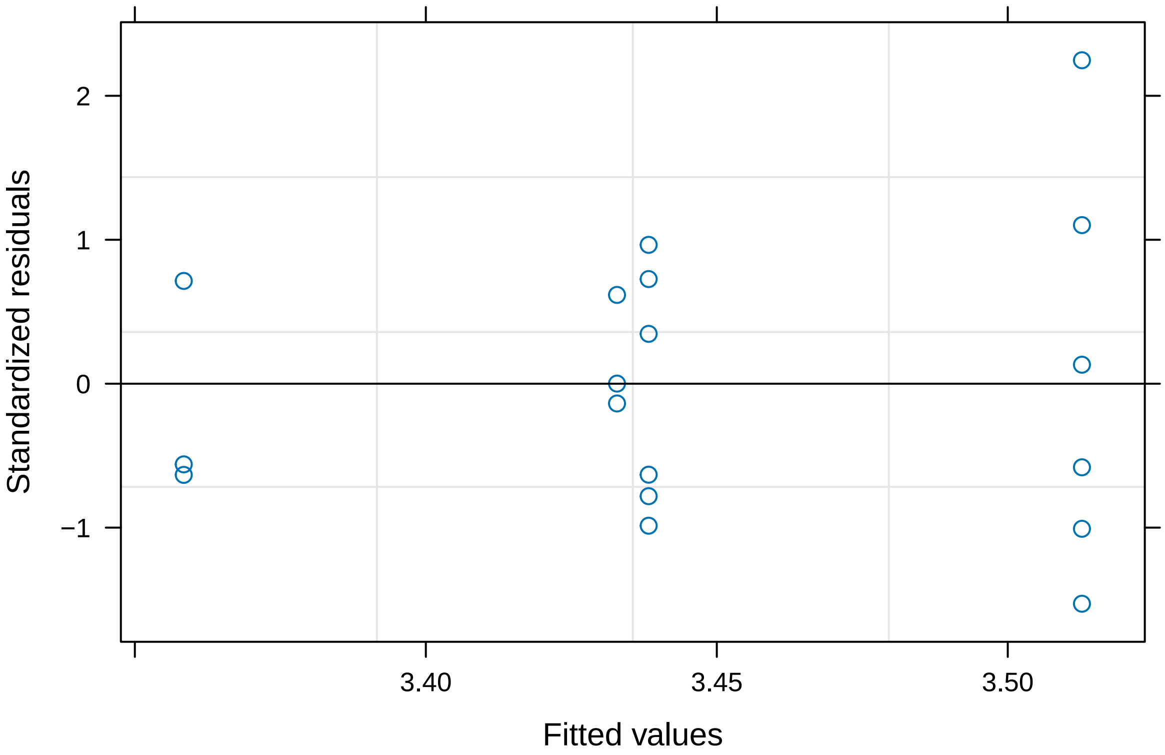


```
qqnorm(MAOMRatioModel4$residuals)
```

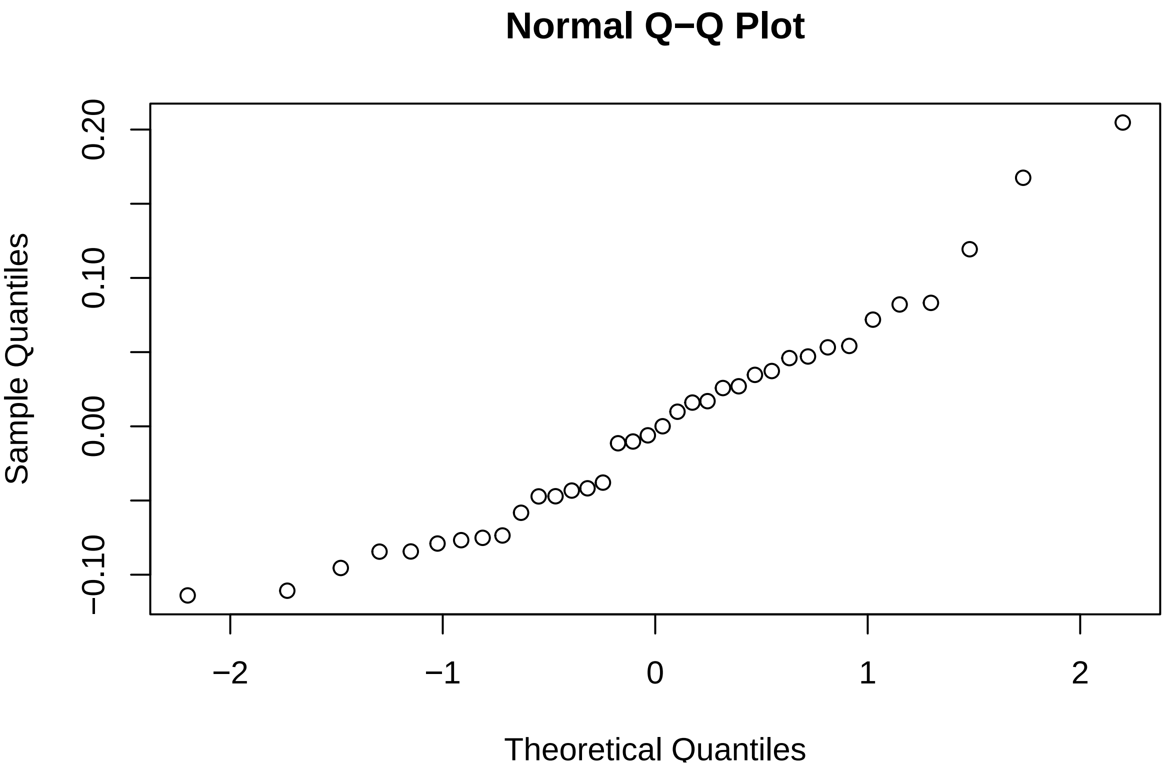


```
MAOMRatioModel5 <- lme(log(MAOM.C.N.Ratio) ~ Treatment, random = ~ 1|Block, data = MAOMRatio)
summary(MAOMRatioModel5)
```

```
## Linear mixed-effects model fit by REML
```

```
##   Data: MAOMRatio
##         AIC       BIC   logLik
##   -41.39693 -38.30657 24.69846
##
## Random effects:
##  Formula: ~1 | Block
##         (Intercept)   Residual
## StdDev:    0.033191 0.04266055
##
## Fixed effects:  log(MAOM.C.N.Ratio) ~ Treatment
##                        Value  Std.Error DF  t-value
## (Intercept)        2.4908353 0.02650436 15 93.97831
## TreatmentUngrazed -0.0461871 0.02133027 15 -2.16533
##                   p-value
## (Intercept)        0.0000
## TreatmentUngrazed  0.0469
##  Correlation:
##                   (Intr)
## TreatmentUngrazed -0.268
##
## Standardized Within-Group Residuals:
##         Min          Q1         Med          Q3
## -1.53710294 -0.65827258 -0.05651659  0.70953122
##         Max
##  2.19307348
##
## Number of Observations: 18
## Number of Groups: 2
```

```r
anova(MAOMRatioModel5)
```

```
##             numDF denDF  F-value p-value
## (Intercept)     1    15 9399.507  <.0001
## Treatment       1    15    4.689  0.0469
```

```r
plot(MAOMRatioModel5)
```

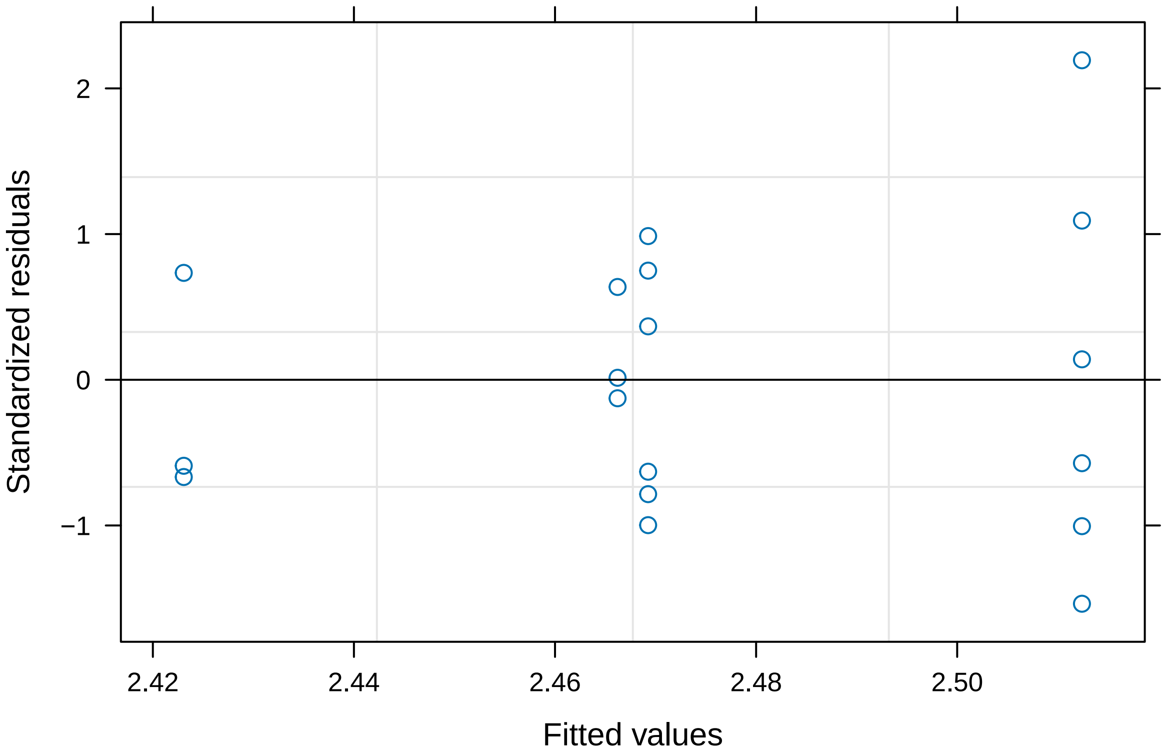


```r
qqnorm(MAOMRatioModel5$residuals)
```

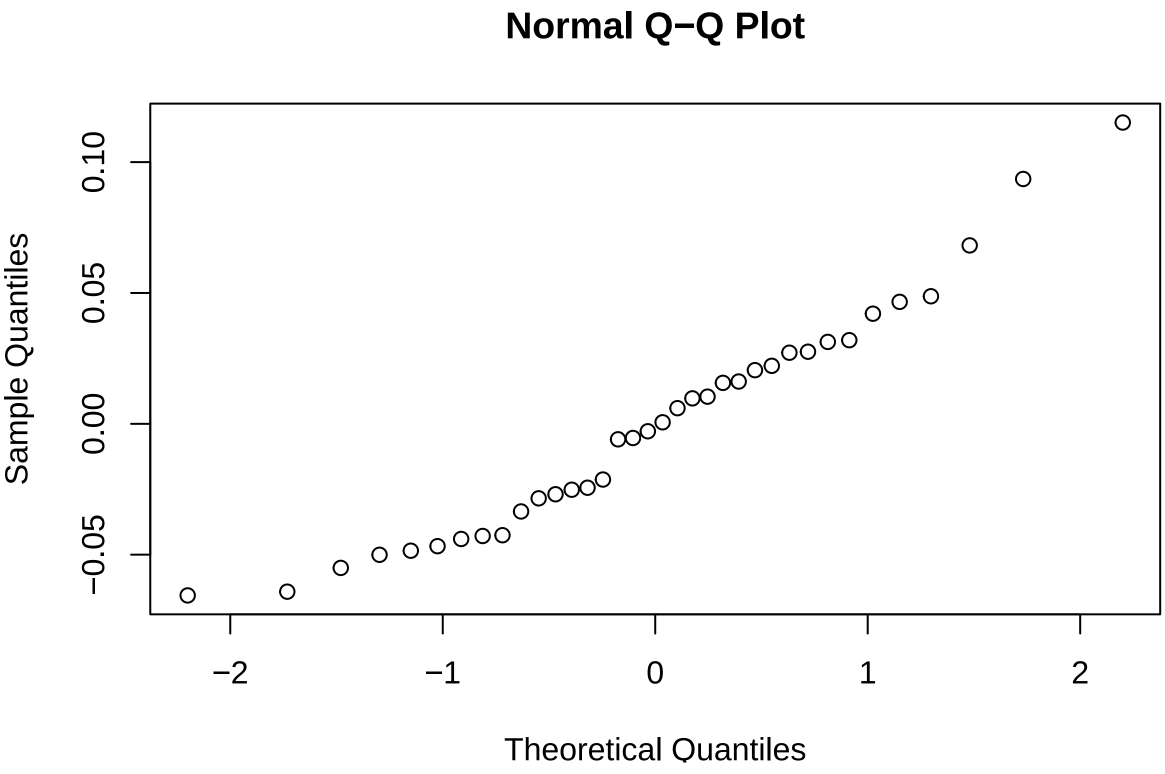

When using the log-transformed data (best option), the model suggests that grazing has a significant positive effect on MAOM C/N ratio ($F1,15 = 4.69$, $p = 0.047$). Though, as shown above, this masks an important distinction between spring-grazed and autumn-grazed paddocks.

Part 3 (Section 2) - POM C/N Ratio Visualization:

```
POMRatio <- completedata2 %>% dplyr::select(Block, ID, Type, Treatment, Study, POM.C.N.Ratio)
yvar <- POMRatio$POM.C.N.Ratio

POMRatioTreatment_means <- POMRatio %>% group_by(Treatment) %>% summarise(Treatment_mean_POM.C.N.Ratio =
POMRatioTreatment_means
```

```
## # A tibble: 2 x 2
##   Treatment Treatment_mean_POM.C.N.Ratio
##   <chr>                            <dbl>
## 1 Grazed                            49.8
## 2 Ungrazed                          47.8
```

```
POMRatioType_means <- POMRatio %>% group_by(Type) %>% summarise(Type_mean_POM.C.N.Ratio = mean(POM.C.N.R
POMRatioType_means
```

```
## # A tibble: 3 x 2
##   Type             Type_mean_POM.C.N.Ratio
##   <chr>                              <dbl>
## 1 Autumn-grazed                       47.9
## 2 Spring-grazed                       51.6
## 3 Ungrazed Control                    47.8
```

```
ggplot(POMRatio) +  geom_point(mapping = aes(x=Treatment, y=yvar, shape = Type, col=Type)) + labs(x = "G
```

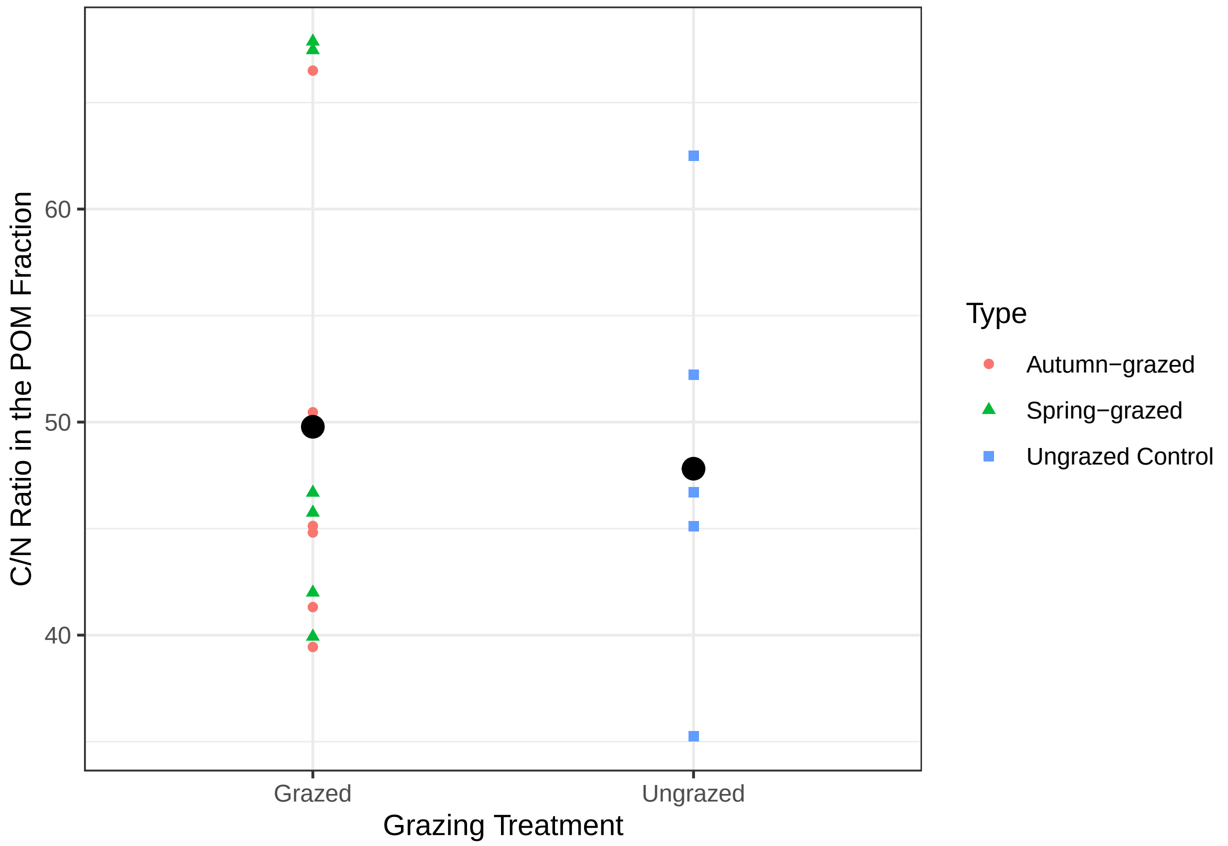


```
ggplot(POMRatio, aes(x=Type, y=POM.C.N.Ratio)) + geom_boxplot(trim=FALSE) + labs(x = "Grazing Type", y =
```

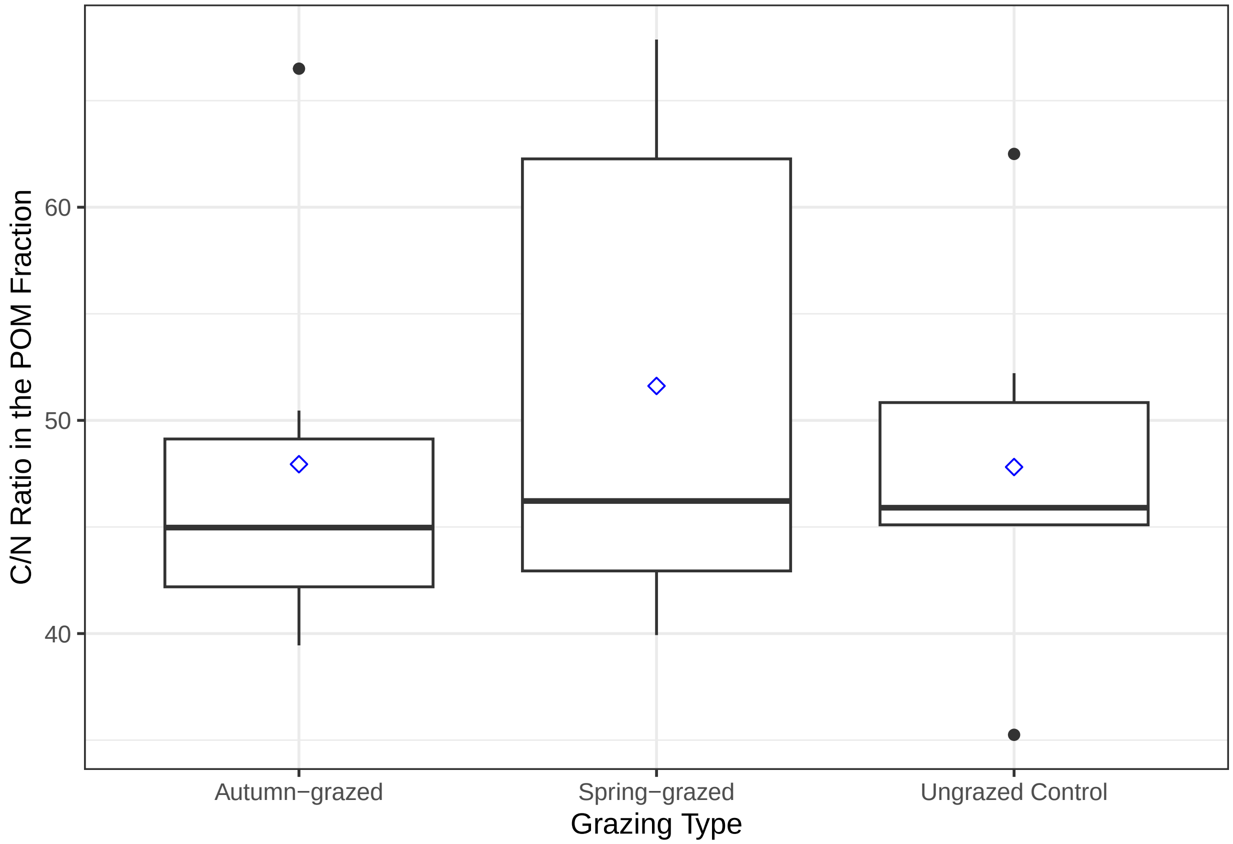


```
# Calculating 95% confidence intervals
UngrazedPOMRatio <- POMRatio[c(1,2,3,10,11,12),]
1.96 * sd(UngrazedPOMRatio$POM.C.N.Ratio)/sqrt(6)
```

```
## [1] 7.23569
```

```
SpringPOMRatio <- POMRatio[c(7,8,9,16,17,18),]
1.96 * sd(SpringPOMRatio$POM.C.N.Ratio)/sqrt(6)
```

```
## [1] 10.13991
```

```
AutumnPOMRatio <- POMRatio[c(4,5,6,13,14,15),]
1.96 * sd(AutumnPOMRatio$POM.C.N.Ratio)/sqrt(6)
```

```
## [1] 7.877122
```

```
GrazedPOMRatio <- POMRatio[c(4,5,6,7,8,9,13,14,15,16,17,18),]
1.96 * sd(GrazedPOMRatio$POM.C.N.Ratio)/sqrt(6)
```

```
## [1] 8.791663
```

Visual conclusions: POM C/N ratio is slightly higher in the grazed treatment than in the ungrazed (unlikely to be significant). Grazing type doesn't seem to have much of an effect. Though worth noting the variability is much greater in the spring-grazed than in the autumn-grazed or ungrazed control.

Mixed Effects Model looking at the effects of just grazing type (Spring vs Autumn vs Ungrazed Control):

```
POMRatioModel1 <- lme(POM.C.N.Ratio ~ Type, random = ~ 1|Block, data = POMRatio)
summary(POMRatioModel1)
```

```
## Linear mixed-effects model fit by REML
```

```
##   Data: POMRatio
##        AIC      BIC    logLik
##   128.8661 132.4064 -59.43305
##
## Random effects:
##  Formula: ~1 | Block
##          (Intercept) Residual
## StdDev: 0.0007090891 10.63435
##
## Fixed effects:  POM.C.N.Ratio ~ Type
##                            Value Std.Error DF   t-value
## (Intercept)             47.94416  4.341454 14 11.043343
## TypeSpring-grazed        3.67155  6.139743 14  0.597998
## TypeUngrazed Control    -0.13246  6.139743 14 -0.021575
##                         p-value
## (Intercept)              0.0000
## TypeSpring-grazed        0.5594
## TypeUngrazed Control     0.9831
##  Correlation:
##                         (Intr) TypSp-
## TypeSpring-grazed       -0.707
## TypeUngrazed Control    -0.707  0.500
##
## Standardized Within-Group Residuals:
##        Min          Q1         Med          Q3         Max
## -1.1812381 -0.6053692 -0.2600467  0.3696784  1.7448972
##
## Number of Observations: 18
## Number of Groups: 2
```

```r
anova(POMRatioModel1)
```

```
##             numDF denDF  F-value p-value
## (Intercept)     1    14 384.0925  <.0001
## Type            2    14   0.2473  0.7842
```

```r
plot(POMRatioModel1)
```

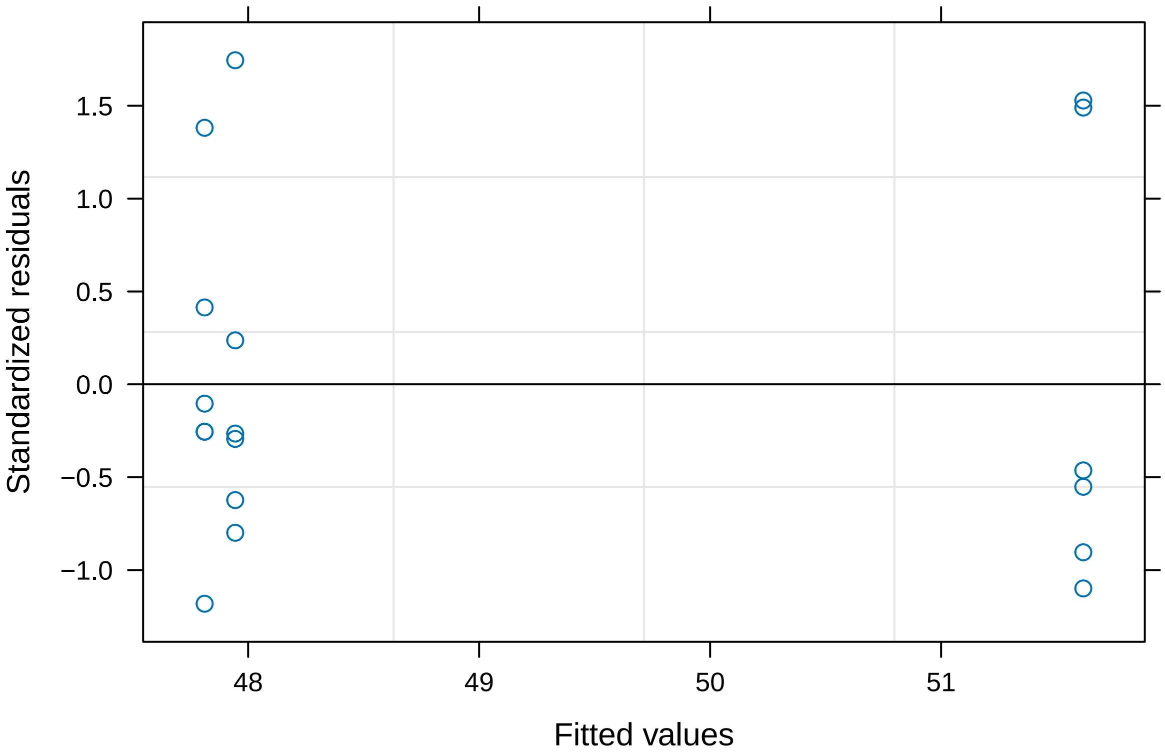


```r
qqnorm(POMRatioModel1$residuals)
```

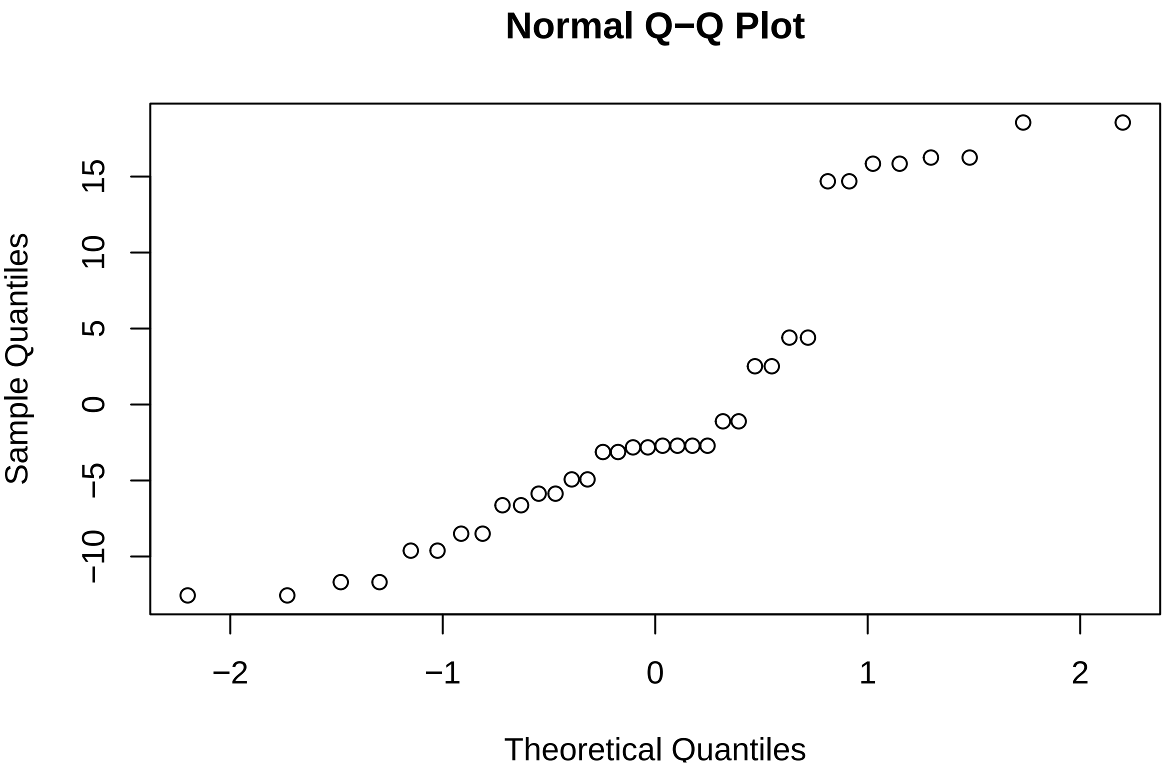


```r
## Changing order to get last comparison
levels(POMRatio$Type)
```

```
## NULL
```

```r
POMRatio$Type <- factor(POMRatio$Type, levels=c('Ungrazed Control','Autumn-grazed', 'Spring-grazed'))
POMRatioModel2 <- lme(POM.C.N.Ratio ~ Type, random = ~ 1|Block, data = POMRatio)
summary(POMRatioModel2)
```

```
## Linear mixed-effects model fit by REML
##   Data: POMRatio
##        AIC      BIC    logLik
##   128.8661 132.4064 -59.43305
##
## Random effects:
##  Formula: ~1 | Block
##         (Intercept) Residual
## StdDev: 0.0007090986 10.63435
##
## Fixed effects:  POM.C.N.Ratio ~ Type
##                       Value Std.Error DF   t-value p-value
## (Intercept)        47.81169  4.341454 14 11.012831  0.0000
## TypeAutumn-grazed  0.13246  6.139743 14  0.021575  0.9831
## TypeSpring-grazed  3.80402  6.139743 14  0.619573  0.5455
##  Correlation:
##                   (Intr) TypAt-
## TypeAutumn-grazed -0.707
## TypeSpring-grazed -0.707  0.500
##
## Standardized Within-Group Residuals:
##        Min         Q1        Med         Q3        Max
## -1.1812381 -0.6053692 -0.2600467  0.3696784  1.7448972
##
## Number of Observations: 18
## Number of Groups: 2
```

```r
anova(POMRatioModel2)
```

```
##             numDF denDF  F-value p-value
## (Intercept)     1    14 384.0925  <.0001
## Type            2    14   0.2473  0.7842
```

```r
plot(POMRatioModel2)
```

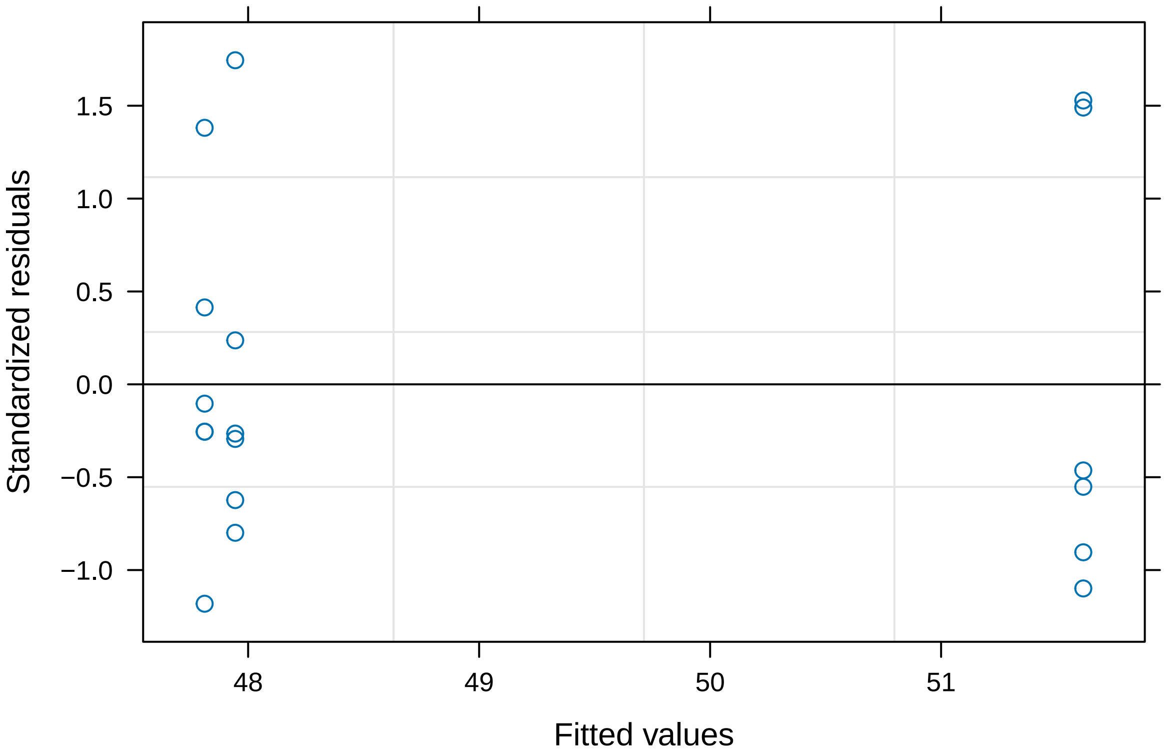


```
qqnorm(POMRatioModel2$residuals)
```

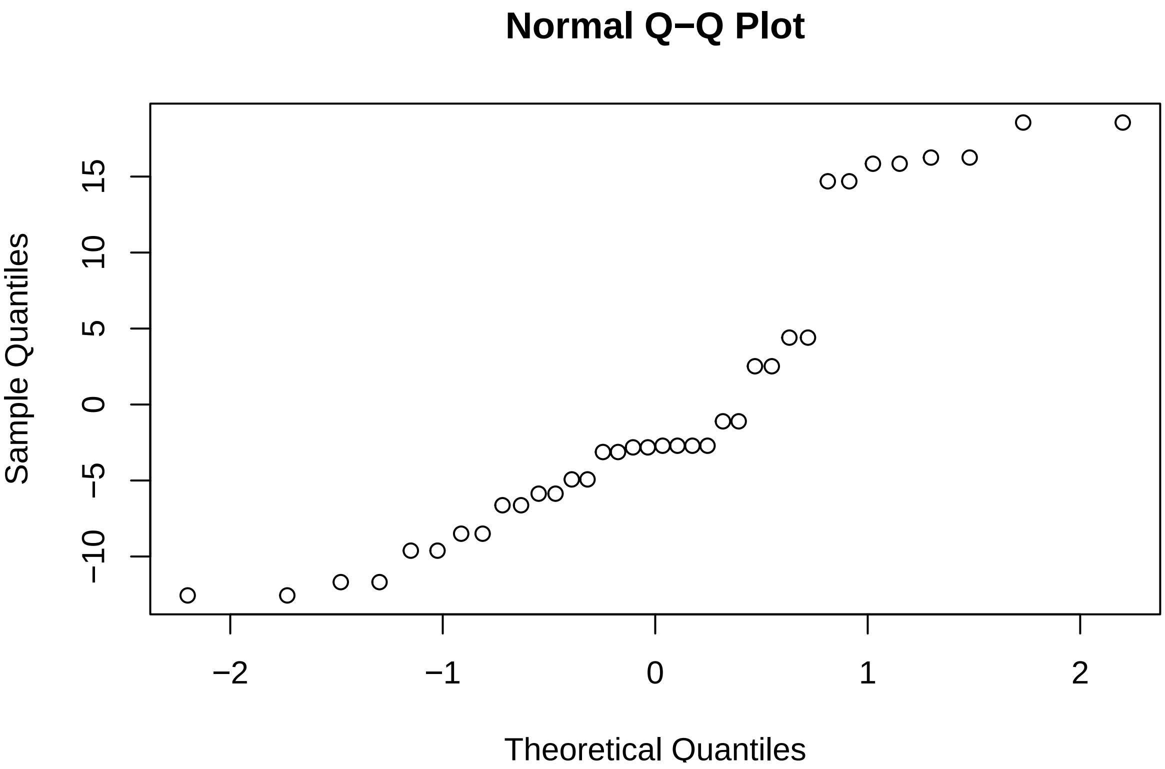

Meets assumptions fine. Suggests grazing type does not affect the C/N Ratio in the POM fraction (F2,14 = 0.247, p = 0.784).

Mixed effects model looking at the effect of just Treatment (grazed vs ungrazed):

```r
POMRatioModel3 <- lme(POM.C.N.Ratio ~ Treatment, random = ~ 1|Block, data = POMRatio)
summary(POMRatioModel3)
```

```
## Linear mixed-effects model fit by REML
##   Data: POMRatio
##        AIC      BIC    logLik
##   132.6779 135.7683 -62.33895
##
## Random effects:
##  Formula: ~1 | Block
##         (Intercept) Residual
## StdDev: 0.0007655182 10.41868
##
## Fixed effects:  POM.C.N.Ratio ~ Treatment
##                       Value Std.Error DF   t-value p-value
## (Intercept)        49.77994  3.007612 15 16.551313  0.0000
## TreatmentUngrazed -1.96824  5.209338 15 -0.377829  0.7109
##  Correlation:
##                   (Intr)
## TreatmentUngrazed -0.577
##
## Standardized Within-Group Residuals:
##        Min         Q1        Med         Q3        Max
## -1.2056902 -0.6791065 -0.2785446  0.3332808  1.7359914
##
## Number of Observations: 18
## Number of Groups: 2
```

```r
anova(POMRatioModel3)
```

```
##             numDF denDF  F-value p-value
## (Intercept)     1    15 400.1588  <.0001
## Treatment       1    15   0.1428  0.7109
```

```r
plot(POMRatioModel3)
```

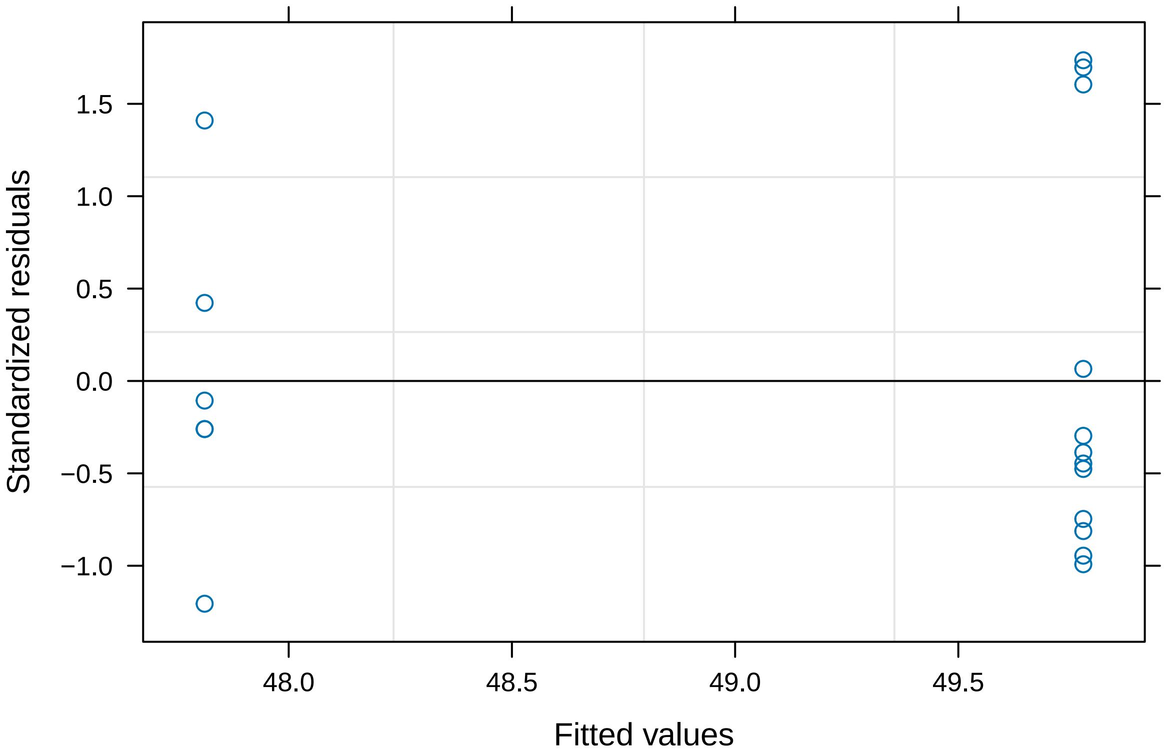


```r
qqnorm(POMRatioModel3$residuals)
```

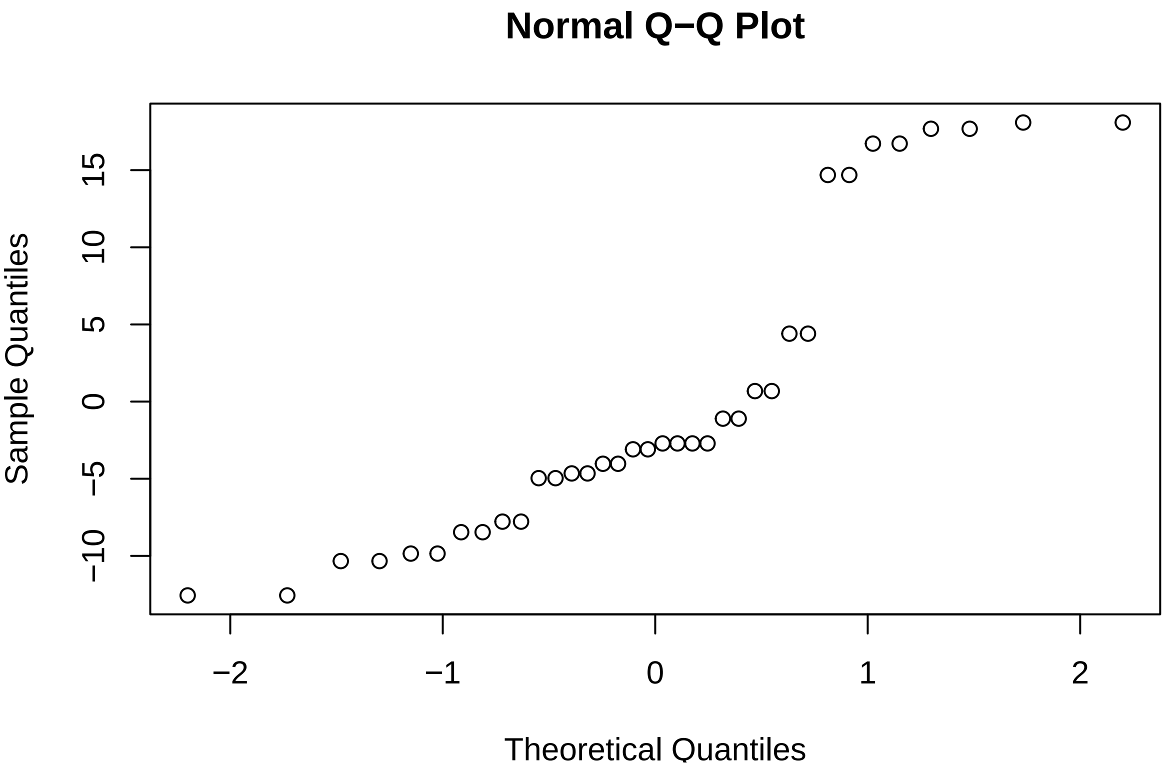

Meets assumptions fine. Suggests grazing does not influence the POM C/N ratio (F1,15 = 0.143, p = 0.711). This seems to be the accurate conclusion given that grazing type does not have a significant effect neither (above).

Part 3 (Section 3) - Total C/N Ratio

Visualization:

```
TotalRatio <- completedata2 %>% dplyr::select(Block, ID, Type, Treatment, Study, C.N.Ratio)
yvar <- TotalRatio$C.N.Ratio

TotalRatioTreatment_means <- TotalRatio %>% group_by(Treatment) %>% summarise(Treatment_mean_C.N.Ratio =
TotalRatioTreatment_means
```

```
## # A tibble: 2 x 2
##   Treatment Treatment_mean_C.N.Ratio
##   <chr>                        <dbl>
## 1 Grazed                        15.5
## 2 Ungrazed                      14.4
```

```
TotalRatioType_means <- TotalRatio %>% group_by(Type) %>% summarise(Type_mean_C.N.Ratio = mean(C.N.Ratio
TotalRatioType_means
```

```
## # A tibble: 3 x 2
##   Type             Type_mean_C.N.Ratio
##   <chr>                          <dbl>
## 1 Autumn-grazed                   14.7
## 2 Spring-grazed                   16.4
## 3 Ungrazed Control                14.4
```

```
ggplot(TotalRatio) +  geom_point(mapping = aes(x=Treatment, y=yvar, shape = Type, col=Type)) + labs(x =
```

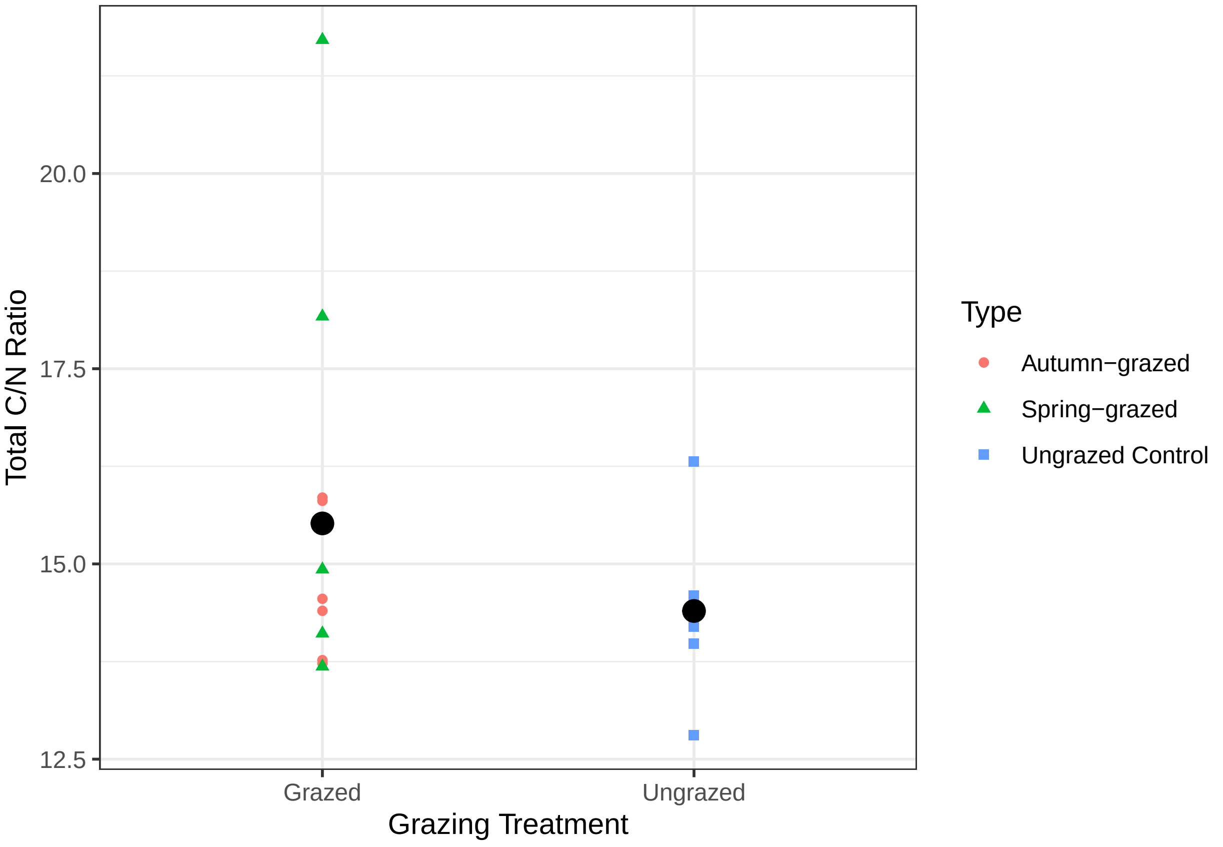

```
ggplot(TotalRatio, aes(x=Type, y=C.N.Ratio)) + geom_boxplot(trim=FALSE) + labs(x = "Grazing Type", y =
```

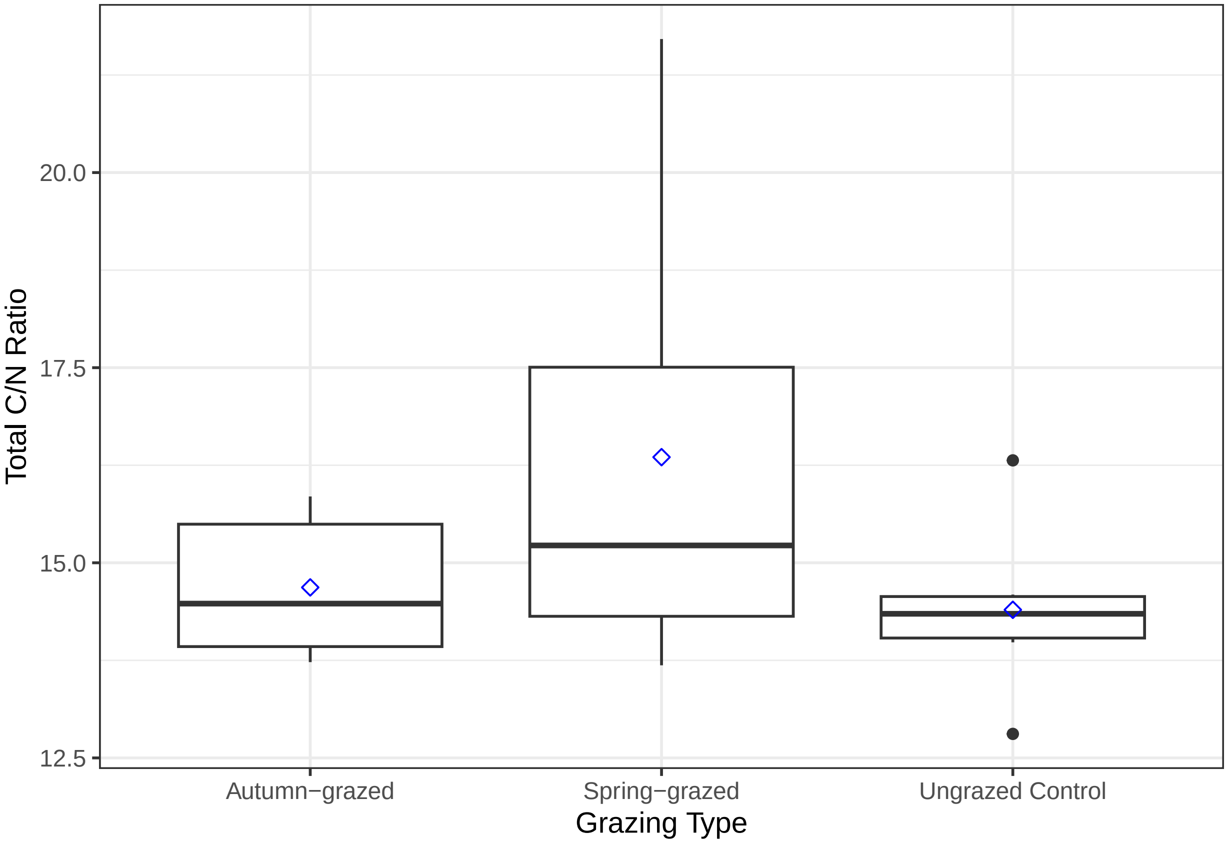


```
# Calculating 95% confidence intervals
UngrazedTotalRatio <- TotalRatio[c(1,2,3,10,11,12),]
1.96 * sd(UngrazedTotalRatio$C.N.Ratio)/sqrt(6)
```

```
## [1] 0.9091116
```

```
SpringTotalRatio <- TotalRatio[c(7,8,9,16,17,18),]
1.96 * sd(SpringTotalRatio$C.N.Ratio)/sqrt(6)
```

```
## [1] 2.450154
```

```
AutumnTotalRatio <- TotalRatio[c(4,5,6,13,14,15),]
1.96 * sd(AutumnTotalRatio$C.N.Ratio)/sqrt(6)
```

```
## [1] 0.7570092
```

```
GrazedTotalRatio <- TotalRatio[c(4,5,6,7,8,9,13,14,15,16,17,18),]
1.96 * sd(GrazedTotalRatio$C.N.Ratio)/sqrt(6)
```

```
## [1] 1.864298
```

Visual conclusions: Total C/N ratio higher in grazed than in ungrazed. Type may have an effect (spring-grazed has highest average total C/N ratio), but the spring-grazed also has more variability.

Mixed Effects Model looking at the effects of just grazing type (Spring vs Autumn vs Ungrazed Control):

```
TotalRatioModel1 <- lme(C.N.Ratio ~ Type, random = ~ 1|Block, data = TotalRatio)
summary(TotalRatioModel1)
```

```
## Linear mixed-effects model fit by REML
##   Data: TotalRatio
##        AIC      BIC    logLik
##   78.18018 81.72043 -34.09009
##
## Random effects:
##  Formula: ~1 | Block
##          (Intercept) Residual
## StdDev: 6.633168e-05 1.963166
##
## Fixed effects:  C.N.Ratio ~ Type
##                            Value Std.Error DF   t-value
## (Intercept)            14.684576  0.801459 14 18.322305
## TypeSpring-grazed       1.668937  1.133434 14  1.472460
## TypeUngrazed Control -0.286797  1.133434 14 -0.253034
##                      p-value
## (Intercept)           0.0000
## TypeSpring-grazed     0.1630
## TypeUngrazed Control  0.8039
##  Correlation:
##                      (Intr) TypSp-
## TypeSpring-grazed    -0.707
## TypeUngrazed Control -0.707  0.500
##
## Standardized Within-Group Residuals:
##        Min         Q1        Med         Q3        Max
## -1.3584924 -0.4824311 -0.1228511  0.4540810  2.7290611
##
## Number of Observations: 18
## Number of Groups: 2
```

```
anova(TotalRatioModel1)
```

```
##             numDF denDF   F-value p-value
## (Intercept)     1    14 1071.3066  <.0001
## Type            2    14    1.7365   0.212
```

```
plot(TotalRatioModel1)
```

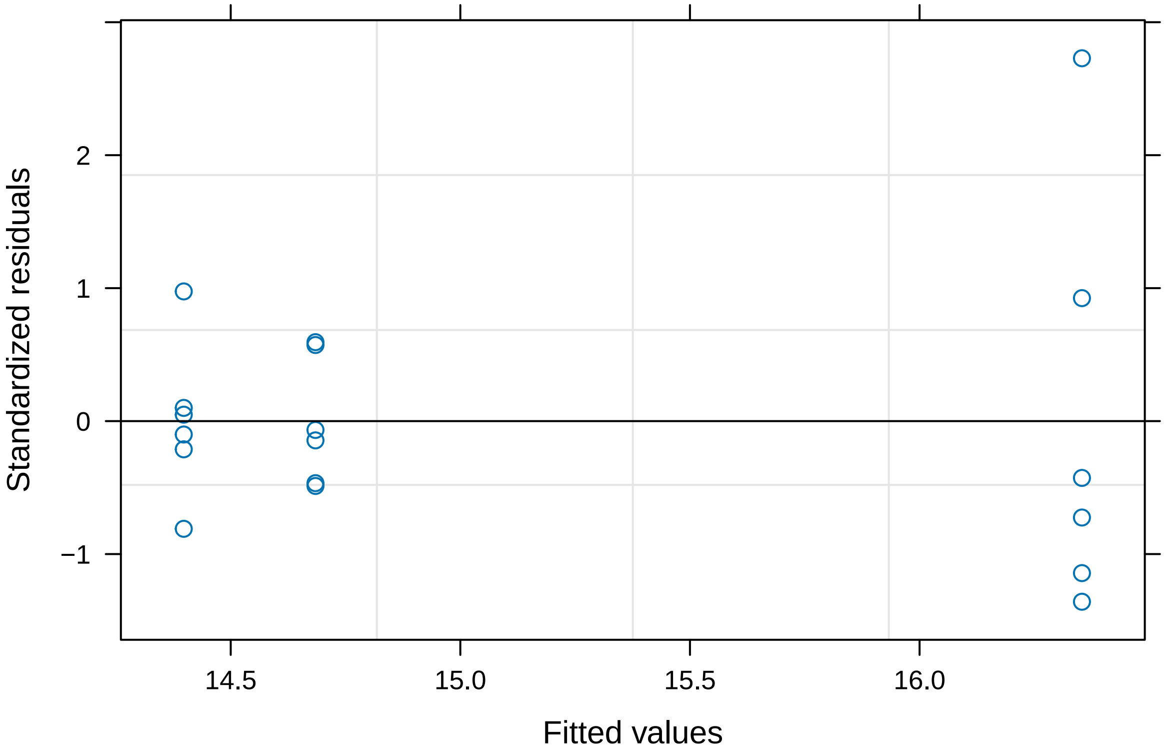


```r
qqnorm(TotalRatioModel1$residuals)
```

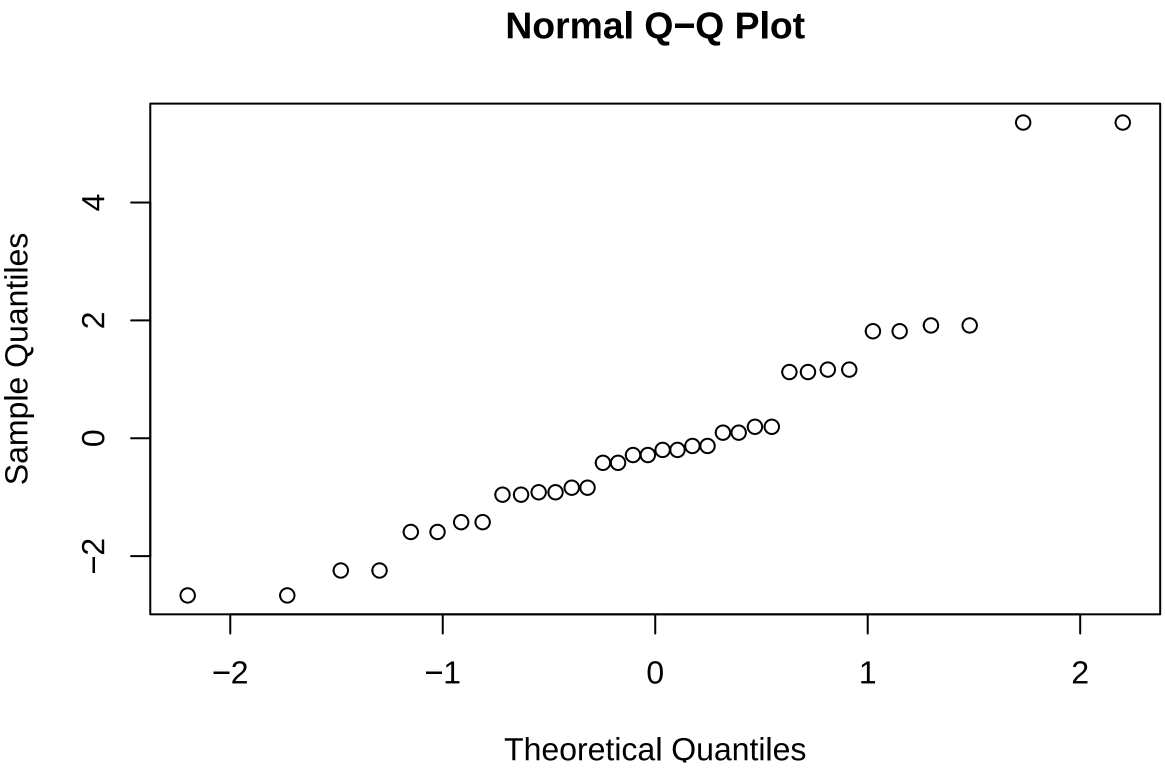


```r
TotalRatioModel1A <- lme(sqrt(C.N.Ratio) ~ Type, random = ~ 1|Block, data = TotalRatio)
summary(TotalRatioModel1A)
```

```
## Linear mixed-effects model fit by REML
```

```
##   Data: TotalRatio
##        AIC      BIC    logLik
##   15.00293 18.54318 -2.501463
##
## Random effects:
##  Formula: ~1 | Block
##          (Intercept) Residual
## StdDev: 1.486462e-06 0.238986
##
## Fixed effects:  sqrt(C.N.Ratio) ~ Type
##                           Value  Std.Error DF  t-value
## (Intercept)            3.830403 0.09756563 14 39.25976
## TypeSpring-grazed      0.199705 0.13797864 14  1.44737
## TypeUngrazed Control -0.038396 0.13797864 14 -0.27828
##                      p-value
## (Intercept)           0.0000
## TypeSpring-grazed     0.1698
## TypeUngrazed Control  0.7849
##  Correlation:
##                      (Intr) TypSp-
## TypeSpring-grazed    -0.707
## TypeUngrazed Control -0.707  0.500
##
## Standardized Within-Group Residuals:
##        Min         Q1        Med         Q3        Max
## -1.3832300 -0.5188288 -0.1242503  0.4860893  2.6336674
##
## Number of Observations: 18
## Number of Groups: 2
```

```
anova(TotalRatioModel1A)
```

```
##             numDF denDF  F-value p-value
## (Intercept)     1    14 4754.717  <.0001
## Type            2    14    1.717  0.2154
```

```
plot(TotalRatioModel1A)
```

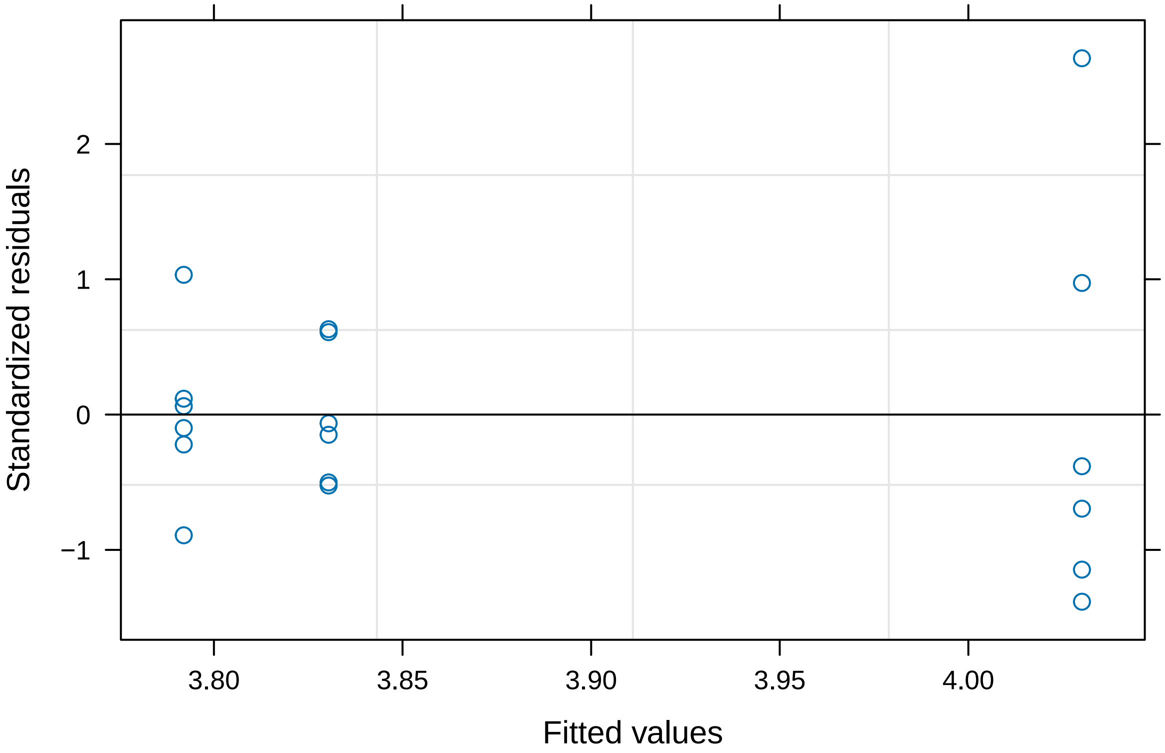


```
qqnorm(TotalRatioModel1A$residuals)
```

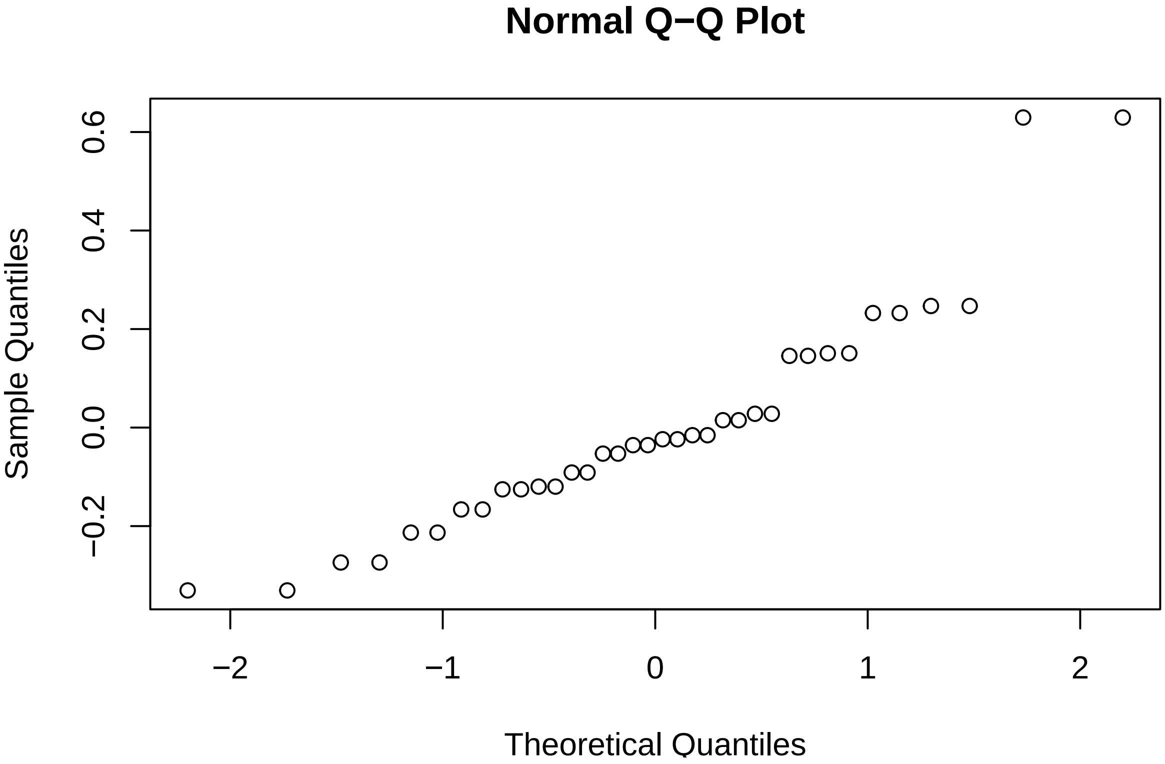

Slightly overdispersed but sqrt transformation should be fine. Suggests grazing type does not influence the total C/N ratio (F2,14 = 1.72, p = 0.215).

Mixed effects model looking at the effect of just Treatment (grazed vs ungrazed):

```r
TotalRatioModel3 <- lme(C.N.Ratio ~ Treatment, random = ~ 1|Block, data = TotalRatio)
summary(TotalRatioModel3)
```

```
## Linear mixed-effects model fit by REML
##   Data: TotalRatio
##        AIC      BIC    logLik
##   80.39603 83.48638 -36.19801
##
## Random effects:
##  Formula: ~1 | Block
##         (Intercept) Residual
## StdDev: 6.838769e-05 2.033567
##
## Fixed effects:  C.N.Ratio ~ Treatment
##                       Value Std.Error DF   t-value
## (Intercept)       15.519044 0.5870403 15 26.436079
## TreatmentUngrazed -1.121266 1.0167836 15 -1.102757
##                   p-value
## (Intercept)        0.0000
## TreatmentUngrazed  0.2875
##  Correlation:
##                   (Intr)
## TreatmentUngrazed -0.577
##
## Standardized Within-Group Residuals:
##        Min         Q1        Med         Q3        Max
## -0.9011145 -0.6574717 -0.1510707  0.1306005  3.0449285
##
## Number of Observations: 18
## Number of Groups: 2
```

```r
anova(TotalRatioModel3)
```

```
##             numDF denDF  F-value p-value
## (Intercept)     1    15 998.4137  <.0001
## Treatment       1    15   1.2161  0.2875
```

```r
plot(TotalRatioModel3)
```

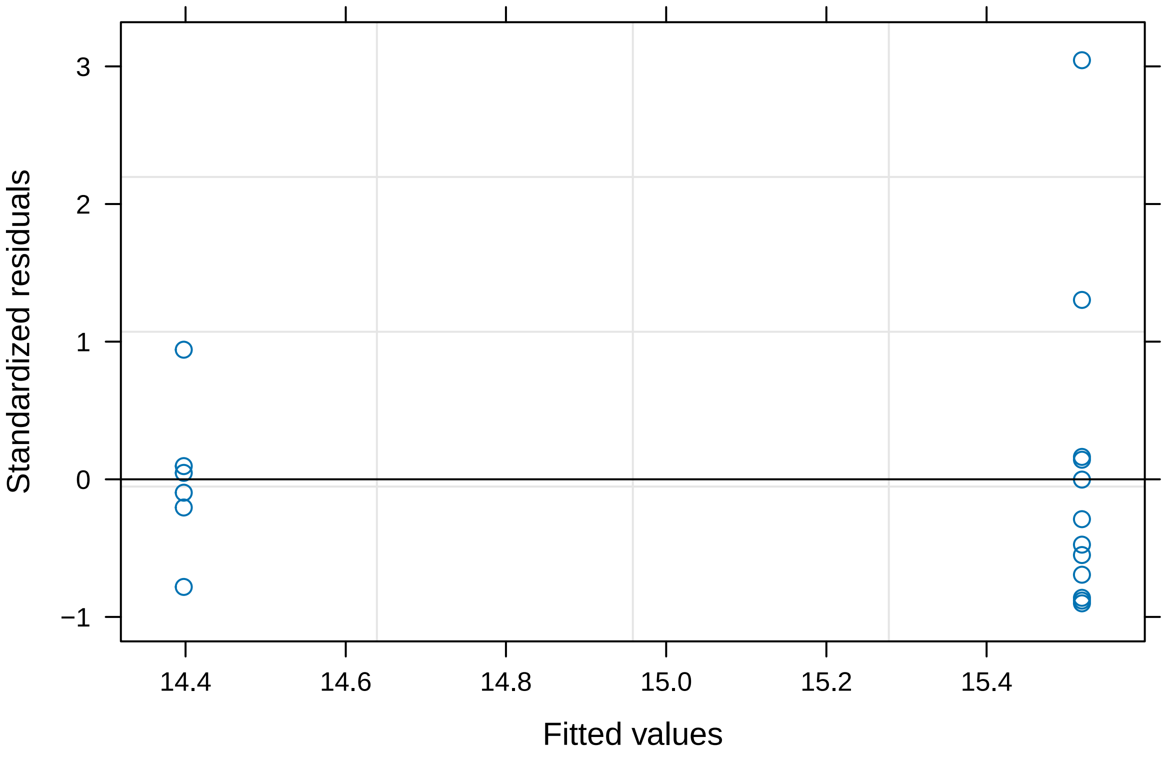


```
qqnorm(TotalRatioModel3$residuals)
```

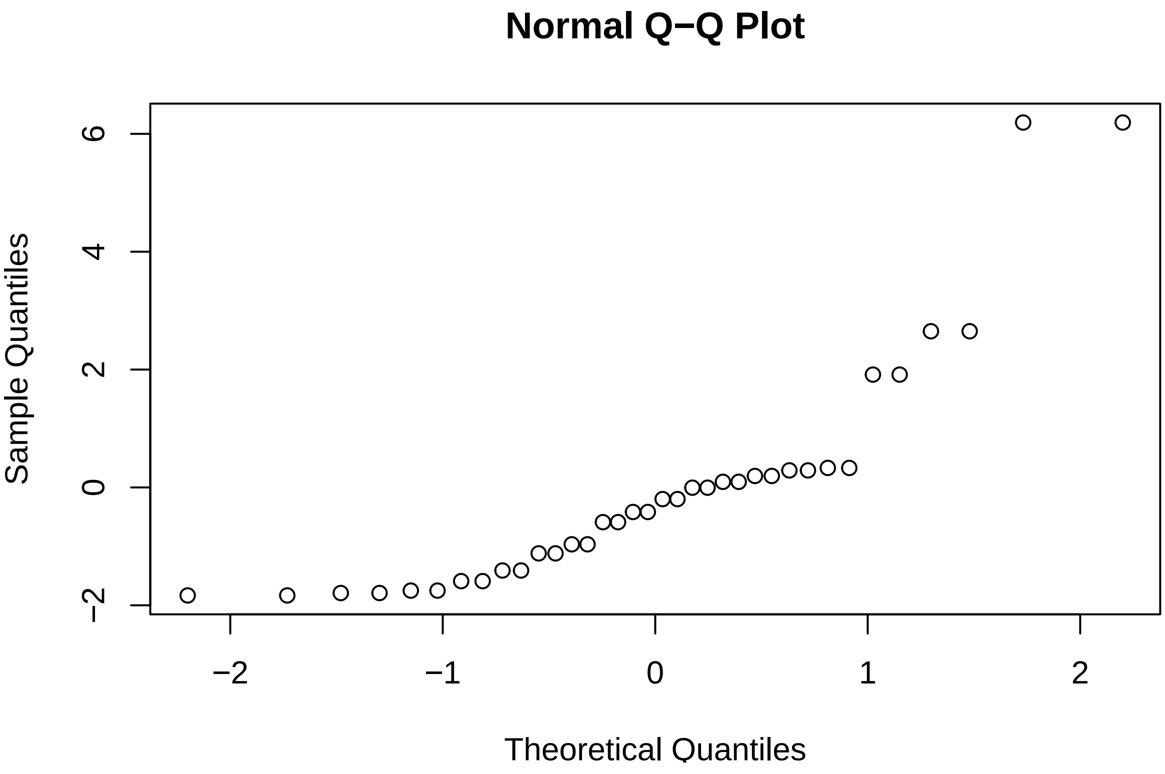


```
TotalRatioModel4 <- lme(log(C.N.Ratio) ~ Treatment, random = ~ 1|Block, data = TotalRatio)
summary(TotalRatioModel4)
```

```
## Linear mixed-effects model fit by REML
```

```
##   Data: TotalRatio
##       AIC       BIC logLik
##   -9.9596 -6.869245 8.9798
##
## Random effects:
##  Formula: ~1 | Block
##          (Intercept)  Residual
## StdDev: 2.209678e-06 0.1207755
##
## Fixed effects:  log(C.N.Ratio) ~ Treatment
##                        Value  Std.Error DF  t-value
## (Intercept)        2.733041 0.03486488 15 78.38951
## TreatmentUngrazed -0.068526 0.06038775 15 -1.13477
##                   p-value
## (Intercept)        0.0000
## TreatmentUngrazed  0.2743
##  Correlation:
##                   (Intr)
## TreatmentUngrazed -0.577
##
## Standardized Within-Group Residuals:
##        Min         Q1        Med         Q3        Max
## -0.9656459 -0.6715316 -0.1576466  0.2038187  2.8547425
##
## Number of Observations: 18
## Number of Groups: 2
```

```r
anova(TotalRatioModel4)
```

```
##             numDF denDF  F-value p-value
## (Intercept)     1    15 9063.945  <.0001
## Treatment       1    15    1.288  0.2743
```

```r
plot(TotalRatioModel4)
```

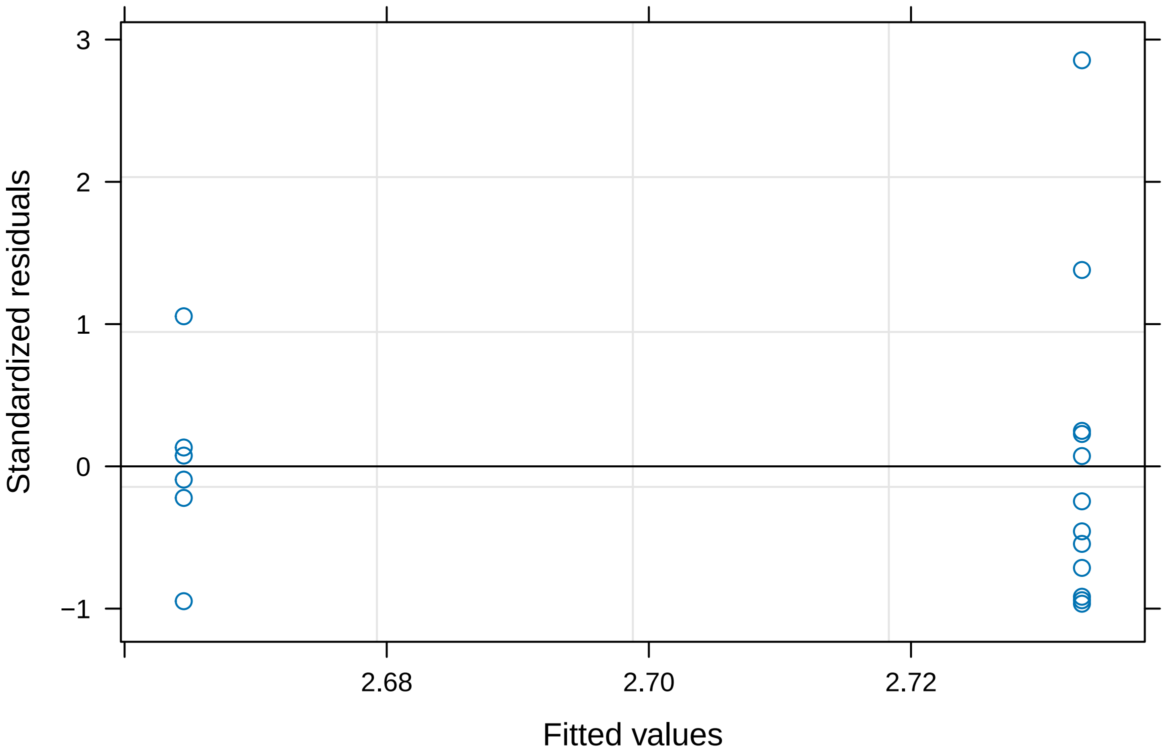


```
qqnorm(TotalRatioModel4$residuals)
```

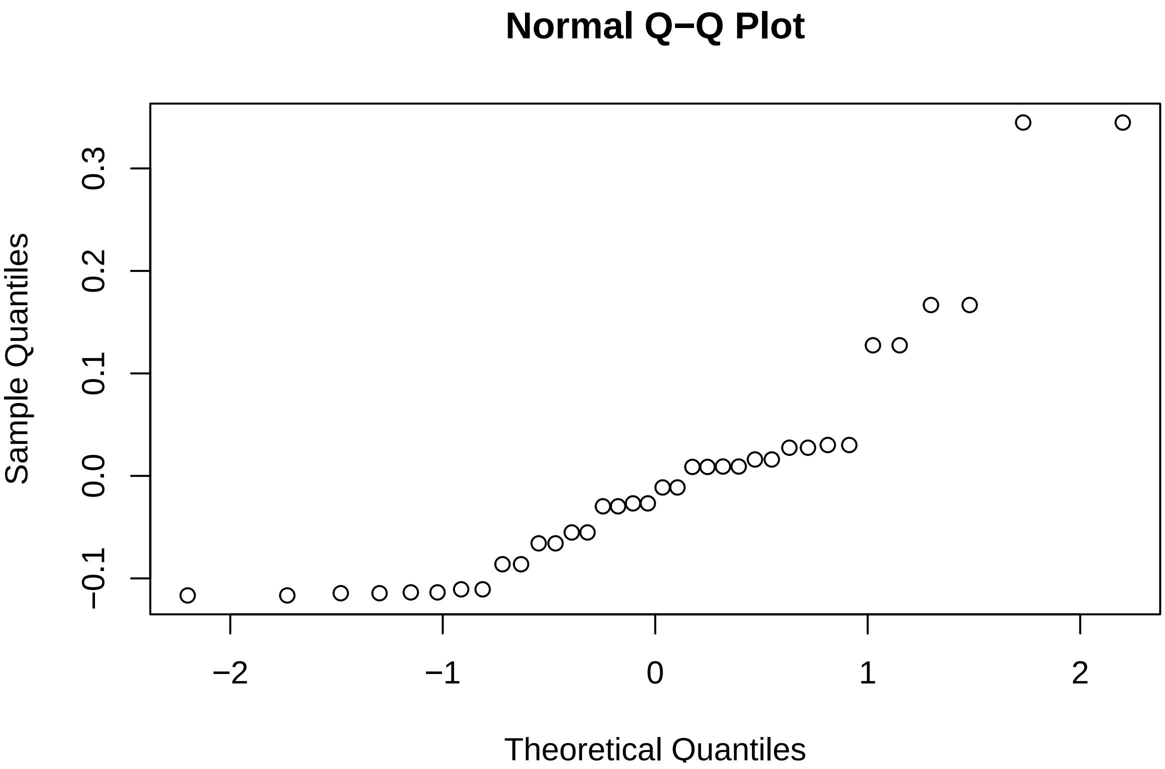

Used log transformation (not perfect but better, esp. given apparent non-significance). Treatment appears to have no effect on total C/N ratio (F1,15 = 1.29, p = 0.274).

Part 4 - Other soil properties Part 4 (Section 1) - pH

Visualization:

```
pH <- completedata2 %>% dplyr::select(Block, ID, Type, Treatment, Study, pH)
yvar <- pH$pH

pHTreatment_means <- pH %>% group_by(Treatment) %>% summarise(Treatment_mean_pH = mean(pH))
pHTreatment_means
```

```
## # A tibble: 2 x 2
##   Treatment Treatment_mean_pH
##   <chr>                 <dbl>
## 1 Grazed                 7.65
## 2 Ungrazed               7.57
```

```
pHType_means <- pH %>% group_by(Type) %>% summarise(Type_mean_pH = mean(pH))
pHType_means
```

```
## # A tibble: 3 x 2
##   Type             Type_mean_pH
##   <chr>                   <dbl>
## 1 Autumn-grazed            7.60
## 2 Spring-grazed            7.70
## 3 Ungrazed Control         7.57
```

```
ggplot(pH) +  geom_point(mapping = aes(x=Treatment, y=yvar, shape = Type, col=Type)) + labs(x = "Grazin
```

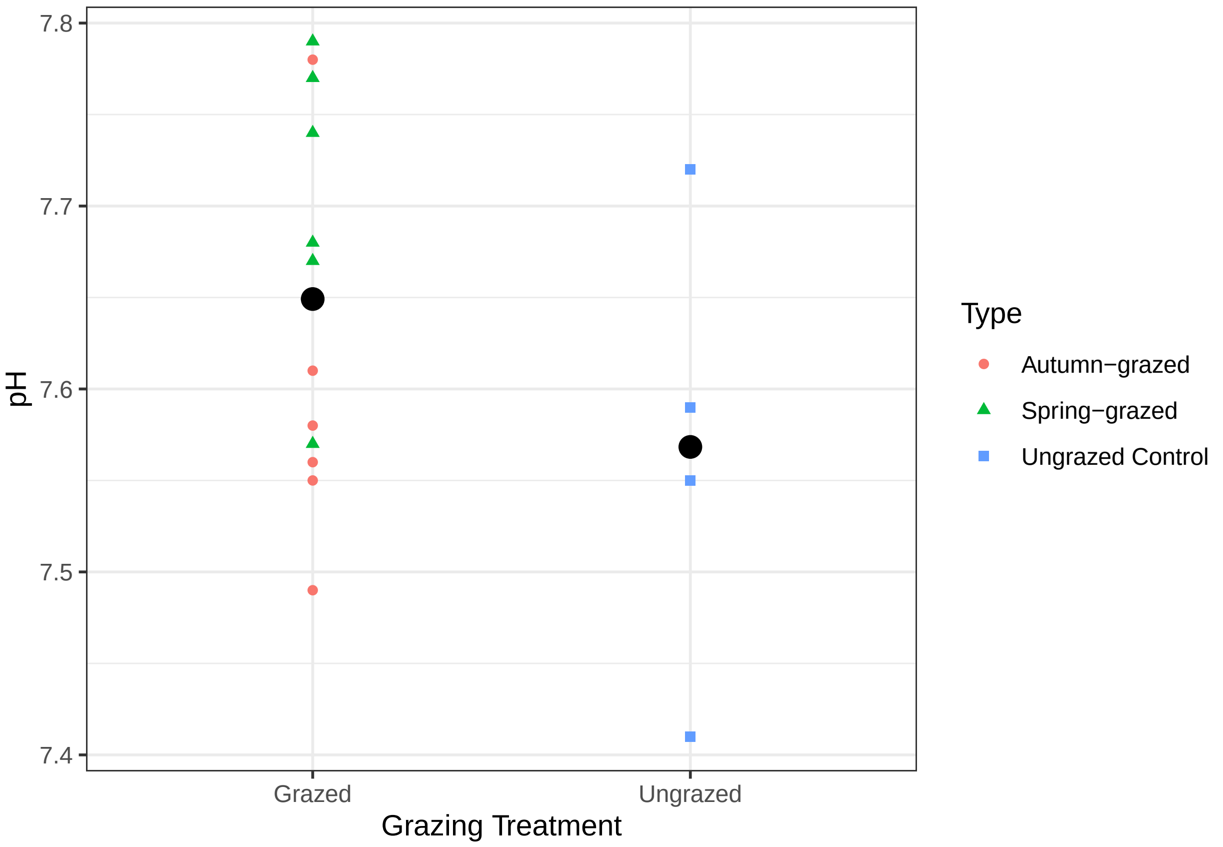

```
ggplot(pH, aes(x=Type, y=pH)) + geom_boxplot(trim=FALSE) + labs(x = "Grazing Type", y = "pH")  + stat_su
```

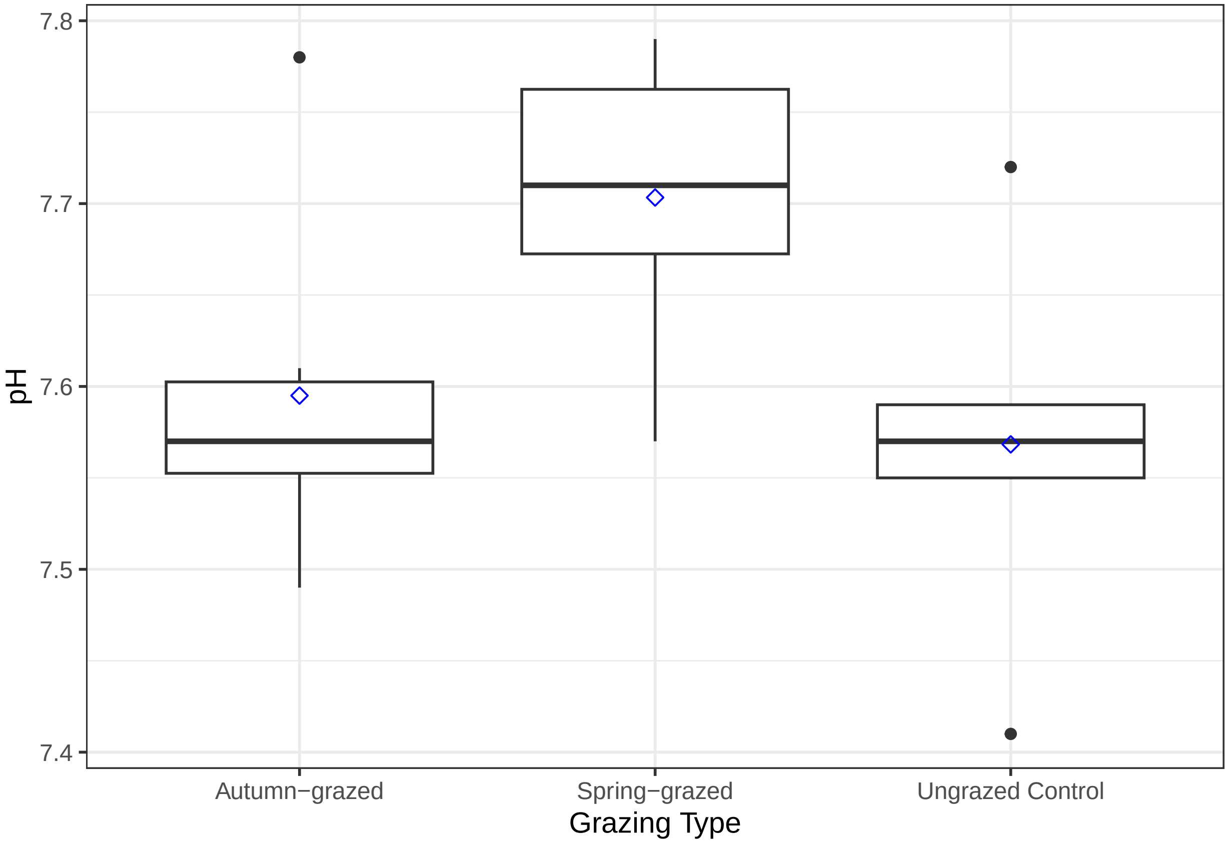


```
ggplot(pH) +  geom_point(mapping = aes(x=Type, y=yvar, shape = Type, col=Type)) + labs(x = "Grazing Typ
```

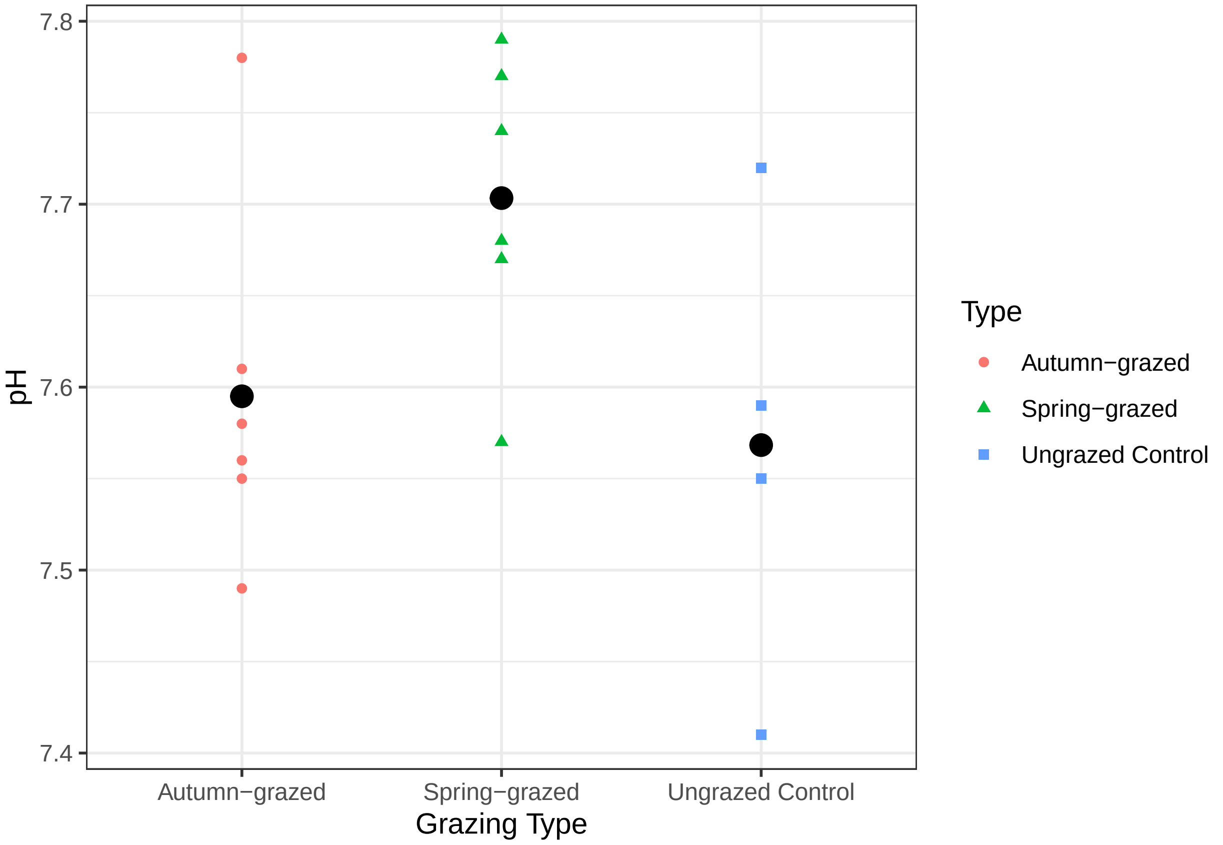


```
# Calculating 95% confidence intervals
UngrazedpH <- pH[c(1,2,3,10,11,12),]
1.96 * sd(UngrazedpH$pH)/sqrt(6)
```

```
## [1] 0.07976288
```

```
SpringpH <- pH[c(7,8,9,16,17,18),]
1.96 * sd(SpringpH$pH)/sqrt(6)
```

```
## [1] 0.06474266
```

```
AutumnpH <- pH[c(4,5,6,13,14,15),]
1.96 * sd(AutumnpH$pH)/sqrt(6)
```

```
## [1] 0.07917203
```

```
GrazedpH <- pH[c(4,5,6,7,8,9,13,14,15,16,17,18),]
1.96 * sd(GrazedpH$pH)/sqrt(6)
```

```
## [1] 0.08248517
```

Visual conclusions: Mean pH is higher in the grazed treatment than the ungrazed treatment. This is mostly driven by the higher pH in the spring-grazed treatment (i.e., type has an effect).

Mixed Effects Model looking at the effects of just grazing type (Spring vs Autumn vs Ungrazed Control):

```
pHModel1 <- lme(pH ~ Type, random = ~ 1|Block, data = pH)
summary(pHModel1)
```

```
## Linear mixed-effects model fit by REML
##   Data: pH
```

```
##          AIC       BIC   logLik
##   -13.14828 -9.608024 11.57414
##
## Random effects:
##  Formula: ~1 | Block
##          (Intercept)   Residual
## StdDev:  0.01542176 0.09281745
##
## Fixed effects:  pH ~ Type
##                           Value  Std.Error DF   t-value
## (Intercept)            7.595000 0.03943047 14 192.61754
## TypeSpring-grazed      0.108333 0.05358818 14   2.02159
## TypeUngrazed Control -0.026667 0.05358818 14  -0.49762
##                       p-value
## (Intercept)            0.0000
## TypeSpring-grazed      0.0628
## TypeUngrazed Control   0.6265
##  Correlation:
##                       (Intr) TypSp-
## TypeSpring-grazed    -0.68
## TypeUngrazed Control -0.68   0.50
##
## Standardized Within-Group Residuals:
##        Min         Q1        Med         Q3        Max
## -1.6534457 -0.3988010 -0.1795639  0.3284329  2.0455713
##
## Number of Observations: 18
## Number of Groups: 2
```

```r
anova(pHModel1)
```

```
##             numDF denDF  F-value p-value
## (Intercept)     1    14 97230.58  <.0001
## Type            2    14     3.56  0.0562
```

```r
plot(pHModel1)
```

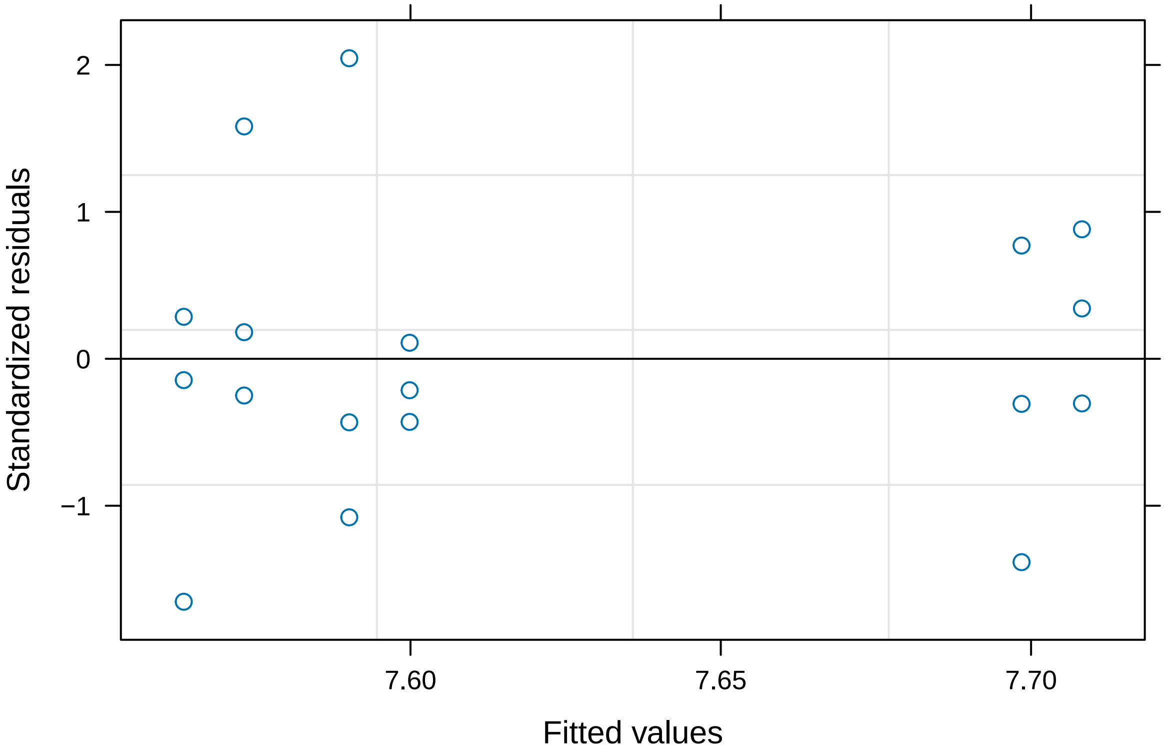


```r
qqnorm(pHModel1$residuals)
```

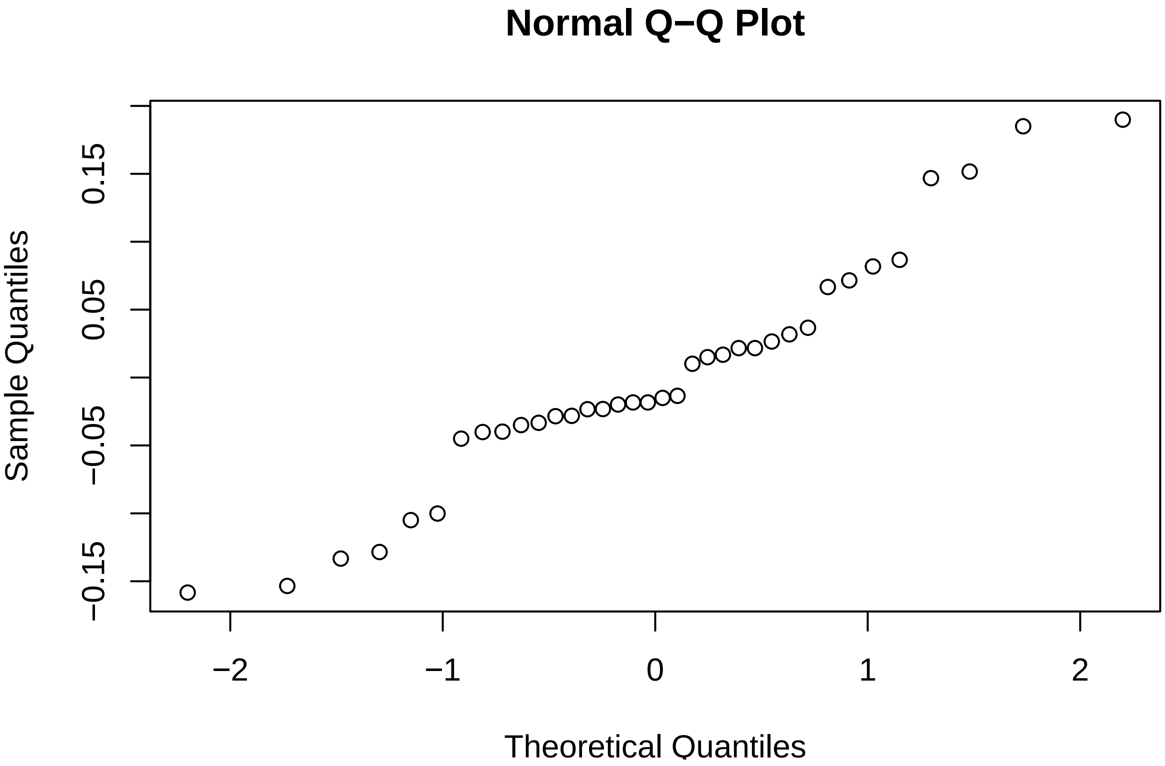


```r
## Changing order to get last comparison
levels(pH$Type)
```

```
## NULL
```

```r
pH$Type <- factor(pH$Type, levels=c('Ungrazed Control','Autumn-grazed', 'Spring-grazed'))
pHModel2 <- lme(pH ~ Type, random = ~ 1|Block, data = pH)
summary(pHModel2)
```

```
## Linear mixed-effects model fit by REML
##   Data: pH
##         AIC       BIC   logLik
##   -13.14828 -9.608024 11.57414
##
## Random effects:
##  Formula: ~1 | Block
##         (Intercept)   Residual
## StdDev:  0.01542176 0.09281745
##
## Fixed effects:  pH ~ Type
##                        Value  Std.Error DF   t-value
## (Intercept)         7.568333 0.03943047 14 191.94124
## TypeAutumn-grazed 0.026667 0.05358818 14   0.49762
## TypeSpring-grazed 0.135000 0.05358818 14   2.51921
##                     p-value
## (Intercept)          0.0000
## TypeAutumn-grazed  0.6265
## TypeSpring-grazed  0.0245
##  Correlation:
##                     (Intr) TypAt-
## TypeAutumn-grazed -0.68
## TypeSpring-grazed -0.68   0.50
##
## Standardized Within-Group Residuals:
##        Min          Q1         Med          Q3         Max
## -1.6534457 -0.3988010 -0.1795639  0.3284329  2.0455713
##
## Number of Observations: 18
## Number of Groups: 2
```

```r
anova(pHModel2)
```

```
##             numDF denDF  F-value p-value
## (Intercept)     1    14 97230.58  <.0001
## Type            2    14     3.56  0.0562
```

```r
plot(pHModel2)
```

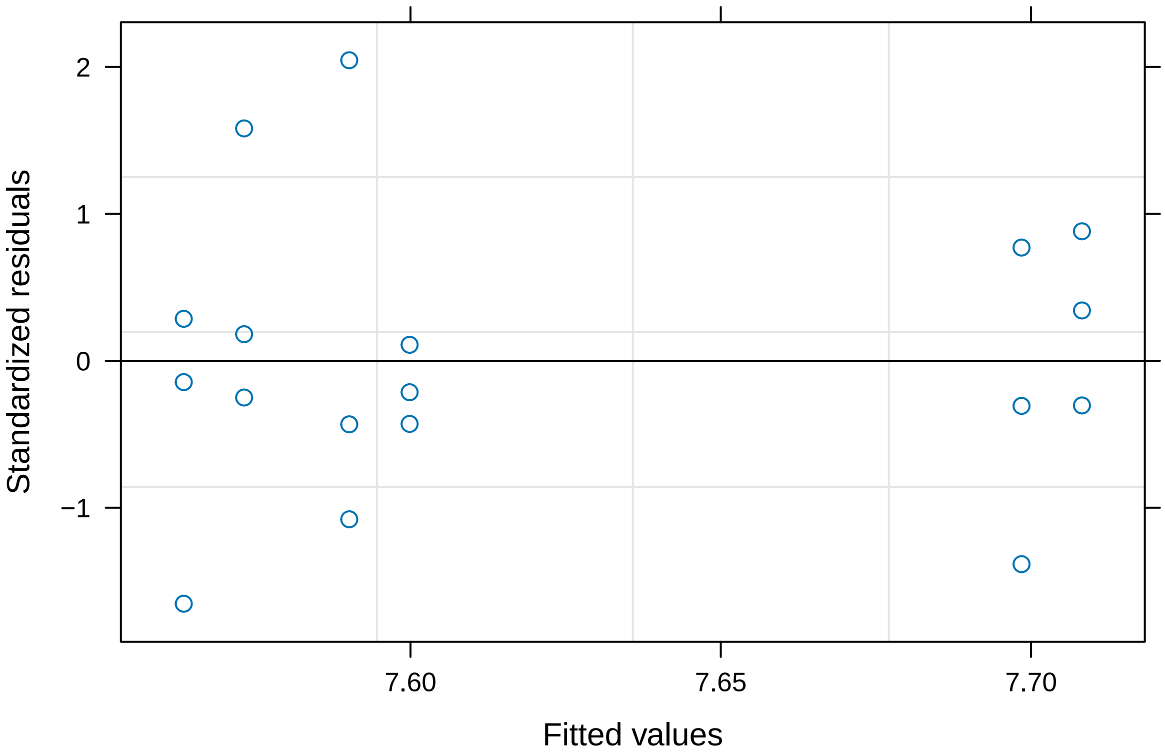


```r
qqnorm(pHModel2$residuals)
```

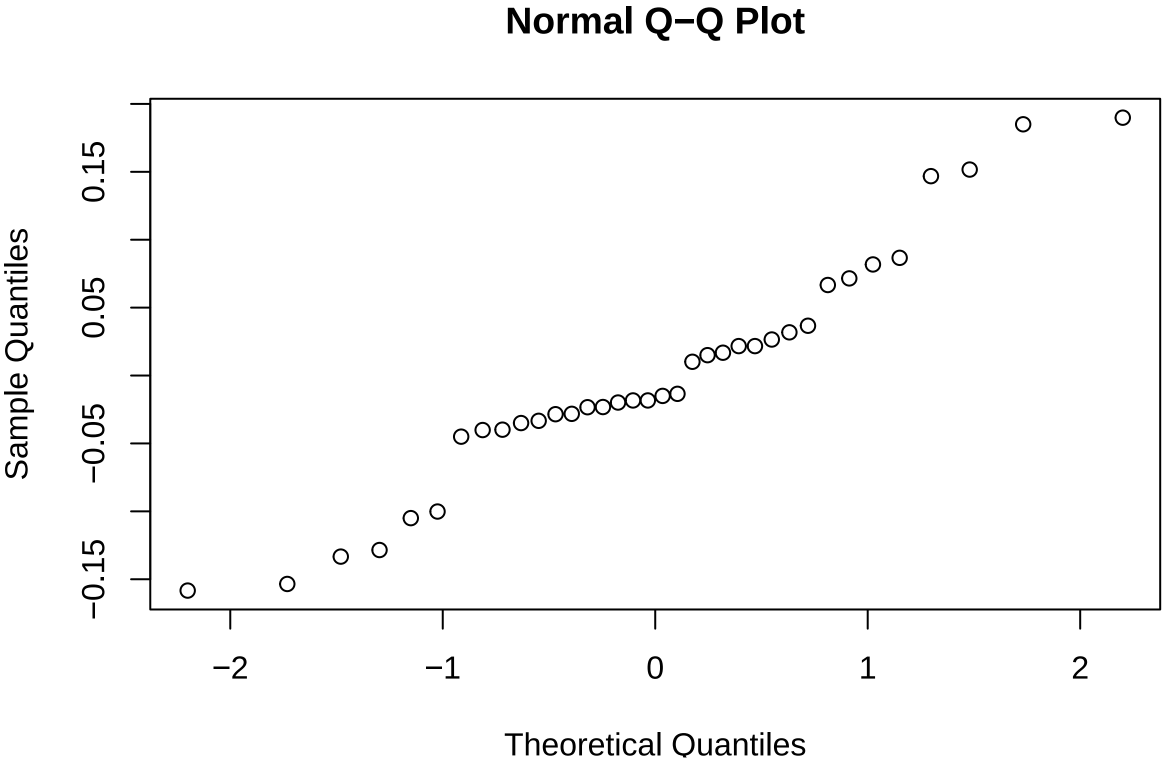

Meets assumptions fine. Suggests that specific grazing type does have a weakly significant impact on pH ($F_{2,14}$ = 3.56, p = 0.056). While the autumn-grazed paddocks did not have a significantly different pH from the ungrazed control (t_{14} = 0.497, p = 0.627), the pH in the spring-grazed paddocks was significantly higher (more alkaline) than the ungrazed control (t_{14} = 2.52, p = 0.025).

Mixed effects model looking at the effect of just Treatment (grazed vs ungrazed):

```
pHModel3 <- lme(pH ~ Treatment, random = ~ 1|Block, data = pH)
summary(pHModel3)
```

```
## Linear mixed-effects model fit by REML
##   Data: pH
##         AIC       BIC   logLik
##   -15.35625 -12.26589 11.67812
##
## Random effects:
##  Formula: ~1 | Block
##         (Intercept)  Residual
## StdDev: 0.006390904 0.1019214
##
## Fixed effects:  pH ~ Treatment
##                        Value  Std.Error DF   t-value
## (Intercept)         7.649167 0.02976719 15 256.96640
## TreatmentUngrazed -0.080833 0.05096068 15  -1.58619
##                    p-value
## (Intercept)         0.0000
## TreatmentUngrazed  0.1335
##  Correlation:
##                    (Intr)
## TreatmentUngrazed -0.571
##
## Standardized Within-Group Residuals:
##         Min          Q1         Med          Q3
## -1.55346469 -0.74811551  0.01635248  0.73584082
##         Max
##  1.47987854
##
## Number of Observations: 18
## Number of Groups: 2
```

```
anova(pHModel3)
```

```
##             numDF denDF  F-value p-value
## (Intercept)     1    15 97230.58  <.0001
## Treatment       1    15     2.52  0.1335
```

```
plot(pHModel3)
```

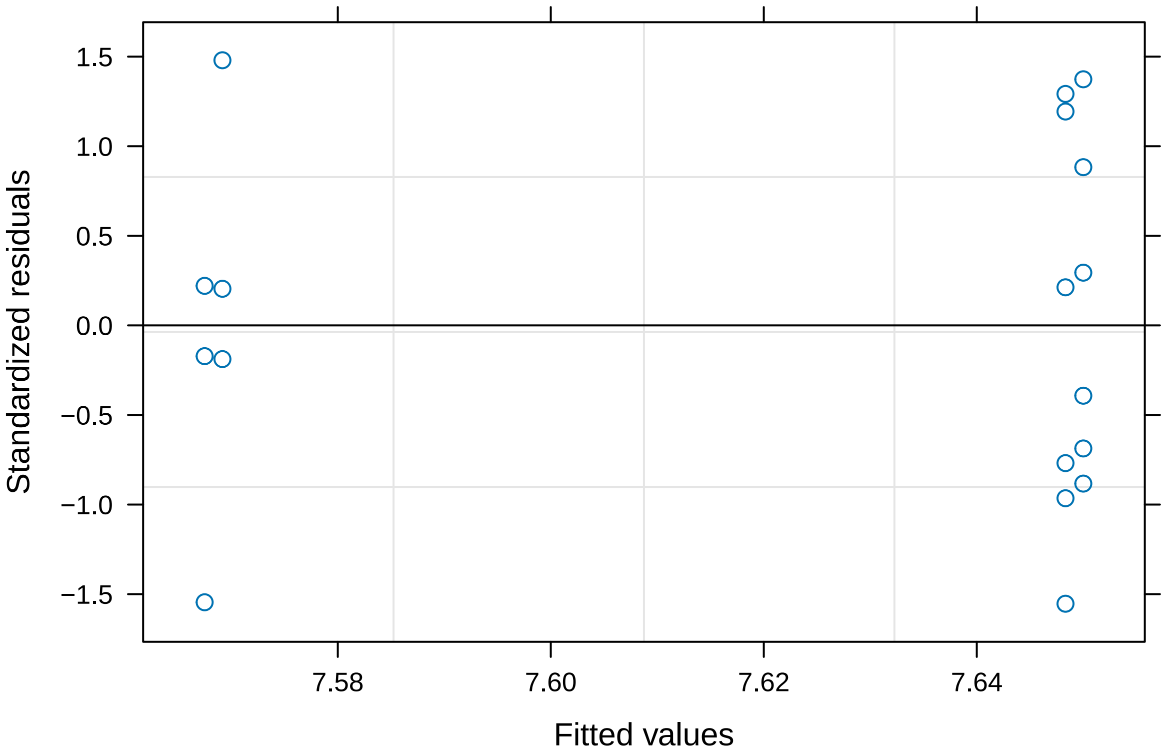


```r
qqnorm(pHModel3$residuals)
```

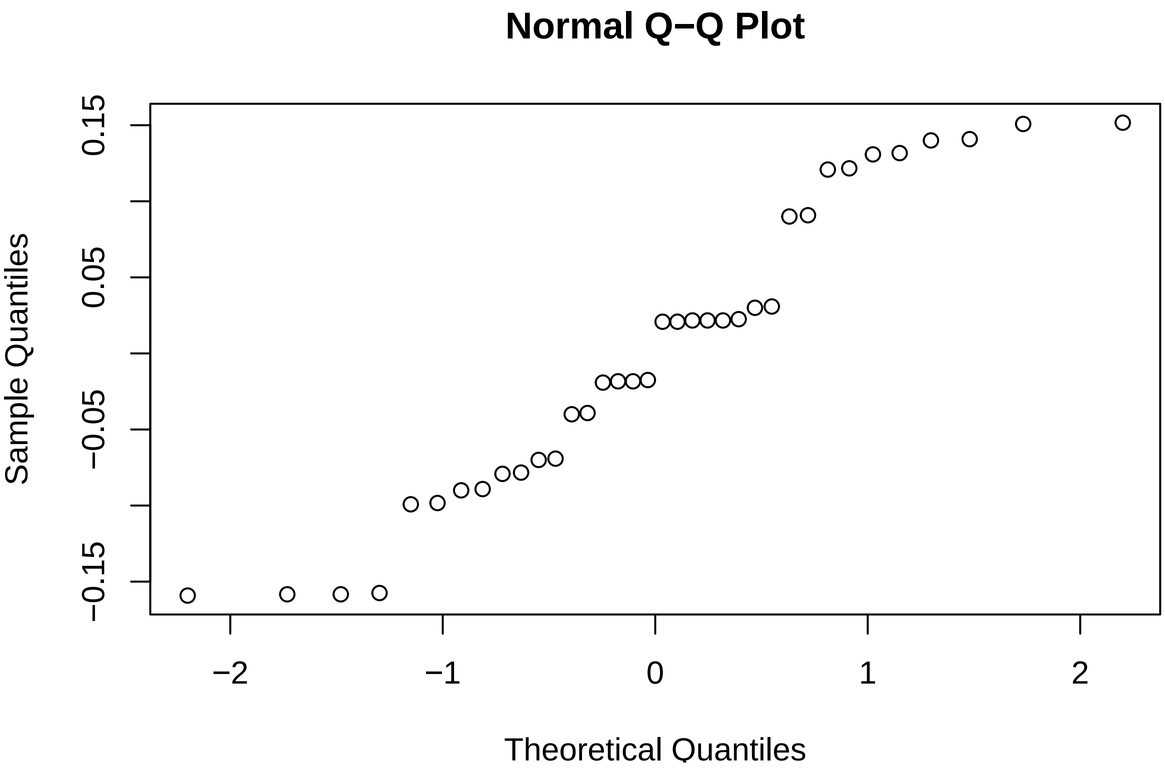

Overall, grazing does not affect pH (F1,15 = 2.52, p = 0.134). However, see effects of grazing type above. This suggests the differences are indeed due to grazing type and not grazing in general.

Comparing models:

```r
anova(pHModel3,pHModel1)
```

```
##          Model df       AIC        BIC   logLik   Test
## pHModel3     1  4 -15.35625 -12.265892 11.67812
## pHModel1     2  5 -13.14827  -9.608024 11.57414 1 vs 2
##            L.Ratio p-value
## pHModel3
## pHModel1 0.2079717  0.6484
```

This suggests the models are not significantly different from each other.

Part 4 (Section 2) - Bulk Density Visualization:

```r
BD <- completedata2 %>% dplyr::select(Block, ID, Type, Treatment, Study, BD)
yvar <- BD$BD

BDTreatment_means <- BD %>% group_by(Treatment) %>% summarise(Treatment_mean_BD = mean(BD))
BDTreatment_means
```

```
## # A tibble: 2 x 2
##   Treatment Treatment_mean_BD
##   <chr>                 <dbl>
## 1 Grazed                0.780
## 2 Ungrazed              0.781
```

```r
BDType_means <- BD %>% group_by(Type) %>% summarise(Type_mean_BD = mean(BD))
BDType_means
```

```
## # A tibble: 3 x 2
##   Type             Type_mean_BD
##   <chr>                   <dbl>
## 1 Autumn-grazed           0.755
## 2 Spring-grazed           0.806
## 3 Ungrazed Control        0.781
```

```r
ggplot(BD) +  geom_point(mapping = aes(x=Treatment, y=yvar, shape = Type, col=Type)) + labs(x = "Grazin
```

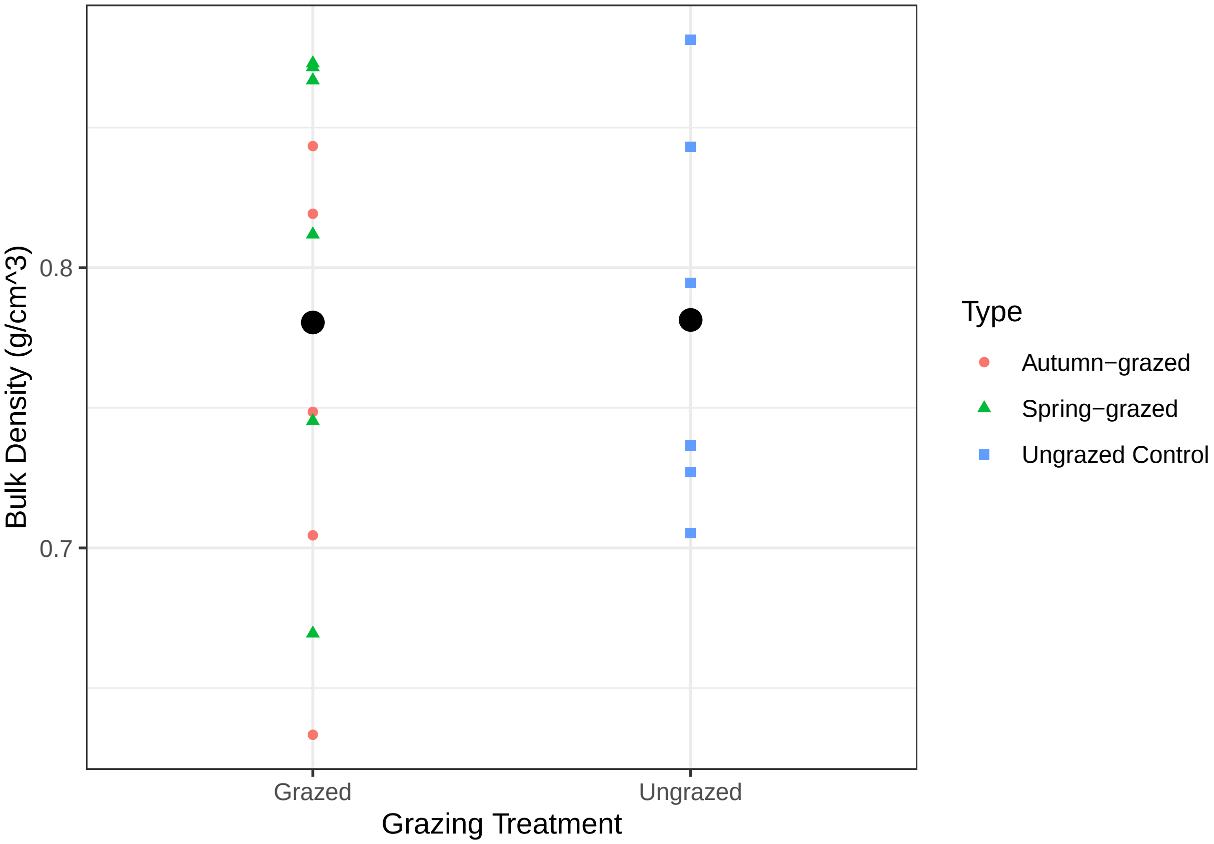


```
ggplot(BD, aes(x=Type, y=BD)) + geom_boxplot(trim=FALSE) + labs(x = "Grazing Type", y = "Bulk Density (g
```

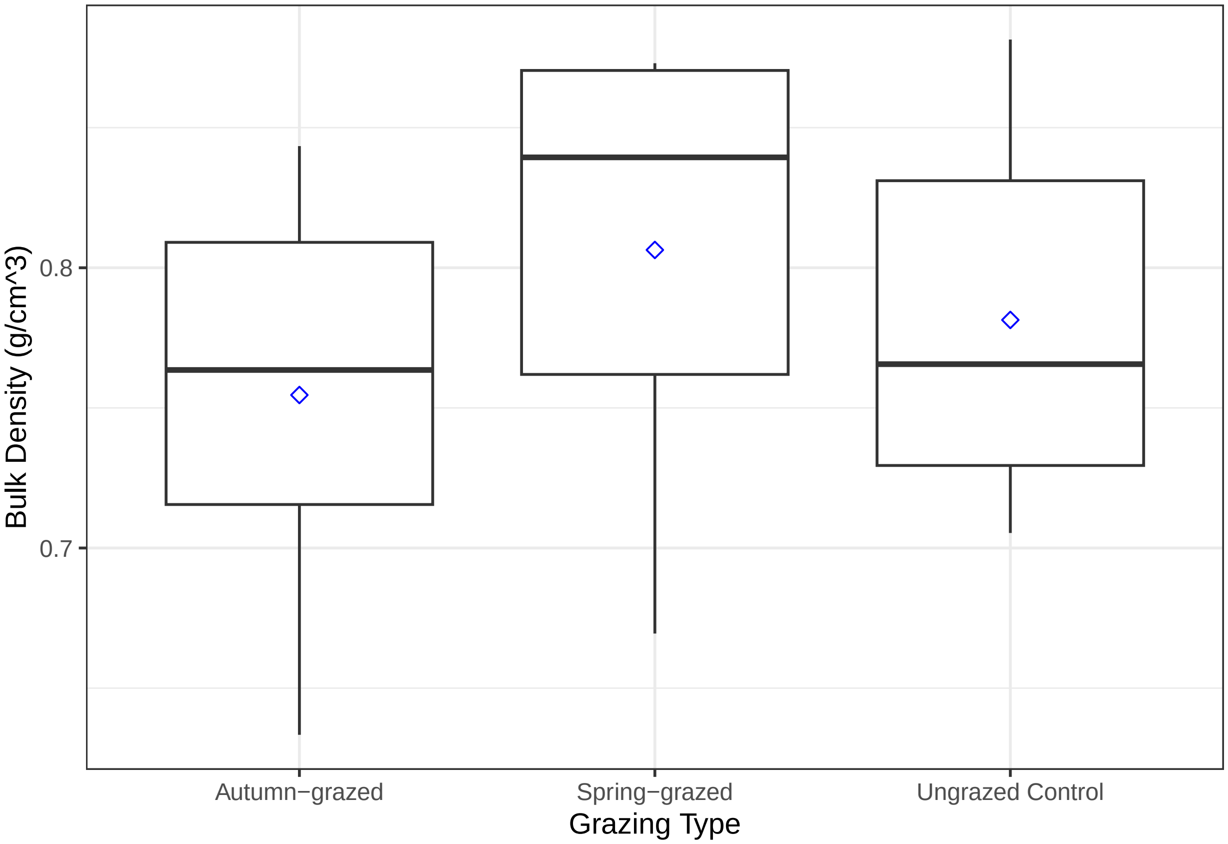


```
# Calculating 95% confidence intervals
UngrazedBD <- BD[c(1,2,3,10,11,12),]
1.96 * sd(UngrazedBD$BD)/sqrt(6)
```

```
## [1] 0.05629206
```

```
SpringBD <- BD[c(7,8,9,16,17,18),]
1.96 * sd(SpringBD$BD)/sqrt(6)
```

```
## [1] 0.0668075
```

```
AutumnBD <- BD[c(4,5,6,13,14,15),]
1.96 * sd(AutumnBD$BD)/sqrt(6)
```

```
## [1] 0.06186856
```

```
GrazedBD <- BD[c(4,5,6,7,8,9,13,14,15,16,17,18),]
1.96 * sd(GrazedBD$BD)/sqrt(6)
```

```
## [1] 0.06508746
```

Visual conclusions: Grazing treatment and grazing type don't seem to have an effect on bulk density.

Mixed Effects Model looking at the effects of just grazing type (Spring vs Autumn vs Ungrazed Control):

```
BDModel1 <- lme(BD ~ Type, random = ~ 1|Block, data = BD)
summary(BDModel1)
```

```
## Linear mixed-effects model fit by REML
##   Data: BD
##         AIC      BIC   logLik
```

```
##   -25.99425 -22.454 17.99712
##
## Random effects:
##  Formula: ~1 | Block
##         (Intercept)  Residual
## StdDev:  0.06909106 0.0556955
##
## Fixed effects:  BD ~ Type
##                          Value  Std.Error DF   t-value
## (Intercept)          0.7545967 0.05388679 14 14.003371
## TypeSpring-grazed    0.0517567 0.03215581 14  1.609559
## TypeUngrazed Control 0.0267833 0.03215581 14  0.832924
##                      p-value
## (Intercept)           0.0000
## TypeSpring-grazed     0.1298
## TypeUngrazed Control  0.4189
##  Correlation:
##                      (Intr) TypSp-
## TypeSpring-grazed    -0.298
## TypeUngrazed Control -0.298  0.500
##
## Standardized Within-Group Residuals:
##          Min           Q1          Med           Q3
## -1.610402313 -0.586772375 -0.004617386  0.340326713
##          Max
##  2.018256868
##
## Number of Observations: 18
## Number of Groups: 2
```

```r
anova(BDModel1)
```

```
##             numDF denDF   F-value p-value
## (Intercept)     1    14 238.21163  <.0001
## Type            2    14   1.29587  0.3045
```

```r
plot(BDModel1)
```

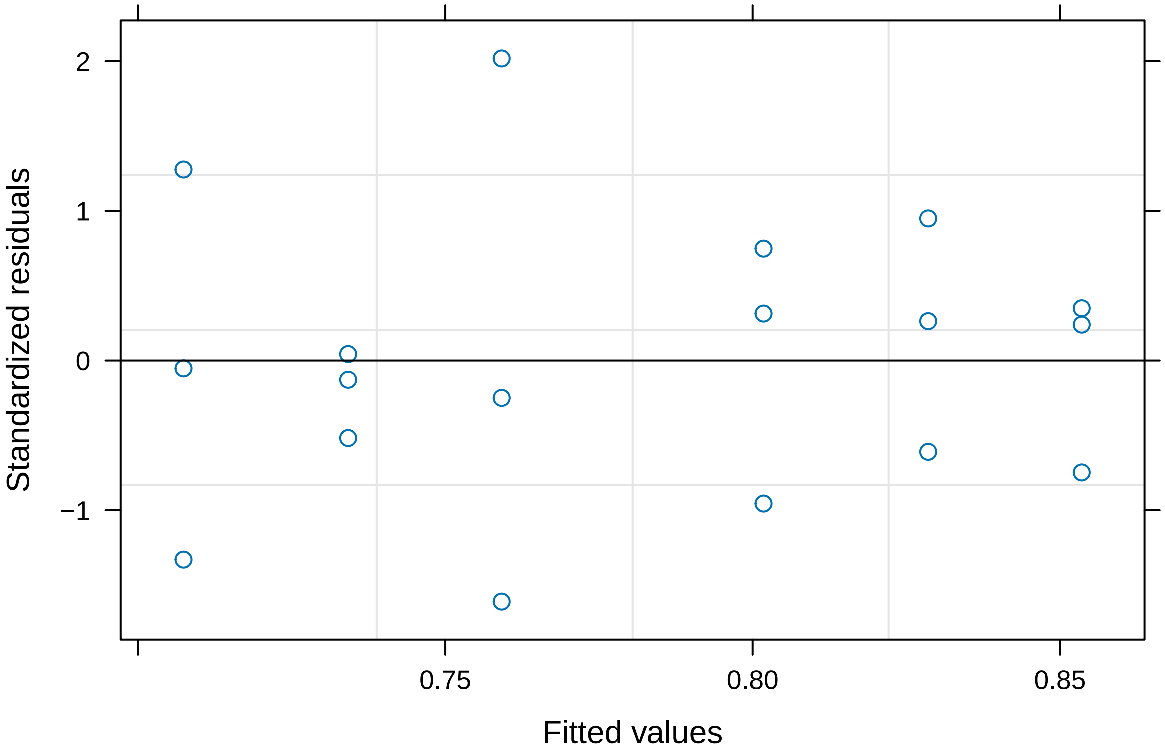


```r
qqnorm(BDModel1$residuals)
```

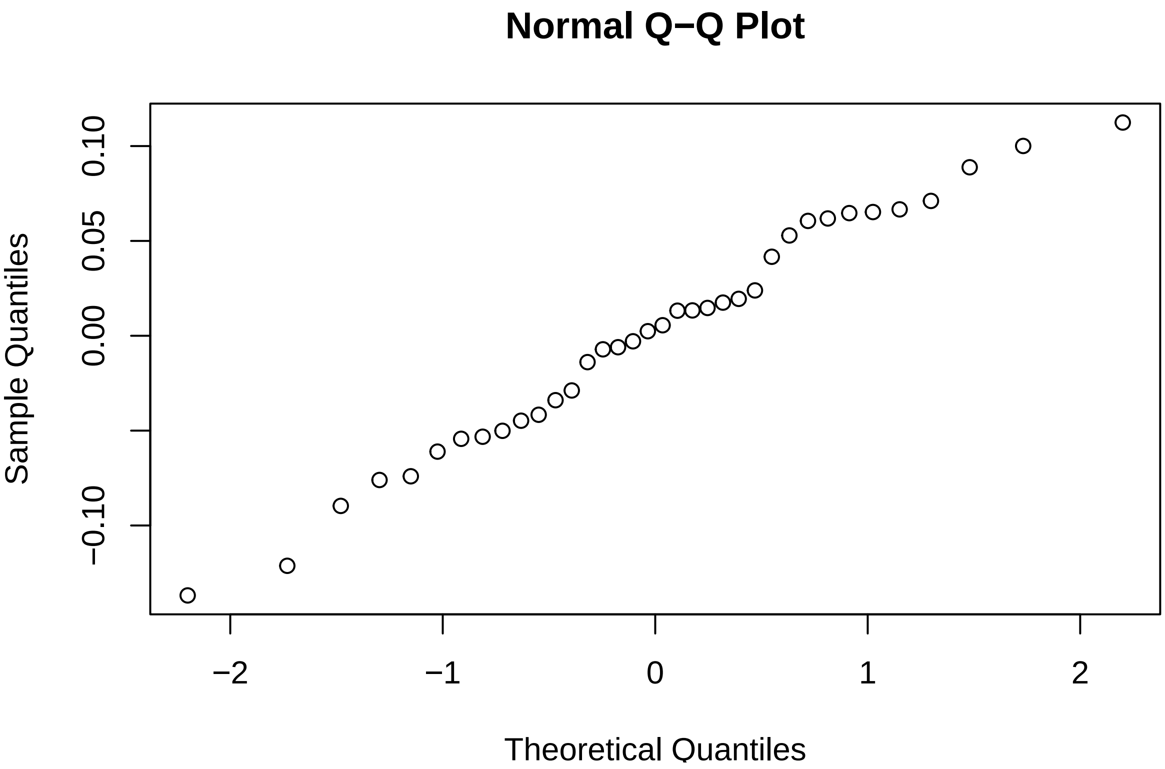


```r
## Changing order to get last comparison
levels(BD$Type)
```

```
## NULL
```

```r
BD$Type <- factor(BD$Type, levels=c('Autumn-grazed', 'Spring-grazed','Ungrazed Control'))
BDModel2 <- lme(BD ~ Type, random = ~ 1|Block, data = BD)
summary(BDModel2)
```

```
## Linear mixed-effects model fit by REML
##   Data: BD
##         AIC     BIC   logLik
##   -25.99425 -22.454 17.99712
##
## Random effects:
##  Formula: ~1 | Block
##         (Intercept)  Residual
## StdDev:  0.06909106 0.0556955
##
## Fixed effects:  BD ~ Type
##                            Value  Std.Error DF   t-value
## (Intercept)            0.7545967 0.05388679 14 14.003371
## TypeSpring-grazed      0.0517567 0.03215581 14  1.609559
## TypeUngrazed Control   0.0267833 0.03215581 14  0.832924
##                        p-value
## (Intercept)             0.0000
## TypeSpring-grazed       0.1298
## TypeUngrazed Control    0.4189
##  Correlation:
##                        (Intr) TypSp-
## TypeSpring-grazed      -0.298
## TypeUngrazed Control -0.298  0.500
##
## Standardized Within-Group Residuals:
##          Min           Q1          Med           Q3
## -1.610402313 -0.586772375 -0.004617386  0.340326713
##          Max
##  2.018256868
##
## Number of Observations: 18
## Number of Groups: 2
```

```r
anova(BDModel2)
```

```
##             numDF denDF   F-value p-value
## (Intercept)     1    14 238.21163  <.0001
## Type            2    14   1.29587  0.3045
```

```r
plot(BDModel2)
```

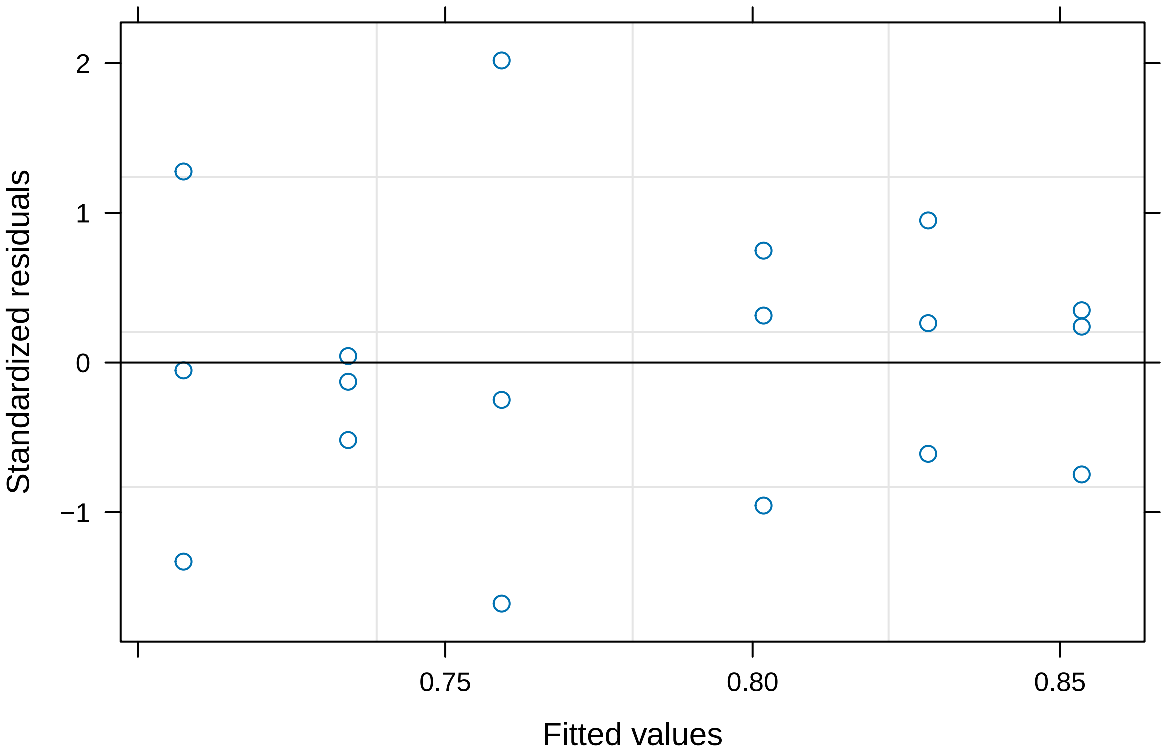


```
qqnorm(BDModel2$residuals)
```

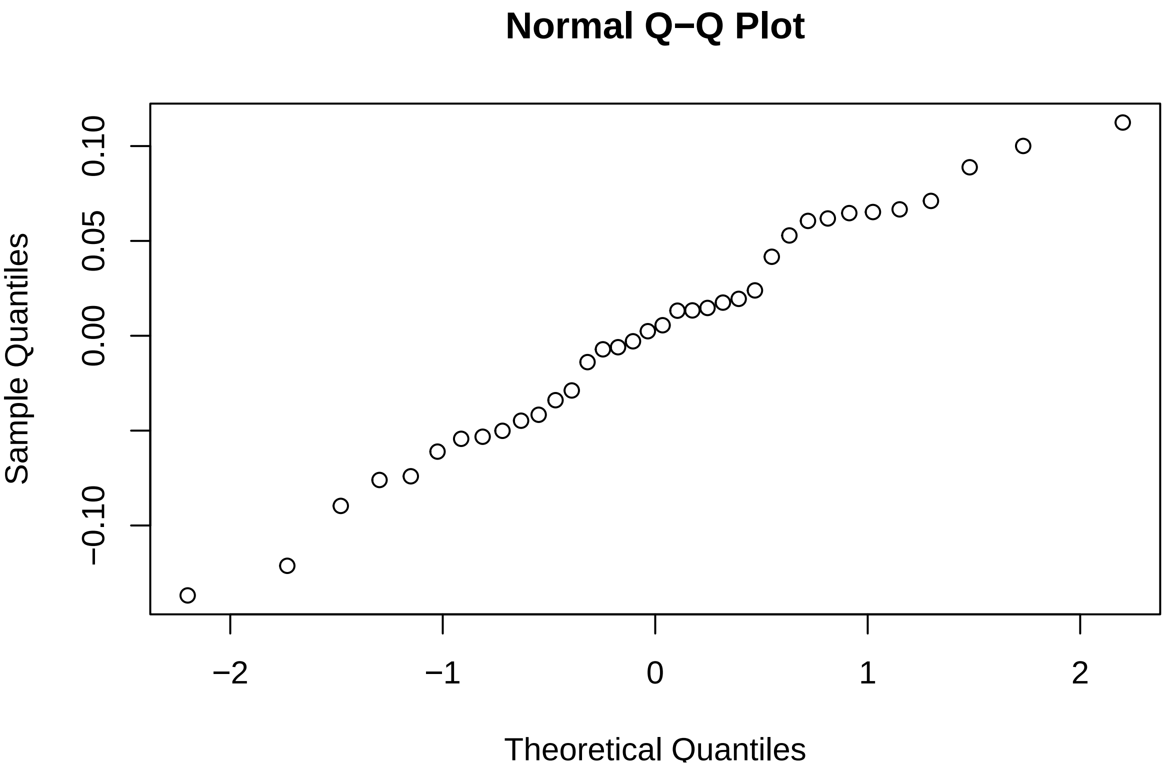

Meets assumptions fine. Suggests grazing type has no effect on BD (F2,14 = 1.30, p = 0.305).

Mixed effects model looking at the effect of just Treatment (grazed vs ungrazed):

```r
BDModel3 <- lme(BD ~ Treatment, random = ~ 1|Block, data = BD)
summary(BDModel3)
```

```
## Linear mixed-effects model fit by REML
##   Data: BD
##         AIC       BIC   logLik
##   -30.51883 -27.42848 19.25942
##
## Random effects:
##  Formula: ~1 | Block
##         (Intercept)   Residual
## StdDev:  0.06882605 0.05857423
##
## Fixed effects:  BD ~ Treatment
##                        Value  Std.Error DF   t-value
## (Intercept)       0.780475 0.05152110 15 15.148647
## TreatmentUngrazed 0.000905 0.02928711 15  0.030901
##                   p-value
## (Intercept)        0.0000
## TreatmentUngrazed  0.9758
##  Correlation:
##                   (Intr)
## TreatmentUngrazed -0.189
##
## Standardized Within-Group Residuals:
##         Min          Q1         Med          Q3
## -1.71261475 -0.49832221 -0.04660905  0.57593740
##         Max
##  2.35470294
##
## Number of Observations: 18
## Number of Groups: 2
```

```r
anova(BDModel3)
```

```
##             numDF denDF   F-value p-value
## (Intercept)     1    15 238.21164  <.0001
## Treatment       1    15   0.00095  0.9758
```

```r
plot(BDModel3)
```

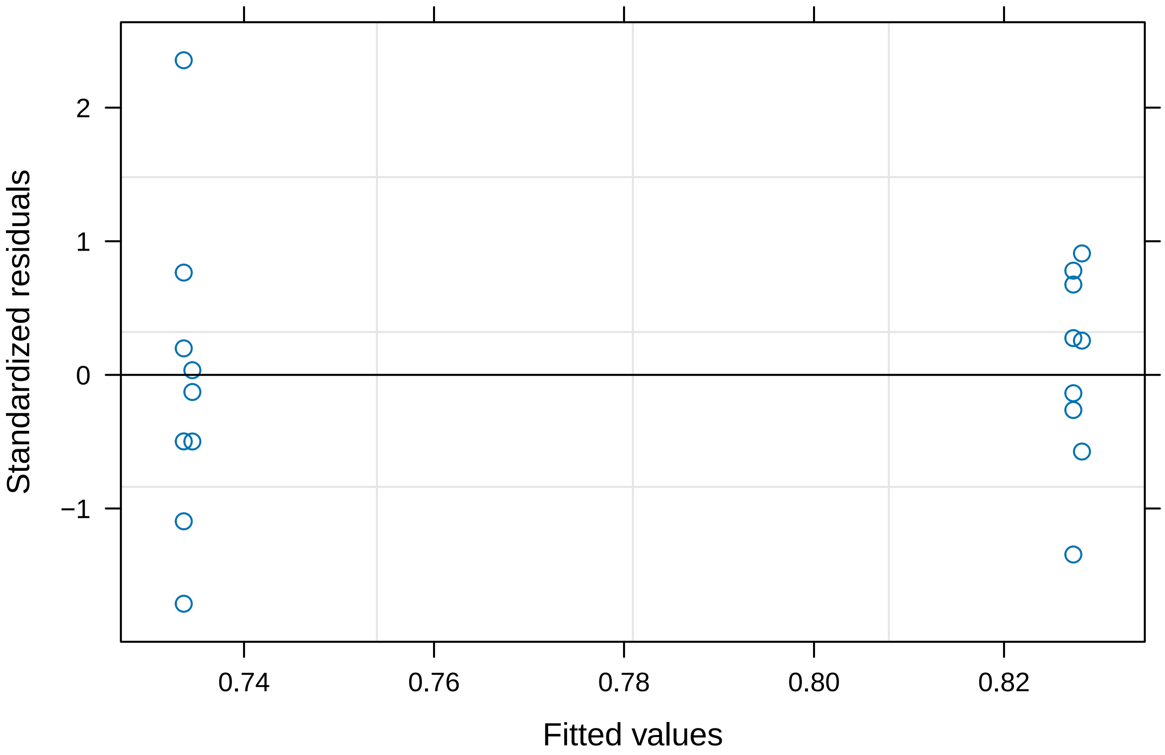


```r
qqnorm(BDModel3$residuals)
```

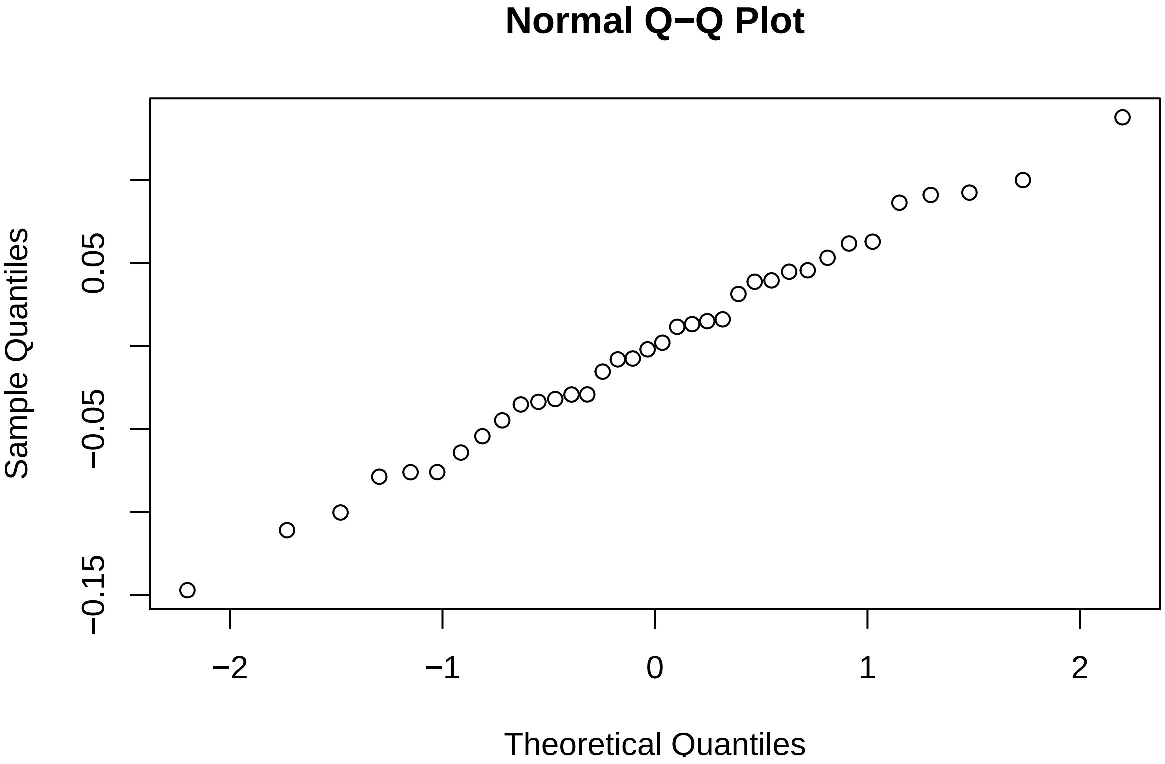

Meets assumptions fine. Suggests grazing has no effect on BD ($F_{1,15} < 0.001$, $p = 0.976$). This is the accurate conclusion given the non-significance of grazing type (above).

Part 4 (Section 3) - Inorganic C Visualization:

```
Inorganic_C <- completedata2 %>% dplyr::select(Block, ID, Type, Treatment, Study, Inorganic.C.stocks..kg
yvar <- Inorganic_C$Inorganic.C.stocks..kgC.m2.

Inorganic_CTreatment_means <- Inorganic_C %>% group_by(Treatment) %>% summarise(Treatment_mean_Inorgani
Inorganic_CTreatment_means
```

```
## # A tibble: 2 x 2
##   Treatment Treatment_mean_Inorganic_C
##   <chr>                          <dbl>
## 1 Grazed                         0.516
## 2 Ungrazed                       0.477
```

```
Inorganic_CType_means <- Inorganic_C %>% group_by(Type) %>% summarise(Type_mean_Inorganic_C = mean(Inorg
Inorganic_CType_means
```

```
## # A tibble: 3 x 2
##   Type             Type_mean_Inorganic_C
##   <chr>                            <dbl>
## 1 Autumn-grazed                    0.468
## 2 Spring-grazed                    0.565
## 3 Ungrazed Control                 0.477
```

```
ggplot(Inorganic_C) +  geom_point(mapping = aes(x=Treatment, y=yvar, shape = Type, col=Type)) + labs(x =
```

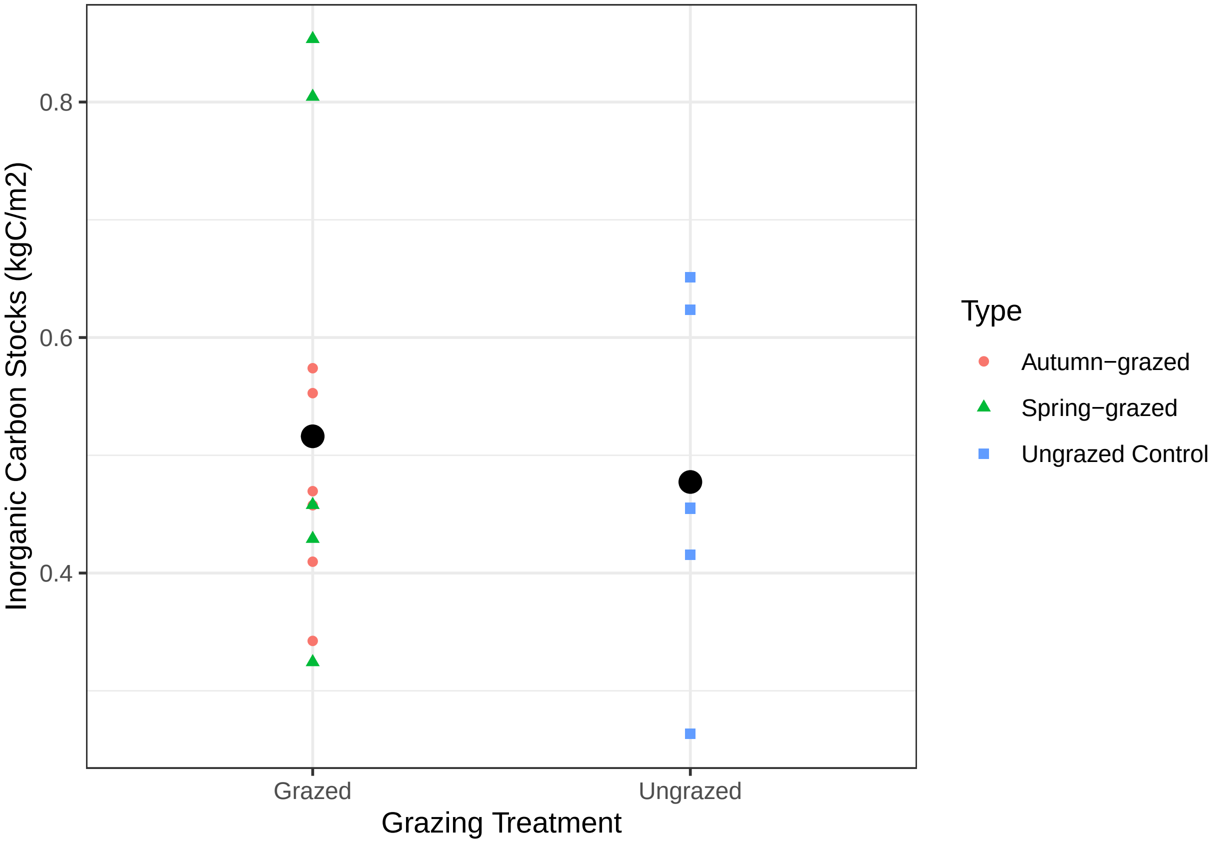


```
ggplot(Inorganic_C, aes(x=Type, y=Inorganic.C.stocks..kgC.m2.)) + geom_boxplot(trim=FALSE) + labs(x = "(
```

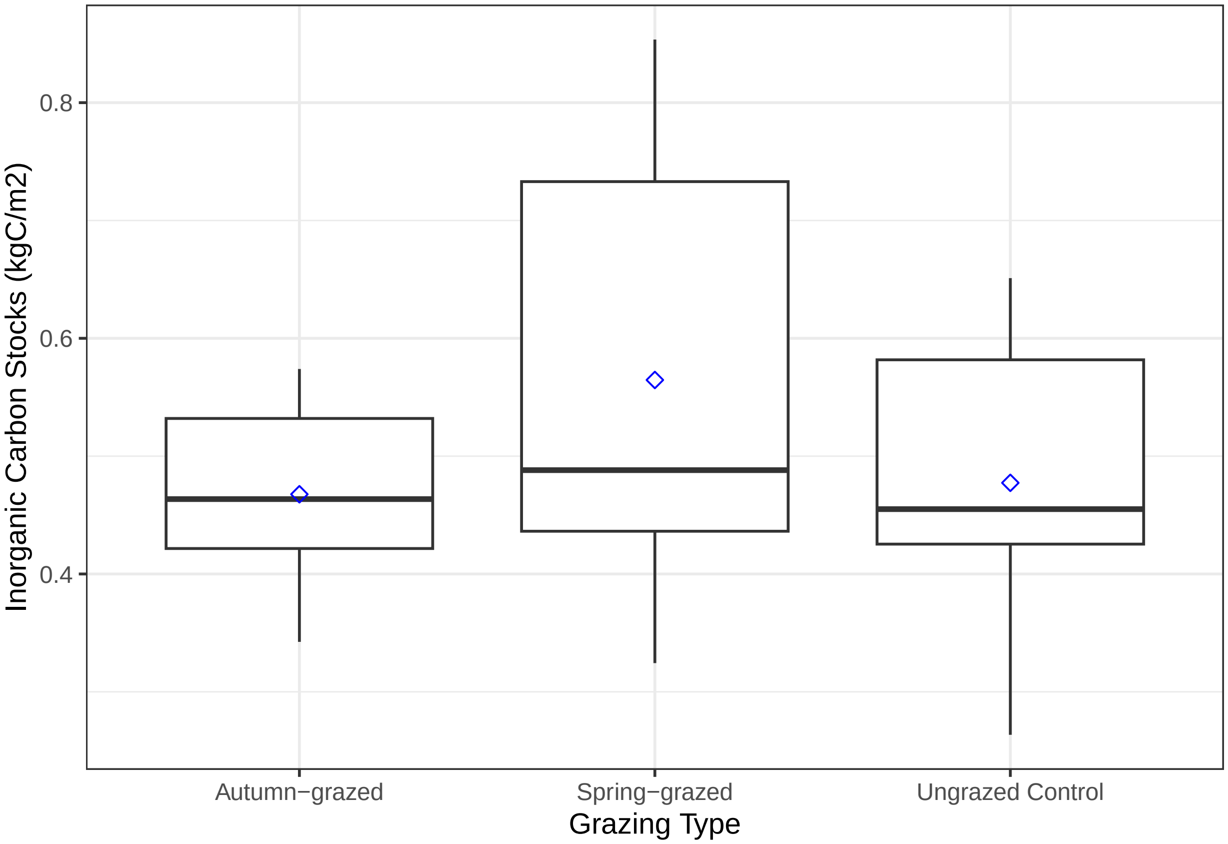


```
# Calculating 95% confidence intervals
UngrazedInorganic_C <- Inorganic_C[c(1,2,3,10,11,12),]
1.96 * sd(UngrazedInorganic_C$Inorganic.C.stocks..kgC.m2.)/sqrt(6)
```

```
## [1] 0.1143815
```

```
SpringInorganic_C <- Inorganic_C[c(7,8,9,16,17,18),]
1.96 * sd(SpringInorganic_C$Inorganic.C.stocks..kgC.m2.)/sqrt(6)
```

```
## [1] 0.1718671
```

```
AutumnInorganic_C <- Inorganic_C[c(4,5,6,13,14,15),]
1.96 * sd(AutumnInorganic_C$Inorganic.C.stocks..kgC.m2.)/sqrt(6)
```

```
## [1] 0.06949511
```

```
GrazedInorganic_C <- Inorganic_C[c(4,5,6,7,8,9,13,14,15,16,17,18),]
1.96 * sd(GrazedInorganic_C$Inorganic.C.stocks..kgC.m2.)/sqrt(6)
```

```
## [1] 0.1313937
```

Visual conclusions: inorganic C is slightly higher in grazed treatment, but unlikely to be significant. Grazing type doesn't seem to have an effect. Important to note that variability is higher in the sprin-grazed than in the autumn-grazed or ungrazed control.

Mixed Effects Model looking at the effects of just grazing type (Spring vs Autumn vs Ungrazed Control):

```
Inorganic_CModel1 <- lme(Inorganic.C.stocks..kgC.m2. ~ Type, random = ~ 1|Block, data = Inorganic_C)
summary(Inorganic_CModel1)
```

```
## Linear mixed-effects model fit by REML
```

```
##   Data: Inorganic_C
##        AIC      BIC   logLik
##   2.431444 5.971695 3.784278
##
## Random effects:
##  Formula: ~1 | Block
##         (Intercept)  Residual
## StdDev: 1.756365e-06 0.1571743
##
## Fixed effects:  Inorganic.C.stocks..kgC.m2. ~ Type
##                           Value  Std.Error DF  t-value
## (Intercept)           0.4676415 0.06416615 14 7.287977
## TypeSpring-grazed     0.0969870 0.09074464 14 1.068790
## TypeUngrazed Control  0.0097043 0.09074464 14 0.106940
##                       p-value
## (Intercept)            0.0000
## TypeSpring-grazed      0.3032
## TypeUngrazed Control   0.9164
##  Correlation:
##                       (Intr) TypSp-
## TypeSpring-grazed    -0.707
## TypeUngrazed Control -0.707  0.500
##
## Standardized Within-Group Residuals:
##        Min         Q1        Med         Q3        Max
## -1.5286075 -0.6075197 -0.1420400  0.6427736  1.8383067
##
## Number of Observations: 18
## Number of Groups: 2
```

```
anova(Inorganic_CModel1)
```

```
##             numDF denDF   F-value p-value
## (Intercept)     1    14 184.50133  <.0001
## Type            2    14   0.69297  0.5165
```

```
plot(Inorganic_CModel1)
```

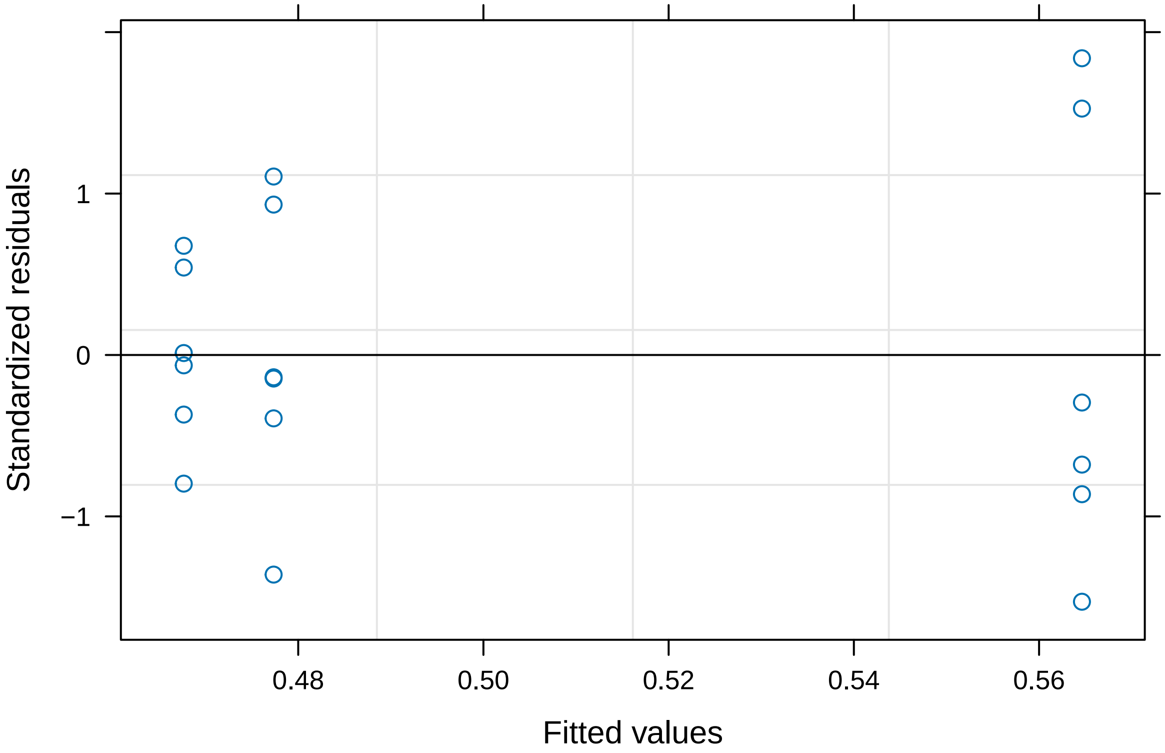


```r
qqnorm(Inorganic_CModel1$residuals)
```

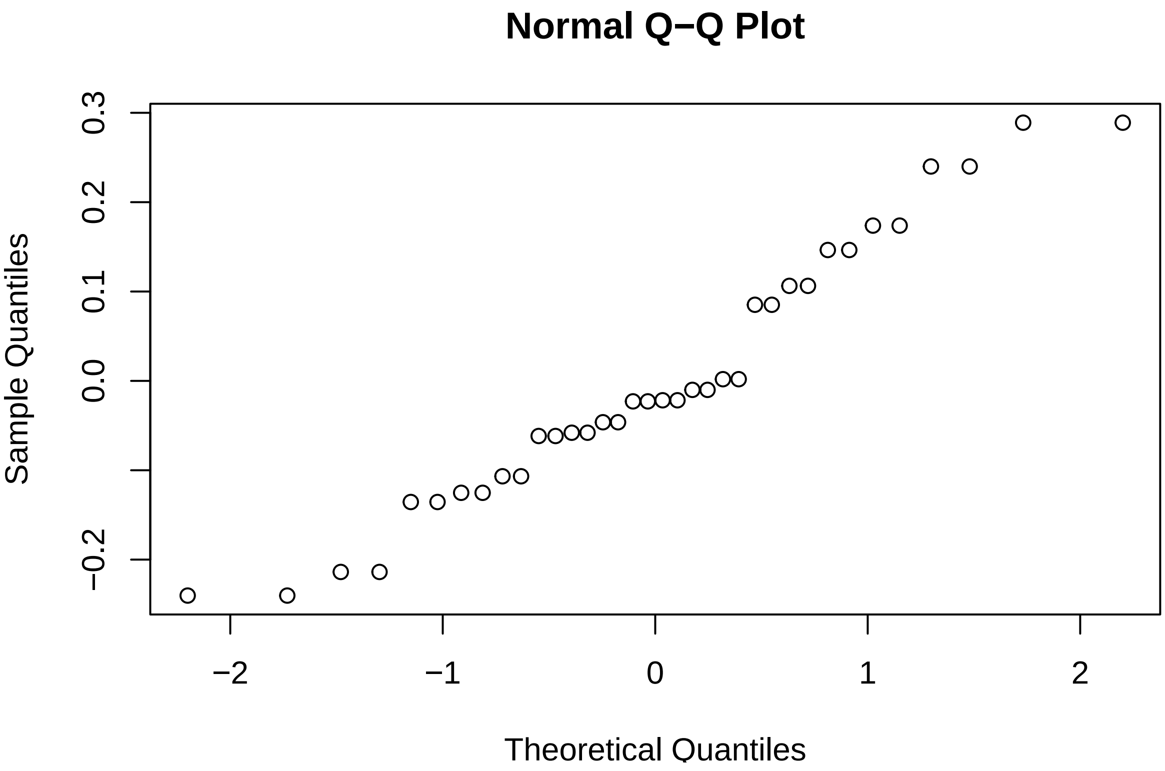


```r
## Changing order to get last comparison
levels(Inorganic_C$Type)
```

```
## NULL
```

```r
Inorganic_C$Type <- factor(Inorganic_C$Type, levels=c('Autumn-grazed', 'Spring-grazed', 'Ungrazed Contr
Inorganic_CModel2 <- lme(Inorganic.C.stocks..kgC.m2. ~ Type, random = ~ 1|Block, data = Inorganic_C)
summary(Inorganic_CModel2)
```

```
## Linear mixed-effects model fit by REML
##   Data: Inorganic_C
##        AIC      BIC   logLik
##   2.431444 5.971695 3.784278
##
## Random effects:
##  Formula: ~1 | Block
##          (Intercept)  Residual
## StdDev: 1.756365e-06 0.1571743
##
## Fixed effects:  Inorganic.C.stocks..kgC.m2. ~ Type
##                           Value  Std.Error DF  t-value
## (Intercept)           0.4676415 0.06416615 14 7.287977
## TypeSpring-grazed     0.0969870 0.09074464 14 1.068790
## TypeUngrazed Control 0.0097043 0.09074464 14 0.106940
##                       p-value
## (Intercept)            0.0000
## TypeSpring-grazed      0.3032
## TypeUngrazed Control   0.9164
##  Correlation:
##                       (Intr) TypSp-
## TypeSpring-grazed    -0.707
## TypeUngrazed Control -0.707  0.500
##
## Standardized Within-Group Residuals:
##        Min         Q1        Med         Q3        Max
## -1.5286075 -0.6075197 -0.1420400  0.6427736  1.8383067
##
## Number of Observations: 18
## Number of Groups: 2
```

```r
anova(Inorganic_CModel2)
```

```
##             numDF denDF   F-value p-value
## (Intercept)     1    14 184.50133  <.0001
## Type            2    14   0.69297  0.5165
```

```r
plot(Inorganic_CModel2)
```

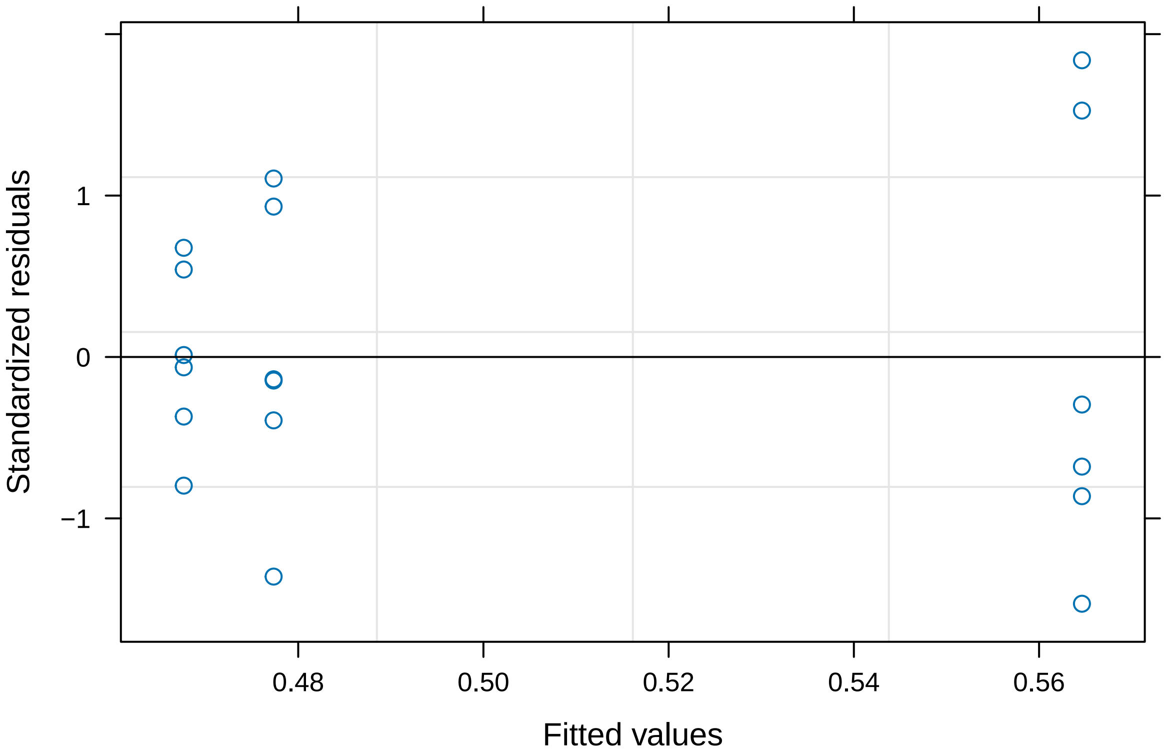


```
qqnorm(Inorganic_CModel2$residuals)
```

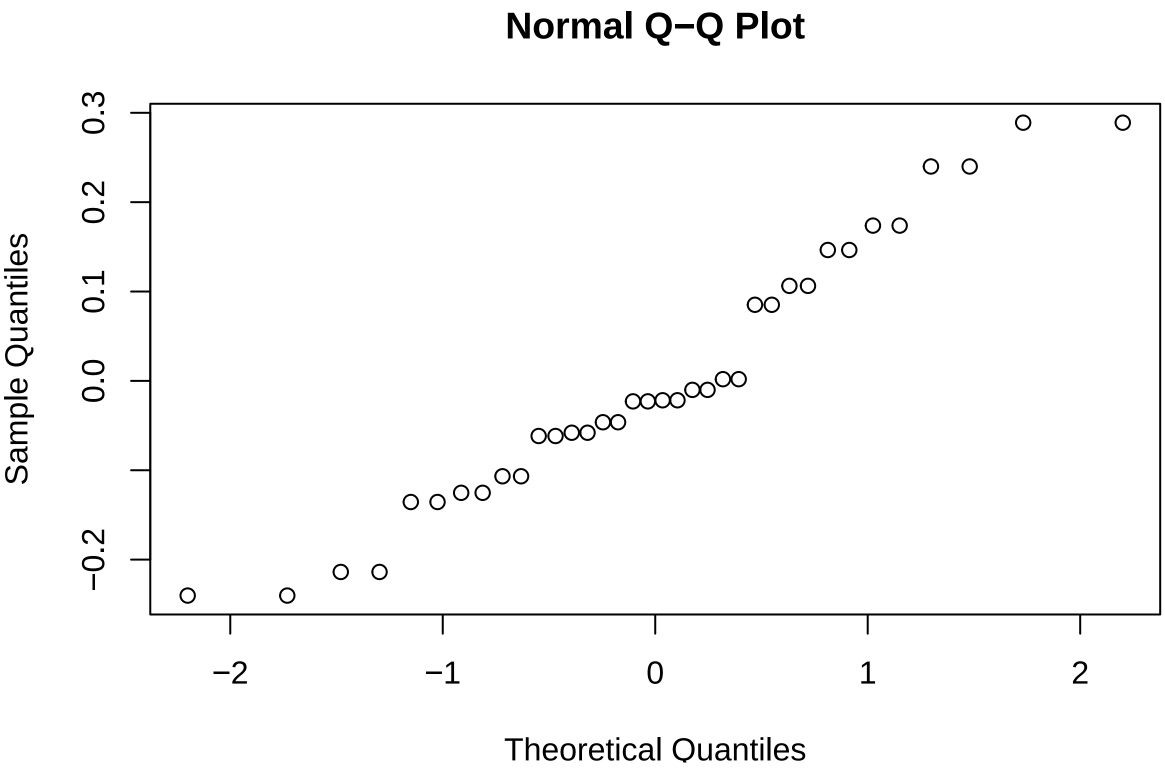

Meets assumptions fine. Suggests grazing type does not influence inorganic C content (F2,14 = 0.693, p = 0.517).

Mixed effects model looking at the effect of just Treatment (grazed vs ungrazed):

```r
Inorganic_CModel3 <- lme(Inorganic.C.stocks..kgC.m2. ~ Treatment, random = ~ 1|Block, data = Inorganic_C
summary(Inorganic_CModel3)
```

```
## Linear mixed-effects model fit by REML
##   Data: Inorganic_C
##         AIC      BIC   logLik
##   -1.388407 1.701948 4.694204
##
## Random effects:
##  Formula: ~1 | Block
##          (Intercept)  Residual
## StdDev: 1.764351e-06 0.1578718
##
## Fixed effects:  Inorganic.C.stocks..kgC.m2. ~ Treatment
##                         Value  Std.Error DF   t-value
## (Intercept)         0.5161349 0.04557366 15 11.325291
## TreatmentUngrazed -0.0387892 0.07893589 15 -0.491402
##                    p-value
## (Intercept)         0.0000
## TreatmentUngrazed  0.6303
##  Correlation:
##                    (Intr)
## TreatmentUngrazed -0.577
##
## Standardized Within-Group Residuals:
##        Min         Q1        Med         Q3        Max
## -1.3545136 -0.5112575 -0.2202274  0.3327639  2.1373555
##
## Number of Observations: 18
## Number of Groups: 2
```

```r
anova(Inorganic_CModel3)
```

```
##             numDF denDF   F-value p-value
## (Intercept)     1    15 182.87474  <.0001
## Treatment       1    15   0.24148  0.6303
```

```r
plot(Inorganic_CModel3)
```

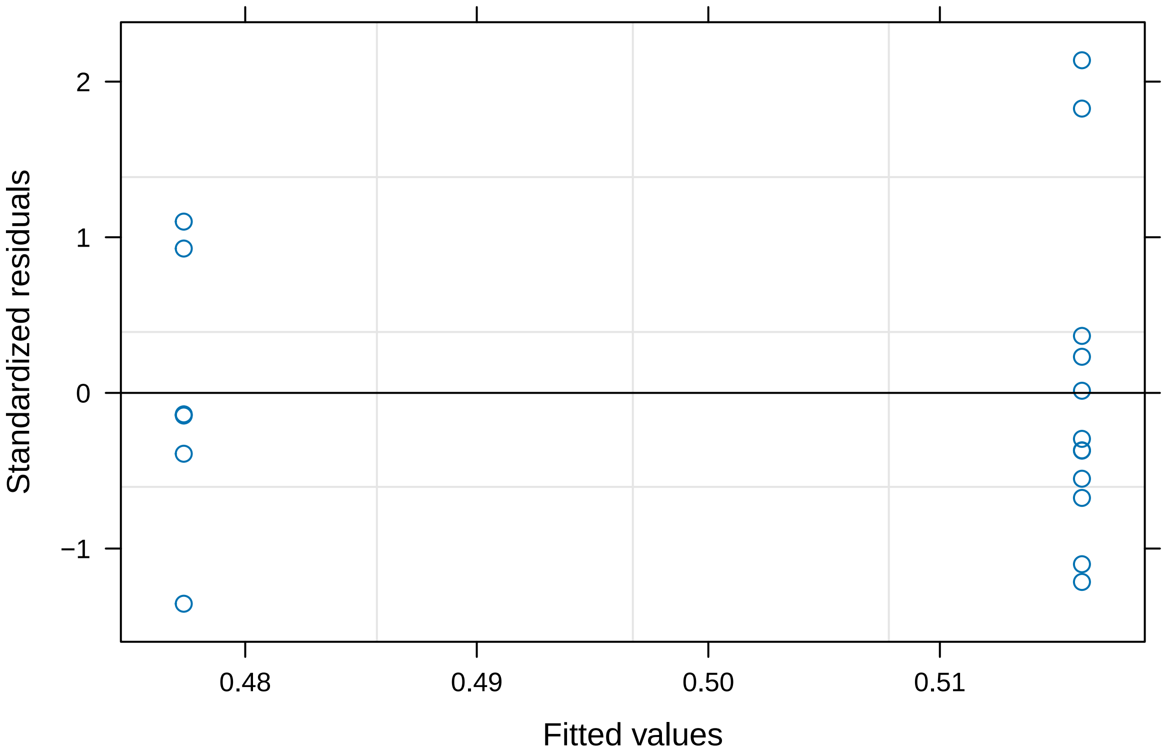


```r
qqnorm(Inorganic_CModel3$residuals)
```

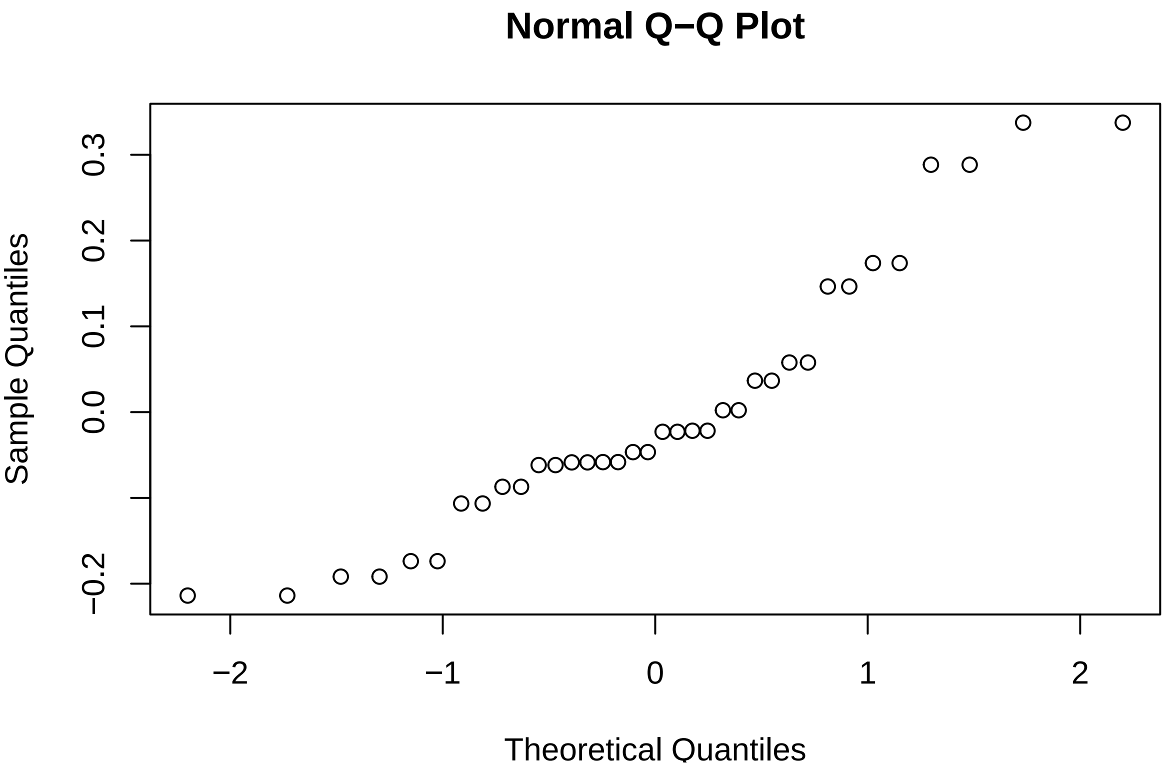

Meets assumptions fine. Suggests grazing does not influence inorganic C content ($F1,15 = 0.241$, $p = 0.630$), which is the accurate conclusion given the non-significance of the effects of underlying grazing type (above).

Total Carbon Stocks

```r
TotalC <- completedata2 %>% dplyr::select(Block, ID, Type, Treatment, Study, Total.Carbon.stocks..kgC.m
yvar <- TotalC$Total.Carbon.stocks..kgC.m2.

TotalCTreatment_means <- TotalC %>% group_by(Treatment) %>% summarise(Treatment_mean_Total.Carbon.stock
TotalCTreatment_means
```

```
## # A tibble: 2 x 2
##   Treatment Treatment_mean_Total.Carbon.stocks..kgC.m2.
##   <chr>                                           <dbl>
## 1 Grazed                                           4.17
## 2 Ungrazed                                         4.16
```

```r
TotalCType_means <- TotalC %>% group_by(Type) %>% summarise(Type_mean_Total.Carbon.stocks..kgC.m2. = mea
TotalCType_means
```

```
## # A tibble: 3 x 2
##   Type             Type_mean_Total.Carbon.stocks..kgC.m2.
##   <chr>                                             <dbl>
## 1 Autumn-grazed                                      4.02
## 2 Spring-grazed                                      4.31
## 3 Ungrazed Control                                   4.16
```

```r
ggplot(TotalC) +  geom_point(mapping = aes(x=Treatment, y=yvar, shape = Type, col=Type)) + labs(x = "Gr
```

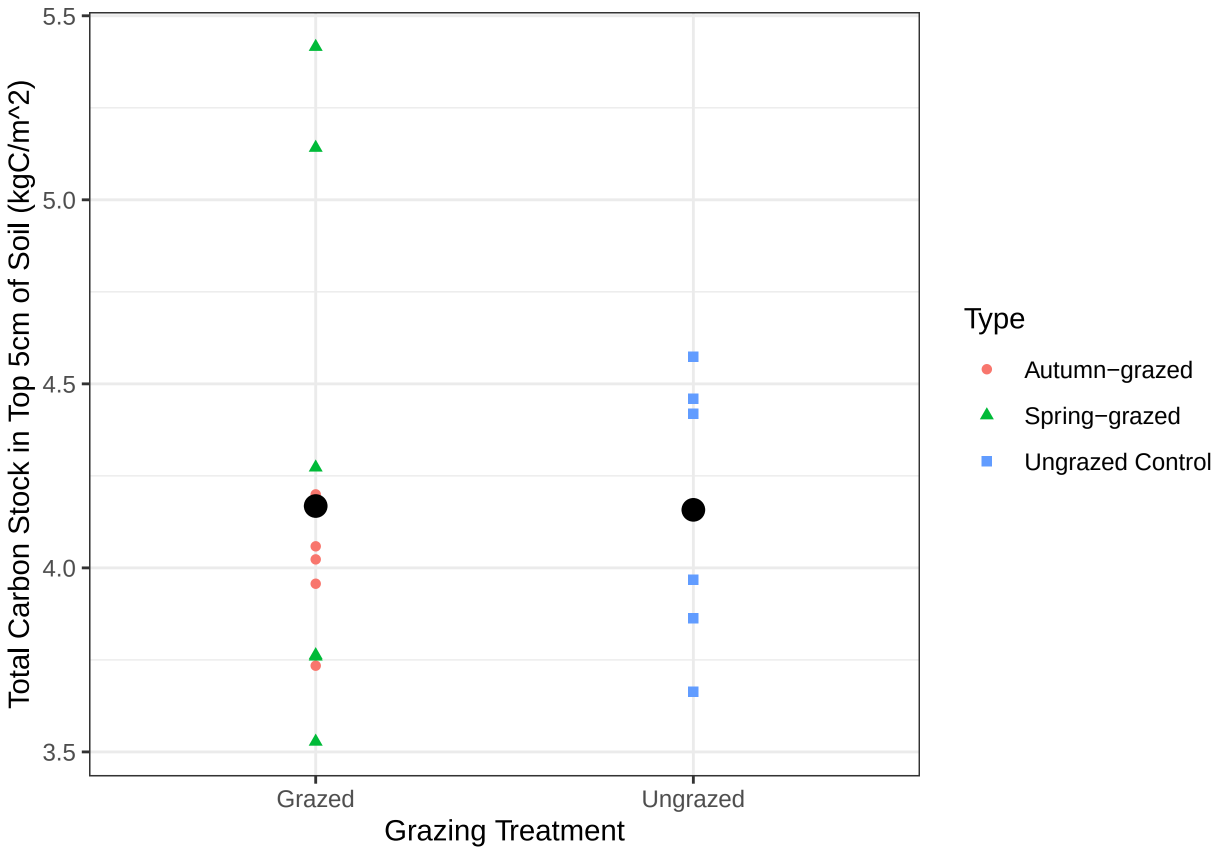


```r
ggplot(TotalC, aes(x=Type, y=Total.Carbon.stocks..kgC.m2.)) + geom_boxplot(trim=FALSE) + labs(x = "Graz
```

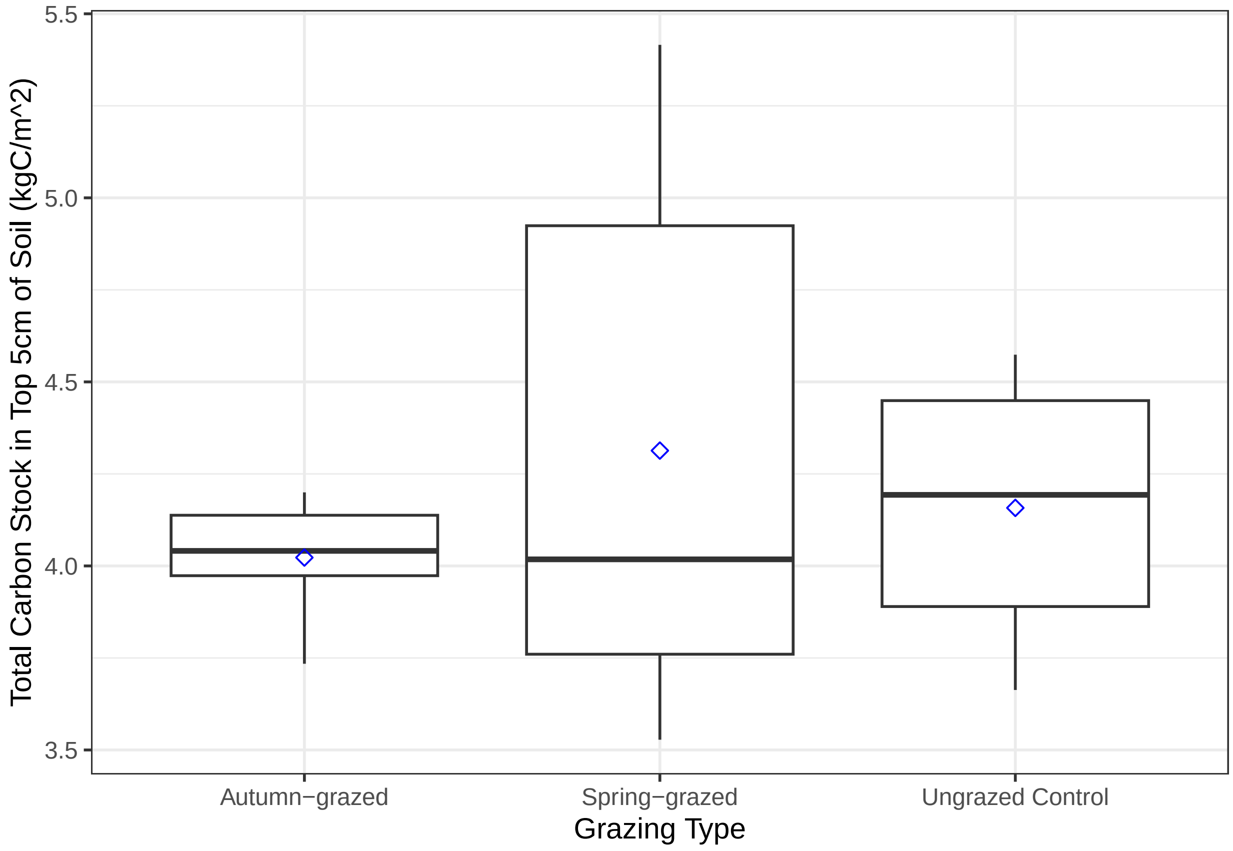


```
# Calculating 95% confidence intervals
UngrazedTotal <- TotalC[c(1,2,3,10,11,12),]
1.96 * sd(UngrazedTotal$Total.Carbon.stocks..kgC.m2.)/sqrt(12)
```

```
## [1] 0.2116518
```

```
SpringTotal <- TotalC[c(7,8,9,16,17,18),]
1.96 * sd(SpringTotal$Total.Carbon.stocks..kgC.m2.)/sqrt(6)
```

```
## [1] 0.6330251
```

```
AutumnTotal <- TotalC[c(4,5,6,13,14,15),]
1.96 * sd(AutumnTotal$Total.Carbon.stocks..kgC.m2.)/sqrt(6)
```

```
## [1] 0.1339711
```

```
GrazedTotal <- TotalC[c(4,5,6,7,8,9,13,14,15,16,17,18),]
1.96 * sd(GrazedTotal$Total.Carbon.stocks..kgC.m2.)/sqrt(12)
```

```
## [1] 0.3201901
```

```
# LME model of effects of type
TotalCModel1 <- lme(Total.Carbon.stocks..kgC.m2. ~ Type, random = ~ 1|Block, data = TotalC)
summary(TotalCModel1)
```

```
## Linear mixed-effects model fit by REML
##   Data: TotalC
##        AIC      BIC    logLik
##   38.00093 41.54118 -14.00047
##
```

```
## Random effects:
##  Formula: ~1 | Block
##         (Intercept)  Residual
## StdDev: 8.197303e-06 0.5144021
##
## Fixed effects:  Total.Carbon.stocks..kgC.m2. ~ Type
##                         Value Std.Error DF   t-value
## (Intercept)          4.022770 0.2100038 14 19.155702
## TypeSpring-grazed    0.290532 0.2969902 14  0.978255
## TypeUngrazed Control 0.134904 0.2969902 14  0.454239
##                      p-value
## (Intercept)           0.0000
## TypeSpring-grazed     0.3446
## TypeUngrazed Control  0.6566
##  Correlation:
##                      (Intr) TypSp-
## TypeSpring-grazed    -0.707
## TypeUngrazed Control -0.707  0.500
##
## Standardized Within-Group Residuals:
##         Min          Q1         Med          Q3
## -1.52682936 -0.56868250 -0.03900059  0.46688907
##         Max
##  2.14289917
##
## Number of Observations: 18
## Number of Groups: 2
```

```r
anova(TotalCModel1)
```

```
##             numDF denDF   F-value p-value
## (Intercept)     1    14 1179.8039  <.0001
## Type            2    14    0.4793   0.629
```

```r
plot(TotalCModel1)
```

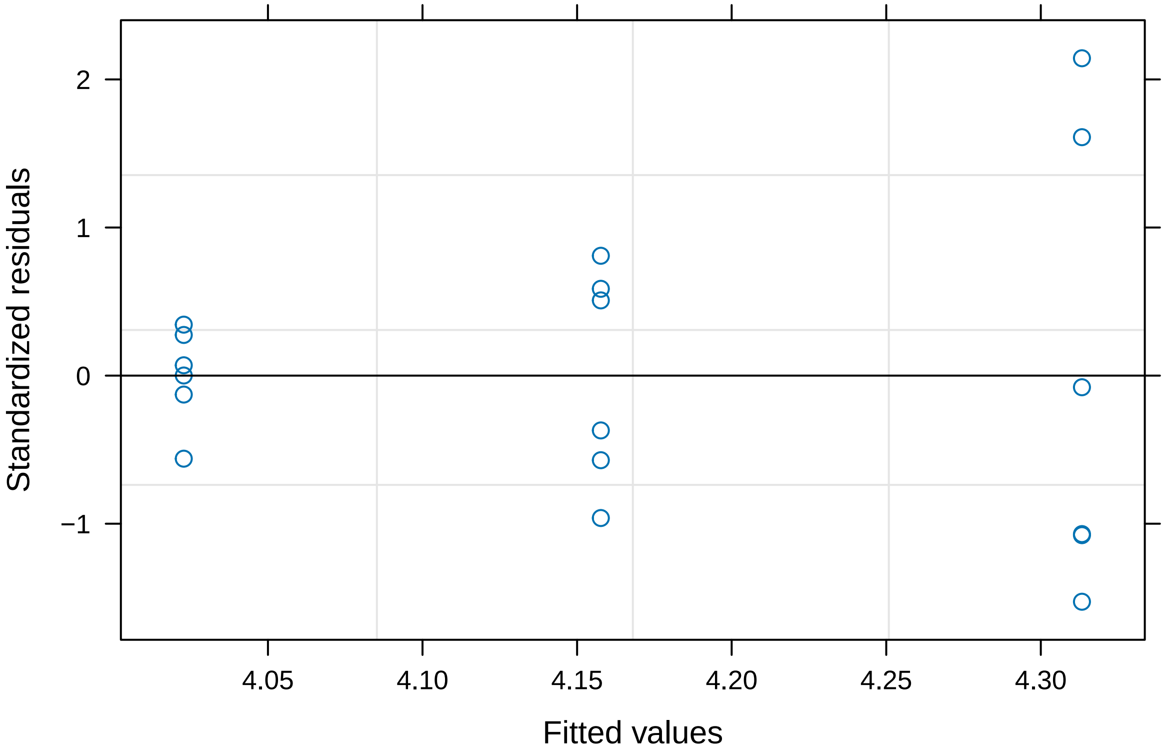


```r
qqnorm(TotalCModel1$residuals)
```

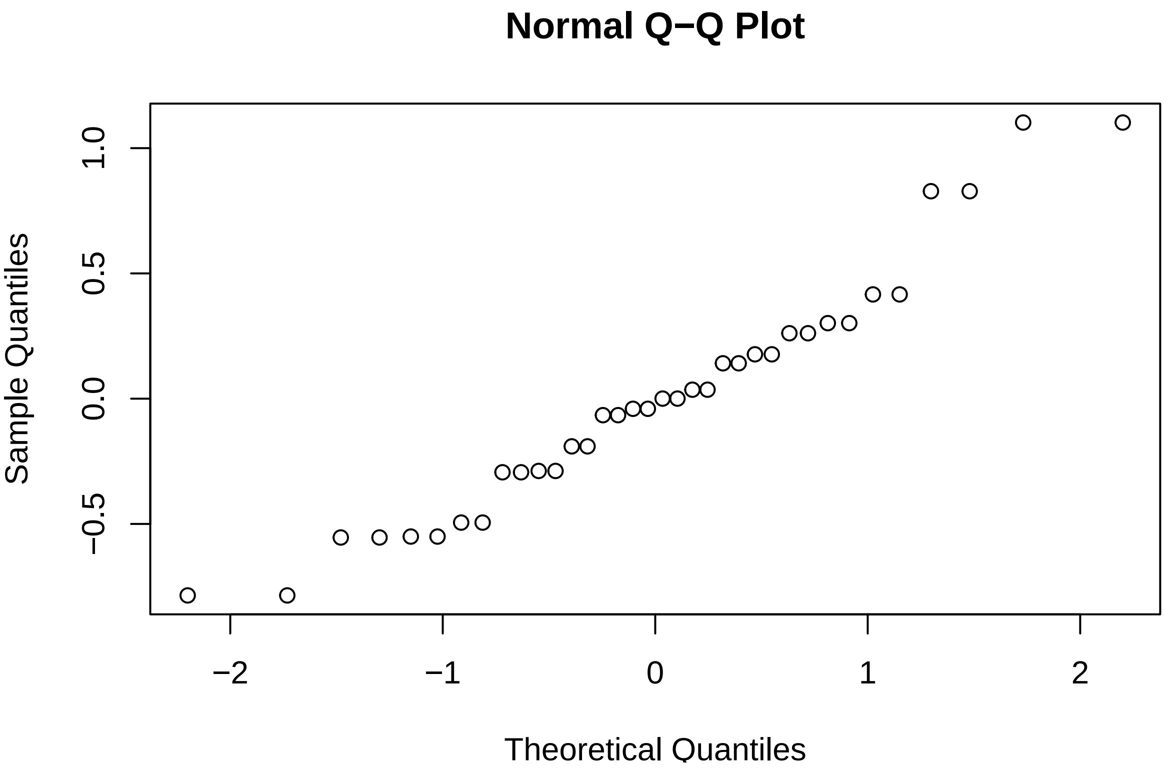


```r
# Transformation
TotalCModel2 <- lme(sqrt(Total.Carbon.stocks..kgC.m2.) ~ Type, random = ~ 1|Block, data = TotalC)
summary(TotalCModel2)
```

```
## Linear mixed-effects model fit by REML
##   Data: TotalC
##         AIC       BIC   logLik
##   -4.898878 -1.358627 7.449439
##
## Random effects:
##  Formula: ~1 | Block
##          (Intercept)  Residual
## StdDev: 1.949005e-06 0.1231018
##
## Fixed effects:  sqrt(Total.Carbon.stocks..kgC.m2.) ~ Type
##                            Value  Std.Error DF  t-value
## (Intercept)          2.0053169 0.05025609 14 39.90197
## TypeSpring-grazed    0.0644568 0.07107285 14  0.90691
## TypeUngrazed Control 0.0319855 0.07107285 14  0.45004
##                      p-value
## (Intercept)           0.0000
## TypeSpring-grazed     0.3798
## TypeUngrazed Control  0.6596
##  Correlation:
##                      (Intr) TypSp-
## TypeSpring-grazed    -0.707
## TypeUngrazed Control -0.707  0.500
##
## Standardized Within-Group Residuals:
##          Min           Q1          Med           Q3
## -1.555655020 -0.589666799 -0.009087521  0.484280562
##          Max
##  2.090736217
##
## Number of Observations: 18
## Number of Groups: 2
```

```
anova(TotalCModel2)
```

```
##             numDF denDF  F-value p-value
## (Intercept)     1    14 4930.873  <.0001
## Type            2    14    0.411  0.6706
```

```
plot(TotalCModel2)
```

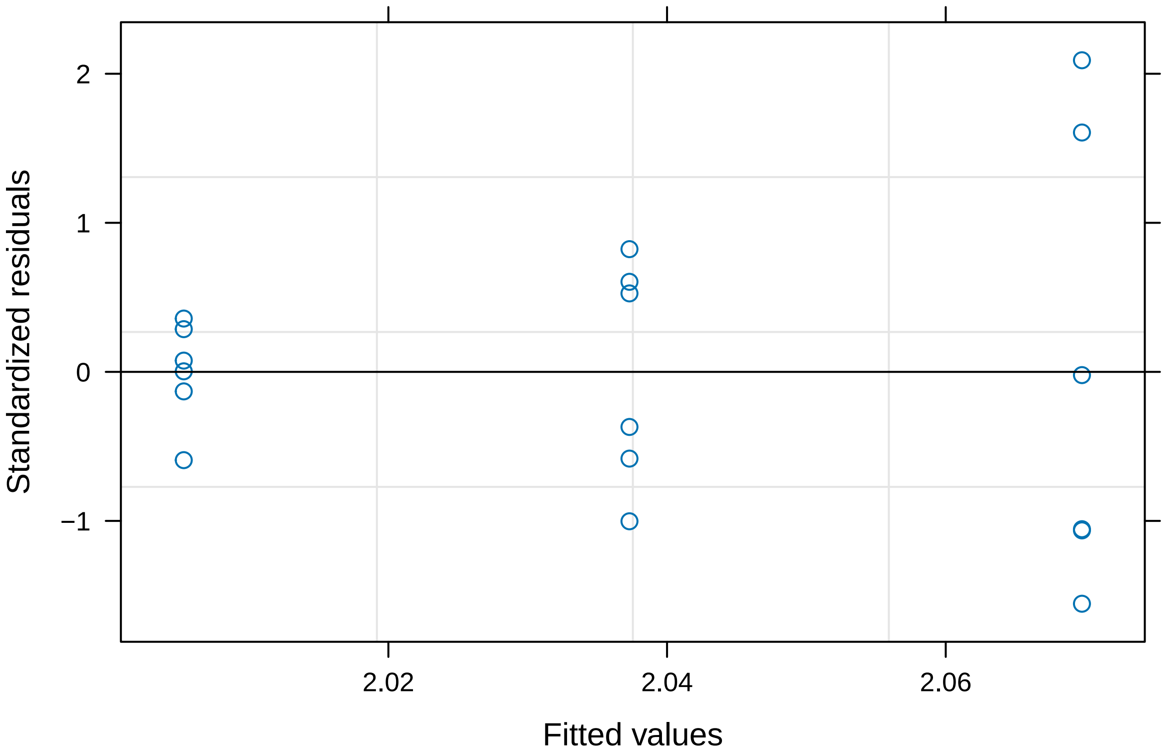


```
qqnorm(TotalCModel2$residuals)
```

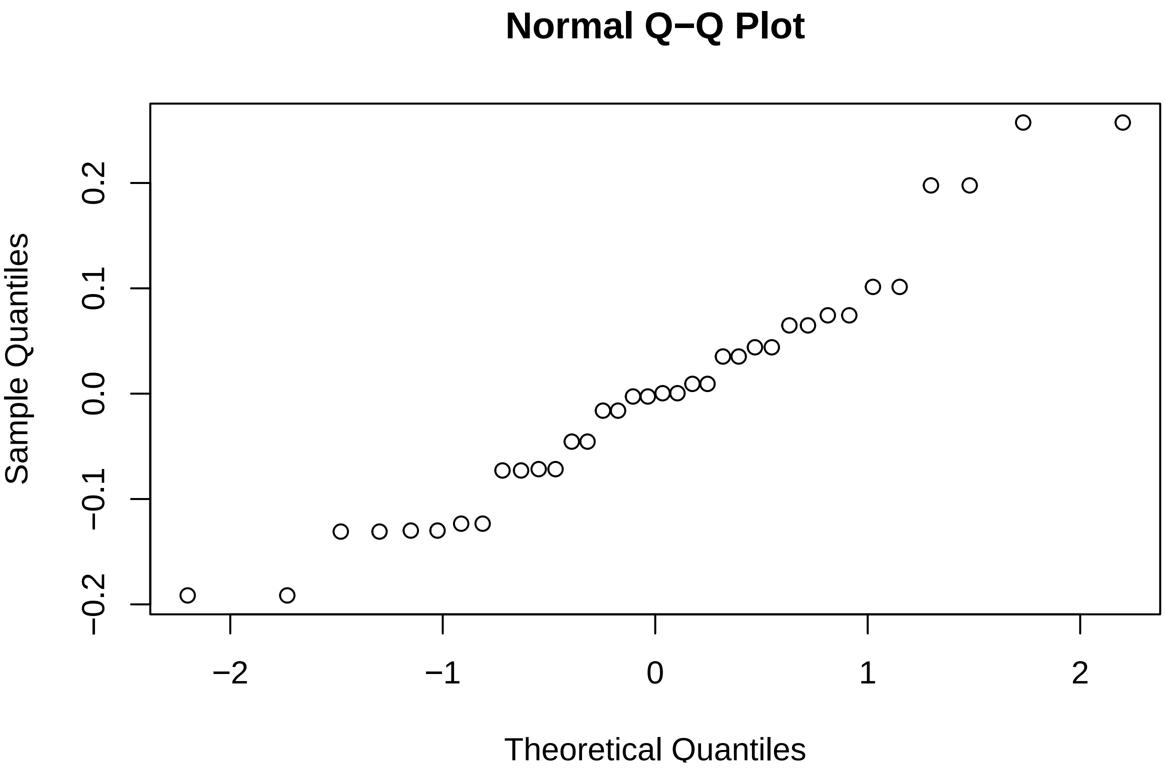


```
# Transformation didn't super work, but can go with sqrt
# Shows that grazing type did not affect total carbon stocks (F2,14 = 0.411, p = 0.671)

# LME looking at effects of grazing treatment alone
```

```r
TotalCModel3 <- lme(Total.Carbon.stocks..kgC.m2. ~ Treatment, random = ~ 1|Block, data = TotalC)
summary(TotalCModel3)
```

```
## Linear mixed-effects model fit by REML
##   Data: TotalC
##        AIC      BIC    logLik
##   36.36762 39.45798 -14.18381
##
## Random effects:
##  Formula: ~1 | Block
##         (Intercept)  Residual
## StdDev: 8.22685e-06 0.5137101
##
## Fixed effects:  Total.Carbon.stocks..kgC.m2. ~ Treatment
##                         Value Std.Error DF  t-value p-value
## (Intercept)          4.168036 0.1482953 15 28.10632  0.0000
## TreatmentUngrazed -0.010362 0.2568551 15 -0.04034  0.9684
##  Correlation:
##                   (Intr)
## TreatmentUngrazed -0.577
##
## Standardized Within-Group Residuals:
##        Min         Q1        Med         Q3        Max
## -1.2461079 -0.7345186 -0.2475999  0.4323799  2.4285639
##
## Number of Observations: 18
## Number of Groups: 2
```

```r
anova(TotalCModel3)
```

```
##             numDF denDF   F-value p-value
## (Intercept)     1    15 1182.9845  <.0001
## Treatment       1    15    0.0016  0.9684
```

```r
plot(TotalCModel3)
```

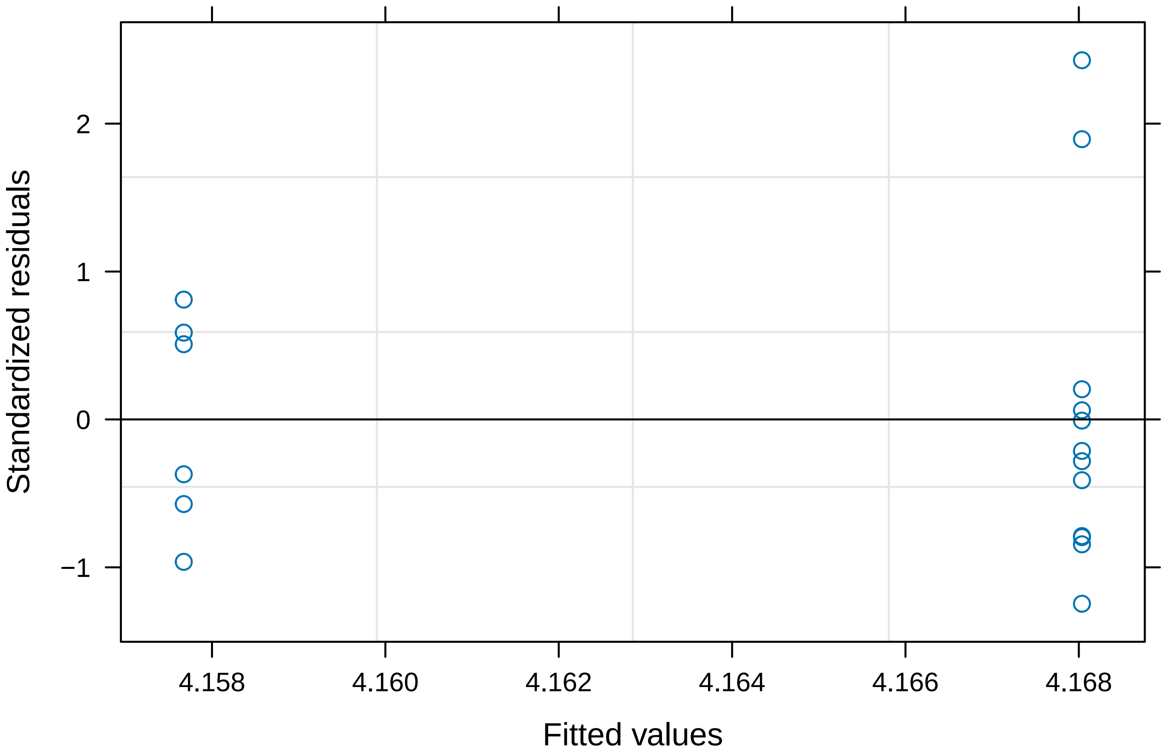


```
qqnorm(TotalCModel3$residuals)
```

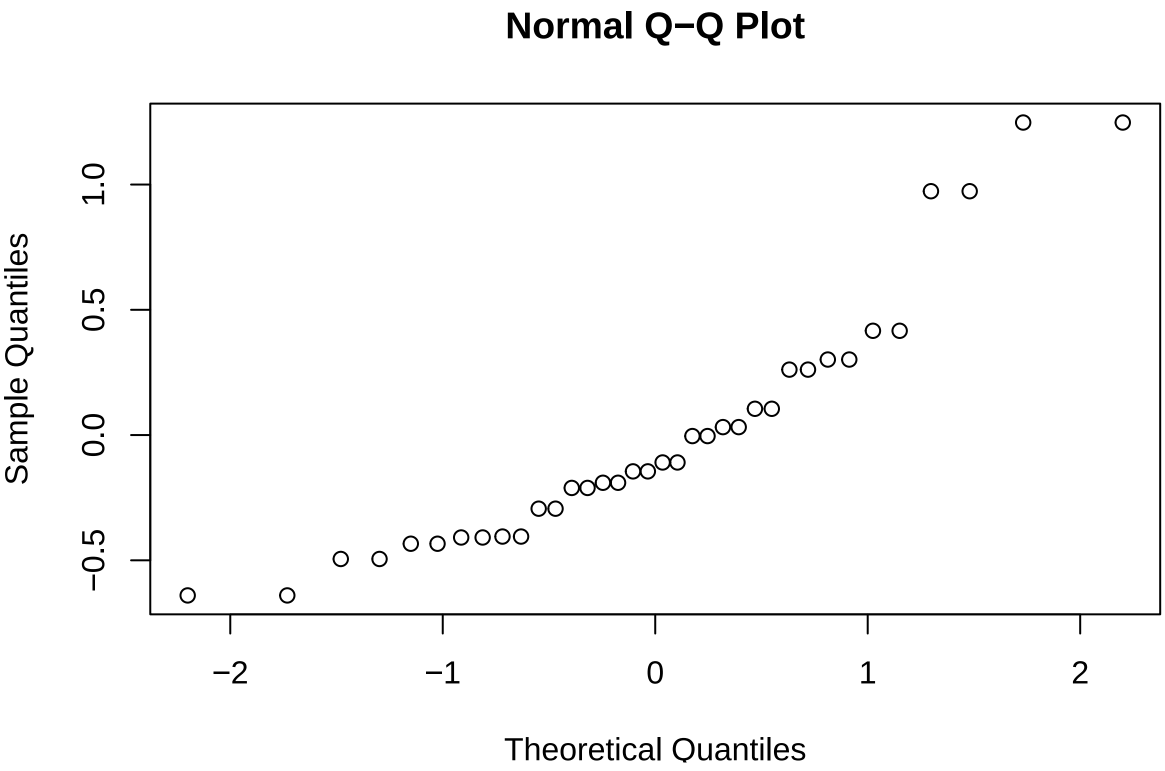


```
# Transformation
TotalCModel4 <- lme(log(Total.Carbon.stocks..kgC.m2.) ~ Treatment, random = ~ 1|Block, data = TotalC)
summary(TotalCModel4)
```

```
## Linear mixed-effects model fit by REML
##   Data: TotalC
##         AIC       BIC   logLik
##   -10.94204 -7.851687 9.471021
##
## Random effects:
##  Formula: ~1 | Block
##          (Intercept)  Residual
## StdDev: 1.854795e-06 0.1171239
##
## Fixed effects:  log(Total.Carbon.stocks..kgC.m2.) ~ Treatment
##                         Value  Std.Error DF  t-value
## (Intercept)       1.4196985 0.03381075 15 41.98956
## TreatmentUngrazed 0.0018405 0.05856194 15  0.03143
##                     p-value
## (Intercept)        0.0000
## TreatmentUngrazed  0.9753
##  Correlation:
##                   (Intr)
## TreatmentUngrazed -0.577
##
## Standardized Within-Group Residuals:
##        Min         Q1        Med         Q3        Max
## -1.3575062 -0.7543797 -0.1985527  0.4816129  2.3017315
##
## Number of Observations: 18
## Number of Groups: 2
```

```
anova(TotalCModel4)
```

```
##             numDF denDF  F-value p-value
## (Intercept)     1    15 2646.971  <.0001
## Treatment       1    15    0.001  0.9753
```

```
plot(TotalCModel4)
```

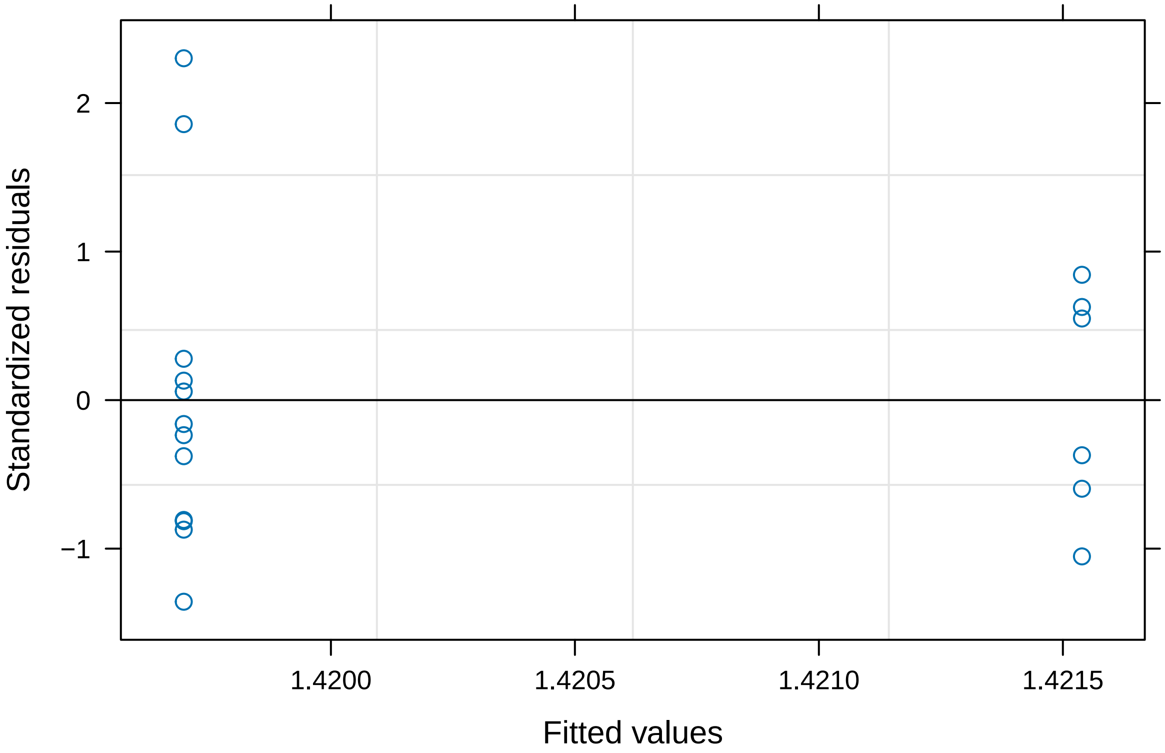


```r
qqnorm(TotalCModel4$residuals)
```

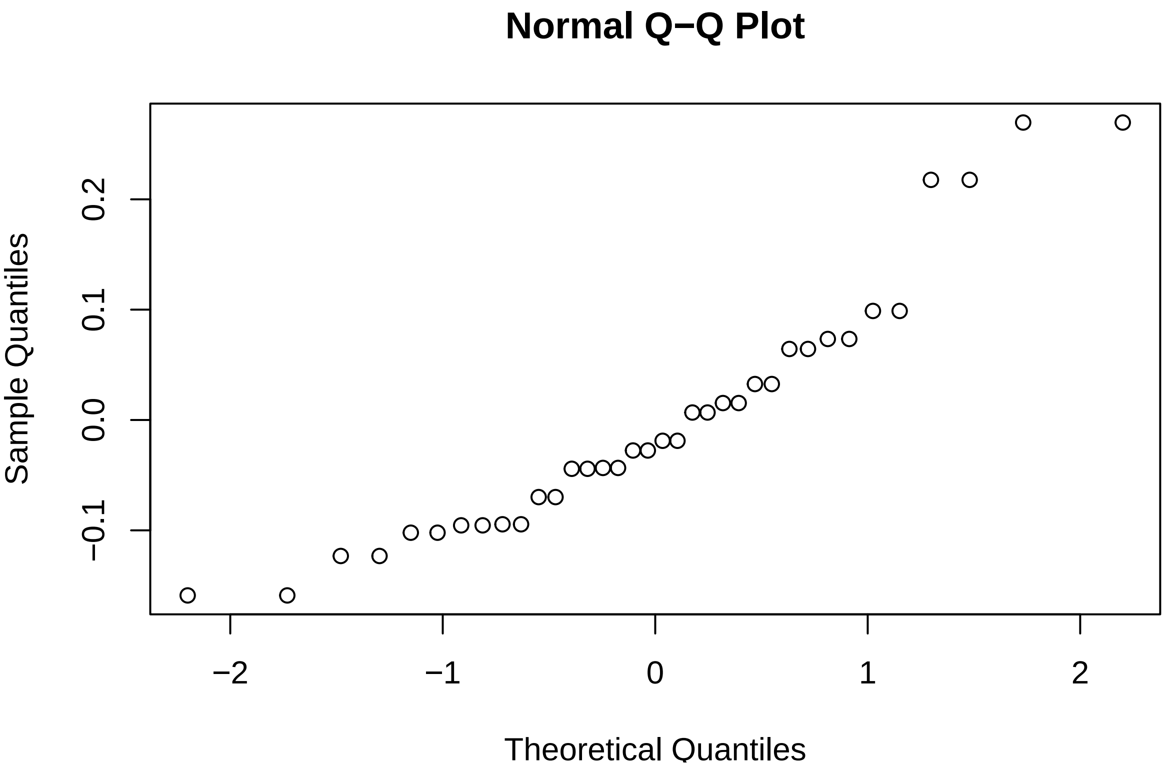


```r
# Log transformation worked, so going with that
# Shows that grazing did not affect total carbon stocks (F1,15 = 0.001, p = 0.975)
```

Load packages

```r
library(ggplot2)
library(cowplot)
```

Make individual plots for grazed vs ungrazed

```r
# Create data frame
MAOMdata <- data.frame(
  Treatment = c("Ungrazed", "Grazed"),
  Mean = c(29.14, 29.2),
  LowerCI = c(26.67, 27.38),
  UpperCI = c(31.61, 31.02)
)
# Create a plot
MAOMplot <- ggplot(MAOMdata, aes(x = Treatment, y = Mean, ymin = LowerCI, ymax = UpperCI)) +
  geom_point() +  # This adds the mean points
  geom_errorbar(width = 0.2) +  # This adds the error bars
  labs(
       y = "MAOM C Stocks (Mg C/ha)") +
  theme_minimal() + theme(axis.title.x = element_blank(), axis.text.x = element_blank(),axis.title.y =

# Display the plot
print(MAOMplot)
```

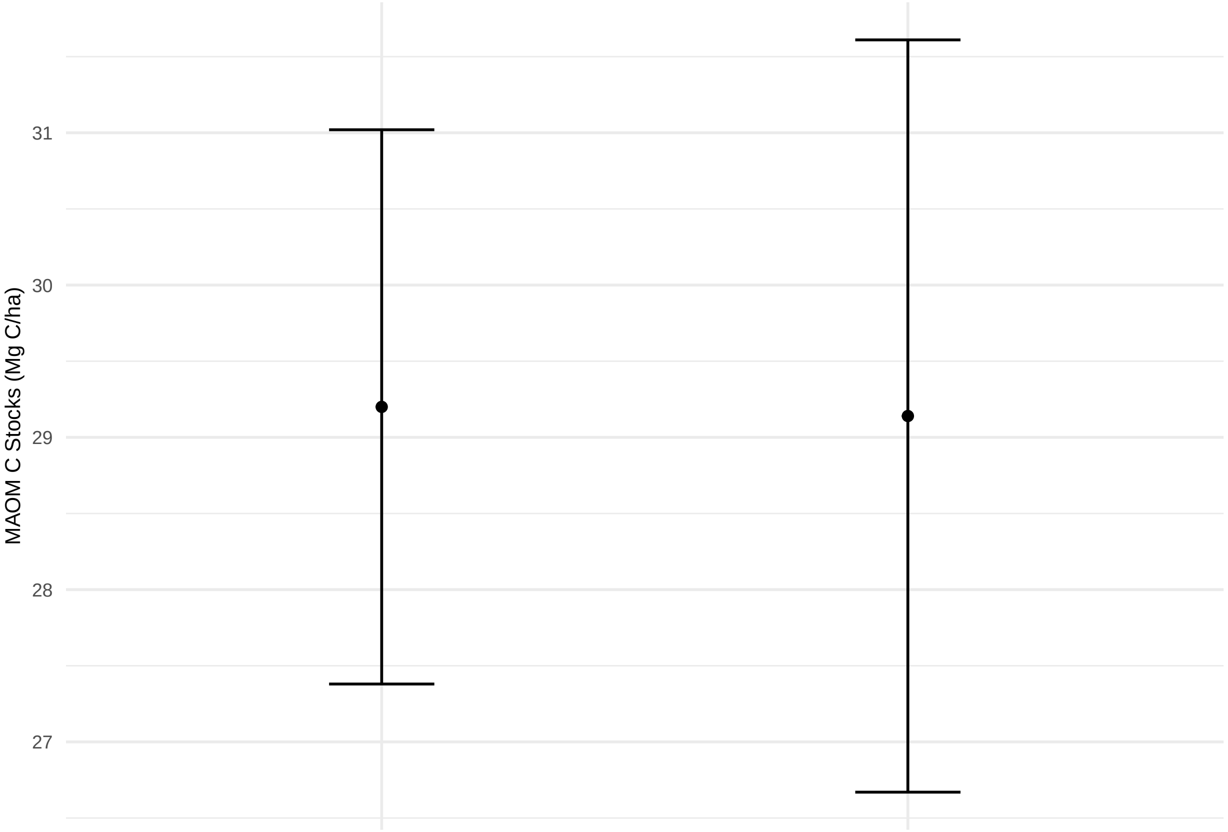


```r
## POM
POMdata <- data.frame(
  Treatment = c("Ungrazed", "Grazed"),
  Mean = c(7.66, 7.32),
  LowerCI = c(6.19, 6.22),
```

```
  UpperCI = c(9.13, 8.42)
)
POMplot <- ggplot(POMdata, aes(x = Treatment, y = Mean, ymin = LowerCI, ymax = UpperCI)) +
  geom_point() +  # This adds the mean points
  geom_errorbar(width = 0.2) +  # This adds the error bars
  labs(
      y = "POM C Stocks (Mg C/ha)") +
  theme_minimal() + theme(axis.title.x = element_blank(), axis.text.x = element_blank(),axis.title.y = e
print(POMplot)
```

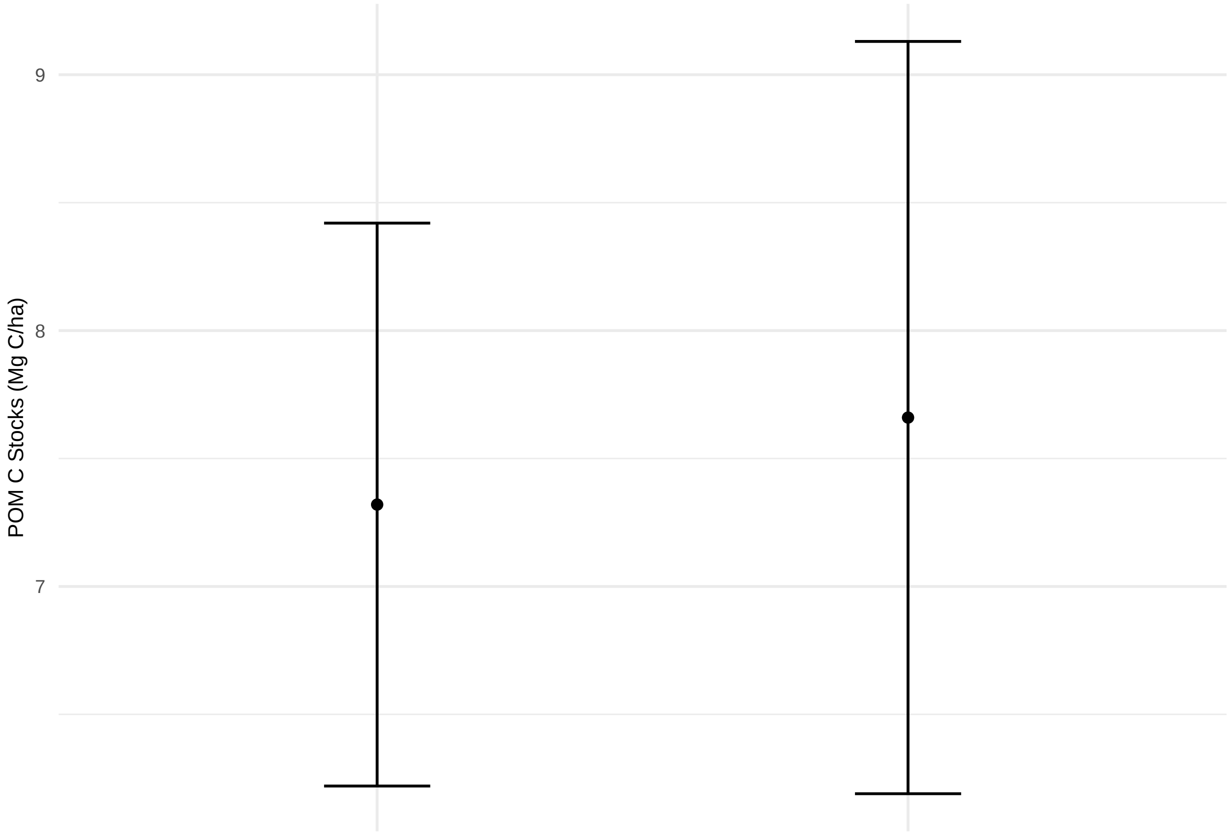


```
## SOC
SOCdata <- data.frame(
  Treatment = c("Ungrazed", "Grazed"),
  Mean = c(36.8, 36.52),
  LowerCI = c(33.69, 33.82),
  UpperCI = c(39.91, 39.22)
)
SOCplot <- ggplot(SOCdata, aes(x = Treatment, y = Mean, ymin = LowerCI, ymax = UpperCI)) +
  geom_point() +  # This adds the mean points
  geom_errorbar(width = 0.2) +  # This adds the error bars
  labs(
      y = "Total SOC Stocks (Mg C/ha)") +
  theme_minimal() + theme(axis.title.x = element_blank(), axis.text.x = element_blank(),axis.title.y = e
print(SOCplot)
```

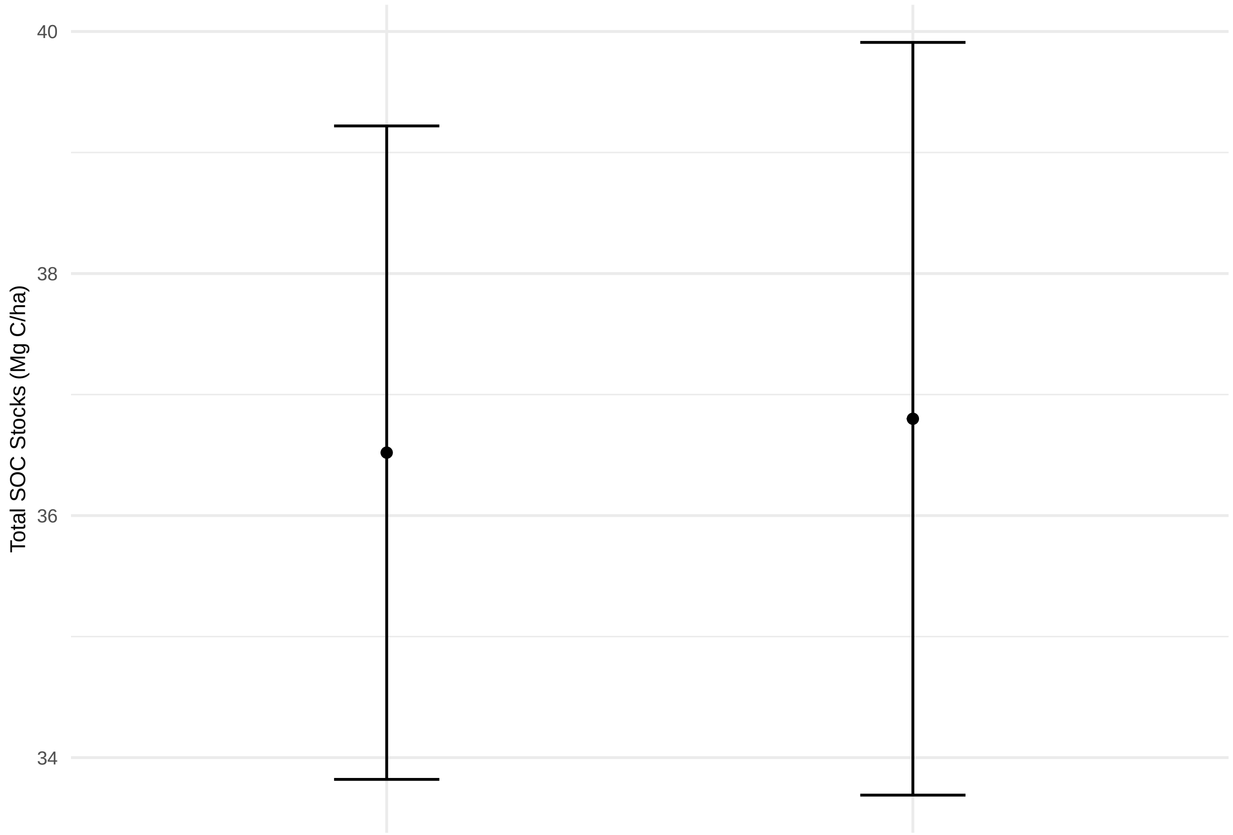


```
## MAOM N
MAOM_N_data <- data.frame(
  Treatment = c("Ungrazed", "Grazed"),
  Mean = c(0.25, 0.24),
  LowerCI = c(0.23, 0.23),
  UpperCI = c(0.28, 0.25)
)
MAOM_N_plot <- ggplot(MAOM_N_data, aes(x = Treatment, y = Mean, ymin = LowerCI, ymax = UpperCI)) +
  geom_point() +  # This adds the mean points
  geom_errorbar(width = 0.2) +  # This adds the error bars
  labs(
      y = "MAOM N Stocks (kg N/m2)") +
  theme_minimal() + theme(axis.title.x = element_blank(), axis.text.x = element_blank(),axis.title.y = e
print(MAOM_N_plot)
```

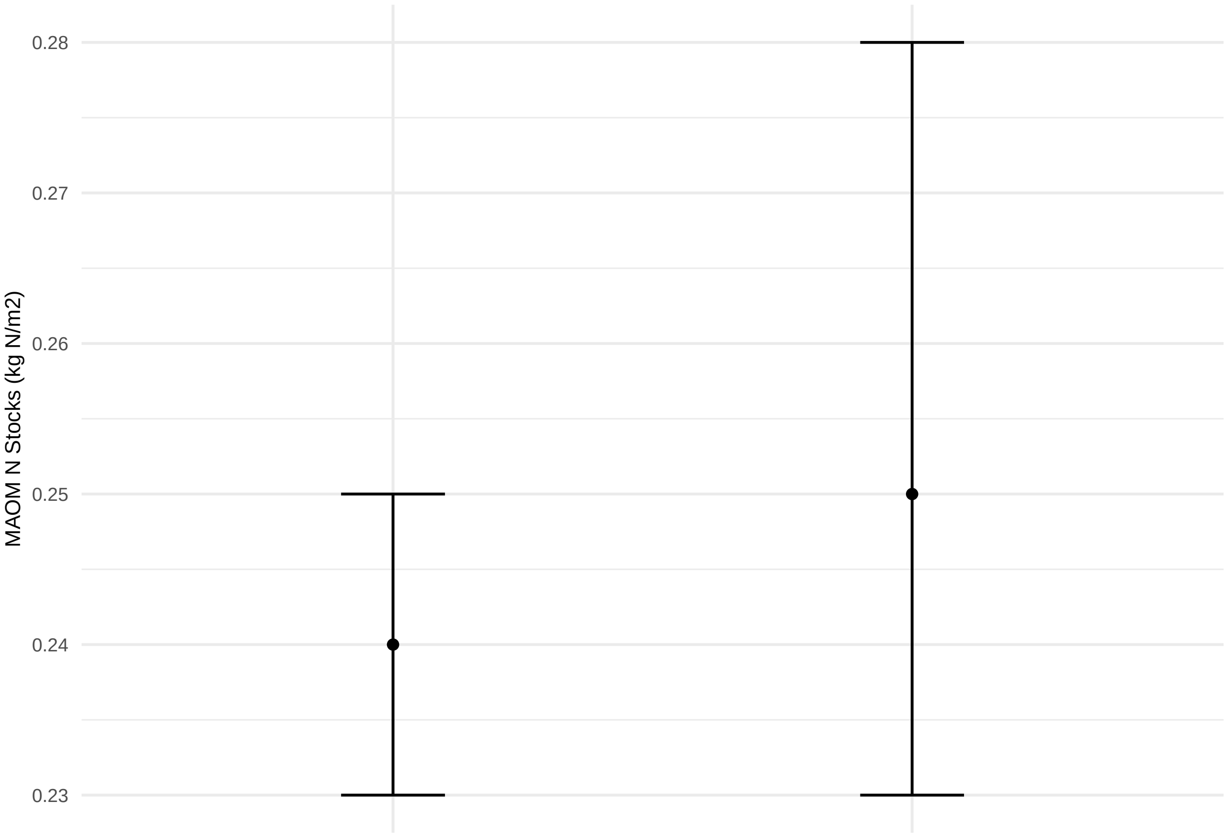


```
## POM N
POM_N_data <- data.frame(
  Treatment = c("Ungrazed", "Grazed"),
  Mean = c(0.016, 0.015),
  LowerCI = c(0.015, 0.013),
  UpperCI = c(0.018, 0.017)
)
POM_N_plot <- ggplot(POM_N_data, aes(x = Treatment, y = Mean, ymin = LowerCI, ymax = UpperCI)) +
  geom_point() +  # This adds the mean points
  geom_errorbar(width = 0.2) +  # This adds the error bars
  labs(
       y = "POM N Stocks (kg N/m2)") +
  theme_minimal() + theme(axis.title.x = element_blank(), axis.text.x = element_blank(),axis.title.y = e
print(POM_N_plot)
```

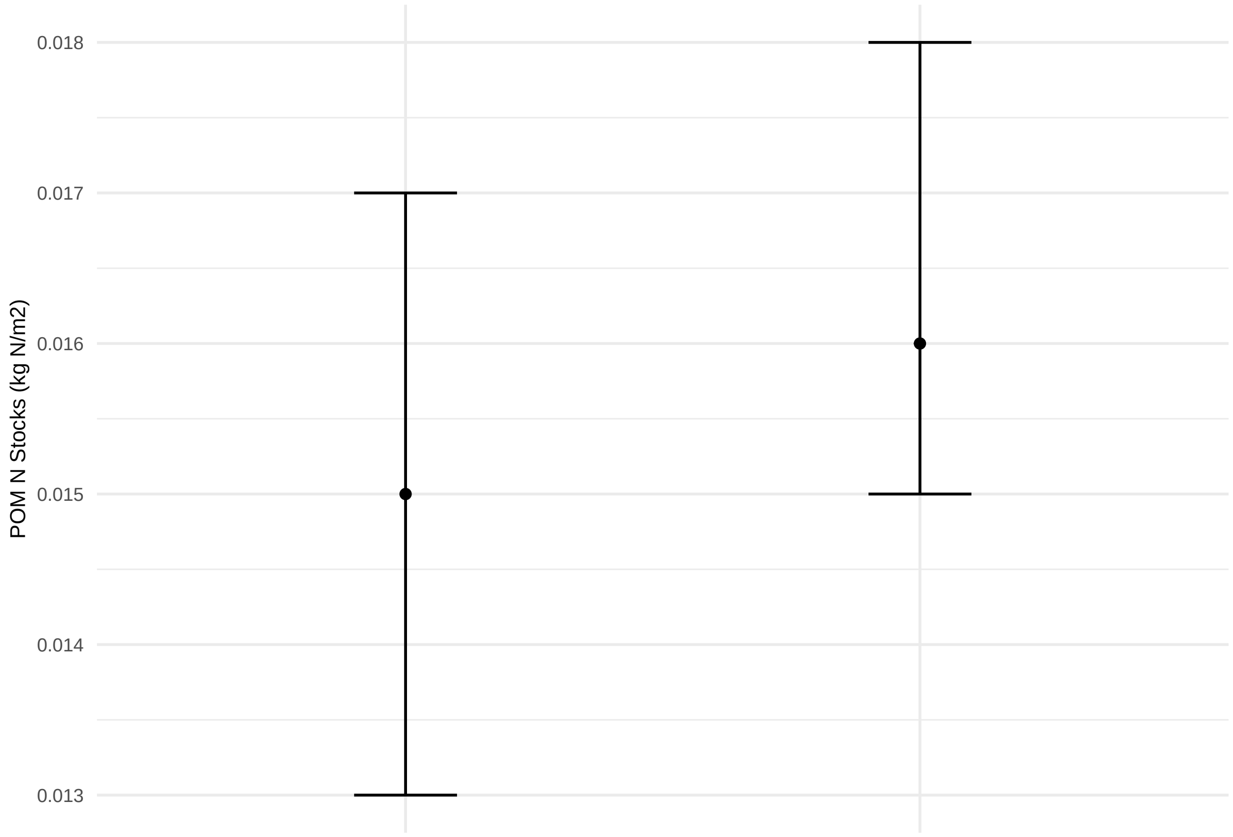


```r
## Total N
Total_N_data <- data.frame(
  Treatment = c("Ungrazed", "Grazed"),
  Mean = c(0.269, 0.257),
  LowerCI = c(0.245, 0.243),
  UpperCI = c(0.293, 0.27)
)
Total_N_plot <- ggplot(Total_N_data, aes(x = Treatment, y = Mean, ymin = LowerCI, ymax = UpperCI)) +
  geom_point() +  # This adds the mean points
  geom_errorbar(width = 0.2) +  # This adds the error bars
  labs(
       y = "Total Organic N (kg N/m^2)") +
  theme_minimal() + theme(axis.title.x = element_blank(), axis.text.x = element_blank(),axis.title.y = e
print(Total_N_plot)
```

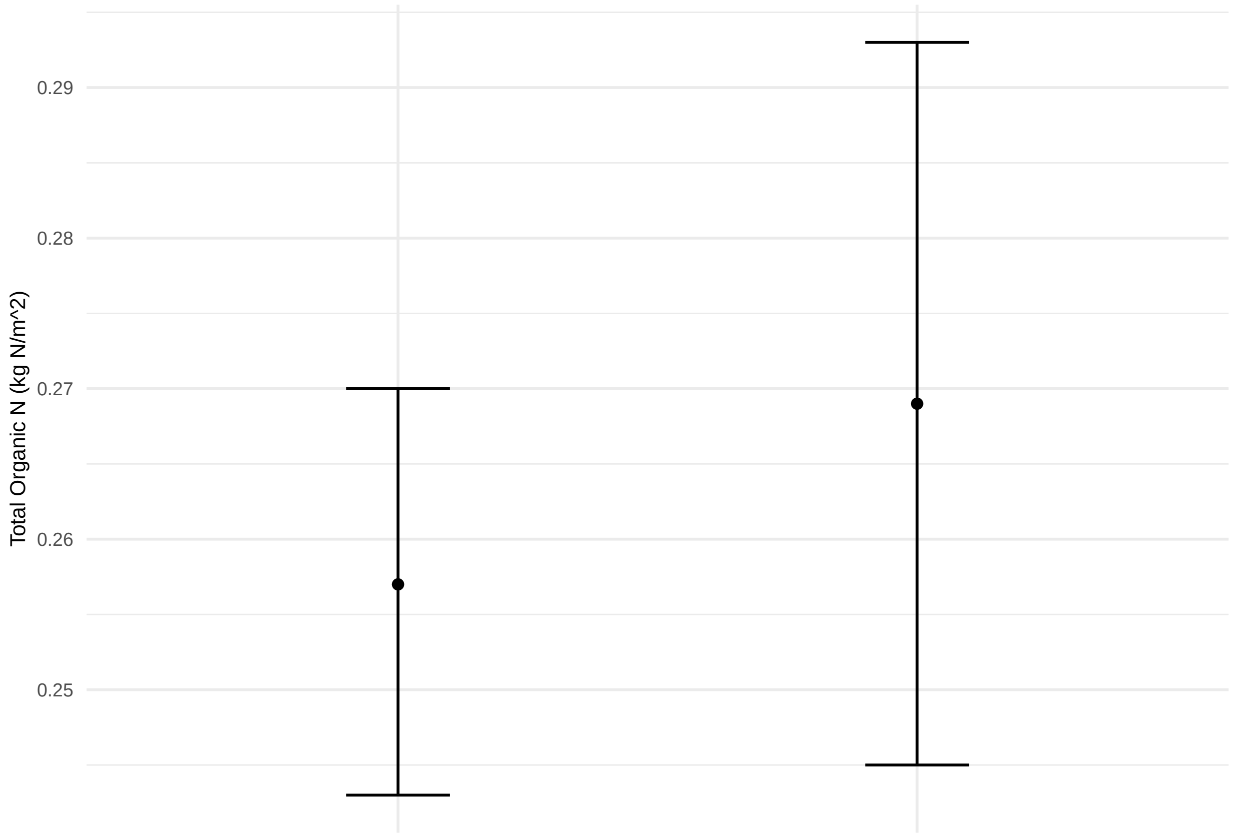


```
## MAOMratio
MAOMratiodata <- data.frame(
  Treatment = c("Ungrazed", "Grazed"),
  Mean = c(11.53, 12.09),
  LowerCI = c(11.06, 11.67),
  UpperCI = c(12.01, 12.50)
)
MAOMRatio_plot <- ggplot(MAOMratiodata, aes(x = Treatment, y = Mean, ymin = LowerCI, ymax = UpperCI)) +
  geom_point() +  # This adds the mean points
  geom_errorbar(width = 0.2) +  # This adds the error bars
  labs(
      y = "MAOM C:N Ratio") +
  theme_minimal() + theme(axis.title.x = element_blank(), axis.text.x = element_blank(),axis.title.y = e
print(MAOMRatio_plot)
```

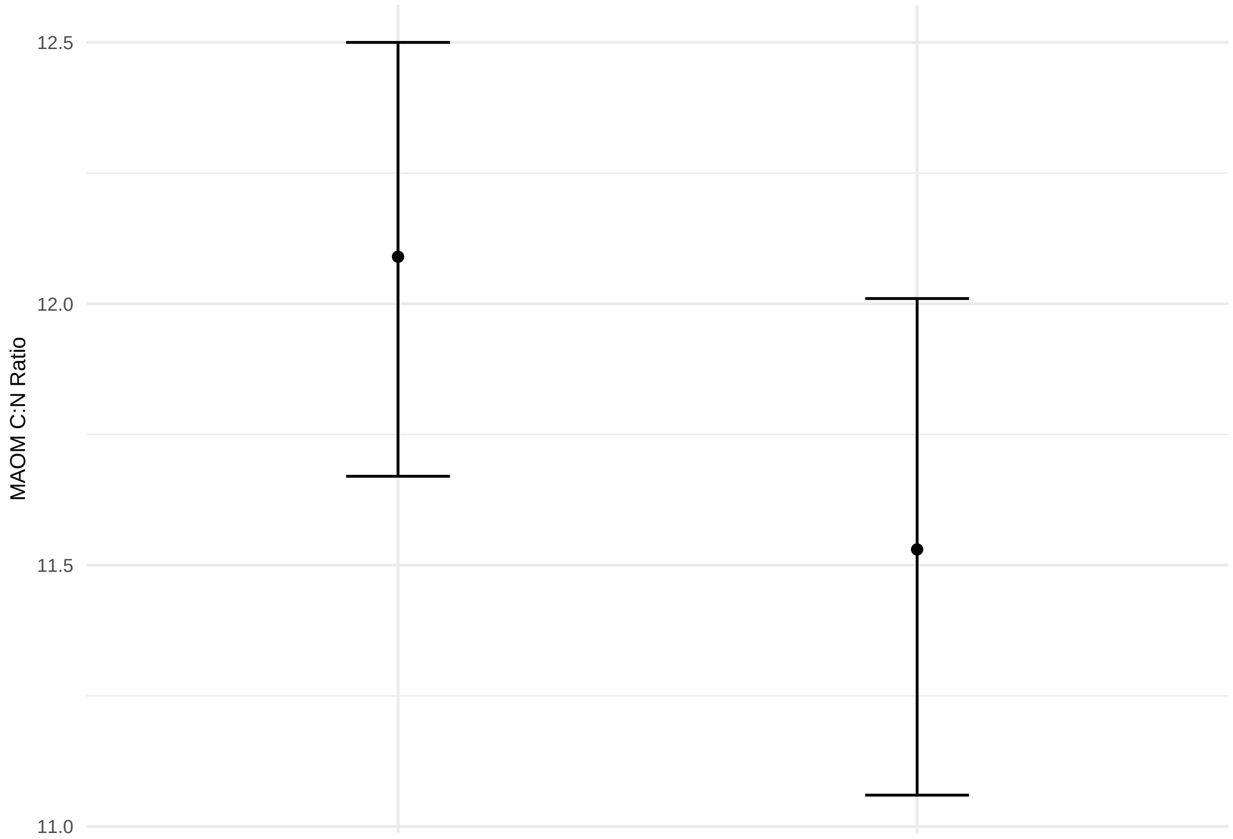


```
## POMratio
POMratiodata <- data.frame(
  Treatment = c("Ungrazed", "Grazed"),
  Mean = c(47.81, 49.78),
  LowerCI = c(38.32, 42.8),
  UpperCI = c(57.3, 56.76)
)
POMRatio_plot <- ggplot(POMratiodata, aes(x = Treatment, y = Mean, ymin = LowerCI, ymax = UpperCI)) +
  geom_point() +  # This adds the mean points
  geom_errorbar(width = 0.2) +  # This adds the error bars
  labs(
       y = "POM C:N Ratio") +
  theme_minimal() + theme(axis.title.x = element_blank(), axis.text.x = element_blank(),axis.title.y = 
print(POMRatio_plot)
```

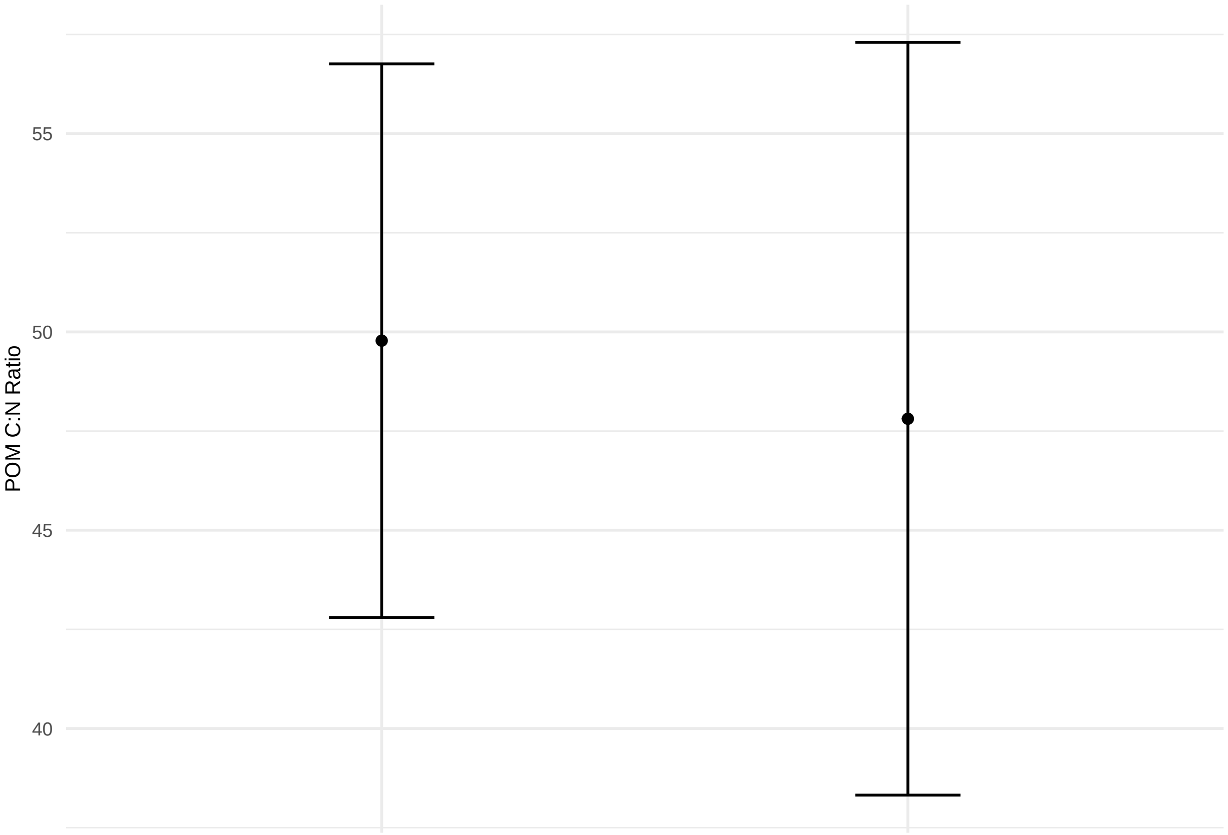


```r
## SOCratio
Totalratiodata <- data.frame(
  Treatment = c("Ungrazed", "Grazed"),
  Mean = c(14.40, 15.52),
  LowerCI = c(13.21, 14.04),
  UpperCI = c(15.59, 16.70)
)
TotalRatio_plot <- ggplot(Totalratiodata, aes(x = Treatment, y = Mean, ymin = LowerCI, ymax = UpperCI))
  geom_point() +  # This adds the mean points
  geom_errorbar(width = 0.2) +  # This adds the error bars
  labs(
       y = "SOC C:N Ratio") +
  theme_minimal() + theme(axis.title.x = element_blank(), axis.text.x = element_blank(),axis.title.y = e
print(TotalRatio_plot)
```

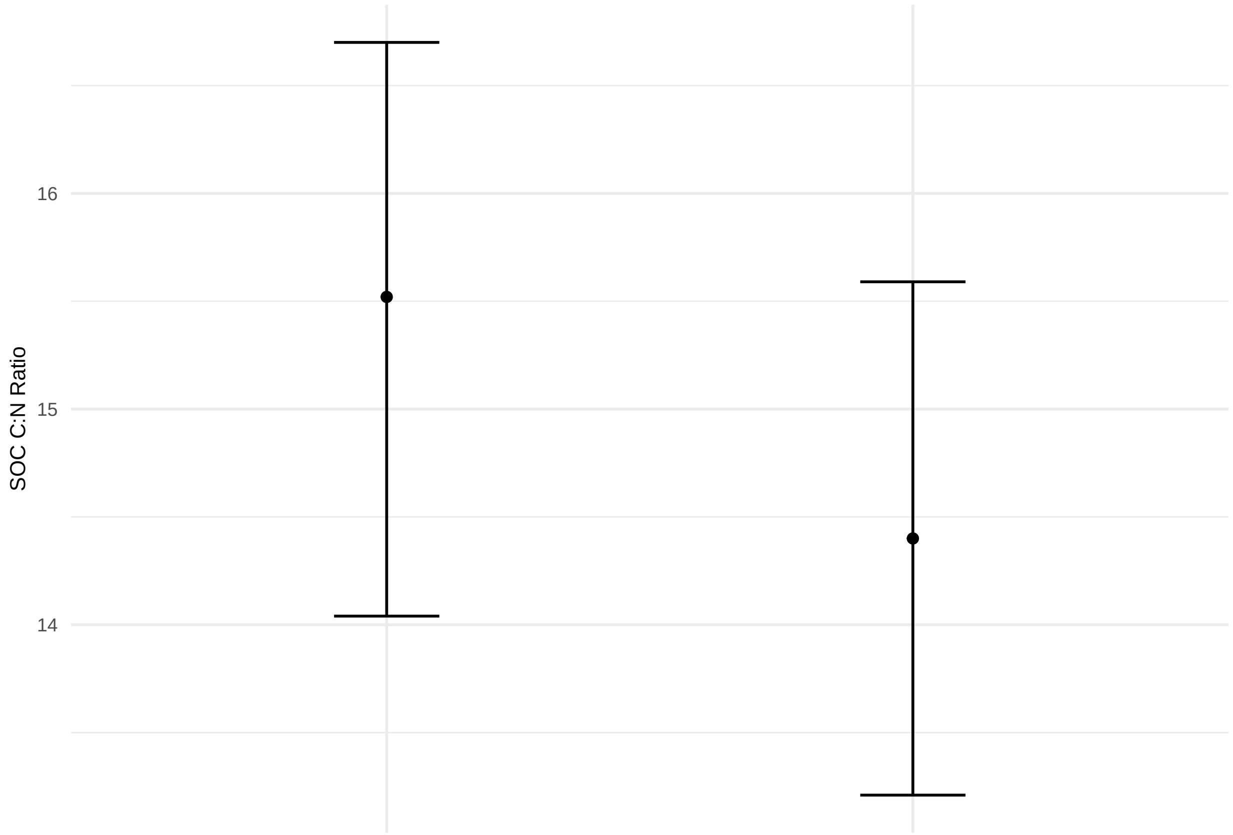


```
## Inorganic C
Inorganicdata <- data.frame(
  Treatment = c("Ungrazed", "Grazed"),
  Mean = c(4.77, 5.16),
  LowerCI = c(3.27, 4.12),
  UpperCI = c(6.27, 6.20)
)
Inorganicplot <- ggplot(Inorganicdata, aes(x = Treatment, y = Mean, ymin = LowerCI, ymax = UpperCI)) +
  geom_point() +  # This adds the mean points
  geom_errorbar(width = 0.2) +  # This adds the error bars
  labs(x = "Treatment",
       y = "Inorganic C Stocks (Mg C/ha)") +
  theme_minimal() + theme(axis.title.x = element_text(size=8), axis.text.x = element_text(size = 7),axis


## pH
pHdata <- data.frame(
  Treatment = c("Ungrazed", "Grazed"),
  Mean = c(7.57, 7.65),
  LowerCI = c(7.46, 7.58),
  UpperCI = c(7.67, 7.71)
)
pHplot <- ggplot(pHdata, aes(x = Treatment, y = Mean, ymin = LowerCI, ymax = UpperCI)) +
  geom_point() +  # This adds the mean points
  geom_errorbar(width = 0.2) +  # This adds the error bars
  labs(
       x = "Treatment",
```

```
        y = "pH") +
  theme_minimal() + theme(axis.title.x = element_text(size=8), axis.text.x = element_text(size = 7),axi
print(pHplot)
```

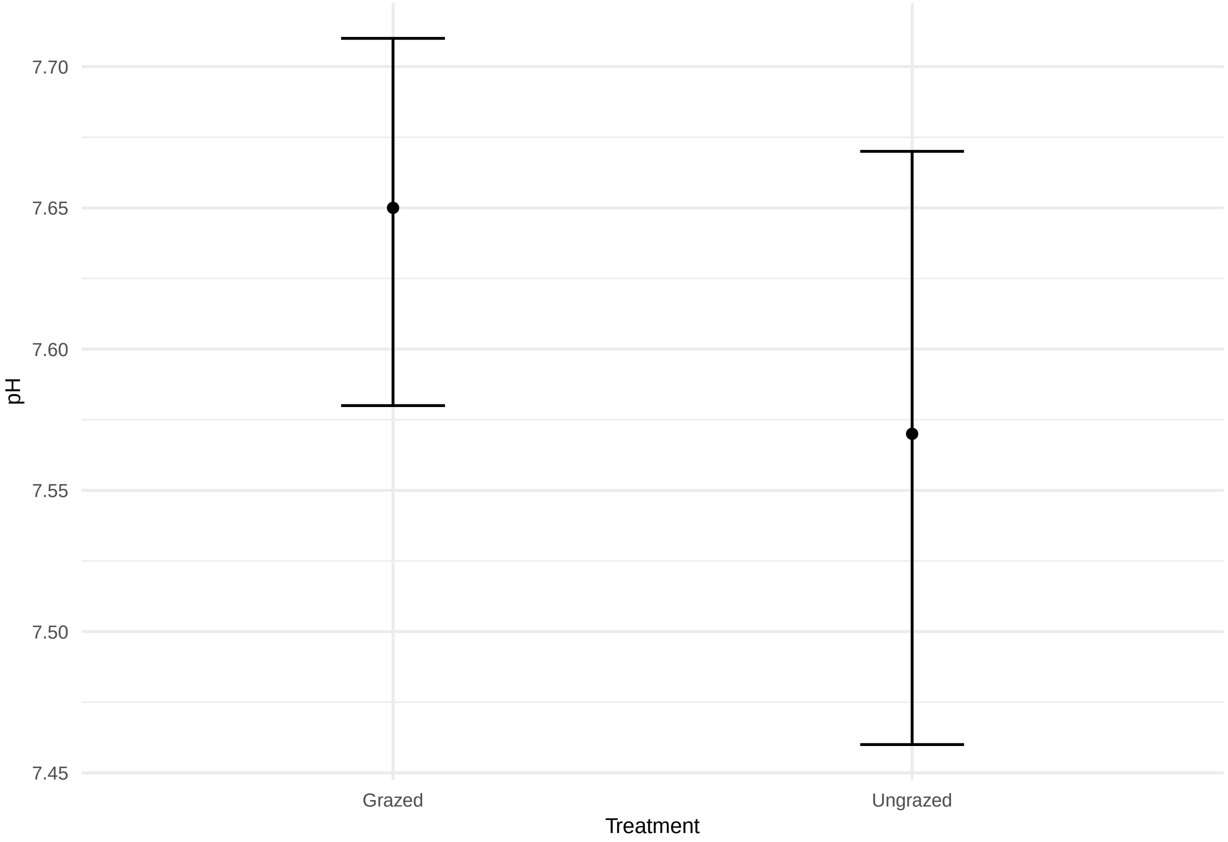


```
## BD
BDdata <- data.frame(
  Treatment = c("Ungrazed", "Grazed"),
  Mean = c(0.78, 0.78),
  LowerCI = c(0.71, 0.73),
  UpperCI = c(0.86, 0.83)
)
BDplot <- ggplot(BDdata, aes(x = Treatment, y = Mean, ymin = LowerCI, ymax = UpperCI)) +
  geom_point() +  # This adds the mean points
  geom_errorbar(width = 0.2) +  # This adds the error bars
  labs(
        x = "Treatment",
        y = "Bulk density (g/cm3)") +
  theme_minimal() + theme(axis.title.x = element_text(size=8), axis.text.x = element_text(size = 7),axi
print(BDplot)
```

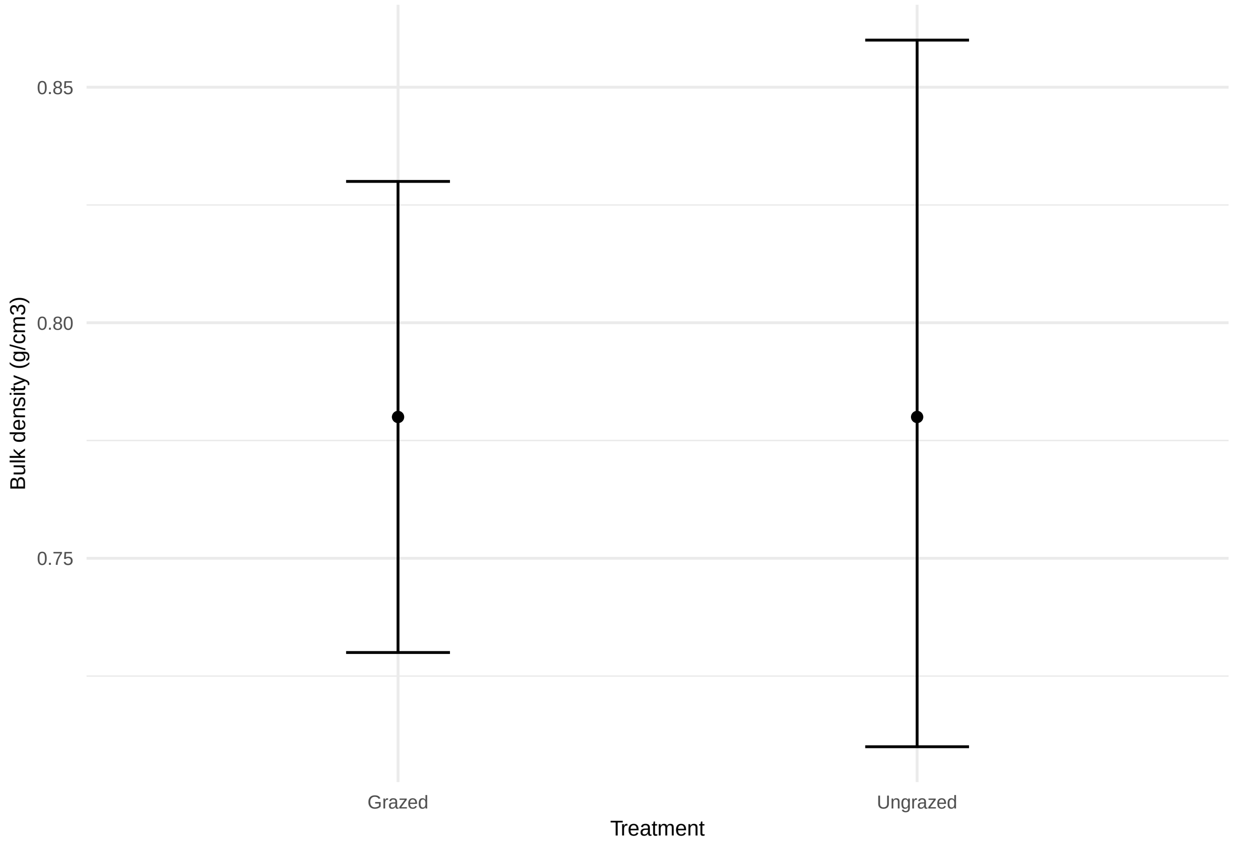


```
library(cowplot)
figure <- plot_grid(MAOMplot, POMplot, SOCplot, MAOM_N_plot, POM_N_plot, Total_N_plot, MAOMRatio_plot, I
print(figure)
```

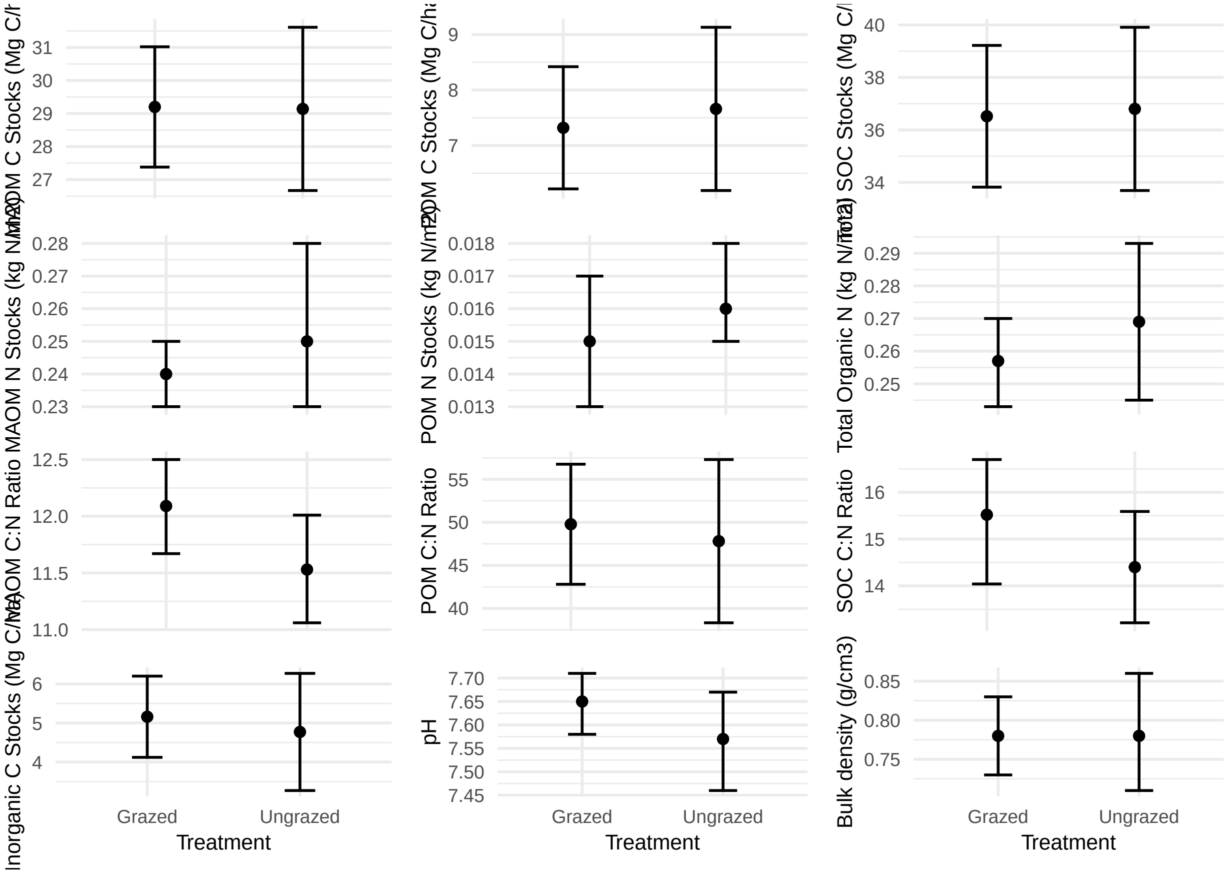
MAOM C Stocks (Mg C/ha)
POM C Stocks (Mg C/ha)
Total SOC Stocks (Mg C/ha)
MAOM N Stocks (kg N/m2)
POM N Stocks (kg N/m2)
Total Organic N (kg N/m2)
MAOM C:N Ratio
POM C:N Ratio
SOC C:N Ratio
Inorganic C Stocks (Mg C/ha)
pH
Bulk density (g/cm3)
Grazed
Ungrazed
Treatment
